# Supplementary material for: Visible light-induced chemoselective 1,2-diheteroarylation of alkenes
Source: Nat Commun. 2024 Jul 19;15:6102. doi: 10.1038/s41467-024-50460-4 (PMC11271625; doi:10.1038/s41467-024-50460-4)
Supplement: Supplementary file 1 — Supplementary Information [file 41467_2024_50460_MOESM1_ESM.pdf]

Supplementary Information for:

Visible Light-Induced Chemoselective 1,2-Diheteroarylation of Alkenes

Shi-Yu Guo,[1] Yi-Peng Liu,[1] Jin-Song Huang,[1] Li-Bowen He,[1,2] Gu-Cheng He,[1,2] Ding-Wei Ji,[1] Boshun Wan[1] and Qing-An Chen[1,2,*]

[1]*Dalian Institute of Chemical Physics, Chinese Academy of Sciences,*
457 Zhongshan Road, Dalian 116023, China
[2]*University of Chinese Academy of Sciences, Beijing 100049, China*
E-mail: qachen@dicp.ac.cn

Table of Contents

1. Supplementary Note 1 .... S2

2. Supplementary Note 2 .... S2

3. Supplementary Note 3 .... S7

4. Supplementary Method S8

5. Supplementary Note 4 .... S9

6. Supplementary Note 5 .... S22

7. Supplementary Discussion S27

8. Supplementary Note 6 .... S45

9. Supplementary Note 7 .... S110

10. Supplementary References S111

1. Supplementary Note 1

General experimental details:

Commercially available reagents were used without further purification. Solvents were treated prior to use according to the standard methods. Unless otherwise stated, all reactions were conducted under inert atmosphere using standard Schlenk techniques or in a nitrogen-filled glove-box. **^{1}H NMR** and **^{13}C NMR** spectra were recorded at room temperature in $CDCl_3$ on 400 or 700 MHz instruments with tetramethylsilane (TMS) as internal standard. Flash column chromatography was performed on silica gel (200-300 mesh). All reactions were monitored by TLC, NMR or GC-FID analysis. **HRMS** data was obtained with Micromass HPLC-Q-TOF mass spectrometer (ESI) or Agilent 6540 Accurate-MS spectrometer (Q-TOF).

2. Supplementary Note 2

Optimization studies of reaction conditions:

Supplementary Table 1. Influence of the photocatalysts[a]

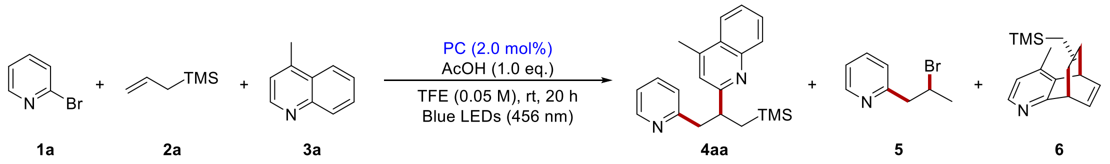

Entry	Photocatalyst	Yield of product (%)		
		**4aa**	**5**[b]	**6**
1	Ir(ppy)$_2$(dtbbpy)PF$_6$ (Ir-I)	90	Trace	5
2	Ir[dF(CF$_3$)ppy]$_2$(dtbbpy)PF$_6$ (Ir-III)	1	0	53
3	Ir[dF(Me)ppy]$_2$(dtbbpy)PF$_6$ (Ir-IV)	3	0	62
4	*fac*-Ir(ppy)$_3$ (Ir-II)	0	0	0
5	Ru(phen)$_3$(PF$_6$)$_2$	0	0	0
6	Ru(bpy)$_3$(PF$_6$)$_2$	0	0	0
7	Ru(dtbbpy)$_3$(PF$_6$)$_2$	0	0	0
8	Eosin Y	0	0	0
9	4CzIPN	0	0	0
10	Thioxanthone	24	Trace	11

[a] Reaction conditions: **1a** (0.20 mmol), **2a** (0.50 mmol), **3a** (0.10 mmol), PC (2.0 mol%), AcOH (0.10 mmol), TFE (2.0 mL), blue LEDs (λ_{max} = 456 nm), room temperature, N_2, 20 h, GC-FID yields of the crude reaction mixture using 1,3,5-trimethoxybenzene as internal standard. [b] Yields were calculated based on **1a**.

Supplementary Table 2. Influence of the wavelength of Kessil light[a]

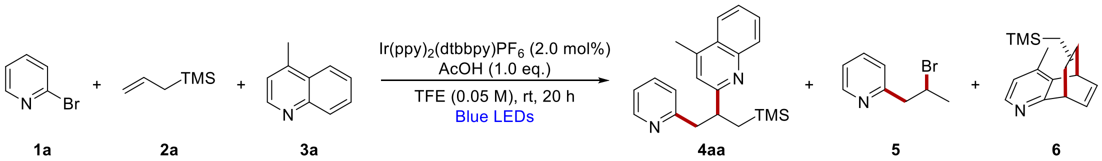

Entry	Wavelength of Kessil light	Yield of product (%)		
		**4aa**	**5**[b]	**6**
1	390 nm	81	Trace	4
2	427 nm	84	Trace	0
3	456 nm	91	Trace	5

[a] Reaction conditions: **1a** (0.20 mmol), **2a** (0.50 mmol), **3a** (0.10 mmol), $Ir(ppy)_2(dtbbpy)PF_6$ (2.0 mol%), AcOH (0.10 mmol), TFE (0.05 M), blue LEDs, room temperature, N_2, 20 h, GC-FID yields of the crude reaction mixture using 1,3,5-trimethoxybenzene as internal standard. [b] Yields were calculated based on **1a**.

Supplementary Table 3. Influence of the solvents[a]

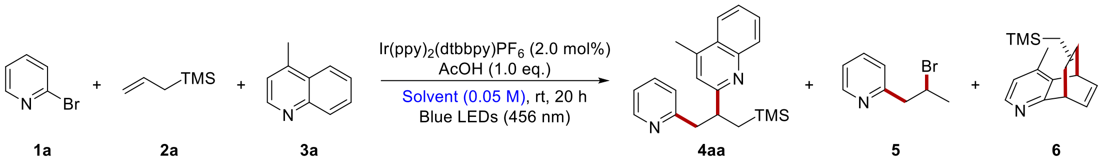

Entry	Solvent	Yield of product (%)		
		**4aa**	**5**[b]	**6**
1	TFE	90	Trace	5
2	HFIP	13	Trace	Trace
3	MeOH	3	0	0
4	EtOH	1	0	0
5	*i*-PrOH	0	0	0
6	DMSO	0	0	0
7	1,4-Dioxane	0	0	0
8	DMF	3	0	0
9	DCM	3	Trace	0
10	Toluene	0	0	0
11	MeCN	3	Trace	0

[a] Reaction conditions: **1a** (0.20 mmol), **2a** (0.50 mmol), **3a** (0.10 mmol), $Ir(ppy)_2(dtbbpy)PF_6$ (2.0 mol%), AcOH (0.10 mmol), solvent (0.05 M), blue LEDs (λ_{max} = 456 nm), room temperature, N_2, 20 h, GC-FID yields of the crude reaction mixture using 1,3,5-trimethoxybenzene as internal standard. [b] Yields were calculated based on **1a**.

Supplementary Table 4. Influence of the acids[a]

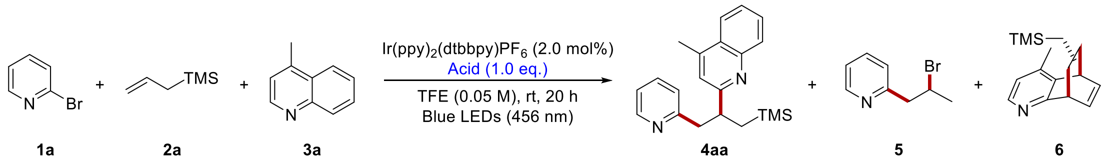

Entry	Acid	Yield of product (%)		
		**4aa**	**5**[b]	**6**
1	TsOH	18	Trace	3
2	TFA	26	Trace	5
3	$(PhO)_2PO_2H$	32	Trace	3
4	MCAA	63	Trace	7
5	HCO_2H	75	Trace	6
6	BzOH	87	Trace	4
7	AcOH	90	Trace	5
8	$C_2H_5CO_2H$	91	Trace	4
9	PivOH	85	Trace	5
10	NH_4Cl	58	Trace	3

[a] Reaction conditions: **1a** (0.20 mmol), **2a** (0.50 mmol), **3a** (0.10 mmol), $Ir(ppy)_2(dtbbpy)PF_6$ (2.0 mol%), Acid (0.10 mmol), TFE (0.05 M), blue LEDs (λ_{max} = 456 nm), room temperature, N_2, 20 h, GC-FID yields of the crude reaction mixture using 1,3,5-trimethoxybenzene as internal standard. [b] Yields were calculated based on **1a**.

Supplementary Table 5. Influence of the reaction concentration[a]

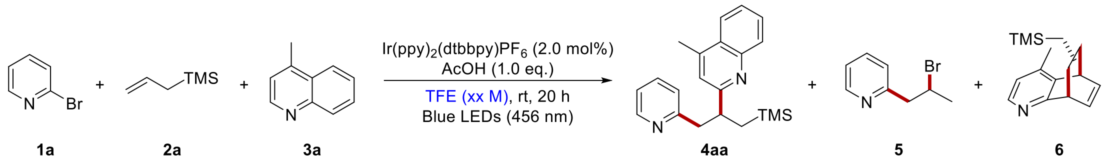

Entry	TFE (mL)	Yield of product (%)		
		**4aa**	**5**[b]	**6**
1	0.5	35	Trace	5
2	1.0	59	Trace	5
3	2.0	90	Trace	5
4	3.0	89	Trace	5
5	4.0	91	Trace	5

[a] Reaction conditions: **1a** (0.20 mmol), **2a** (0.50 mmol), **3a** (0.10 mmol), $Ir(ppy)_2(dtbbpy)PF_6$ (2.0 mol%), AcOH (0.10 mmol), TFE (xx M), blue LEDs (λ_{max} = 456 nm), room temperature, N_2, 20 h, GC-FID yields of the crude reaction mixture using 1,3,5-trimethoxybenzene as internal standard. [b] Yields were calculated based on **1a**.

Supplementary Table 6. Influence of the catalysts dosage[a]

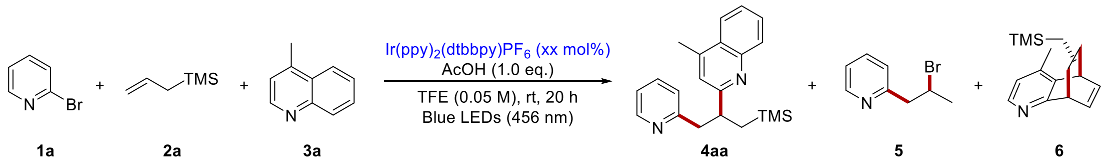

Entry	$Ir(ppy)_2(dtbbpy)PF_6$ (mol%)	Yield of product (%)		
		**4aa**	**5**[b]	**6**
1	0.01	3	Trace	0
2	0.05	22	Trace	3
3	0.1	86	Trace	2
4	0.5	93	Trace	4
5	1.0	92	Trace	4
6	2.0	90	Trace	5
7	3.0	86	Trace	7

[a] Reaction conditions: **1a** (0.20 mmol), **2a** (0.50 mmol), **3a** (0.10 mmol), $Ir(ppy)_2(dtbbpy)PF_6$ (xx mol%), AcOH (0.10 mmol), TFE (0.05 M), blue LEDs (λ_{max} = 456 nm), room temperature, N_2, 20 h, GC-FID yields of the crude reaction mixture using 1,3,5-trimethoxybenzene as internal standard. [b] Yields were calculated based on **1a**.

Supplementary Table 7. Influence of the alkenes dosage[a]

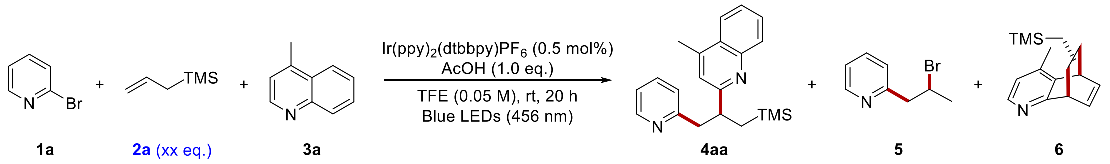

Entry	Alkene **2a** (eq.)	Yield of product (%)		
		**4aa**	**5**[b]	**6**
1	2.0	59	Trace	3
2	3.0	76	Trace	4
3	4.0	87	Trace	4
4	5.0	93	Trace	4

[a] Reaction conditions: **1a** (0.20 mmol), **2a** (xx mmol), **3a** (0.10 mmol), $Ir(ppy)_2(dtbbpy)PF_6$ (0.5 mol%), AcOH (0.10 mmol), TFE (0.05 M), blue LEDs (λ_{max} = 456 nm), room temperature, N_2, 20 h, GC-FID yields of the crude reaction mixture using 1,3,5-trimethoxybenzene as internal standard. [b] Yields were calculated based on **1a**.

Supplementary Table 8. Control experiments[a]

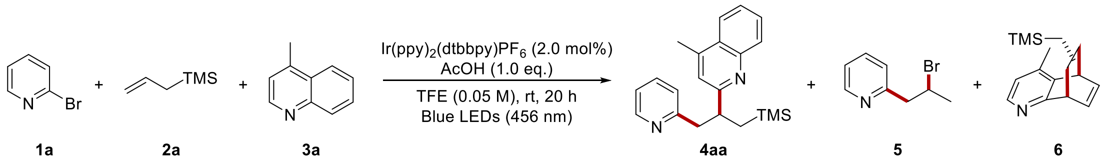

Entry	Deviation from standard conditions	Yield of product (%)		
		**4aa**	**5**[b]	**6**
1	None	90	Trace	5
2	Air instead of N_2	Trace	Trace	0
3	10 eq. of H_2O added	89	Trace	5
4	No PC	0	0	0
5	No AcOH	20	0	3
6	in Dark	0	0	0

[a] Reaction conditions: **1a** (0.20 mmol), **2a** (0.50 mmol), **3a** (0.10 mmol), $Ir(ppy)_2(dtbbpy)PF_6$ (2.0 mol%), AcOH (0.10 mmol), TFE (0.05 M), blue LEDs (λ_{max} = 456 nm), room temperature, N_2, 20 h, GC-FID yields of the crude reaction mixture using 1,3,5-trimethoxybenzene as internal standard. [b] Yields were calculated based on **1a**.

Comments on the solvent effect:

TFE as a strongly polar protic solvent could take a strong hydrogen bonding interaction with pyridines and quinolines. Besides, the acidity of strong protonic solvent could also increase electrophilicity of pyridine radical and aza-arenes. Moreover, TFE could also effectively increase the redox potential of aza-arenes to be more easily reduced by the photocatalyst. Hence, the model reaction could also provide 20% yield of product without the use of a Brønsted acid, perhaps owing to the acidity of strong protonic solvent TFE (Supplementary Table 8, entry 5).

3. Supplementary Note 3

Unsuccessful substrates:

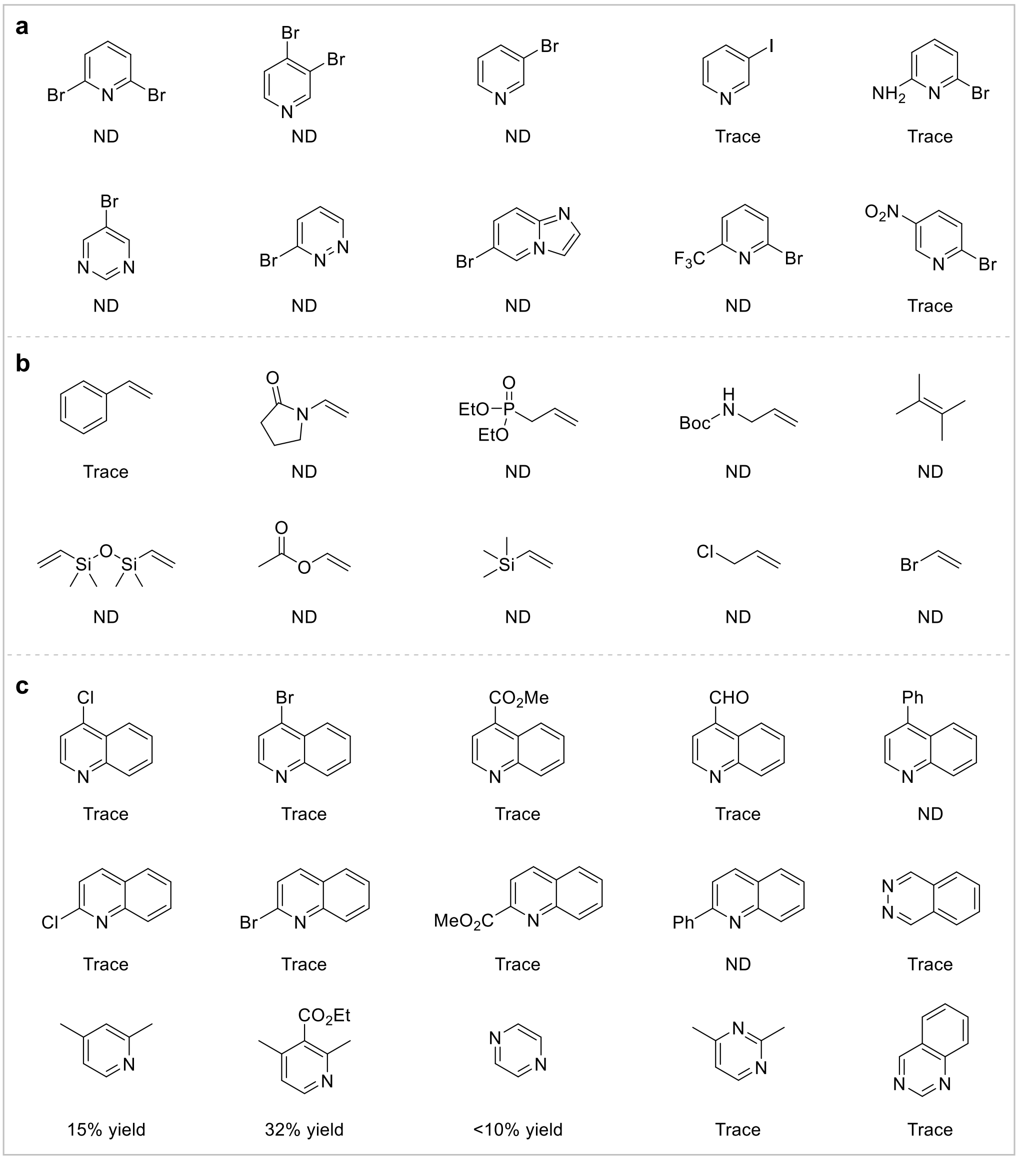

Supplementary Figure 1. Unsuccessful substrates. **a**, Heteroaryl bromide substrates. **b**, Alkenes substrates. **c**, Aza-arene substrates.

Comments on unsuccessful substrates:

a) Polyhalogenated (2,6 and 3,4-dibromo) pyridines and 3-Br/I substituted pyridines were not suitable for this standard conditions. Amino group was intolerant in this reaction. Some other halogenated aza-arenes like pyrimidine, pyridazine *etc.* couldn't be activated under standard conditions.

b) The generated pyridyl radical is electrophilic under our conditions, so styrene or alkenes with electron-withdrawing substituents were not suitable for this reaction. α-Functionalized alkenes were also not feasible, properly owing to the instability of the radical intermediate or steric hindrance problem.

c) Phenyl, ester and halo groups substituted quinolines were not suitable for this reaction. Because electron-withdrawing substituents on *N*-containing aromatic rings might alter the redox capacity of the quinoline, thereby disrupting the electron transfer. Other aza-arenes like pyridine, pyrazine, pyrimidine etc. were also reactive, but afforded low products yields compared with quinolines.

4. Supplementary Method

General procedure of proton-coupled electron shuttle for chemoselective 1,2-diheteroarylation of alkenes under photocatalysis:

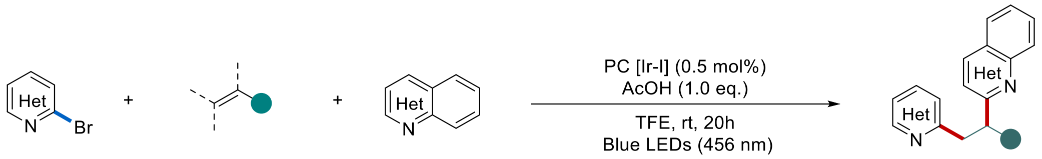

To an oven-dried 20 mL vial was added Ir(ppy)$_2$(dtbbpy)PF$_6$ (0.002 mmol, 0.5 mol%), bromopyridine **1** (0.80 mmol, 2.0 eq.), alkene **2** (2.0 ~ 2.8 mmol, 5.0 ~ 7.0 eq.), quinoline **3** (0.40 mmol, 1.0 eq.), AcOH (0.40 mmol, 1.0 eq.) and TFE (8.0 mL, 0.05 M) in the nitrogen glove box. The vial was capped with a septum and wrapped with parafilm. The reaction mixture was stirred for 20 h under visible light irradiation (Kessil PR160, λ_{max}= 456 nm, 40 W, irradiation temperature maintained between 25-30 ˚C). Upon completion, the crude product was neutralized with saturated $NaHCO_3$ solution or Et_3N and extracted with ethyl acetate. Organic layer was washed with brine solution and dried over anhydrous Na_2SO_4. Removal of the organic solvent in a vacuum rotavapor followed by flash silica gel column chromatographic purification affords the desired products **4** and **7** in moderate to good yields.

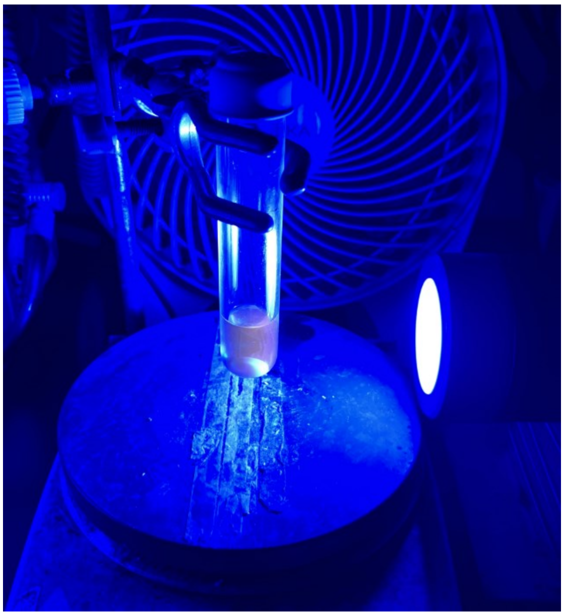

Supplementary Figure 2. Reaction setups.

5. Supplementary Note 4

Analytical data for the 1,2-diheteroarylation products:

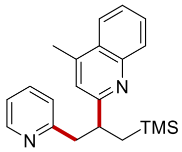

4-Methyl-2-(1-(pyridin-2-yl)-3-(trimethylsilyl)propan-2-yl)quinoline (4aa): Colorless oil, 120.4 mg, 90% yield, PE/EA = 6/1. **^{1}H NMR** (400 MHz, $CDCl_3$) δ 8.52 – 8.48 (m, 1H), 8.04 (dd, *J* = 8.4, 0.5 Hz, 1H), 7.90 (dd, *J* = 8.3, 1.0 Hz, 1H), 7.67 – 7.61 (m, 1H), 7.50 – 7.44 (m, 1H), 7.42 – 7.36 (m, 1H), 7.04 (d, *J* = 0.5 Hz, 1H), 7.02 – 6.98 (m, 1H), 6.93 (d, *J* = 7.8 Hz, 1H), 3.63 – 3.53 (m, 1H), 3.32 (dd, *J* = 13.3, 7.9 Hz, 1H), 3.15 (dd, *J* = 13.3, 7.1 Hz, 1H), 2.60 (d, *J* = 0.7 Hz, 3H), 1.36 (dd, *J* = 14.6, 10.5 Hz, 1H), 0.98 (dd, *J* = 14.6, 4.6 Hz, 1H), -0.23 (s, 9H). **^{13}C NMR** (100 MHz, $CDCl_3$) δ 165.51, 160.67, 149.26, 147.83, 143.93, 136.03, 129.71, 128.88, 127.13, 125.47, 123.90, 123.69, 122.01, 121.06, 47.88, 45.17, 22.91, 18.75, -0.92. **HRMS** (m/z) $[M+H]^+$ calcd. for $C_{21}H_{27}N_2Si$, 335.1944, found 335.1947.

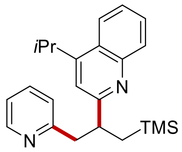

4-Isopropyl-2-(1-(pyridin-2-yl)-3-(trimethylsilyl)propan-2-yl)quinoline (4ab): Colorless oil, 126.2 mg, 87% yield, PE/EA = 6/1. **^{1}H NMR** (400 MHz, $CDCl_3$) δ 8.50 (d, *J* = 4.6 Hz, 1H), 8.06 (d, *J* = 8.4 Hz, 1H), 7.97 (d, *J* = 8.3 Hz, 1H), 7.64 – 7.57 (m, 1H), 7.46 – 7.40 (m, 1H), 7.36 – 7.30 (m, 1H), 7.03 – 6.94 (m, 2H), 6.83 (d, *J* = 7.8 Hz, 1H), 3.66 – 3.54 (m, 2H), 3.28 (dd, *J* = 13.1, 7.9 Hz, 1H), 3.18 (dd, *J* = 13.1, 7.2 Hz, 1H), 1.41 (dd, *J* = 14.5, 10.9 Hz, 1H), 1.28 (d, *J* = 6.8 Hz, 3H), 1.21 (d, *J* = 6.8 Hz, 3H), 0.98 (dd, *J* = 14.5, 4.3 Hz, 1H), -0.27 (s, 9H). **^{13}C NMR** (100 MHz, $CDCl_3$) δ 165.35, 160.61, 153.79, 149.19, 148.24, 135.95, 130.00, 128.50, 125.57, 125.31, 123.93, 122.88, 120.95, 117.41, 48.06, 45.37, 28.16, 22.99, 22.89, 22.58, -0.98. **HRMS** (m/z) $[M+H]^+$ calcd. for $C_{23}H_{31}N_2Si$, 363.2257, found 363.2261.

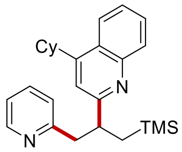

4-Cyclohexyl-2-(1-(pyridin-2-yl)-3-(trimethylsilyl)propan-2-yl)quinoline (4ac): Colorless oil, 136.9 mg, 85% yield, PE/EA = 6/1. **^{1}H NMR** (400 MHz, $CDCl_3$) δ 8.49 (d, *J* = 4.8 Hz, 1H), 8.05 (d, *J* = 7.8 Hz, 1H), 7.95 (d, *J* = 8.4 Hz, 1H), 7.58 (t, *J* = 7.6 Hz, 1H), 7.41 (t, *J* = 7.6 Hz, 1H), 7.35 – 7.28 (m, 1H), 7.00 (s, 1H), 6.97 – 6.90 (m, 1H), 6.83 (d, *J* = 7.8 Hz, 1H), 3.66 – 3.53 (m, 1H), 3.28 (dd, *J* = 13.1, 7.8 Hz, 1H), 3.23 – 3.11 (m, 2H), 1.93 – 1.74 (m, 5H), 1.53 – 1.36 (m, 4H), 1.35 – 1.23 (m, 2H), 0.97 (dd, *J* = 14.5, 4.3 Hz, 1H), -0.28 (s, 9H). **^{13}C NMR** (100 MHz, $CDCl_3$) δ 165.27, 160.57, 152.77, 149.13, 148.20, 135.90, 129.96, 128.42, 125.54, 125.20, 123.85, 122.76, 120.89, 117.89, 47.99, 45.33, 38.71, 33.55, 33.46, 26.89, 26.27, 22.59, -1.02. **HRMS** (m/z) $[M+H]^+$ calcd. for $C_{26}H_{35}N_2Si$, 403.2570, found 403.2575.

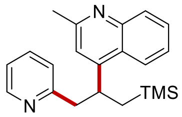

2-Methyl-4-(1-(pyridin-2-yl)-3-(trimethylsilyl)propan-2-yl)quinoline (4ad): Colorless oil, 121.8 mg, 91% yield, PE/EA = 6/1. **^{1}H NMR** (400 MHz, $CDCl_3$) δ 8.48 (dd, *J* = 4.8, 0.8 Hz, 1H), 8.07 – 7.92 (m, 2H), 7.61 – 7.53 (m, 1H), 7.37 (t, *J* = 7.3 Hz, 1H), 7.31 (td, *J* = 7.6, 1.8 Hz, 1H), 7.26 – 7.18 (m, 1H), 6.99 – 6.92 (m, 1H), 6.72 (d, *J* = 7.7 Hz, 1H), 4.17 (s, 1H), 3.13 (d, *J* = 6.9 Hz, 2H), 2.70 (s, 3H), 1.31 – 1.20 (m, 1H), 1.10 (dd, *J* = 14.8, 4.8 Hz, 1H), -0.25 (s, 9H). **^{13}C NMR** (175 MHz, $CDCl_3$) δ 159.87, 158.51, 153.04, 149.60, 148.28, 136.14, 129.49, 129.14, 125.63, 125.55, 123.78, 123.13, 121.43, 119.89, 49.35, 35.38, 25.66, 23.51, -0.65. **HRMS** (m/z) $[M+H]^+$ calcd. for $C_{21}H_{27}N_2Si$, 335.1944, found 335.1946.

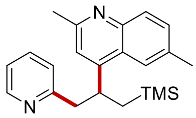

2,6-Dimethyl-4-(1-(pyridin-2-yl)-3-(trimethylsilyl)propan-2-yl)quinoline (4ae): Colorless oil, 103.2 mg, 74% yield, PE/EA = 6/1. **^{1}H NMR** (400 MHz, $CDCl_3$) δ 8.49 (d, *J* = 4.8 Hz, 1H), 7.87 (d, *J* = 8.5 Hz, 1H), 7.68 (s, 1H), 7.40 (dd, *J* = 8.5, 1.1

Hz, 1H), 7.29 (dd, J = 7.6, 1.6 Hz, 1H), 7.21 (s, 1H), 6.99 – 6.93 (m, 1H), 6.67 (d, J = 7.7 Hz, 1H), 4.23 – 4.04 (m, 1H), 3.23 – 3.03 (m, 2H), 2.69 (s, 3H), 2.45 (s, 3H), 1.29 – 1.20 (m, 1H), 1.11 (dd, J = 14.8, 4.9 Hz, 1H), -0.23 (s, 9H). **^{13}C NMR** (100 MHz, $CDCl_3$) δ 159.74, 157.22, 152.47, 149.30, 146.30, 135.96, 135.05, 131.29, 128.74, 125.47, 123.64, 122.08, 121.28, 119.71, 49.15, 35.05, 25.22, 23.59, 21.94, -0.74. **HRMS** (m/z) $[M+H]^+$ calcd. for $C_{22}H_{29}N_2Si$, 349.2100, found 349.2105.

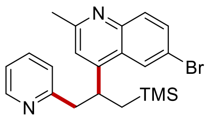

6-Bromo-2-methyl-4-(1-(pyridin-2-yl)-3-(trimethylsilyl)propan-2-yl)quinolie (4af): Colorless oil, 115.8 mg, 70% yield, PE/EA = 8/1. **^{1}H NMR** (700 MHz, $CDCl_3$) δ 8.51 (d, J = 4.1 Hz, 1H), 8.04 (s, 1H), 7.80 (d, J = 8.9 Hz, 1H), 7.62 (dd, J = 8.9, 1.8 Hz, 1H), 7.32 – 7.26 (m, 2H), 7.00 – 6.95 (m, 1H), 6.66 (d, J = 7.7 Hz, 1H), 4.14 – 3.99 (m, 1H), 3.21 (dd, J = 13.2, 6.0 Hz, 1H), 3.09 – 3.00 (m, 1H), 2.68 (s, 3H), 1.27 – 1.22 (m, 1H), 1.16 (dd, J = 14.9, 5.1 Hz, 1H), -0.20 (s, 9H). **^{13}C NMR** (175 MHz, $CDCl_3$) δ 159.44, 159.04, 152.50, 149.63, 146.71, 136.18, 132.53, 131.08, 126.97, 125.78, 123.64, 121.63, 120.65, 119.63, 49.36, 35.71, 25.62, 23.92, -0.57. **HRMS** (m/z) $[M+H]^+$ calcd. for $C_{21}H_{26}BrN_2Si$, 413.1049, found 413.1046.

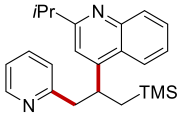

2-Isopropyl-4-(1-(pyridin-2-yl)-3-(trimethylsilyl)propan-2-yl)quinoline (4ag): Colorless oil, 123.3 mg, 85% yield, PE/EA = 6/1. **^{1}H NMR** (400 MHz, $CDCl_3$) δ 8.49 (d, J = 4.6 Hz, 1H), 8.16 – 7.90 (m, 2H), 7.58 (t, J = 7.5 Hz, 1H), 7.39 (t, J = 7.6 Hz, 1H), 7.33 – 7.18 (m, 2H), 7.00 – 6.91 (m, 1H), 6.69 (d, J = 7.7 Hz, 1H), 4.46 – 3.89 (m, 1H), 3.28 – 3.08 (m, 3H), 1.40 – 1.31 (m, 6H), 1.30 – 1.20 (m, 1H), 1.11 (dd, J = 14.8, 4.6 Hz, 1H), -0.26 (s, 9H). **^{13}C NMR** (100 MHz, $CDCl_3$) δ 166.93, 159.77, 152.60, 149.40, 148.07, 135.95, 129.80, 128.77, 125.89, 125.37, 123.63, 122.97, 121.23, 116.83, 48.95, 37.49, 35.47, 23.19, 22.67, 22.63, -0.78. **HRMS** (m/z) $[M+H]^+$ calcd. for $C_{23}H_{31}N_2Si$, 363.2257, found 363.2260.

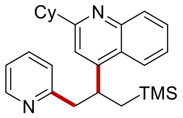

2-Cyclohexyl-4-(1-(pyridin-2-yl)-3-(trimethylsilyl)propan-2-yl)quinoline (4ah): Colorless oil, 135.5 mg, 84% yield, PE/EA = 6/1. **^{1}H NMR** (700 MHz, $CDCl_3$) δ 8.47 (d, J = 4.4 Hz, 1H), 8.09 – 7.90 (m, 2H), 7.58 – 7.52 (m, 1H), 7.39 – 7.34 (m, 1H), 7.31 – 7.18 (m, 2H), 6.95 – 6.90 (m, 1H), 6.66 (d, J = 7.5 Hz, 1H), 4.21 (s, 1H), 3.13 (s, 2H), 2.90 – 2.80 (m, 1H), 2.03 – 1.93 (m, 2H), 1.87 (d, J = 12.4 Hz, 2H), 1.76 (d, J = 12.6 Hz, 1H), 1.64 – 1.53 (m, 2H), 1.49 – 1.41 (m, 2H), 1.37 – 1.30 (m, 1H), 1.30 – 1.20 (m, 1H), 1.10 (d, J = 12.3 Hz, 1H), -0.28 (s, 9H). **^{13}C NM** (175 MHz, $CDCl_3$) δ 166.19, 159.86, 152.60, 149.48, 148.21, 136.03, 129.86, 128.84, 126.02, 125.41, 123.74, 122.96, 121.31, 117.30, 49.24, 47.98, 35.48, 33.06, 26.75, 26.30, 23.37, -0.67. **HRMS** (m/z) $[M+H]^+$ calcd. for $C_{26}H_{35}N_2Si$, 403.2570, found 403.2574.

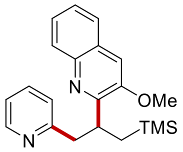

3-Methoxy-2-(1-(pyridin-2-yl)-3-(trimethylsilyl)propan-2-yl)quinoline (4ai): Colorless oil, 101.1 mg, 72% yield, >20:1 rr, PE/EA = 6/1. **^{1}H NMR** (400 MHz, $CDCl_3$) δ 8.49 (d, J = 4.6 Hz, 1H), 8.01 (d, J = 8.3 Hz, 1H), 7.63 (d, J = 7.9 Hz, 1H), 7.52 – 7.46 (m, 1H), 7.40 (t, J = 7.4 Hz, 1H), 7.34 (td, J = 7.6, 1.7 Hz, 1H), 7.16 (s, 1H), 6.98 – 6.93 (m, 1H), 6.87 (d, J = 7.8 Hz, 1H), 4.21 – 4.11 (m, 1H), 3.75 (s, 3H), 3.39 (dd, J = 13.0, 8.1 Hz, 1H), 3.13 (dd, J = 13.0, 6.8 Hz, 1H), 1.51 (dd, J = 14.4, 10.4 Hz, 1H), 0.94 (dd, J = 14.4, 4.5 Hz, 1H), -0.24 (s, 9H). **^{13}C NMR** (175 MHz, $CDCl_3$) δ 161.27, 158.99, 151.66, 149.18, 142.91, 135.82, 128.93, 128.22, 126.36, 126.32, 126.16, 123.93, 120.87, 111.15, 55.29, 46.90, 37.15, 22.09, -0.81. **HRMS** (m/z) $[M+H]^+$ calcd. for $C_{21}H_{27}N_2OSi$, 351.1893, found 351.1896.

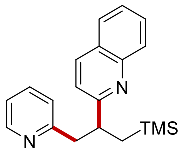

2-(1-(Pyridin-2-yl)-3-(trimethylsilyl)propan-2-yl)quinoline (4aj): Colorless oil, 33.5 mg, 26% yield, PE/EA = 4/1. **^{1}H NMR** (400 MHz, $CDCl_3$) δ 8.49 (d, *J* = 4.7 Hz, 1H), 8.06 (d, *J* = 8.5 Hz, 1H), 7.96 (d, *J* = 8.4 Hz, 1H), 7.73 (d, *J* = 8.1 Hz, 1H), 7.69 – 7.62 (m, 1H), 7.46 (t, *J* = 7.5 Hz, 1H), 7.39 (td, *J* = 7.7, 1.6 Hz, 1H), 7.19 (d, *J* = 8.4 Hz, 1H), 7.04 – 6.97 (m, 1H), 6.91 (d, *J* = 7.8 Hz, 1H), 3.71 – 3.61 (m, 1H), 3.35 (dd, *J* = 13.3, 8.2 Hz, 1H), 3.20 (dd, *J* = 13.3, 6.9 Hz, 1H), 1.40 (dd, *J* = 14.6, 10.6 Hz, 1H), 1.02 (dd, *J* = 14.6, 4.5 Hz, 1H), -0.24 (s, 9H). **^{13}C NMR** (175 MHz, $CDCl_3$) δ 165.93, 160.62, 149.30, 148.05, 136.35, 136.27, 129.44, 129.24, 127.75, 127.21, 125.94, 124.09, 121.57, 121.28, 48.09, 45.45, 23.34, -0.79. **HRMS** (m/z) $[M+H]^+$ calcd. for $C_{20}H_{25}N_2Si$, 321.1787, found 321.1789.

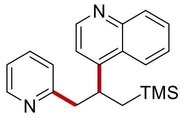

4-(1-(Pyridin-2-yl)-3-(trimethylsilyl)propan-2-yl)quinoline (4aj'): Colorless oil, 32.9 mg, 26% yield, PE/EA = 4/1. **^{1}H NMR** (400 MHz, $CDCl_3$) δ 8.83 (d, *J* = 4.4 Hz, 1H), 8.48 (dd, *J* = 4.8, 0.7 Hz, 1H), 8.15 – 7.99 (m, 2H), 7.66 – 7.59 (m, 1H), 7.45 (t, *J* = 7.3 Hz, 1H), 7.39 (s, 1H), 7.31 (td, *J* = 7.6, 1.8 Hz, 1H), 7.01 – 6.93 (m, 1H), 6.72 (d, *J* = 7.7 Hz, 1H), 4.24 (s, 1H), 3.26 – 3.08 (m, 2H), 1.34 – 1.26 (m, 1H), 1.14 (dd, *J* = 14.8, 4.8 Hz, 1H), -0.24 (s, 9H). **^{13}C NMR** (175 MHz, $CDCl_3$) δ 159.87, 158.51, 153.04, 149.60, 148.28, 136.14, 129.49, 129.14, 125.63, 125.55, 123.78, 123.13, 121.43, 119.89, 49.35, 35.38, 25.66, 23.51, -0.65. **HRMS** (m/z) $[M+H]^+$ calcd. for $C_{20}H_{25}N_2Si$, 321.1787, found 321.1792.

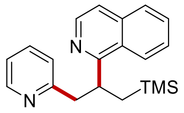

1-(1-(Pyridin-2-yl)-3-(trimethylsilyl)propan-2-yl)isoquinoline (4ak): Colorless oil, 61.5 mg, 48% yield, PE/EA = 5/1. **^{1}H NMR** (400 MHz, $CDCl_3$) δ 8.52 (d, J = 5.6 Hz, 1H), 8.50 – 8.46 (m, 1H), 8.15 (d, J = 8.5 Hz, 1H), 7.72 (d, J = 8.1 Hz, 1H), 7.59 – 7.53 (m, 1H), 7.49 – 7.41 (m, 2H), 7.25 (td, J = 7.6, 1.8 Hz, 1H), 6.96 – 6.87 (m, 1H), 6.70 (d, J = 7.7 Hz, 1H), 4.51 – 4.39 (m, 1H), 3.37 (dd, J = 12.9, 8.2 Hz, 1H), 3.20 (dd, J = 12.9, 6.5 Hz, 1H), 1.59 (dd, J = 14.4, 10.5 Hz, 1H), 1.03 (dd, J = 14.4, 4.3 Hz, 1H), -0.33 (s, 9H). **^{13}C NMR** (100 MHz, $CDCl_3$) δ 165.04, 160.64, 149.36, 141.93, 136.25, 135.82, 129.66, 127.28, 127.16, 126.88, 124.98, 123.99, 120.98, 119.17, 48.46, 38.10, 29.85, 23.16, -0.98. **HRMS** (m/z) $[M+H]^+$ calcd. for $C_{20}H_{25}N_2Si$, 321.1787, found 321.1791.

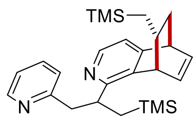

1-(1-(Pyridin-2-yl)-3-(trimethylsilyl)propan-2-yl)-9-((trimethylsilyl)methyl)-5,8-dihydro-8,5-ethanoisoquinoline (4ak'): Colorless oil, 59.1 mg, 34% yield, PE/EA = 8/1. **^{1}H NMR** (700 MHz, $CDCl_3$) δ 8.53 – 8.50 (m, 1H), 8.33 (d, J = 4.7 Hz, 1H), 7.23 (td, J = 7.6, 1.8 Hz, 1H), 6.96 – 6.93 (m, 1H), 6.85 (d, J = 4.7 Hz, 1H), 6.48 (d, J = 7.7 Hz, 1H), 6.11 – 6.07 (m, 1H), 5.94 – 5.90 (m, 1H), 3.93 – 3.87 (m, 1H), 3.71 (d, J = 6.3 Hz, 1H), 3.70 – 3.66 (m, 1H), 3.10 (dd, J = 12.9, 5.3 Hz, 1H), 3.06 (dd, J = 12.9, 9.7 Hz, 1H), 1.80 – 1.70 (m, 2H), 1.21 – 1.16 (m, 2H), 0.67 – 0.62 (m, 1H), 0.60 (dd, J = 14.3, 1.8 Hz, 1H), 0.01 (s, 9H), -0.12 (s, 9H), -0.18 (dd, J = 14.1, 12.3 Hz, 1H). **^{13}C NMR** (175 MHz, $CDCl_3$) δ 160.87, 159.70, 153.46, 149.25, 146.06, 137.06, 135.80, 134.35, 131.91, 123.89, 120.84, 115.70, 47.50, 44.98, 40.57, 37.71, 34.29, 33.88, 25.94, 23.05, -0.37, -0.75. **HRMS** (m/z) $[M+H]^+$ calcd. for $C_{26}H_{39}N_2Si_2$, 435.2646, found 435.2649.

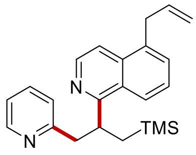

5-Allyl-1-(1-(pyridin-2-yl)-3-(trimethylsilyl)propan-2-yl)isoquinoline (4al): Colorless oil, 101.5 mg, 70% yield, PE/EA = 6/1. **^{1}H NMR** (400 MHz, $CDCl_3$) δ 8.53 (d, *J* = 5.9 Hz, 1H), 8.47 (d, *J* = 4.2 Hz, 1H), 8.08 (d, *J* = 8.2 Hz, 1H), 7.60 (d, *J* = 5.9 Hz, 1H), 7.44 – 7.35 (m, 2H), 7.28 – 7.23 (m, 1H), 6.94 – 6.88 (m, 1H), 6.73

(d, J = 7.7 Hz, 1H), 6.11 – 5.98 (m, 1H), 5.10 (dd, J = 10.1, 1.4 Hz, 1H), 4.98 (dd, J = 17.1, 1.6 Hz, 1H), 4.52 – 4.41 (m, 1H), 3.74 (d, J = 6.2 Hz, 2H), 3.39 (dd, J = 13.0, 8.0 Hz, 1H), 3.18 (dd, J = 13.0, 6.6 Hz, 1H), 1.58 (dd, J = 14.4, 10.4 Hz, 1H), 1.02 (dd, J = 14.4, 4.4 Hz, 1H), -0.33 (s, 9H). **^{13}C NMR** (175 MHz, $CDCl_3$) δ 165.69, 160.78, 149.46, 141.93, 136.54, 135.95, 135.82, 135.28, 129.99, 127.47, 126.61, 124.15, 123.79, 121.11, 116.87, 115.53, 48.47, 38.33, 37.07, 23.28, -0.85. **HRMS** (m/z) $[M+H]^+$ calcd. for $C_{23}H_{29}N_2Si$, 361.2100, found 361.2103.

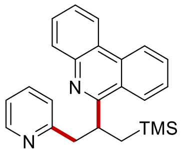

6-(1-(Pyridin-2-yl)-3-(trimethylsilyl)propan-2-yl)phenanthridine (4am): Colorless oil, 136.5 mg, 92% yield, PE/EA = 6/1. **^{1}H NMR** (400 MHz, $CDCl_3$) δ 8.59 (d, J = 8.2 Hz, 1H), 8.55 – 8.48 (m, 2H), 8.35 (d, J = 8.3 Hz, 1H), 8.16 (dd, J = 8.1, 0.9 Hz, 1H), 7.79 – 7.68 (m, 2H), 7.65 – 7.56 (m, 2H), 7.29 (td, J = 7.6, 1.8 Hz, 1H), 6.98 – 6.92 (m, 1H), 6.89 (d, J = 7.7 Hz, 1H), 4.63 – 4.49 (m, 1H), 3.58 (dd, J = 13.1, 7.6 Hz, 1H), 3.19 (dd, J = 13.1, 6.7 Hz, 1H), 1.75 (dd, J = 14.4, 9.6 Hz, 1H), 1.04 (dd, J = 14.4, 4.8 Hz, 1H), -0.24 (s, 9H). **^{13}C NMR** (100 MHz, $CDCl_3$) δ 164.75, 160.96, 149.34, 143.81, 135.88, 132.89, 130.03, 129.91, 128.47, 127.24, 126.30, 125.87, 125.34, 124.26, 123.48, 122.43, 122.00, 120.99, 47.32, 38.73, 22.44, -0.64. **HRMS** (m/z) $[M+H]^+$ calcd. for $C_{24}H_{27}N_2Si$, 371.1944, found 371.1944.

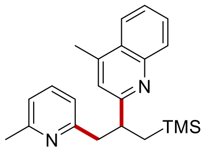

4-Methyl-2-(1-(6-methylpyridin-2-yl)-3-(trimethylsilyl)propan-2-yl)quinoline (4ba): Colorless oil, 121.3 mg, 87% yield, PE/EA = 6/1. **^{1}H NMR** (400 MHz, $CDCl_3$) δ 8.04 (d, J = 8.4 Hz, 1H), 7.90 (d, J = 8.3 Hz, 1H), 7.67 – 7.60 (m, 1H), 7.50 – 7.43 (m, 1H), 7.31 – 7.26 (m, 1H), 7.08 (s, 1H), 6.86 (d, J = 7.6 Hz, 1H), 6.73 (d, J = 7.7 Hz, 1H), 3.62 – 3.53 (m, 1H), 3.30 (dd, J = 13.4, 7.8 Hz, 1H), 3.13 (dd, J = 13.4, 7.2 Hz, 1H), 2.60 (s, 3H), 2.50 (s, 3H), 1.35 (dd, J = 14.6, 10.8 Hz, 1H), 0.98 (dd, J = 14.6, 4.2 Hz, 1H), -0.25 (s, 9H). **^{13}C NMR** (100 MHz, $CDCl_3$) δ 165.63, 159.86, 157.64, 147.77, 143.78, 136.22, 129.69, 128.84, 127.11, 125.43, 123.65, 122.02, 120.65, 120.53, 47.92, 45.05, 24.62, 22.85, 18.74, -0.95. **HRMS** (m/z) $[M+H]^+$ calcd. for $C_{22}H_{29}N_2Si$, 349.2100, found 349.2103.

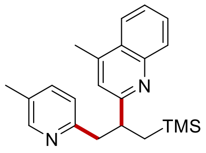

4-Methyl-2-(1-(5-methylpyridin-2-yl)-3-(trimethylsilyl)propan-2-yl)quinoline (4ca): Colorless oil, 124.1 mg, 89% yield, PE/EA = 6/1. **^{1}H NMR** (400 MHz, $CDCl_3$) δ 8.32 (d, J = 2.1 Hz, 1H), 8.04 (d, J = 8.4 Hz, 1H), 7.90 (dd, J = 8.3, 0.9 Hz, 1H), 7.67 – 7.60 (m, 1H), 7.49 – 7.43 (m, 1H), 7.20 (dd, J = 7.9, 1.9 Hz, 1H), 7.07 (s, 1H), 6.84 (d, J = 7.9 Hz, 1H), 3.61 – 3.51 (m, 1H), 3.29 (dd, J = 13.4, 7.8 Hz, 1H), 3.11 (dd, J = 13.4, 7.2 Hz, 1H), 2.60 (d, J = 0.7 Hz, 3H), 2.21 (s, 3H), 1.34 (dd, J = 14.6, 10.5 Hz, 1H), 0.97 (dd, J = 14.6, 4.6 Hz, 1H), -0.24 (s, 9H). **^{13}C NMR** (100 MHz, $CDCl_3$) δ 165.63, 157.59, 149.57, 147.79, 143.88, 136.65, 130.16, 129.70, 128.83, 127.11, 125.42, 123.66, 123.28, 121.97, 47.37, 45.28, 22.87, 18.74, 18.11, -0.93. **HRMS** (m/z) $[M+H]^+$ calcd. for $C_{22}H_{29}N_2Si$, 349.2100, found 349.2102.

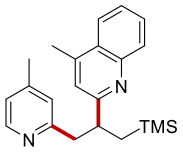

4-Methyl-2-(1-(4-methylpyridin-2-yl)-3-(trimethylsilyl)propan-2-yl)quinoline (4da): Colorless oil, 118.5 mg, 85% yield, PE/EA = 6/1. **^{1}H NMR** (400 MHz, $CDCl_3$) δ 8.35 (d, J = 5.0 Hz, 1H), 8.05 (d, J = 8.4 Hz, 1H), 7.91 (d, J = 7.6 Hz, 1H), 7.69 – 7.61 (m, 1H), 7.52 – 7.44 (m, 1H), 7.09 (s, 1H), 6.90 – 6.78 (m, 2H), 3.65 – 3.54 (m, 1H), 3.27 (dd, J = 13.4, 7.5 Hz, 1H), 3.11 (dd, J = 13.4, 7.5 Hz, 1H), 2.62 (s, 3H), 2.17 (s, 3H), 1.35 (dd, J = 14.6, 10.5 Hz, 1H), 0.97 (dd, J = 14.6, 4.5 Hz, 1H), -0.24 (s, 9H). **^{13}C NMR** (175 MHz, $CDCl_3$) δ 165.79, 160.43, 149.01, 147.87, 147.30, 144.16, 129.79, 129.05, 127.29, 125.62, 125.03, 123.83, 122.31,

122.14, 47.81, 45.21, 22.84, 21.17, 18.93, -0.76. **HRMS** (m/z) $[M+H]^+$ calcd. for $C_{22}H_{29}N_2Si$, 349.2100, found 349.2105.

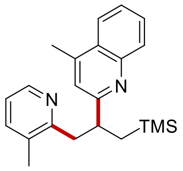

4-Methyl-2-(1-(3-methylpyridin-2-yl)-3-(trimethylsilyl)propan-2-yl)quinoline (4ea): Colorless oil, 125.5 mg, 90% yield, PE/EA = 6/1. **^{1}H NMR** (400 MHz, $CDCl_3$) δ 8.35 (dd, J = 4.6, 1.1 Hz, 1H), 8.03 (d, J = 8.3 Hz, 1H), 7.87 (dd, J = 8.3, 0.9 Hz, 1H), 7.66 – 7.59 (m, 1H), 7.48 – 7.40 (m, 1H), 7.21 (d, J = 7.5 Hz, 1H), 6.98 (s, 1H), 6.90 (dd, J = 7.5, 4.8 Hz, 1H), 3.75 – 3.66 (m, 1H), 3.36 (dd, J = 13.5, 8.0 Hz, 1H), 3.11 (dd, J = 13.5, 6.8 Hz, 1H), 2.55 (d, J = 0.6 Hz, 3H), 2.10 (s, 3H), 1.48 (dd, J = 14.5, 10.7 Hz, 1H), 1.02 (dd, J = 14.5, 4.5 Hz, 1H), -0.23 (s, 9H). **^{13}C NMR** (100 MHz, $CDCl_3$) δ 165.60, 159.11, 147.76, 146.57, 143.53, 137.45, 131.74, 129.65, 128.74, 127.05, 125.31, 123.62, 122.40, 120.95, 44.51, 44.34, 22.75, 19.09, 18.64, -0.88. **HRMS** (m/z) $[M+H]^+$ calcd. for $C_{22}H_{29}N_2Si$, 349.2100, found 349.2101.

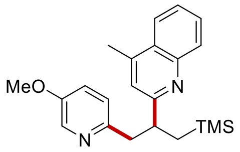

2-(1-(5-Methoxypyridin-2-yl)-3-(trimethylsilyl)propan-2-yl)-4-methylquinoline (4fa): Colorless oil, 106.4 mg, 73% yield, PE/EA = 7/1. **^{1}H NMR** (700 MHz, $CDCl_3$) δ 8.20 (d, J = 2.8 Hz, 1H), 8.04 (d, J = 8.4 Hz, 1H), 7.91 (d, J = 8.2 Hz, 1H), 7.64 (t, J = 7.6 Hz, 1H), 7.47 (t, J = 7.5 Hz, 1H), 7.05 (s, 1H), 6.93 (dd, J = 8.5, 2.9 Hz, 1H), 6.85 (d, J = 8.5 Hz, 1H), 3.77 (s, 3H), 3.56 – 3.50 (m, 1H), 3.27 (dd, J = 13.5, 8.0 Hz, 1H), 3.10 (dd, J = 13.6, 7.1 Hz, 1H), 2.61 (s, 3H), 1.33 (dd, J = 14.7, 10.4 Hz, 1H), 0.98 (dd, J = 14.7, 4.6 Hz, 1H), -0.23 (s, 9H). **^{13}C NMR** (175 MHz, $CDCl_3$) δ 165.68, 153.83, 152.75, 147.81, 143.89, 136.58, 129.70, 128.86, 127.12, 125.44, 123.88, 123.69, 122.02, 120.81, 55.61, 46.85, 45.35, 22.90, 18.79, -0.89. **HRMS** (m/z) $[M+H]^+$ calcd. for $C_{22}H_{29}N_2OSi$, 365.2049, found 365.2053.

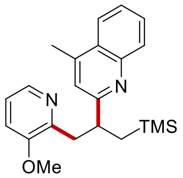

2-(1-(3-Methoxypyridin-2-yl)-3-(trimethylsilyl)propan-2-yl)-4-methylquinoline (4ga): Colorless oil, 116.7 mg, 80% yield, PE/EA = 7/1. **^{1}H NMR** (400 MHz, $CDCl_3$) δ 8.04 (dd, J = 4.6, 1.1 Hz, 1H), 8.00 (d, J = 8.4 Hz, 1H), 7.89 (d, J = 8.2 Hz, 1H), 7.64 – 7.57 (m, 1H), 7.48 – 7.40 (m, 1H), 7.10 (s, 1H), 7.02 – 6.97 (m, 1H), 6.94 (dd, J = 8.2, 1.0 Hz, 1H), 3.70 – 3.61 (m, 1H), 3.57 (s, 3H), 3.33 (dd, J = 13.3, 7.9 Hz, 1H), 3.21 (dd, J = 13.3, 7.0 Hz, 1H), 2.61 (s, 3H), 1.39 (dd, J = 14.6, 10.1 Hz, 1H), 1.01 (dd, J = 14.6, 5.0 Hz, 1H), -0.20 (s, 9H). **^{13}C NMR** (100 MHz, $CDCl_3$) δ 166.05, 154.02, 150.78, 147.69, 143.34, 140.50, 129.75, 128.63, 127.09, 125.26, 123.53, 121.86, 121.75, 116.65, 55.10, 43.98, 41.88, 22.70, 18.76, -0.86. **HRMS** (m/z) $[M+H]^+$ calcd. for $C_{22}H_{29}N_2OSi$, 365.2049, found 365.2051.

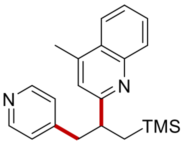

4-Methyl-2-(1-(pyridin-4-yl)-3-(trimethylsilyl)propan-2-yl)quinoline (4ha): Colorless oil, 100.4 mg, 75% yield, PE/EA = 4/1. **^{1}H NMR** (400 MHz, $CDCl_3$) δ 8.36 (d, J = 5.8 Hz, 2H), 8.03 (d, J = 8.4 Hz, 1H), 7.92 (d, J = 7.8 Hz, 1H), 7.69 – 7.63 (m, 1H), 7.52 – 7.46 (m, 1H), 7.00 – 6.95 (m, 3H), 3.36 – 3.27 (m, 1H), 3.20 (dd, J = 13.2, 8.2 Hz, 1H), 2.95 (dd, J = 13.3, 6.6 Hz, 1H), 2.62 (s, 3H), 1.27 (dd, J = 14.6, 9.8 Hz, 1H), 0.99 (dd, J = 14.6, 5.1 Hz, 1H), -0.20 (s, 9H). **^{13}C NMR** (100 MHz, $CDCl_3$) δ 164.65, 149.79, 149.57, 147.80, 144.26, 129.70, 129.13, 127.16, 125.72, 124.72, 123.74, 121.59, 45.95, 44.59, 23.26, 18.82, -0.95. **HRMS** (m/z) $[M+H]^+$ calcd. for $C_{21}H_{27}N_2Si$, 335.1944, found 335.1947.

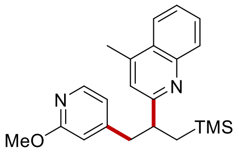

2-(1-(2-Methoxypyridin-4-yl)-3-(trimethylsilyl)propan-2-yl)-4-methylquinoline (4ia): Colorless oil, 117.7 mg, 81% yield, PE/EA = 6/1. **^{1}H NMR** (400 MHz, $CDCl_3$) δ 8.05 (d, *J* = 8.3 Hz, 1H), 7.98 – 7.88 (m, 2H), 7.71 – 7.62 (m, 1H), 7.54 – 7.45 (m, 1H), 7.01 (s, 1H), 6.59 (dd, *J* = 5.3, 1.1 Hz, 1H), 6.50 (s, 1H), 3.85 (s, 3H), 3.41 – 3.23 (m, 1H), 3.16 (dd, *J* = 13.3, 7.9 Hz, 1H), 2.89 (dd, *J* = 13.4, 6.9 Hz, 1H), 2.63 (d, *J* = 0.5 Hz, 3H), 1.24 (dd, *J* = 14.6, 9.9 Hz, 1H), 0.99 (dd, *J* = 14.6, 5.1 Hz, 1H), -0.20 (s, 9H). **^{13}C NMR** (100 MHz, $CDCl_3$) δ 164.88, 164.44, 152.62, 146.44, 129.71, 129.12, 127.18, 125.69, 123.73, 121.58, 118.25, 111.19, 53.34, 45.71, 44.43, 23.13, 18.84, -0.94. **HRMS** (m/z) $[M+H]^+$ calcd. for $C_{22}H_{29}N_2OSi$, 365.2049, found 365.2052.

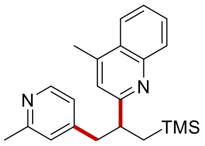

4-Methyl-2-(1-(2-methylpyridin-4-yl)-3-(trimethylsilyl)propan-2-yl)quinoline (4ja): Colorless oil, 99.0 mg, 71% yield, PE/EA = 6/1. **^{1}H NMR** (400 MHz, $CDCl_3$) δ 8.25 (d, *J* = 5.1 Hz, 1H), 8.04 (d, *J* = 8.0 Hz, 1H), 7.93 (dd, *J* = 8.3, 0.9 Hz, 1H), 7.70 – 7.63 (m, 1H), 7.54 – 7.46 (m, 1H), 6.99 (s, 1H), 6.88 (s, 1H), 6.78 (d, *J* = 5.1 Hz, 1H), 3.36 – 3.27 (m, 1H), 3.15 (dd, *J* = 13.3, 7.9 Hz, 1H), 2.90 (dd, *J* = 13.3, 6.9 Hz, 1H), 2.63 (d, *J* = 0.7 Hz, 3H), 2.43 (s, 3H), 1.25 (dd, *J* = 14.6, 10.0 Hz, 1H), 0.98 (dd, *J* = 14.6, 5.0 Hz, 1H), -0.20 (s, 9H). **^{13}C NMR** (100 MHz, $CDCl_3$) δ 164.86, 158.08, 150.01, 148.85, 147.80, 144.21, 129.71, 129.12, 127.17, 125.70, 124.30, 123.73, 121.82, 121.54, 45.91, 44.63, 24.41, 23.09, 18.84, -0.93. **HRMS** (m/z) $[M+H]^+$ calcd. for $C_{22}H_{29}N_2Si$, 349.2100, found 349.2104.

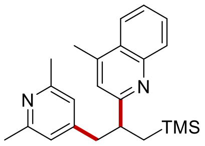

2-(1-(2,6-Dimethylpyridin-4-yl)-3-(trimethylsilyl)propan-2-yl)-4-methylquinoline (4ka): Colorless oil, 105.9 mg, 73% yield, PE/EA = 6/1. **^{1}H NMR** (400 MHz, $CDCl_3$) δ 8.04 (d, *J* = 8.3 Hz, 1H), 7.93 (dd, *J* = 8.3, 0.8 Hz, 1H), 7.70 – 7.64 (m, 1H), 7.53 – 7.48 (m, 1H), 7.00 (s, 1H), 6.70 (s, 2H), 3.35 – 3.26 (m, 1H), 3.10 (dd, *J* = 13.3, 7.7 Hz, 1H), 2.86 (dd, *J* = 13.3, 7.1 Hz, 1H), 2.64 (d, *J* = 0.6 Hz, 3H), 2.42 (s, 6H), 1.24 (dd, *J* = 14.6, 10.1 Hz, 2H), 0.96 (dd, *J* = 14.6, 4.9 Hz, 1H), -0.21 (s, 9H). **^{13}C NMR** (100 MHz, $CDCl_3$) δ 164.93, 157.10, 150.83, 147.78, 144.24, 129.70, 129.15, 127.18, 125.72, 123.73, 121.59, 121.49, 45.82, 44.65, 24.13, 22.98, 18.86, -0.92. **HRMS** (m/z) $[M+H]^+$ calcd. for $C_{23}H_{31}N_2Si$, 363.2257, found 363.2253.

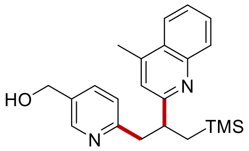

(6-(2-(4-Methylquinolin-2-yl)-3-(trimethylsilyl)propyl)pyridin-3-yl)methanol (4la): Colorless oil, 126.9 mg, 87% yield, PE/EA = 1/1. **^{1}H NMR** (400 MHz, $CDCl_3$) δ 8.42 – 8.32 (m, 1H), 8.01 (d, *J* = 8.4 Hz, 1H), 7.90 (d, *J* = 8.2 Hz, 1H), 7.62 (t, *J* = 7.6 Hz, 1H), 7.51 – 7.40 (m, 2H), 7.09 (s, 1H), 6.93 (d, *J* = 7.9 Hz, 1H), 4.60 (s, 2H), 3.67 – 3.46 (m, 2H), 3.31 (dd, *J* = 13.4, 8.0 Hz, 1H), 3.14 (dd, *J* = 13.4, 7.0 Hz, 1H), 2.61 (s, 3H), 1.31 (dd, *J* = 14.6, 10.4 Hz, 1H), 0.97 (dd, *J* = 14.6, 4.7 Hz, 1H), -0.24 (s, 9H). **^{13}C NMR** (100 MHz, $CDCl_3$) δ 165.44, 159.68, 147.97, 147.56, 144.34, 135.33, 133.92, 129.43, 129.05, 127.14, 125.60, 123.7, 123.66, 121.79, 62.35, 47.33, 45.17, 23.17, 18.82, -0.94. **HRMS** (m/z) $[M+H]^+$ calcd. for $C_{22}H_{29}N_2OSi$, 365.2049, found 365.2052.

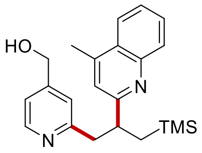

(2-(2-(4-Methylquinolin-2-yl)-3-(trimethylsilyl)propyl)pyridin-4-yl)methanol (4ma): Light yellow oil, 126.9 mg, 87% yield, PE/EA = 1/1. **^{1}H NMR** (400 MHz, $CDCl_3$) δ 8.34 (d, J = 5.1 Hz, 1H), 7.98 (d, J = 8.3 Hz, 1H), 7.89 (d, J = 8.0 Hz, 1H), 7.63 – 7.57 (m, 1H), 7.50 – 7.41 (m, 1H), 7.13 (s, 1H), 7.08 – 6.99 (m, 2H), 4.58 (s,

2H), 4.34 (br, 1H), 3.64 – 3.53 (m, 1H), 3.25 (dd, J = 13.4, 7.7 Hz, 1H), 3.13 (dd, J = 13.5, 7.4 Hz, 1H), 2.61 (s, 3H), 1.30 (dd, J = 14.6, 10.5 Hz, 1H), 0.96 (dd, J = 14.6, 4.6 Hz, 1H), -0.26 (s, 9H). **^{13}C NMR** (100 MHz, $CDCl_3$) δ 165.60, 160.44, 150.77, 149.01, 147.48, 144.41, 129.23, 129.05, 127.13, 125.59, 123.72, 121.73, 121.30, 118.81, 63.08, 47.70, 45.06, 22.84, 18.83, -0.95. **HRMS** (m/z) $[M+H]^+$ calcd. for $C_{22}H_{29}N_2OSi$, 365.2049, found 365.2051.

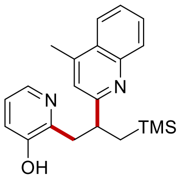

2-(2-(4-Methylquinolin-2-yl)-3-(trimethylsilyl)propyl)pyridin-3-ol (4na): Colorless oil, 88.3 mg, 63% yield, PE/EA = 2/1. **^{1}H NMR** (400 MHz, $CDCl_3$) δ 11.56 (br, 1H), 8.13 (d, *J* = 8.4 Hz, 1H), 8.02 (d, *J* = 4.0 Hz, 1H), 7.91 (dd, *J* = 8.3, 1.3 Hz, 1H), 7.75 – 7.68 (m, 1H), 7.56 – 7.48 (m, 1H), 7.16 (s, 1H), 6.99 (d, *J* = 7.9 Hz, 1H), 6.89 (dd, *J* = 8.0, 4.6 Hz, 1H), 3.89 – 3.71 (m, 2H), 3.11 – 3.00 (m, 1H), 2.63 (s, 3H), 1.17 (d, *J* = 6.5 Hz, 2H), 0.00 (s, 9H). **^{13}C NMR** (100 MHz, $CDCl_3$) δ 165.86, 151.72, 149.70, 145.89, 145.74, 140.76, 129.95, 127.98, 127.24, 126.22, 124.77, 123.90, 122.98, 122.57, 44.99, 37.27, 27.20, 18.90, -0.35. **HRMS** (m/z) $[M+H]^+$ calcd. for $C_{21}H_{27}N_2OSi$, 351.1893, found 351.1896.

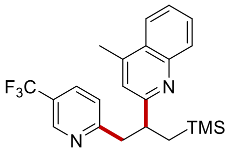

4-Methyl-2-(1-(5-(trifluoromethyl)pyridin-2-yl)-3-(trimethylsilyl)propan-2-yl)quinoline (4oa): Light brown solid, m.p. 108-110 ˚C, 117.5 mg, 73% yield, PE/EA = 5/1. **^{1}H NMR** (400 MHz, $CDCl_3$) δ 8.76 (s, 1H), 8.04 (d, *J* = 8.4 Hz, 1H), 7.90 (d, *J* = 8.3 Hz, 1H), 7.69 – 7.57 (m, 2H), 7.52 – 7.44 (m, 1H), 7.04 (d, *J* = 7.9 Hz, 2H), 3.68 – 3.57 (m, 1H), 3.45 (dd, *J* = 13.3, 8.4 Hz, 1H), 3.24 (dd, *J* = 13.3, 6.5 Hz, 1H), 2.60 (s, 3H), 1.37 (dd, *J* = 14.6, 10.1 Hz, 1H), 1.01 (dd, *J* = 14.6, 5.0 Hz, 1H), -0.20 (s, 9H). **^{13}C NMR** (100 MHz, $CDCl_3$) δ 164.86 (d, J_{CF} = 1.2 Hz), 164.81, 147.73, 146.15 (q, J_{CF} = 4.0 Hz), 144.28, 133.01 (q, J_{CF} = 3.4 Hz), 129.61, 129.06, 127.11, 125.65, 124.03 (q, J_{CF} = 32.8Hz), 123.80 (q, J_{CF} = 272.0 Hz, CF_3), 123.72, 123.54, 121.86, 47.44, 44.90, 23.42, 18.74, -0.95. **HRMS** (m/z) $[M+H]^+$ calcd. for $C_{22}H_{26}F_3N_2Si$, 403.1817, found 403.1822.

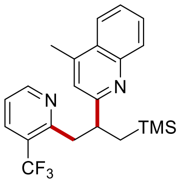

4-Methyl-2-(1-(3-(trifluoromethyl)pyridin-2-yl)-3-(trimethylsilyl)propan-2-yl)quinoline (4pa): Light brown oil, 135.3 mg, 84% yield, PE/EA = 5/1. **^{1}H NMR** (400 MHz, $CDCl_3$) δ 8.62 (d, J = 4.2 Hz, 1H), 8.03 (d, J = 8.2 Hz, 1H), 7.89 (d, J = 8.3 Hz, 1H), 7.79 (d, J = 7.9 Hz, 1H), 7.65 – 7.58 (m, 1H), 7.48 – 7.41 (m, 1H), 7.15 (s, 1H), 7.10 (dd, J = 7.8, 4.9 Hz, 1H), 4.02 – 3.93 (m, 1H), 3.58 (dd, J = 14.8, 7.7 Hz, 1H), 3.37 (dd, J = 14.8, 6.8 Hz, 1H), 2.62 (s, 3H), 1.43 (dd, J = 14.5, 10.2 Hz, 1H), 0.99 (dd, J = 14.6, 4.9 Hz, 1H), -0.18 (s, 9H). **^{13}C NMR** (100 MHz, $CDCl_3$) δ 165.36, 159.23 (d, J_{CF} = 1.3 Hz), 151.71 (d, J_{CF} = 0.7 Hz), 147.84, 143.64, 133.70 (q, J_{CF} = 5.4 Hz), 129.86, 128.70, 127.05, 125.35, 125.11 (q, J_{CF} = 31.2 Hz), 124.09 (q, J_{CF} = 273.4 Hz), 123.53, 121.95, 120.39, 43.79 (d, J_{CF} = 1.4 Hz), 43.41, 22.97, 18.71, -0.93. **HRMS** (m/z) $[M+H]^+$ calcd. for $C_{22}H_{26}F_3N_2Si$, 403.1817, found 403.1815.

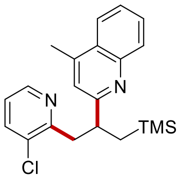

2-(1-(3-Chloropyridin-2-yl)-3-(trimethylsilyl)propan-2-yl)-4-methylquinoline (4qa): Colorless oil, 119.5 mg, 81% yield, PE/EA = 6/1. **^{1}H NMR** (400 MHz, $CDCl_3$) δ 8.34 (dd, *J* = 4.6, 1.4 Hz, 1H), 8.02 (d, *J* = 8.3 Hz, 1H), 7.93 – 7.87 (m, 1H), 7.67 – 7.58 (m, 1H), 7.52 (dd, *J* = 8.0, 1.4 Hz, 1H), 7.50 – 7.40 (m, 1H), 7.12 (s, 1H), 6.98 (dd, *J* = 8.0, 4.7 Hz, 1H), 3.81 – 3.71 (m, 1H), 3.49 (dd, *J* = 13.8, 7.7 Hz, 1H), 3.33 (dd, *J* = 13.8, 7.1 Hz, 1H), 2.62 (s, 3H), 1.44 (dd, *J* = 14.6, 10.2 Hz, 1H), 1.01 (dd, *J* = 14.6, 4.9 Hz, 1H), -0.20 (s, 9H). **^{13}C NMR** (100 MHz, $CDCl_3$) δ 165.30, 157.97, 147.79, 147.10, 143.74, 136.72, 131.79,

129.85, 128.78, 127.13, 125.42, 123.60, 122.09, 121.85, 44.20, 43.73, 22.74, 18.79, -0.87. **HRMS** (m/z) $[M+H]^+$ calcd. for $C_{21}H_{26}ClN_2Si$, 369.1554, found 369.1557.

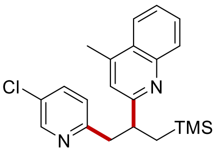

2-(1-(5-Chloropyridin-2-yl)-3-(trimethylsilyl)propan-2-yl)-4-methylquinolinel (4ra): Colorless oil, 70.8 mg, 48% yield, PE/EA = 6/1. **^{1}H NMR** (400 MHz, $CDCl_3$) δ 8.45 (d, J = 2.4 Hz, 1H), 8.03 (d, J = 8.4 Hz, 1H), 7.94 – 7.87 (m, 2H), 7.69 – 7.60 (m, 1H), 7.52 – 7.43 (m, 1H), 7.35 (dd, J = 8.3, 2.5 Hz, 1H), 7.03 (s, 1H), 6.87 (d, J = 8.3 Hz, 1H), 3.61 – 3.49 (m, 1H), 3.33 (dd, J = 13.4, 8.3 Hz, 1H), 3.14 (dd, J = 13.4, 6.7 Hz, 1H), 2.61 (d, J = 1.0 Hz, 3H), 1.34 (dd, J = 14.5, 10.2 Hz, 1H), 0.98 (dd, J = 14.6, 4.9 Hz, 1H), -0.21 (s, 9H). **^{13}C NMR** (100 MHz, Chloroform-*d*) δ 165.09, 158.94, 148.04, 147.81, 144.05, 135.67, 129.69, 129.33, 128.95, 127.10, 125.54, 124.59, 123.70, 121.91, 46.92, 45.08, 23.24, 18.79, -0.91. **HRMS** (m/z) $[M+H]^+$ calcd. for $C_{21}H_{26}ClN_2Si$, 369.1554, found 369.1558.

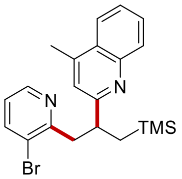

2-(1-(3-Bromopyridin-2-yl)-3-(trimethylsilyl)propan-2-yl)-4-methylquinoline (4sa): Colorless oil, 125.7 mg, 76% yield, PE/EA = 6/1. **^{1}H NMR** (400 MHz, $CDCl_3$) δ 8.38 (dd, *J* = 4.6, 1.3 Hz, 1H), 8.03 (d, *J* = 8.3 Hz, 1H), 7.90 (d, *J* = 7.8 Hz, 1H), 7.71 (dd, *J* = 8.0, 1.4 Hz, 1H), 7.67 – 7.59 (m, 1H), 7.51 – 7.42 (m, 1H), 7.12 (s, 1H), 6.90 (dd, *J* = 8.0, 4.6 Hz, 1H), 3.84 – 3.70 (m, 1H), 3.52 (dd, *J* = 13.9, 7.8 Hz, 1H), 3.34 (dd, *J* = 13.9, 7.0 Hz, 1H), 2.62 (s, 3H), 1.45 (dd, *J* = 14.6, 10.2 Hz, 1H), 1.02 (dd, *J* = 14.6, 4.8 Hz, 1H), -0.19 (s, 9H). **^{13}C NMR** (100 MHz, $CDCl_3$) δ 165.26, 159.14, 147.67, 140.08, 129.87, 128.78, 127.13, 125.42, 123.60, 122.32, 122.16, 121.94, 46.15, 43.82, 22.73, 18.80, -0.85. **HRMS** (m/z) $[M+H]^+$ calcd. for $C_{21}H_{26}BrN_2Si$, 413.1049, found 413.1053.

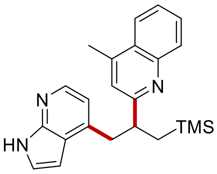

2-(1-(1H-pyrrolo[2,3-b]pyridin-4-yl)-3-(trimethylsilyl)propan-2-yl)-4-methylquinoline (4ta): Light yellow oil, 82.2 mg, 55% yield, PE/EA = 1/1. **^{1}H NMR** (700 MHz, Chloroform-*d*) δ 11.23 (s, 1H), 8.12 – 8.06 (m, 2H), 7.91 (d, *J* = 8.1 Hz, 1H), 7.69 – 7.65 (m, 1H), 7.51 – 7.47 (m, 1H), 7.27 (d, *J* = 3.5 Hz, 1H), 6.97 (s, 1H), 6.80 (d, *J* = 5.0 Hz, 1H), 6.53 (d, *J* = 3.5 Hz, 1H), 3.58 – 3.53 (m, 1H), 3.49 (dd, *J* = 13.4, 7.9 Hz, 1H), 3.29 (dd, *J* = 13.4, 6.9 Hz, 1H), 2.58 (s, 3H), 1.38 (dd, *J* = 14.7, 10.0 Hz, 1H), 1.06 (dd, *J* = 14.8, 4.8 Hz, 1H), -0.20 (s, 9H). **^{13}C NMR** (175 MHz, $CDCl_3$) δ 165.37, 148.50, 147.92, 144.34, 143.71, 142.18, 129.78, 129.19, 127.31, 125.73, 124.80, 123.85, 121.89, 121.31, 116.67, 99.67, 45.46, 42.71, 23.44, 18.91, -0.78. **HRMS** (m/z) $[M+H]^+$ calcd. for $C_{23}H_{28}N_3Si$, 374.2052, found 374.2055.

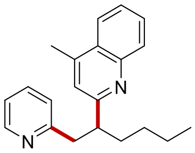

4-Methyl-2-(1-(pyridin-2-yl)hexan-2-yl)quinoline (7a): Colorless oil, 90.1 mg, 74% yield, PE/EA = 6/1. **^{1}H NMR** (700 MHz, $CDCl_3$) δ 8.49 (d, *J* = 4.5 Hz, 1H), 8.06 (d, *J* = 8.4 Hz, 1H), 7.91 (d, *J* = 8.2 Hz, 1H), 7.65 (t, *J* = 7.6 Hz, 1H), 7.48 (t, *J* = 7.5 Hz, 1H), 7.40 (td, *J* = 7.7, 1.1 Hz, 1H), 7.04 (s, 1H), 7.02 – 6.96 (m, 2H), 3.48 – 3.41 (m, 1H), 3.35 (dd, *J* = 13.5, 8.2 Hz, 1H), 3.20 (dd, *J* = 13.5, 6.8 Hz, 1H), 2.61 (s, 3H), 1.96 – 1.88 (m, 1H), 1.77 – 1.70 (m, 1H), 1.26 – 1.20 (m, 3H), 1.17 – 1.09 (m, 1H), 0.78 (t, *J* = 7.0 Hz, 3H). **^{13}C NMR** (175 MHz, $CDCl_3$) δ 164.67, 160.86, 149.19, 147.92, 143.91, 136.09, 129.75, 128.88, 127.14, 125.48, 123.88, 123.72, 122.21, 121.04, 48.83, 44.07, 35.01, 29.88, 22.89, 18.83, 14.07. **HRMS** (m/z) $[M+H]^+$ calcd. for $C_{21}H_{25}N_2$, 305.2018, found 305.2020.

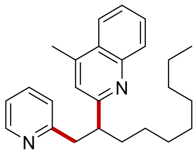

4-Methyl-2-(1-(pyridin-2-yl)decan-2-yl)quinoline (7b): Colorless oil, 103.8 mg, 72% yield, PE/EA = 6/1. **^{1}H NMR** (700 MHz, $CDCl_3$) δ 8.49 (d, *J* = 4.5 Hz, 1H), 8.06 (d, *J* = 8.4 Hz, 1H), 7.91 (d, *J* = 8.3 Hz, 1H), 7.65 (t, *J* = 7.6 Hz, 1H), 7.47 (t, *J* = 7.5 Hz, 1H), 7.41 – 7.37 (m, 1H), 7.04 (s, 1H), 7.01 – 6.97 (m, 2H), 3.48 – 3.43 (m, 1H), 3.35 (dd, *J* = 13.5, 8.2 Hz, 1H), 3.20 (dd, *J* = 13.6, 6.8 Hz, 1H), 2.61 (s, 3H), 1.95 – 1.89 (m, 1H), 1.75 – 1.70 (m, 1H), 1.26 – 1.19 (m, 5H), 1.18 – 1.13 (m, 7H), 0.82 (t, *J* = 7.2 Hz, 3H). **^{13}C NMR** (100 MHz, C_6D_6) δ 164.67, 160.86, 149.21, 147.86, 143.92, 136.04, 129.72, 128.88, 127.12, 125.47, 123.86, 123.70, 122.21, 121.00, 48.82, 44.09, 35.28, 31.93, 29.80, 29.49, 29.31, 27.64, 22.73, 18.82, 14.20. **HRMS** (m/z) $[M+H]^+$ calcd. for $C_{25}H_{33}N_2$, 361.2644, found 361.2649.

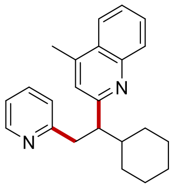

2-(1-Cyclohexyl-2-(pyridin-2-yl)ethyl)-4-methylquinoline (7c): Colorless oil, 88.6 mg, 67% yield, PE/EA = 6/1. **^{1}H NMR** (400 MHz, $CDCl_3$) δ 8.41 (d, *J* = 4.5 Hz, 1H), 8.06 (d, *J* = 8.4 Hz, 1H), 7.88 (d, *J* = 8.3 Hz, 1H), 7.64 (t, *J* = 7.6 Hz, 1H), 7.46 (t, *J* = 7.6 Hz, 1H), 7.28 – 7.23 (m, 1H), 6.94 (s, 1H), 6.92 – 6.84 (m, 2H), 3.47 – 3.35 (m, 2H), 3.35 – 3.26 (m, 1H), 2.56 (s, 3H), 2.05 (d, *J* = 12.3 Hz, 1H), 1.92 – 1.82 (m, 1H), 1.76 (d, *J* = 12.7 Hz, 1H), 1.60 (d, *J* = 9.5 Hz, 2H), 1.46 (d, *J* = 12.4 Hz, 1H), 1.31 – 1.23 (m, 1H), 1.19 – 1.08 (m, 3H), 1.06 – 0.98 (m, 1H). **^{13}C NMR** (100 MHz, $CDCl_3$) δ 163.77, 161.37, 148.96, 147.55, 143.35, 135.90, 129.63, 128.81, 126.96, 125.41, 123.94, 123.67, 123.38, 120.74, 54.44, 42.96, 40.17, 31.43, 31.11, 26.67, 26.61, 26.57, 18.78. **HRMS** (m/z) $[M+H]^+$ calcd. for $C_{23}H_{27}N_2$, 331.2174, found 331.2177.

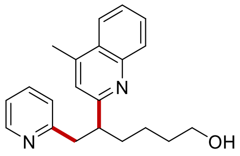

5-(4-Methylquinolin-2-yl)-6-(pyridin-2-yl)hexan-1-ol (7d): Colorless oil, 96.1 mg, 75% yield, PE/EA = 3/1. **^{1}H NMR** (400 MHz, $CDCl_3$) δ 8.49 (d, *J* = 4.2 Hz, 1H), 8.05 (d, *J* = 8.3 Hz, 1H), 7.91 (dd, *J* = 8.3, 0.9 Hz, 1H), 7.70 – 7.60 (m, 1H), 7.52 – 7.46 (m, 1H), 7.42 (td, *J* = 7.7, 1.8 Hz, 1H), 7.07 – 6.99 (m, 2H), 6.97 (d, *J* = 7.8 Hz, 1H), 3.56 (t, *J* = 6.4 Hz, 2H), 3.52 – 3.43 (m, 1H), 3.32 (dd, *J* = 13.5, 7.6 Hz, 1H), 3.17 (dd, *J* = 13.5, 7.2 Hz, 1H), 2.61 (s, 3H), 2.40 (s, 1H), 2.05 – 1.93 (m, 1H), 1.79 – 1.69 (m, 1H), 1.56 – 1.46 (m, 2H), 1.37 – 1.26 (m, 2H). **^{13}C NMR** (100 MHz, $CDCl_3$) δ 164.34, 160.57, 149.19, 147.80, 144.27, 136.25, 129.58, 129.07, 127.17, 125.65, 123.97, 123.74, 122.18, 121.20, 62.51, 48.55, 44.02, 34.06, 32.57, 23.57, 18.84. **HRMS** (m/z) $[M+H]^+$ calcd. for $C_{21}H_{25}N_2O$, 321.1967, found 321.1964.

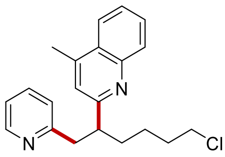

2-(6-Chloro-1-(pyridin-2-yl)hexan-2-yl)-4-methylquinoline (7e): Colorless oil, 71.8 mg, 53% yield. PE/EA = 5/1. **^{1}H NMR** (400 MHz, $CDCl_3$) δ 8.50 (d, *J* = 4.5 Hz, 1H), 8.06 (d, *J* = 8.4 Hz, 1H), 7.92 (d, *J* = 8.3 Hz, 1H), 7.66 (t, *J* = 7.6 Hz, 1H), 7.48 (t, *J* = 7.5 Hz, 1H), 7.40 (t, *J* = 7.6 Hz, 1H), 7.07 – 6.93 (m, 3H), 3.50 – 3.37 (m, 3H), 3.37 – 3.29 (m, 1H), 3.18 (dd, *J* = 13.4, 7.0 Hz, 1H), 2.61 (s, 3H), 2.04 – 1.92 (m, 1H), 1.79 – 1.65 (m, 3H), 1.44 – 1.32 (m, 1H), 1.32 – 1.23 (m, 1H) .**^{13}C NMR** (100 MHz, $CDCl_3$) δ 164.06, 160.51, 149.24, 147.84, 144.15, 136.14, 129.67, 128.98, 127.12, 125.59, 123.87, 123.72, 122.19, 121.12, 48.49, 44.94, 44.08, 34.16, 32.71, 24.95, 18.82. **HRMS** (m/z) $[M+H]^+$ calcd. for $C_{21}H_{24}ClN_2$, 339.1628, found 339.1633.

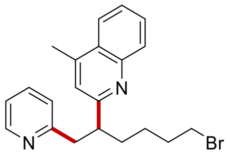

2-(6-Bromo-1-(pyridin-2-yl)hexan-2-yl)-4-methylquinoline (7f): Colorless oil, 95.1 mg, 62% yield, PE/EA = 5/1. **^{1}H NMR** (400 MHz, $CDCl_3$) δ 8.50 (d, *J* = 4.2 Hz, 1H), 8.07 (d, *J* = 8.4 Hz, 1H), 7.93 (d, *J* = 8.0 Hz, 1H), 7.72 – 7.62 (m, 1H), 7.53 – 7.46 (m, 1H), 7.42 (td, *J* = 7.7, 1.7 Hz, 1H), 7.07 – 6.93 (m, 3H), 3.53 – 3.44 (m, 1H), 3.38 – 3.23 (m, 3H), 3.19 (dd, *J* = 13.5, 7.0 Hz, 1H), 2.62 (s, 3H), 2.04 – 1.93 (m, 1H), 1.85 – 1.70 (m, 3H), 1.45 – 1.34 (m, 1H), 1.33 – 1.26 (m, 1H). **^{13}C NMR** (175 MHz, $CDCl_3$) δ 164.17, 160.62, 149.39, 147.89, 144.50, 136.33, 129.75, 129.22, 127.29, 125.81, 124.05, 123.89, 122.35, 121.31, 48.54, 44.21, 34.16, 33.87, 33.05, 26.38, 18.99. **HRMS** (m/z) $[M+H]^+$ calcd. for $C_{21}H_{24}BrN_2$, 383.1123, found 383.1127.

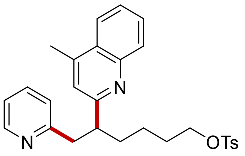

5-(4-Methylquinolin-2-yl)-6-(pyridin-2-yl)hexyl 4-methylbenzenesulfonate (7g): Light brown oil, 113.9 mg, 60% yield, PE/EA = 2/1. **^{1}H NMR** (400 MHz, $CDCl_3$) δ 8.48 (d, *J* = 4.0 Hz, 1H), 8.04 (d, *J* = 8.4 Hz, 1H), 7.91 (d, *J* = 8.3 Hz, 1H), 7.72 – 7.60 (m, 3H), 7.52 – 7.45 (m, 1H), 7.43 – 7.35 (m, 1H), 7.27 – 7.20 (m, 2H), 7.06 – 6.91 (m, 3H), 3.89 (t, *J* = 6.5 Hz, 2H), 3.47 – 3.37 (m, 1H), 3.30 (dd, *J* = 13.1, 7.9 Hz, 1H), 3.13 (dd, *J* = 13.3, 6.9 Hz, 1H), 2.60 (s, 3H), 2.37 (s, 3H), 1.97 – 1.85 (m, 1H), 1.72 – 1.62 (m, 1H), 1.61 – 1.49 (m, 2H), 1.30 – 1.20 (m, 1H), 1.20 – 1.09 (m, 1H). **^{13}C NMR** (100 MHz, $CDCl_3$) δ 163.89, 160.40, 149.21, 147.75, 144.62, 144.28, 136.14, 133.17, 129.81, 129.60, 129.01, 127.86, 27.12, 125.63, 123.83, 123.73, 122.12, 121.12, 70.50, 48.33, 43.99, 34.20, 28.88, 23.44, 21.65, 18.79. **HRMS** (m/z) $[M+H]^+$ calcd. for $C_{28}H_{31}N_2O_3S$, 475.2055, found 475.2056.

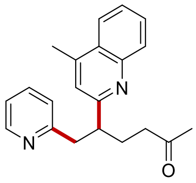

5-(4-Methylquinolin-2-yl)-6-(pyridin-2-yl)hexan-2-one (7h): Colorless oil, 75.1 mg, 59% yield, PE/EA = 3/1. **^{1}H NMR** (700 MHz, $CDCl_3$) δ 8.50 (d, *J* = 4.5 Hz, 1H), 8.06 (d, *J* = 8.2 Hz, 1H), 7.94 (d, *J* = 8.2 Hz, 1H), 7.67 (t, *J* = 7.3 Hz, 1H), 7.51 (t, *J* = 7.4 Hz, 1H), 7.44 (td, *J* = 7.6, 1.4 Hz, 1H), 7.07 – 6.99 (m, 3H), 3.52 – 3.45 (m, 1H), 3.36 (dd, *J* = 13.7, 7.7 Hz, 1H), 3.19 (dd, *J* = 13.7, 7.3 Hz, 1H), 2.63 (s, 3H), 2.40 – 2.35 (m, 1H), 2.32 – 2.27 (m, 1H), 2.24 – 2.17 (m, 1H), 2.07 – 2.02 (m, 1H), 2.00 (s, 3H). **^{13}C NMR** (175 MHz, $CDCl_3$) δ 209.02, 163.62, 160.34, 149.44, 147.89, 144.69, 136.43, 129.86, 129.28, 127.35, 125.94, 124.08, 123.92, 122.20, 121.41, 47.87, 44.10, 41.91, 30.11, 28.77, 19.01. **HRMS** (m/z) $[M+H]^+$ calcd. for $C_{21}H_{23}N_2O$, 319.1810, found 319.1812.

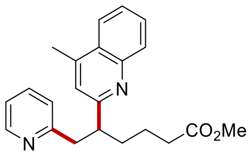

Methyl-5-(4-methylquinolin-2-yl)-6-(pyridin-2-yl)hexanoate (7i): Colorless oil, 85.2 mg, 61% yield, PE/EA = 3/1. **^{1}H NMR** (400 MHz, $CDCl_3$) δ 8.49 (d, J = 4.1 Hz, 1H), 8.04 (d, J = 8.4 Hz, 1H), 7.91 (dd, J = 8.3, 1.3 Hz, 1H), 7.68 – 7.62 (m, 1H), 7.51 – 7.45 (m, 1H), 7.40 (td, J = 7.6, 1.8 Hz, 1H), 7.03 – 6.94 (m, 3H), 3.56 (s, 3H), 3.51 – 3.42 (m, 1H), 3.34 (dd, J = 13.4, 7.9 Hz, 1H), 3.18 (dd, J = 13.4, 7.0 Hz, 1H), 2.60 (s, 3H), 2.28 – 2.20 (m, 2H), 2.05 – 1.94 (m, 1H), 1.80 – 1.70 (m, 1H), 1.60 – 1.45 (m, 2H). **^{13}C NMR** (100 MHz, $CDCl_3$) δ 174.02, 163.90, 160.53, 149.28, 147.97, 144.05, 136.08, 129.78, 128.92, 127.16, 125.56, 123.86, 123.71, 122.24, 121.10, 51.48, 48.44, 44.11, 34.41, 34.23, 23.11, 18.80. **HRMS** (m/z) $[M+H]^+$ calcd. for $C_{22}H_{25}N_2O_2$, 349.1916, found 349.1914.

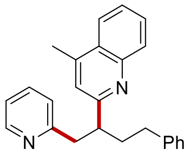

4-Methyl-2-(4-phenyl-1-(pyridin-2-yl)butan-2-yl)quinoline (7j): Colorless oil, 90.3 mg, 64% yield, PE/EA = 5/1. **^{1}H NMR** (700 MHz, $CDCl_3$) δ 8.50 (d, *J* = 4.7 Hz, 1H), 8.13 (d, *J* = 8.2 Hz, 1H), 7.94 (d, *J* = 8.2 Hz, 1H), 7.69 (t, *J* = 7.6 Hz, 1H),

7.52 (t, *J* = 7.5 Hz, 1H), 7.41 (td, *J* = 7.6, 1.5 Hz, 1H), 7.20 (t, *J* = 7.6 Hz, 2H), 7.12 (t, *J* = 7.3 Hz, 1H), 7.09 – 7.05 (m, 3H), 7.04 – 7.01 (m, 1H), 6.99 (d, *J* = 7.7 Hz, 1H), 3.61 – 3.53 (m, 1H), 3.40 (dd, *J* = 13.6, 8.0 Hz, 1H), 3.26 (dd, *J* = 13.6, 7.0 Hz, 1H), 2.63 (s, 3H), 2.57 – 2.47 (m, 2H), 2.35 – 2.29 (m, 1H), 2.11 – 2.05 (m, 1H). **^{13}C NMR** (175 MHz, $CDCl_3$) δ 164.14, 160.59, 149.37, 147.82, 144.54, 142.59, 136.35, 129.75, 129.27, 128.60, 128.45, 127.32, 125.87, 124.08, 123.90, 122.53, 121.31, 48.49, 44.25, 36.83, 34.08, 19.00. **HRMS** (m/z) $[M+H]^+$ calcd. for $C_{25}H_{25}N_2$, 353.2018, found 353.2023.

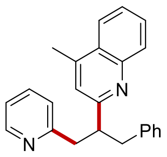

4-Methyl-2-(1-phenyl-3-(pyridin-2-yl)propan-2-yl)quinoline (7k): Colorless oil, 82.6 mg, 61% yield, PE/EA = 5/1. **^{1}H NMR** (400 MHz, $CDCl_3$) δ 8.49 (d, J = 4.2 Hz, 1H), 8.10 (d, J = 8.4 Hz, 1H), 7.88 (d, J = 8.0 Hz, 1H), 7.70 – 7.64 (m, 1H), 7.50 – 7.45 (m, 1H), 7.39 – 7.34 (m, 1H), 7.17 – 7.12 (m, 2H), 7.11 – 7.06 (m, 3H), 7.00 – 6.94 (m, 2H), 6.80 (s, 1H), 3.90 - 3.76 (m, 1H), 3.45 (dd, J = 13.6, 8.7 Hz, 1H), 3.35 – 3.22 (m, 2H), 3.07 (dd, J = 13.5, 6.3 Hz, 1H), 2.49 (s, 3H). **^{13}C NMR** (100 MHz, $CDCl_3$) δ 163.50, 160.60, 149.23, 147.95, 143.60, 140.45, 136.01, 129.75, 129.36, 128.81, 128.16, 127.11, 125.90, 125.48, 124.04, 123.71, 122.95, 121.03, 50.24, 43.36, 41.50, 18.61. **HRMS** (m/z) $[M+H]^+$ calcd. for $C_{24}H_{23}N_2$, 339.1861, found 339.1866.

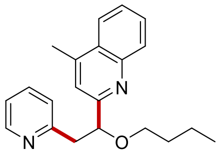

2-(1-Butoxy-2-(pyridin-2-yl)ethyl)-4-methylquinoline (7m): Colorless oil, 57.7 mg, 45% yield, PE/EA = 3/1. **^{1}H NMR** (400 MHz, $CDCl_3$) δ 8.54 (d, J = 4.2 Hz, 1H), 8.07 (d, J = 8.4 Hz, 1H), 7.98 (d, J = 8.1 Hz, 1H), 7.71 – 7.65 (m, 1H), 7.59 – 7.51 (m, 2H), 7.44 (s, 1H), 7.24 (d, J = 7.8 Hz, 1H), 7.15 – 7.07 (m, 1H), 4.95 (t, J = 6.8 Hz, 1H), 3.45 – 3.37 (m, 1H), 3.32 – 3.25 (m, 3H), 2.72 (s, 3H), 1.50 – 1.40 (m, 2H), 1.22 – 1.14 (m, 2H), 0.77 (t, J = 7.4 Hz, 3H). **^{13}C NMR** (100 MHz, $CDCl_3$) δ 162.32, 158.93, 149.22, 147.56, 145.05, 136.06, 129.94, 129.21, 127.80, 126.12, 124.13, 123.81, 121.38, 119.39, 83.56, 69.59, 45.67, 31.86, 19.24, 19.10, 13.88. **HRMS** (m/z) $[M+H]^+$ calcd. for $C_{21}H_{25}N_2O$, 321.1967, found 321.1971.

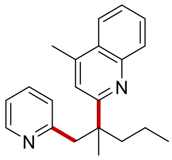

4-Methyl-2-(2-methyl-1-(pyridin-2-yl)pentan-2-yl)quinoline (7n): Colorless oil, 86.4 mg, 71% yield, PE/EA = 6/1. **^{1}H NMR** (700 MHz, $CDCl_3$) δ 8.51 – 8.43 (m, 1H), 8.07 (dd, *J* = 8.4, 0.5 Hz, 1H), 7.95 (dd, *J* = 8.3, 0.9 Hz, 1H), 7.69 – 7.66 (m, 1H), 7.53 – 7.49 (m, 1H), 7.29 (td, *J* = 7.7, 1.9 Hz, 1H), 7.19 (d, *J* = 0.6 Hz, 1H), 7.02 – 6.98 (m, 1H), 6.65 (d, *J* = 7.9 Hz, 1H), 3.56 (d, *J* = 12.9 Hz, 1H), 3.20 (d, *J* = 12.9 Hz, 1H), 2.65 (d, *J* = 0.8 Hz, 3H), 2.10 – 2.04 (m, 1H), 1.70 – 1.65 (m, 1H), 1.45 (s, 3H), 1.25 – 1.20 (m, 1H), 1.04 – 0.97 (m, 1H), 0.82 (t, *J* = 7.3 Hz, 3H). **^{13}C NMR** (100 MHz, $CDCl_3$) δ 166.46, 160.00, 148.63, 147.48, 143.42, 135.52, 130.18, 128.80, 126.62, 125.61, 124.84, 123.59, 121.03, 120.19, 50.10, 45.80, 44.68, 23.19, 19.12, 17.86, 14.92. **HRMS** (m/z) $[M+H]^+$ calcd. for $C_{21}H_{25}N_2$, 305.2018, found 305.2022.

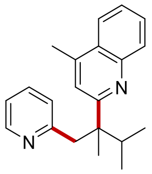

2-(2,3-Dimethyl-1-(pyridin-2-yl)butan-2-yl)-4-methylquinoline (7o): Colorless oil, 65.8 mg, 54% yield, PE/EA = 6/1. **^{1}H NMR** (700 MHz, $CDCl_3$) δ 8.45 – 8.37 (m, 1H), 8.08 (d, J = 8.0 Hz, 1H), 7.94 (dd, J = 8.3, 1.4 Hz, 1H), 7.70 – 7.66 (m, 1H), 7.53 – 7.49 (m, 1H), 7.10 – 7.06 (m, 2H), 6.89 (ddd, J = 7.5, 4.9, 1.2 Hz, 1H), 6.41 – 6.37 (m, 1H), 3.82 (d, J = 12.7 Hz, 1H), 3.15 (d, J = 12.7 Hz, 1H), 2.61 – 2.59 (m, 3H), 2.53 – 2.48 (m, 1H), 1.36 (s, 3H), 1.08 (d, J = 6.9 Hz, 3H), 0.66 (d, J = 6.8 Hz, 3H). **^{13}C NMR** (175 MHz, $CDCl_3$) δ 166.39, 160.58, 148.39, 147.34, 142.99, 135.27, 130.15, 128.75, 126.54, 125.53, 124.64, 123.59, 120.95, 120.76, 48.90, 47.54, 37.84, 19.07, 18.39, 17.85, 17.03. **HRMS** (m/z) $[M+H]^+$ calcd. for $C_{21}H_{25}N_2$, 305.2018, found 305.2019.

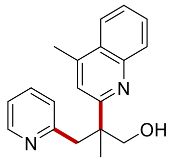

2-Methyl-2-(4-methylquinolin-2-yl)-3-(pyridin-2-yl)propan-1-ol (7p): Colorless oil, 70.5 mg, 60% yield, PE/EA = 2/1. **^{1}H NMR** (700 MHz, $CDCl_3$) δ 8.51 (d, *J* = 4.6 Hz, 1H), 8.03 (d, *J* = 8.4 Hz, 1H), 7.95 (d, *J* = 8.3 Hz, 1H), 7.68 (t, *J* = 7.6 Hz, 1H), 7.53 (t, *J* = 7.6 Hz, 2H), 7.28 (s, 1H), 7.17 (d, *J* = 7.7 Hz, 1H), 7.13 – 7.10 (m, 1H), 4.01 (d, *J* = 11.6 Hz, 1H), 3.67 (d, *J* = 11.6 Hz, 1H), 3.53 (d, *J* = 13.1 Hz, 1H), 3.23 (d, *J* = 13.1 Hz, 1H), 2.67 (s, 3H), 1.33 (s, 3H). **^{13}C NMR** (175 MHz, $CDCl_3$) δ 167.12, 159.18, 148.77, 146.65, 145.08, 136.44, 129.78, 129.47, 127.05, 126.24, 125.62, 123.84, 121.60, 120.29, 69.05, 46.58, 45.94, 22.65, 19.26. **HRMS** (m/z) $[M+H]^+$ calcd. for $C_{19}H_{21}N_2O$, 293.1654, found 293.1658.

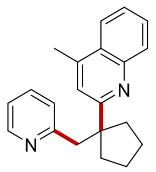

4-Methyl-2-(1-(pyridin-2-ylmethyl)cyclopentyl)quinoline (7q): Colorless oil, 93.1 mg, 77% yield, PE/EA = 6/1. **^{1}H NMR** (700 MHz, $CDCl_3$) δ 8.45 (d, *J* = 4.6 Hz, 1H), 8.05 (d, *J* = 8.4 Hz, 1H), 7.92 (d, *J* = 8.3 Hz, 1H), 7.65 (t, *J* = 7.6 Hz, 1H), 7.49 (t, *J* = 7.5 Hz, 1H), 7.23 (t, *J* = 7.6 Hz, 1H), 7.07 (s, 1H), 7.00 – 6.96 (m, 1H), 6.46 (d, *J* = 7.8 Hz, 1H), 3.34 (s, 2H), 2.60 (s, 3H), 2.36 – 2.31 (m, 2H), 2.11 – 2.06 (m, 2H), 1.83 – 1.79 (m, 2H), 1.68 – 1.62 (m, 2H). **^{13}C NMR** (175 MHz, $CDCl_3$) δ 166.87, 160.06, 148.84, 147.45, 143.54, 135.55, 130.17, 128.92, 126.78, 125.71, 124.59, 123.67, 121.16, 120.98, 55.23, 49.16, 37.02, 24.27, 19.09. **HRMS** (m/z) $[M+H]^+$ calcd. for $C_{21}H_{23}N_2$, 303.1861, found 303.1867.

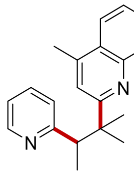

4-Methyl-2-(2-methyl-3-(pyridin-2-yl)butan-2-yl)quinoline (7r): Colorless oil, 74.0 mg, 64% yield, PE/EA = 6/1. **^{1}H NMR** (400 MHz, $CDCl_3$) δ 8.59 – 8.53 (m, 1H), 8.08 (d, J = 8.1 Hz, 1H), 7.96 – 7.91 (m, 1H), 7.70 – 7.64 (m, 1H), 7.52 – 7.47 (m, 1H), 7.44 (td, J = 7.7, 1.9 Hz, 1H), 7.28 (s, 1H), 7.06 (ddd, J = 7.4, 4.9, 1.0 Hz, 1H), 6.99 (d, J = 7.9 Hz, 1H), 3.72 (q, J = 7.1 Hz, 1H), 2.67 – 2.63 (m, 3H), 1.53 (s, 3H), 1.32 (s, 3H), 1.18 (d, J = 7.2 Hz, 3H). **^{13}C NMR** (100 MHz, $CDCl_3$) δ 167.80, 163.98, 148.52, 147.41, 143.37, 135.31, 130.16, 128.73, 126.58, 125.53, 124.37, 123.51, 121.20, 119.77, 50.65, 44.80, 27.18, 22.52, 19.04, 15.29. **HRMS** (m/z) $[M+H]^+$ calcd. for $C_{20}H_{23}N_2$, 291.1861, found 291.1864.

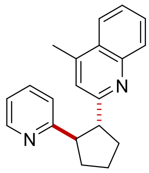

4-Methyl-2-(pyridin-2-yl)cyclopentyl)quinoline (7s): Colorless oil, 88.7 mg, 77% yield, >20:1 dr, PE/EA = 6/1. **^{1}H NMR** (700 MHz, $CDCl_3$) δ 8.53 (d, *J* = 4.4 Hz, 1H), 8.03 (d, *J* = 8.4 Hz, 1H), 7.89 (d, *J* = 8.2 Hz, 1H), 7.63 (t, *J* = 7.5 Hz, 1H), 7.46 (t, *J* = 7.5 Hz, 1H), 7.42 (td, *J* = 7.7, 1.5 Hz, 1H), 7.08 (d, *J* = 7.8 Hz, 1H), 7.02 – 6.98 (m, 2H), 3.80 (dd, *J* = 18.3, 9.6 Hz, 1H), 3.71 (dd, *J* = 18.2, 9.8 Hz, 1H), 2.58 (s, 3H), 2.39 – 2.31 (m, 2H), 2.17 – 2.11 (m, 2H), 2.05 – 2.00 (m, 2H). **^{13}C NMR** (175 MHz, $CDCl_3$) δ 164.17, 149.55, 148.05, 144.05, 136.28, 129.87, 129.01, 127.32, 125.56, 123.81, 123.27, 122.20, 121.34, 55.28, 54.02, 34.76, 34.47, 25.34, 18.92. **HRMS** (m/z) $[M+H]^+$ calcd. for $C_{20}H_{21}N_2$, 289.1705, found 289.1703.

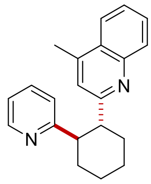

4-Methyl-2-(pyridin-2-yl)cyclohexyl)quinoline (7t): Colorless oil, 104.3 mg, 86% yield, >20:1 dr, PE/EA = 6/1. **^{1}H NMR** (400 MHz, $CDCl_3$) δ 8.39 (d, *J* = 4.4 Hz, 1H), 7.98 (d, *J* = 8.4 Hz, 1H), 7.82 (d, *J* = 8.3 Hz, 1H), 7.62 – 7.56 (m, 1H), 7.44 – 7.38 (m, 1H), 7.32 – 7.27 (m, 1H), 7.03 (d, *J* = 7.8 Hz, 1H), 6.98 (s, 1H), 6.88 – 6.83 (m, 1H), 3.45 – 3.34 (m, 2H), 2.51 (s, 3H), 2.11 – 2.02 (m, 2H), 1.97 – 1.89 (m, 2H), 1.84 – 1.72 (m, 2H), 1.65 – 1.56 (m, 2H). **^{13}C NMR** (100 MHz, $CDCl_3$) δ 164.95, 164.79, 148.92, 147.70, 143.87, 136.10, 129.52, 128.74, 126.96, 125.31, 123.62, 123.00, 121.87, 120.94, 51.25, 50.42, 34.32, 34.20, 26.49, 18.71. **HRMS** (m/z) $[M+H]^+$ calcd. for $C_{21}H_{23}N_2$, 303.1861, found 303.1865.

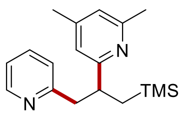

2,4-Dimethyl-6-(1-(pyridin-2-yl)-3-(trimethylsilyl)propan-2-yl)pyridine (4an): Colorless oil, 17.9 mg, 15% yield, PE/EA = 3/1. **^{1}H NMR** (400 MHz, $CDCl_3$) δ 8.53 – 8.46 (m, 1H), 7.43 (td, J = 7.6, 1.8 Hz, 1H), 7.05 – 6.99 (m, 1H), 6.87 (d, J = 7.8 Hz, 1H), 6.72 (s, 1H), 6.61 (s, 1H), 3.36 – 3.27 (m, 1H), 3.19 (dd, J = 13.0, 7.8 Hz, 1H), 3.05 (dd, J = 13.0, 7.1 Hz, 1H), 2.46 (s, 3H), 2.17 (s, 3H), 1.26 – 1.19 (m, 2H), 0.90 – 0.84 (m, 1H), -0.26 (s, 9H). **^{13}C NMR** (100 MHz, $CDCl_3$) δ 164.34, 161.01, 157.47, 149.24, 147.01, 135.87, 123.91, 121.77, 120.93, 120.77, 48.20, 44.52, 24.52, 22.97, 20.90, -1.02. **HRMS** (m/z) $[M+H]^+$ calcd. for $C_{18}H_{27}N_2Si$, 299.1938, found 299.1941.

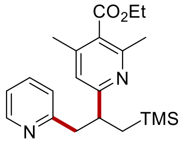

Ethyl-2,4-dimethyl-6-(1-(pyridin-2-yl)-3-(trimethylsilyl)propan-2-yl)nicotinate (4ao): Colorless oil, 47.4 mg, 32% yield, PE/EA = 3/1. **^{1}H NMR** (400 MHz, $CDCl_3$) δ 8.55 – 8.48 (m, 1H), 7.45 (td, J = 7.6, 1.8 Hz, 1H), 7.07 – 7.01 (m, 1H), 6.88 (d, J = 7.8 Hz, 1H), 6.68 (s, 1H), 4.38 (q, J = 7.1 Hz, 2H), 3.40 – 3.31 (m, 1H), 3.17 (dd, J = 13.2, 7.9 Hz, 1H), 3.04 (dd, J = 13.2, 7.0 Hz, 1H), 2.51 (s, 3H), 2.21 (s, 3H), 1.38 (t, J = 7.1 Hz, 3H), 1.25 – 1.18 (m, 1H), 0.90 – 0.84 (m, 1H), -0.22 (s, 9H). **^{13}C NMR** (100 MHz, $CDCl_3$) δ 169.29, 165.38, 160.64, 154.83, 149.33, 144.82, 136.03, 127.01, 123.94, 121.71, 121.07, 61.32, 47.81, 44.33, 23.25, 22.81, 19.60, 14.39, -0.90. **HRMS** (m/z) $[M+H]^+$ calcd. for $C_{21}H_{31}N_2O_2Si$, 371.2149, found 371.2153.

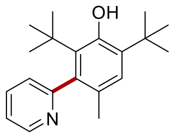

2,6-Di-tert-butyl-4-methyl-3-(pyridin-2-yl)phenol (Int-B): Colorless oil, 44.0 mg, 37% yield, PE/EA = 5/1. **^{1}H NMR** (400 MHz, $CDCl_3$) δ 8.65 – 8.58 (m, 1H), 7.64 (td, J = 7.7, 1.8 Hz, 1H), 7.26 – 7.23 (m, 1H), 7.19 (ddd, J = 7.5, 4.9, 1.1 Hz, 1H), 7.02 (s, 1H), 5.21 (s, 1H), 1.74 (s, 3H), 1.46 (s, 9H), 1.20 (s, 9H). **^{13}C NMR** (100 MHz, $CDCl_3$) δ 163.00, 152.85, 148.66, 138.60, 135.79, 135.31, 133.47, 127.87, 126.53, 126.01, 121.43, 37.05, 34.34, 32.58, 30.48, 21.05. **HRMS** (m/z) $[M+H]^+$ calcd. for $C_{20}H_{28}NO$, 298.2165, found 298.2168.

6. Supplementary Note 5

Gram-scale reactions and synthetic transformations：

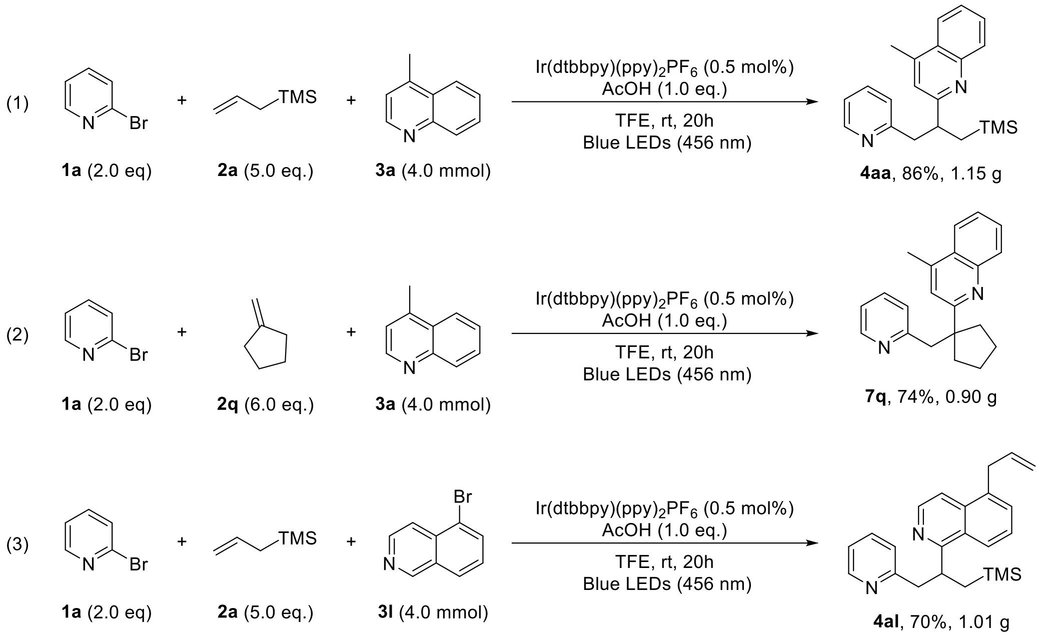

Supplementary Figure 3. Gram-scale reactions.

Following General Procedure: To an oven-dried 100 mL vial was added $Ir(ppy)_2(dtbbpy)PF_6$ (0.02 mmol, 0.5 mol%), bromopyridine **1a** (8.0 mmol, 2.0 eq.), alkene **2** (20 ~ 24 mmol, 5.0 ~ 6.0 eq.), quinoline **3** (4.0 mmol, 1.0 eq.), AcOH (4.0 mmol, 1.0 eq.) and TFE (60 mL, 0.06 M) in the nitrogen glove box. The vial was capped with a septum and wrapped with parafilm. The reaction mixture was stirred for 20 h under visible light irradiation (2 x Kessil PR160, λ_{max}= 456 nm, 40 W, irradiation temperature maintained between 25-30 ˚C). After reaction completed, the crude product was neutralized with saturated $NaHCO_3$ solution or Et_3N and extracted with ethyl acetate. Organic layer was washed with brine solution and dried over anhydrous Na_2SO_4. Removal of the organic solvent in a vacuum rotavapor followed by flash silica gel column chromatographic purification (hexane/ethyl acetate) afforded the desired products **4aa**, **7q** and **4al** in 86% (1.15 g), 74% (0.90 g) and 70% (1.01 g) yields, respectively. The reaction products can be stored stably at room temperature.

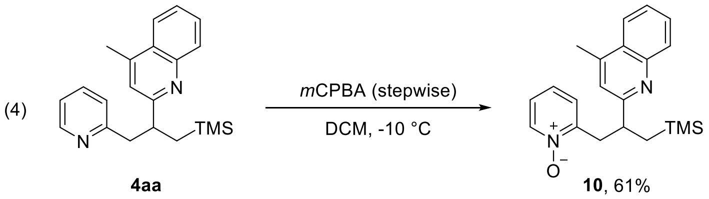

4aa **10**, 61%

Following a modified procedure from reference[1]: To a solution of **4aa** (0.20 mmol, 1.0 eq.) in DCM (4 mL) was added *m*CPBA stepwise (0.24 mmol, 1.2 eq., 3-4 times in 1.5 h) at -10 ˚C. The mixture

was allowed to stir at -10 °C for 3 h. Upon completion, the crude product was neutralized with sodium bicarbonate and extracted with DCM. Organic layer was washed with brine solution and dried over anhydrous sodium sulfate. After concentration in vacuo, the resulting crude product was purified by chromatography by preparative TLC (Silica gel, DCM: MeOH 30:1) to afford 61% yield of the title product **10**.

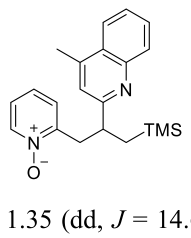

2-(2-(4-Methylquinolin-2-yl)-3-(trimethylsilyl)propyl)pyridine 1-oxide (10): Colorless oil, 42.8 mg, 61% yield. ^{1}**H NMR** (700 MHz, $CDCl_3$) δ 8.25 – 8.17 (m, 1H), 8.04 (d, *J* = 8.4 Hz, 1H), 7.90 (d, *J* = 8.2 Hz, 1H), 7.68 – 7.63 (m, 1H), 7.51 – 7.46 (m, 1H), 7.06 (s, 1H), 7.03 – 6.98 (m, 2H), 6.91 (t, *J* = 7.5 Hz, 1H), 3.83 – 3.76 (m, 1H), 3.45 (dd, *J* = 14.0, 5.4 Hz, 1H), 3.39 (dd, *J* = 14.0, 9.3 Hz, 1H), 2.58 (s, 3H), 1.35 (dd, *J* = 14.6, 9.1 Hz, 1H), 1.07 (dd, *J* = 14.6, 5.9 Hz, 1H), -0.14 (s, 9H). ^{13}**C NMR** (175 MHz, $CDCl_3$) δ 165.30, 151.34, 147.82, 144.57, 139.78, 129.76, 129.19, 127.46, 127.31, 125.77, 125.62, 123.90, 123.65, 122.11, 40.88, 39.78, 24.26, 18.85, -0.69. **HRMS** (m/z) $[M+H]^+$ calcd. for $C_{21}H_{27}N_2OSi$, 351.1893, found 351.1896.

(5)

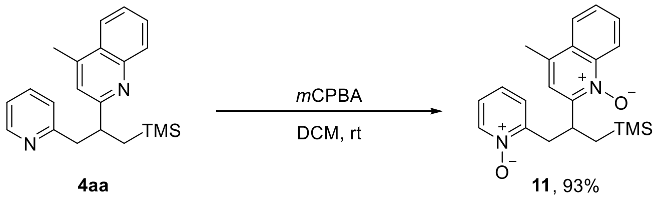

4aa **11**, 93%

Following a modified procedure from reference[1]: To a solution of **4aa** (0.20 mmol, 1.0 eq.) in DCM (2 mL) was added *m*CPBA (0.60 mmol, 3.0 eq.) at 0 °C. The mixture was allowed to stir at room temperature for 4 h. Upon completion, the crude product was neutralized with sodium bicarbonate and extracted with DCM. Organic layer was washed with brine solution and dried over anhydrous sodium sulfate. After concentration in vacuo, the resulting crude product was purified by chromatography by preparative TLC (Silica gel, EA: triethylamine 20:1) to afford 93% yield of the title product **11**.

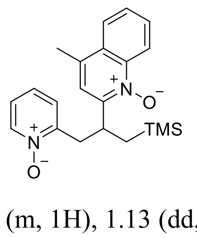

4-Methyl-2-(1-(1-oxidopyridin-2-yl)-3-(trimethylsilyl)propan-2-yl)quinoline 1-oxide (11): Light yellow oil, 68.3 mg, 93% yield. ^{1}**H NMR** (400 MHz, $CDCl_3$) δ 8.80 (d, *J* = 8.7 Hz, 1H), 8.21 – 8.14 (m, 1H), 7.87 (d, *J* = 8.2 Hz, 1H), 7.73 (t, *J* = 7.7 Hz, 1H), 7.60 (t, *J* = 7.5 Hz, 1H), 7.37 (s, 1H), 7.12 (s, 1H), 7.06 – 6.93 (m, 2H), 4.47 (s, 1H), 3.83 – 3.59 (m, 1H), 3.47 (dd, *J* = 15.5, 5.5 Hz, 1H), 2.54 (s, 3H), 1.57 – 1.31 (m, 1H), 1.13 (dd, *J* = 14.7, 6.4 Hz, 1H), -0.10 (s, 9H). ^{13}**C NMR** (100 MHz, $CDCl_3$) δ 150.69, 141.28, 139.39, 134.15, 130.22, 128.54, 127.89, 126.04, 124.69, 123.64, 120.40, 36.07, 18.36, -1.10. **HRMS** (m/z) $[M+H]^+$ calcd. for $C_{21}H_{27}N_2O_2Si$, 367.1842, found 367.1844.

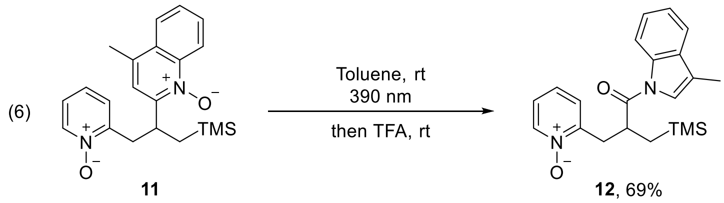

Following a modified procedure from reference[2]: A screw cap vial equipped with a magnetic stir bar and PTFE silicone septum was charged with bis-*N*-oxide **11** (0.20 mmol, 1.0 eq.) and toluene (3 mL) under air. The reaction mixture was stirred for 5 h under purple LED irradiation (Kessil PR160, λ_{max}= 390 nm, 40 W, irradiation temperature maintained between 25-30 ˚C). Then, the light was turned off and TFA (0.20 mmol, 1.0 eq.) was added dropwise while stirring at room temperature for another 5 h. Upon completion, the crude product was neutralized with sodium bicarbonate and extracted with DCM. The combined organic layer was washed with brine, dried over anhydrous sodium sulfate. After concentration in vacuo, the resulting crude product was purified by chromatography by preparative TLC (Silica gel, hexanes/EtOAc = 1:6) to afford 69% yield of the title product **12**.

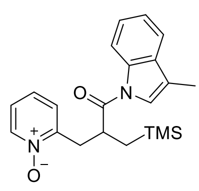

2-(3-(3-Methyl-1H-indol-1-yl)-3-oxo-2-((trimethylsilyl)methyl)propyl)pyridine 1-oxide (12): Light yellow oil, 50.6 mg, 69% yield. **^{1}H NMR** (400 MHz, $CDCl_3$) δ 8.46 (d, *J* = 8.1 Hz, 1H), 8.30 – 8.16 (m, 1H), 7.54 (d, *J* = 0.8 Hz, 1H), 7.45 (d, *J* = 7.2 Hz, 1H), 7.35 – 7.30 (m, 1H), 7.29 – 7.22 (m, 2H), 7.15 – 7.04 (m, 2H), 4.27 – 4.06 (m, 1H), 3.29 (dd, *J* = 12.7, 8.1 Hz, 1H), 3.20 (dd, *J* = 12.7, 6.2 Hz, 1H), 2.23 (d, *J* = 1.1 Hz, 3H), 1.30 (dd, *J* = 14.6, 8.1 Hz, 1H), 0.82 (dd, *J* = 14.6, 6.2 Hz, 1H), 0.03 (s, 9H). **^{13}C NMR** (100 MHz, $CDCl_3$) δ 174.59, 149.41, 139.63, 136.12, 131.87, 128.13, 125.97, 125.07, 124.51, 123.54, 122.06, 118.77, 118.49, 116.97, 37.95, 36.71, 20.45, 9.81, -0.89. **HRMS** (m/z) $[M+H]^+$ calcd. for $C_{21}H_{27}N_2O_2Si$, 367.1842, found 367.1845.

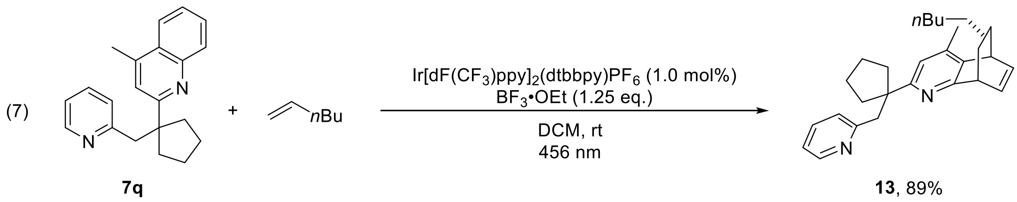

Following a modified procedure from reference[3]: A flame-dried Schlenk flask equipped with a magnetic stir bar was charged with $Ir[dF(CF_3)ppy]_2(dtbbpy)PF_6$ (0.002 mmol, 1.0 mol%), **7q** (0.20 mmol, 1.0 eq.), 1-hexene (1.0 mmol, 5.0 eq.) and DCM (2.0 mL, 0.1 M) in the nitrogen glove box. The reaction mixture was stirred for 24 h under visible light irradiation (Kessil PR160, λ_{max}= 456 nm, 40 W, irradiation temperature maintained between 25-30 ˚C). Upon completion, the crude product was neutralized with saturated $NaHCO_3$ solution and extracted with DCM. Organic layer was washed with brine solution and dried over anhydrous Na_2SO_4. After concentration in vacuo, the

resulting crude product was purified by chromatography by preparative TLC (Silica gel, hexanes/EtOAc = 5:1) to afford 89% yield of the title product **13** (**13a** and **13b**).

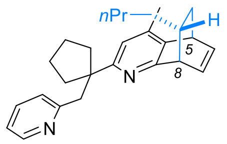

4-Methyl-9-pentyl-2-(1-(pyridin-2-ylmethyl)cyclopentyl)-5,8-dihydro-5,8-ethanoquinoline (13a): Colorless oil, 25.8 mg, 39% yield, an inseparable mixture of diastereoisomer (6.1:1 dr) **^{1}H NMR** (700 MHz, $CDCl_3$) δ 8.45 (d, *J* = 4.8 Hz, 1H), 7.23 (td, *J* = 7.8, 1.6 Hz, 1H), 6.99 – 6.96 (m, 1H), 6.61 (t, *J* = 6.9 Hz, 1H), 6.52 (s, 1H), 6.44 (t, *J* = 6.5 Hz, 1H), 6.33 (d, *J* = 7.8 Hz, 1H), 4.05 (d, *J* = 4.3 Hz, 1H), 3.93 (d, *J* = 6.0 Hz, 1H), 3.32 (d, *J* = 12.9 Hz, 1H), 3.17 (d, *J* = 12.9 Hz, 1H), 2.20 (s, 3H), 2.12 – 2.08 (m, 1H), 2.06 – 2.02 (m, 1H), 1.96 – 1.91 (m, 2H), 1.89 – 1.84 (m, 2H), 1.79 – 1.73 (m, 2H), 1.66 – 1.59 (m, 1H), 1.58 – 1.53 (m, 1H), 1.40 – 1.34 (m, 1H), 1.33 – 1.29 (m, 1H), 1.21 – 1.16 (m, 2H), 0.97 – 0.91 (m, 1H), 0.91 – 0.86 (m, 1H), 0.82 (t, *J* = 7.2 Hz, 3H), 0.75 – 0.70 (m, 1H). **^{13}C NMR** (175 MHz, $CDCl_3$) δ 161.39, 161.28, 160.51, 148.61, 137.82, 136.26, 135.13, 134.63, 133.08, 124.43, 120.85, 119.40, 54.15, 48.92, 47.78, 37.70, 37.52, 36.55, 36.42, 35.40, 33.68, 30.03, 29.84, 23.75, 23.68, 22.93, 17.99, 14.23. **HRMS** (m/z) $[M+H]^+$ calcd. for $C_{27}H_{35}N_2$, 387.2800, found 387.2803.

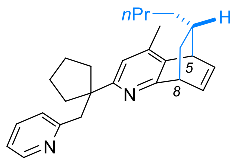

4-Methyl-10-pentyl-2-(1-(pyridin-2-ylmethyl)cyclopentyl)-5,8-dihydro-5,8-ethanoquinoline (13b): Colorless oil, 33.0 mg, 50% yield, an inseparable mixture of diastereoisomer (6.8:1 dr). **^{1}H NMR** (700 MHz, $CDCl_3$) δ 8.42 (d, *J* = 4.2 Hz, 1H), 7.14 (td, *J* = 7.7, 1.7 Hz, 1H), 6.96 – 6.93 (m, 1H), 6.55 – 6.49 (m, 2H), 6.44 (s, 1H), 6.11 (d, *J* = 7.8 Hz, 1H), 4.00 – 3.97 (m, 1H), 3.95 – 3.91 (m, 1H), 3.25 (d, *J* = 12.6 Hz, 1H), 3.08 (d, *J* = 12.6 Hz, 1H), 2.15 (s, 3H), 2.06 – 2.03 (m, 1H), 2.02 – 1.98 (m, 2H), 1.91 – 1.87 (m, 1H), 1.86 – 1.77 (m, 3H), 1.69 – 1.64 (m, 1H), 1.62 – 1.58 (m, 1H), 1.32 – 1.29 (m, 2H), 1.23 – 1.21 (m, 2H), 0.98 – 0.94 (m, 1H), 0.93 – 0.90 (m, 1H), 0.86 (t, *J* = 7.1 Hz, 3H), 0.84 – 0.82 (m, 1H), 0.77 – 0.73 (m, 1H). **^{13}C NMR** (175 MHz, $CDCl_3$) δ 163.51, 161.42, 160.43, 148.54, 140.36, 136.09, 134.81, 134.63, 130.47, 124.43, 120.79, 119.44, 54.42, 49.22, 43.39, 40.39, 38.27, 37.51, 36.20, 35.98, 33.20, 30.42, 29.84, 23.85, 23.70, 23.07, 17.94, 14.25. **HRMS** (m/z) $[M+H]^+$ calcd. for $C_{27}H_{35}N_2$, 387.2800, found 387.2805.

(8) 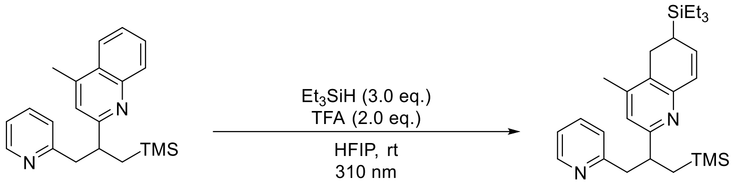

4aa **14**, 76%, 1.4:1 dr

Following a modified procedure from reference[4]: A flame-dried Schlenk flask equipped with a magnetic stir bar was charged with **4aa** (0.20 mmol, 1.0 eq.), Et_3SiH (0.60 mmol, 3.0 eq.), TFA (0.40 mmol, 2.0 eq.) and HFIP (2.0 mL, 0.1 M) in the nitrogen glove box. The reaction mixture was stirred for 20 h under UVA light irradiation (310 nm, 12 W, irradiation temperature maintained between 25-30 ˚C). Upon completion, the crude product was neutralized with saturated $NaHCO_3$ solution and extracted with DCM. Organic layer was washed with brine solution and dried over anhydrous Na_2SO_4. After concentration in vacuo, the resulting crude product was purified by

chromatography by preparative TLC (Silica gel, hexanes/EtOAc = 7:1) to afford 76% yield (1.4:1 dr) of the title product **14**.

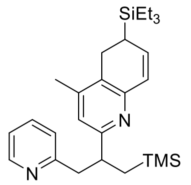

4-Methyl-2-(1-(pyridin-2-yl)-3-(trimethylsilyl)propan-2-yl)-6-(triethylsilyl)-5,6-dihydroquinoline (14): Colorless oil, 68.5 mg, 76% yield, an inseparable mixture of diastereoisomer (1.4:1 dr). **^{1}H NMR** (400 MHz, $CDCl_3$) δ 8.52 – 8.44 (m, 1H), 7.43 – 7.33 (m, 1H), 7.02 – 6.96 (m, 1H), 6.87 (d, *J* = 7.8 Hz, 0.4H), 6.82 (d, *J* = 7.8 Hz, 0.6H), 6.57 (s, 0.4H), 6.53 – 6.40 (m, 1.6H), 6.28 – 6.16 (m, 1H), 3.31 – 3.11 (m, 2H), 3.05 – 2.92 (m, 2H), 2.84 – 2.76 (m, 1H), 2.11 – 2.04 (m, 4H), 1.26 – 1.18 (m, 1H), 0.91 – 0.79 (m, 10H), 0.56 – 0.33 (m, 6H), -0.20 – -0.26 (m, 9H). **^{13}C NMR** (100 MHz, $CDCl_3$) δ 161.60, 161.33, 161.08, 161.02, 152.79, 152.67, 149.14, 149.12, 142.95, 142.81, 136.07, 135.98, 135.83, 135.73, 127.38, 127.33, 126.19, 126.16, 123.97, 122.74, 122.61, 120.85, 48.29, 48.22, 44.2, 44.04, 24.20, 24.17, 23.19, 23.16, 22.89, 22.82, 18.71, 18.68, 7.63, 2.53, -0.90, -0.93. **HRMS** (m/z) $[M+H]^+$ calcd. for $C_{27}H_{43}N_2Si_2$, 451.2965, found 451.2969.

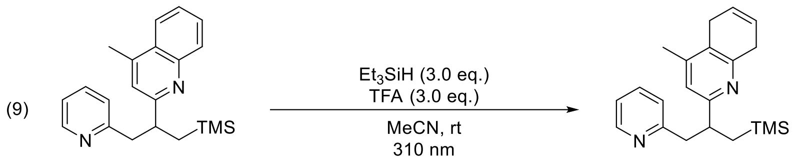

Following a modified procedure from reference[4]: A flame-dried Schlenk flask equipped with a magnetic stir bar was charged with **4aa** (0.20 mmol, 1.0 eq.), Et_3SiH (0.60 mmol, 3.0 eq.), TFA (0.60 mmol, 3.0 eq.) and MeCN (1.0 mL, 0.2 M) in the nitrogen glove box. The reaction mixture was stirred for 20 h under UVA light irradiation (310 nm, 12 W, irradiation temperature maintained between 25-30 ˚C). Upon completion, the crude product was neutralized with saturated $NaHCO_3$ solution and extracted with DCM. Organic layer was washed with brine solution and dried over anhydrous Na_2SO_4. After concentration in vacuo, the resulting crude product was purified by chromatography by preparative TLC (Silica gel, hexanes/EtOAc = 7:1) to afford 81% yield of the title product **15**.

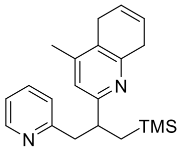

4-Methyl-2-(1-(pyridin-2-yl)-3-(trimethylsilyl)propan-2-yl)-5,8-dihydroquinoline (15): Colorless oil, 54.4 mg, 81% yield. **^{1}H NMR** (400 MHz, $CDCl_3$) δ 8.48 (dd, *J* = 4.8, 0.7 Hz, 1H), 7.41 (td, *J* = 7.6, 1.8 Hz, 1H), 7.03 – 6.96 (m, 1H), 6.88 (d, *J* = 7.8 Hz, 1H), 6.66 (s, 1H), 5.96 – 5.88 (m, 1H), 5.87 – 5.76 (m, 1H), 3.53 – 3.43 (m, 2H), 3.34 – 3.25 (m, 1H), 3.24 – 3.13 (m, 3H), 3.02 (dd, *J* = 13.1, 7.2 Hz, 1H), 2.09 (s, 3H), 1.19 (dd, *J* = 14.5, 10.8 Hz, 1H), 0.85 (dd, *J* = 14.5, 4.3 Hz, 1H), -0.26 (s, 9H). **^{13}C NMR** (100 MHz, $CDCl_3$) δ 162.09, 160.92, 152.81, 149.16, 145.03, 135.89, 125.44, 125.21, 123.88, 123.34, 121.92, 120.90, 48.15, 44.12, 33.17, 27.26, 22.86, 18.85, -0.99. **HRMS** (m/z) $[M+H]^+$ calcd. for $C_{21}H_{29}N_2Si$, 337.2100, found 337.2104.

7. Supplementary Discussion

Reaction development and mechanistic studies:

7.1 UV-vis absorption spectroscopy

UV-vis absorption experiments were performed on various reagents and combinations in TFE solvent. For example, black line is Ir(dtbbpy)(ppy)$_2$PF$_6$ **[Ir-I]** (5*10^{-4} M); red line is 2-bromopyridine **1a** (0.05 M); brown line is [**1a**+AcOH] (molar ratio 1:1, 0.05 M); pink line is 4-methylquinoline **3a** (0.05 M); green line is [**3a**+AcOH] (molar ratio 1:1, 0.05 M); blue line is [**1a**+**3a**] (molar ratio 1:1, 0.05 M).

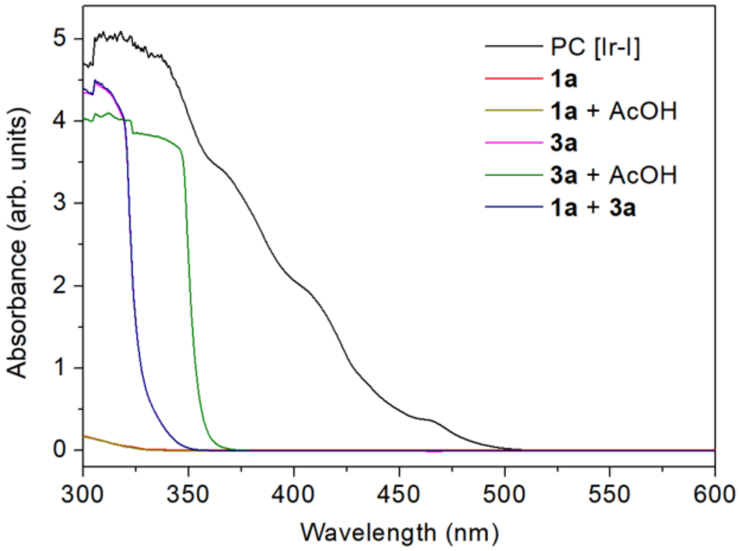

Supplementary Figure 4. UV-vis absorption spectroscopy.

7.2 Stern-Volmer fluorescence quenching studies

Stern-Volmer experiments for all the components of the reaction mixture were carried out to monitor the emission intensity of solutions of Ir(ppy)$_2$(dtbbpy)PF$_6$ (0.5 mM) containing variable amounts of the quencher in trifluoroethanol (TFE). The emmision intensity at 550 nm was collected with excited wavelength of 430 nm in TFE using a PTI QM-400 Spectrofluorophotometer. After degassing the sample with a stream of argon for 10 minutes, the emission intensity of the sample was collected and plots were constructed according to the Stern-Volmer equation $I_0/I = 1 + Kqt_0[Q]$.

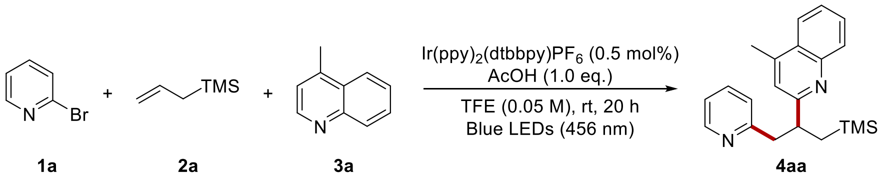

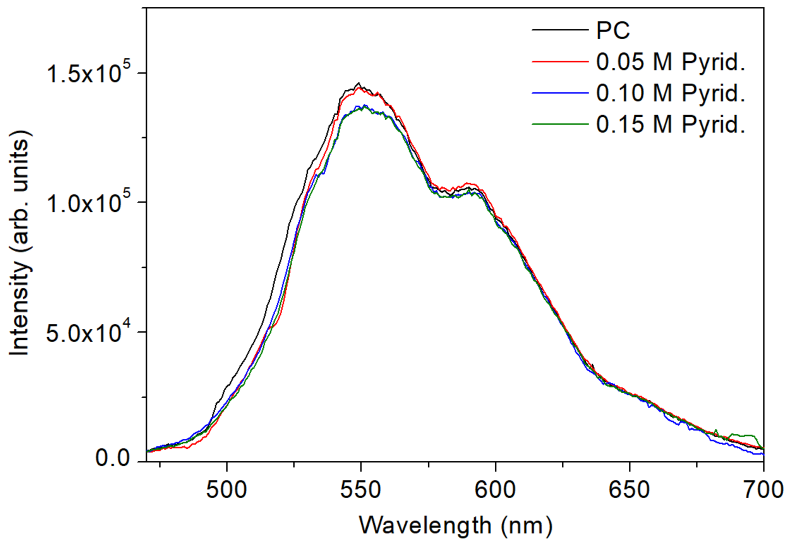

Supplementary Figure 5. Ir(ppy)$_2$(dtbbpy)PF$_6$ emission quenching with halopyridine **1a**.

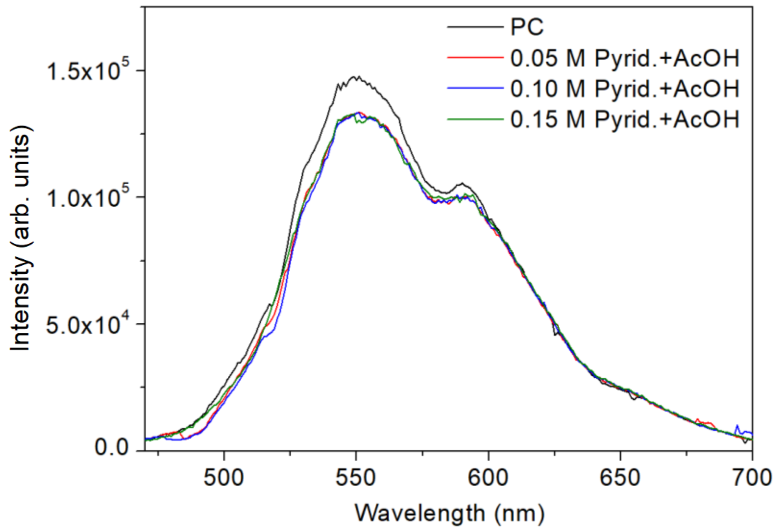

Supplementary Figure 6. Ir(ppy)$_2$(dtbbpy)PF$_6$ emission quenching with [**1a**+AcOH] combine (molar ratio 1:1).

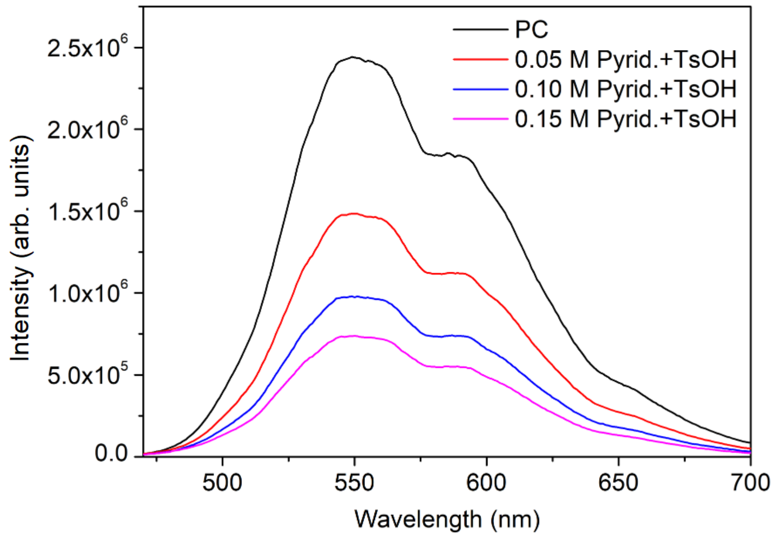

Supplementary Figure 7. Ir(ppy)$_2$(dtbbpy)PF$_6$ emission quenching with [**1a**+TsOH] combine (molar ratio 1:1).

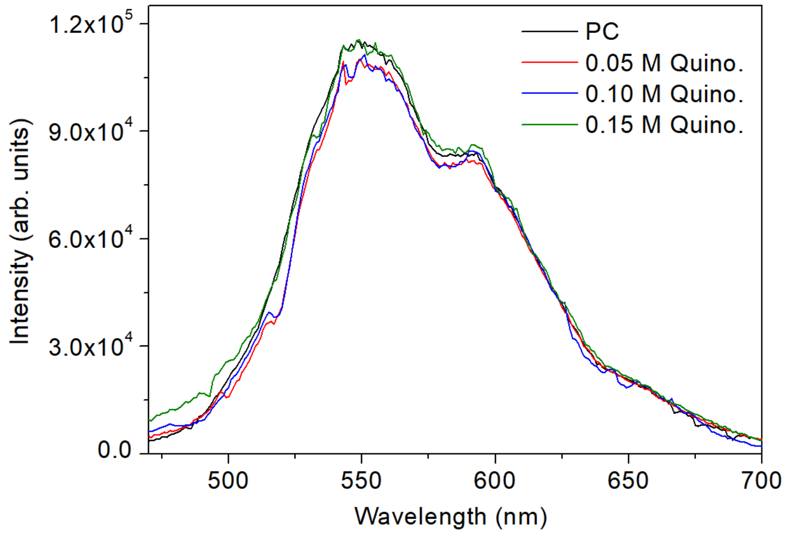

Supplementary Figure 8. Ir(ppy)$_2$(dtbbpy)PF$_6$ emission quenching with quinoline **3a**.

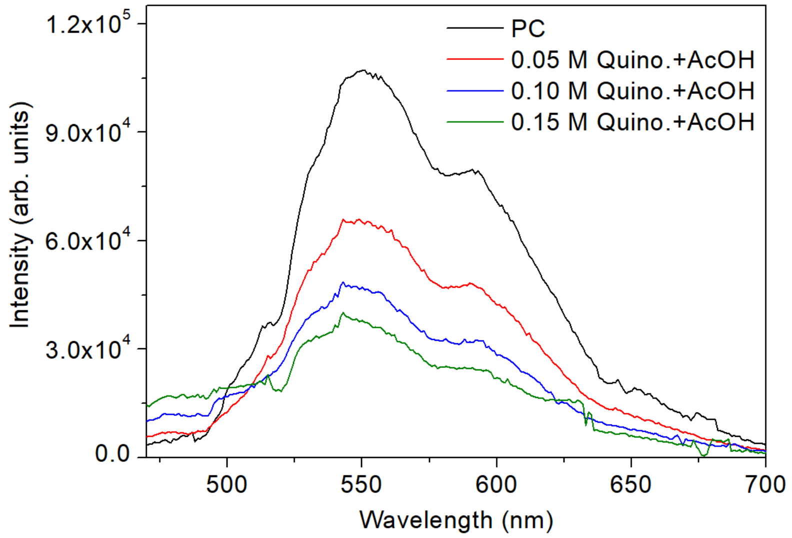

Supplementary Figure 9. Ir(ppy)$_2$(dtbbpy)PF$_6$ emission quenching with [**3a**+AcOH] combine (molar ratio 1:1).

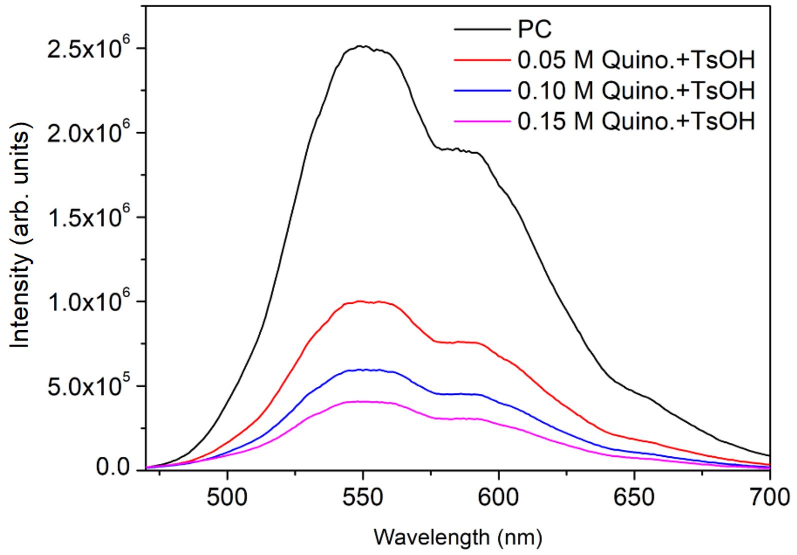

Supplementary Figure 10. Ir(ppy)$_2$(dtbbpy)PF$_6$ emission quenching with [**3a**+TsOH] combine (molar ratio 1:1).

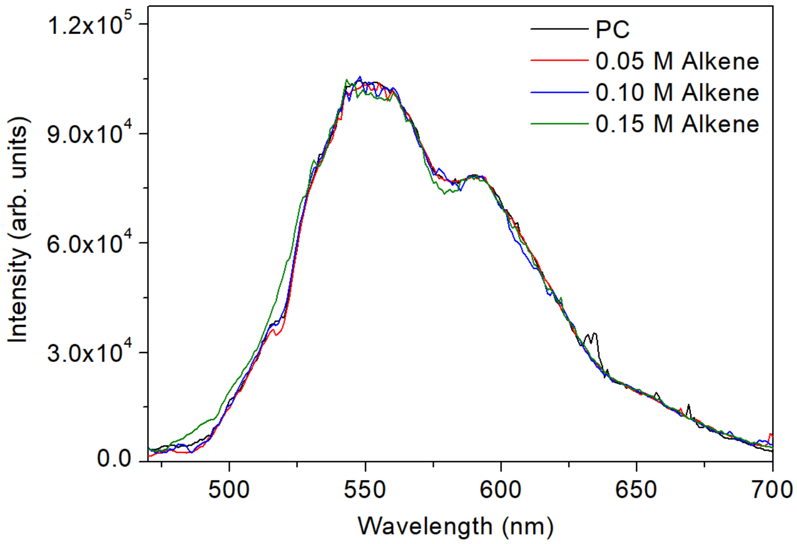

Supplementary Figure 11. Ir(ppy)$_2$(dtbbpy)PF$_6$ emission quenching with alkene **2a**.

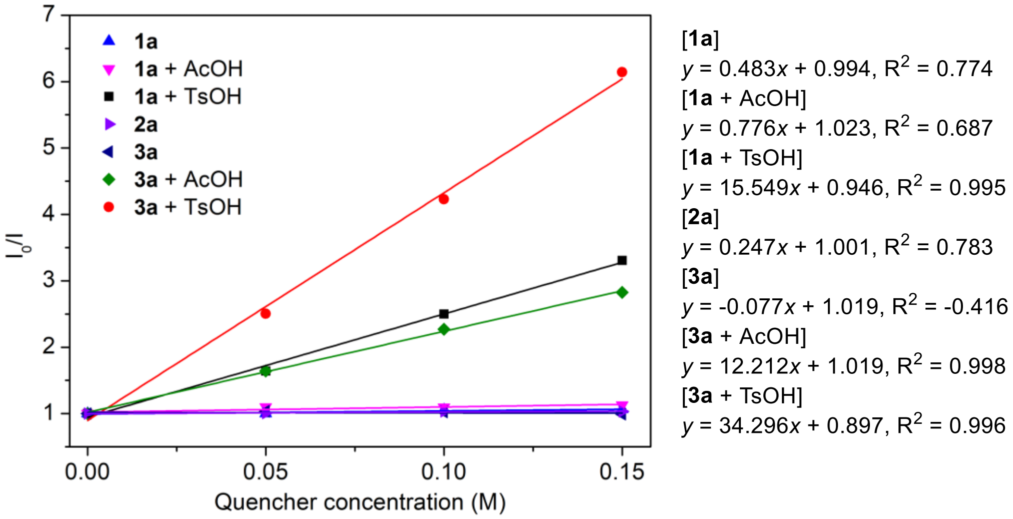

Supplementary Figure 12. Stern-Volmer quenching studies.

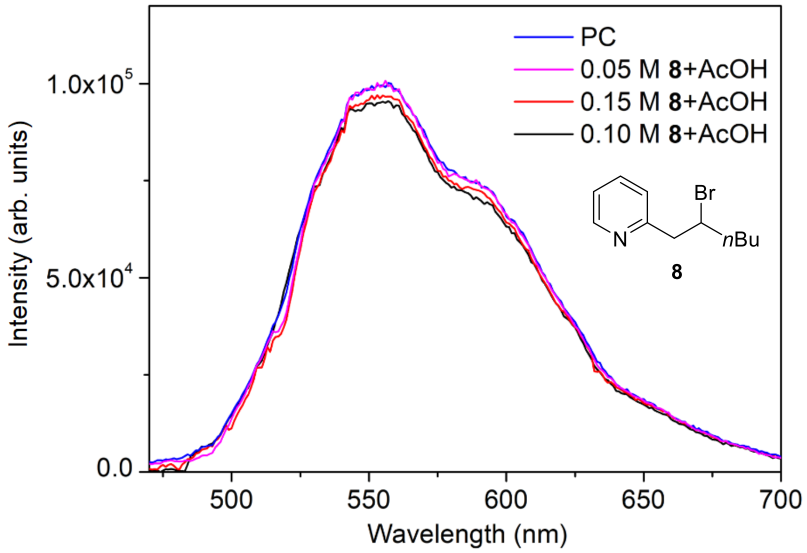

Supplementary Figure 13. Ir(ppy)$_2$(dtbbpy)PF$_6$ emission quenching with [**8**+AcOH] combine (molar ratio 1:1).

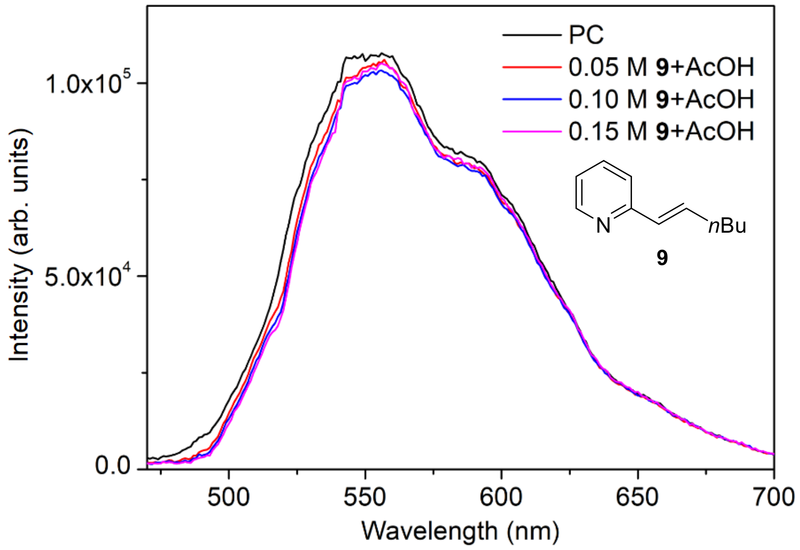

Supplementary Figure 14. Ir(ppy)$_2$(dtbbpy)PF$_6$ emission quenching with [**9**+AcOH] combine (molar ratio 1:1).

7.3 Cyclic Voltammetry

A standard three-electrode cell configuration was used to collect cyclic voltammograms at room temperature with a CHI660e electrochemical workstation. A 3 mm glassy carbon disc electrode as the working electrode, a saturated calomel electrode (SCE) as the reference electrode, and a platinum wire as the counter electrode were employed. Every electrolyte solution contains 0.1 M tetrabutylammonium hexafluorophosphate (TBAPF$_6$). The electrolyte solutions were degassed with N_2 for 5 minutes. Measurement of the reduction potential was tested by cathodic reduction scan.

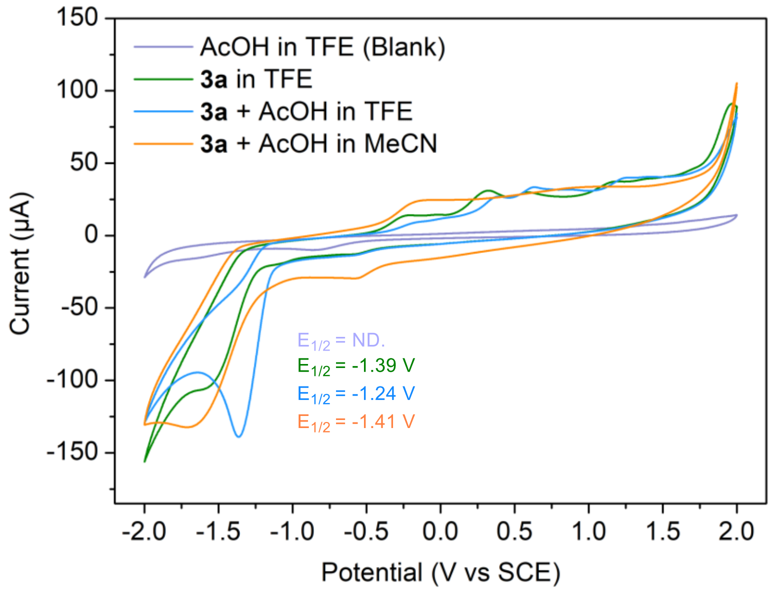

Supplementary Figure 15. Cyclic voltammograms of AcOH (0.01 M) in TFE, **3a** (0.01 M) in TFE, [**3a**+AcOH] (1:1 molar ratio, 0.01 M) in TFE, and [**3a**+AcOH] (1:1 molar ratio, 0.01 M) in MeCN.

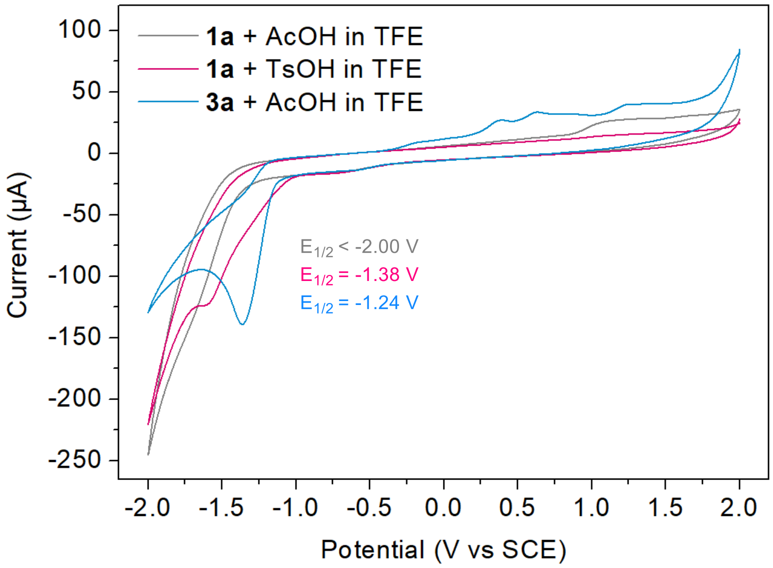

Supplementary Figure 16. Cyclic voltammograms of [**1a**+AcOH] (1:1 molar ratio, 0.01 M) in TFE, [**1a**+TsOH] (1:1 molar ratio, 0.01 M) in TFE, and [**3a**+AcOH] (1:1 molar ratio, 0.01 M) in TFE.

7.4 Light on/off experiments

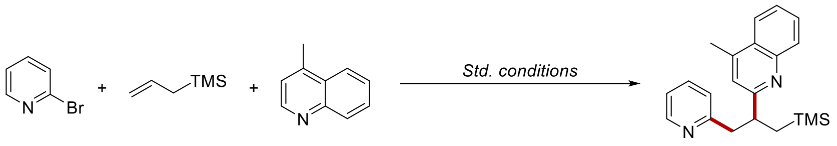

To an oven-dried 4 mL vial was added Ir(ppy)$_2$(dtbbpy)PF$_6$ (0.001 mmol, 0.5 mol%), 2-bromopyridine **1a** (0.40 mmol, 2.0 equiv.), alkene **2a** (1.0 mmol, 5.0 equiv.), 4-methylquinoline **3a** (0.20 mmol, 1.0 eq.), AcOH (0.2 mmol, 1.0 eq.), dimethyl terephthalate as internal standard (0.1 mmol) and TFE (4 mL, 0.05 M) in the nitrogen glove box. The vial was capped with a septum and wrapped with parafilm. The reaction was sequentially stirred under visible light irradiation (Kessil PR160, λ_{max}= 456 nm, 40 W, irradiation temperature maintained between 25-30 °C) and the dark atmosphere. Every one hours an aliquot of 10 μL was removed *via* syringe and analyzed by GC-FID. After a total of 8 h the determined yields were plotted against the reaction time.

Time (h)	0	1	2	3	4	5	6	7	8
Yield **4aa** (%)	0	12	12	30	30	48	48	59	60

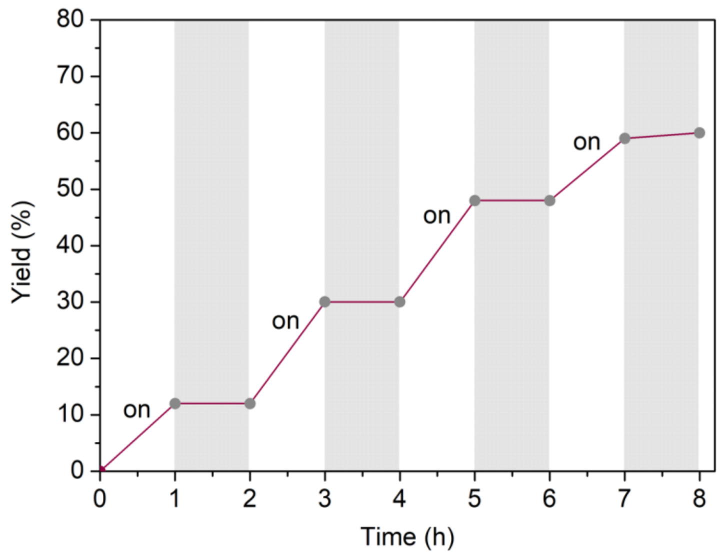

Supplementary Figure 17. Effect of Blue LEDs irradiation.

7.5 Quantum yield measurements (Blue LEDs)

(a) Determination of the photon flux of Kessil Blue LEDs (456 nm)：

Kessil LEDs (λ_{max} = 456 nm) was used with 25% intensity for measurement of quantum yield. According the reported procedure of Yoon,[5] the photon flux of the LEDs (λ_{max} = 456 nm) was determined by standard ferrioxalate actinometry. A 0.15 M solution of ferrioxalate was prepared by dissolving potassium ferrioxalate hydrate (0.737 g) in H_2SO_4 (10 mL of a 0.05 M solution). A buffered solution of 1,10-phenanthroline was prepared by dissolving 1,10-phenanthroline (5.0 mg) and sodium acetate (1.13 g) in H_2SO_4 (5.0 mL of a 0.5 M solution). Both solutions were stored in the dark. To determine the photon flux of Kessil LEDs, the ferrioxalate solution (2.0 mL) was placed in a cuvette and irradiated for 90 s at λ_{max} = 456 nm (25% intensity). After irradiation, the phenanthroline solution (0.35 mL) was added to the cuvette and the mixture was allowed to stir in the dark for 1 h to allow the ferrous ions to completely coordinate to the phenanthroline. The absorbance of the solution was measured at 510 nm. Three groups of parallel experiments were carried out to calculate the average value. A non-irradiated sample was also prepared and the absorbance at 510 nm was measured. And the results were summarized in Supplementary Table 9. Conversion was calculated using eq (1).

Supplementary Table 9. Absorbance of ferrioxalate samples at $A_{510\ nm}$

Irradiation-1	Irradiation-2	Irradiation-3	Non-Irradiation	$\Delta A_{510\ nm}$
2.7809	2.7982	2.5263	1.4885	1.2133
Average value = 2.7018				

$$mol\ of\ Fe^{2+} = \frac{V \times \Delta A_{510\ nm}}{l \times \varepsilon} = \frac{(0.00235\ L) \times 1.2133}{(1.0\ cm) \times (11100\ \frac{L}{mol \cdot cm})} = 2.57\ \times 10^{-7}$$

Supplementary equation (1)

"V" is the total volume (0.00235 L) of the solution after addition of phenanthroline, "ΔA" is the difference in absorbance at 510 nm between the irradiated and non-irradiated solutions, "l" is the path length of cuvette (1.00 cm), and "ε" is the molar absorptivity of the ferrioxalate actinometer at 510 nm (11100 L·mol^{-1}cm^{-1}).[6] The photon flux can be calculated using eq (2) and (3).

$$f = 1 - 10^{-A_{456\ nm}} = 1 - 10^{-2.1219} = 0.9924$$

Supplementary equation (2)

$$Photo\ flux_{Kessil\ 456\ nm} = \frac{mol\ of\ Fe^{2+}}{\emptyset \times t \times f} = \frac{2.57 \times 10^{-7}}{(0.84) \times (90\ s) \times 0.9924} = 3.43 \times 10^{-9}\ einstein/s$$

Supplementary equation (3)

"Φ" is the quantum yield for the ferrioxalate actinometer (0.84 at λ = 456 nm),[7] "t" is the irradiation time (90 s), and "f" is the fraction of light absorbed at 456 nm by the ferrioxalate actinometer, which is calculated using eq (2) where $A_{456\ nm}$ is the absorbance of the ferrioxalate solution at 456 nm. An absorption spectrum gave an $A_{456\ nm}$ value of 2.1219 (Supplementary Figure 18, black curve), indicating that the fraction of absorbed light (f) is 0.9924. The photon flux was thus calculated (average of three experiments) to be 3.43 × 10^{-9} einsteins·s^{-1}.

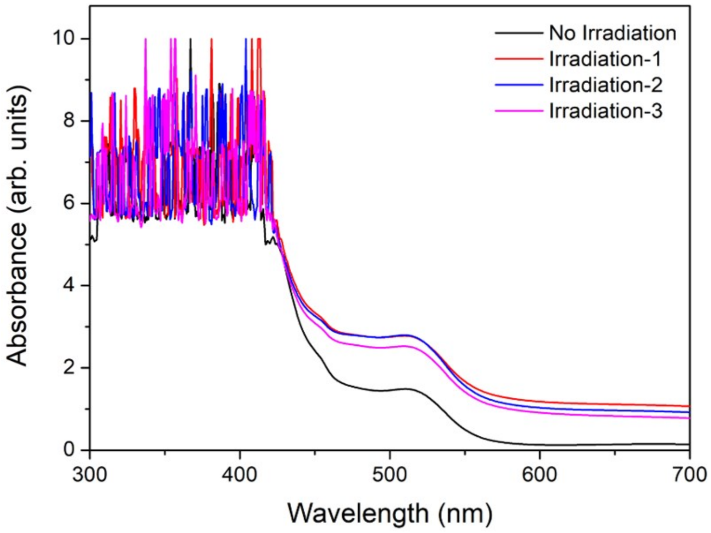

Supplementary Figure 18. Absorption spectra of irradiation and non-irradiation experiments.

(b) Determination of the reaction quantum yield:

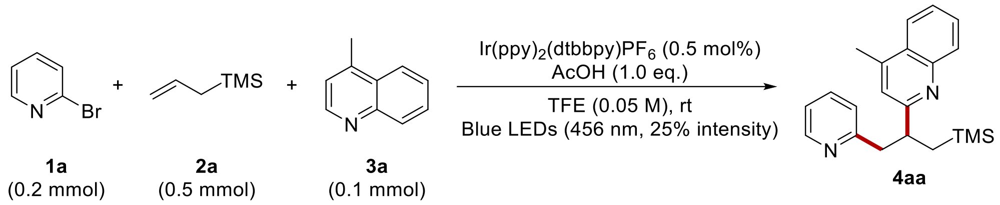

The reaction mixture was stirred and irradiated by Kessil LEDs (λ = 456 nm, 25% intensity) for 7200 s. The yield of product **4aa** was determined by GC-FID analysis of the crude reaction mixture using mesitylene as internal standard. The yield of **3aa** was determined to be 14.5% (0.0145×10^{-3} mol). The reaction quantum yield "Φ" was determined using eq (5) where the photon flux is 3.43×10^{-9} einsteins·s^{-1} (determined by actinometry as described above), "t" is the reaction time (7200 s) and "f" is the fraction of incident light absorbed by the catalyst, determined using eq (4). An absorption spectrum of the catalyst $Ir(ppy)_2(dtbbpy)PF_6$ (PC Ir-I, 0.00025 M in TFE) gave an absorbance value of 0.4861 at 456 nm (Supplementary Figure 19), indicating that the fraction of light absorbed by the photocatalyst (f) is 0.6735. The reaction quantum yield (Φ) was calculated to be 0.87.

$$f = 1 - 10^{-A_{456\,nm}} = 1 - 10^{-0.4861} = 0.6735$$

Supplementary equation (4)

$$\emptyset = \frac{mol\ of\ \mathbf{4aa}}{photo\ flux_{kessil\ 456\,nm} \times t \times f} = \frac{0.0145 \times 10^{-3}\ mol}{3.43 \times 10^{-9}\ einstein \cdot s^{-1} \times 7200\ s \times 0.6735} = 0.87$$

Supplementary equation (5)

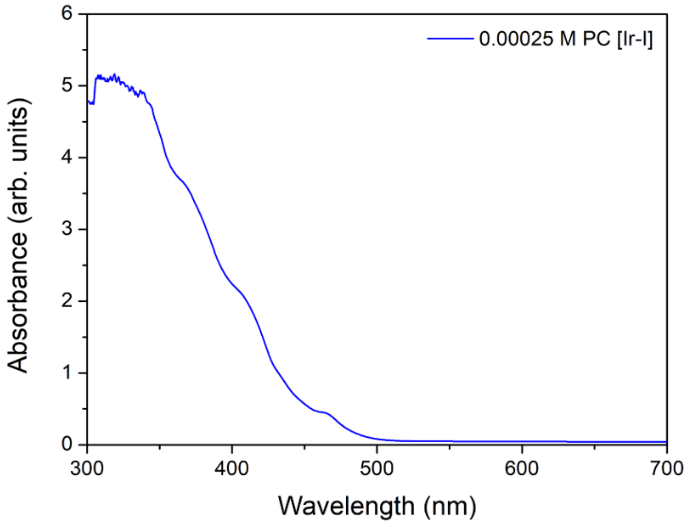

Supplementary Figure 19. Absorption spectra of 0.00025 M solution of $Ir(ppy)_2(dtbbpy)PF_6$ in TFE.

7.6 Radical trapping experiments

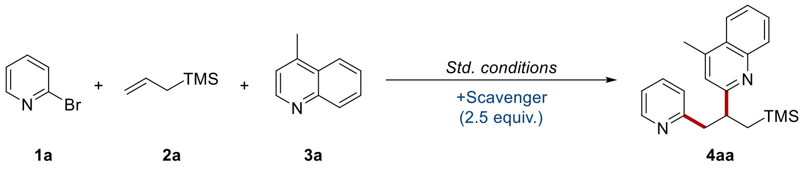

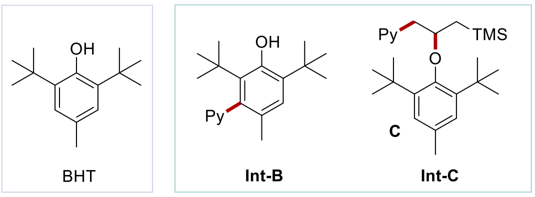

Radical scavenger	Yield **4aa**
None	92%
TEMPO	Trace
BHT	33% with **Int-B, C**

Detected by HRMS or NMR

Supplementary Figure 20. Radical trapping experiments.

To an oven-dried 4 mL vial was added Ir(ppy)$_2$(dtbbpy)PF$_6$ (0.001 mmol, 0.5 mol%), 2-bromopyridine **1a** (0.40 mmol, 2.0 equiv.), alkene **2a** (1.0 mmol, 5.0 equiv.), 4-methylquinoline **3a** (0.20 mmol, 1.0 eq.), AcOH (0.20 mmol, 1.0 eq.), BHT or TEMPO (0.50 mmol, 2.5 eq.) and TFE (4 mL, 0.05 M) in the nitrogen glove box. The vial was capped with a septum and wrapped with parafilm. The reaction mixture was stirred for 20 h under visible light irradiation (Kessil PR160, λ_{max}= 456 nm, 40 W, irradiation temperature maintained between 25-30 °C). After reaction completed, the crude product was neutralized with saturated $NaHCO_3$ solution or Et_3N and extracted with ethyl acetate. Organic layer was washed with brine solution and dried over anhydrous Na_2SO_4. Removal of the organic solvent in a vacuum rotavapor followed by silica chromatography. The intermediates were analyzed by NMR and HRMS (m/z) analysis.

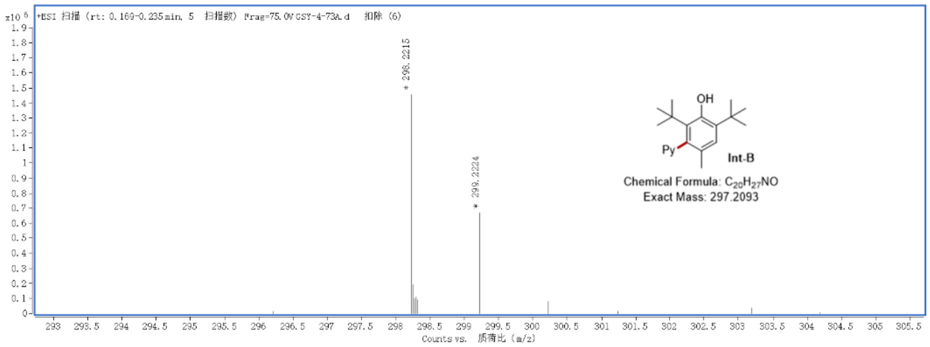

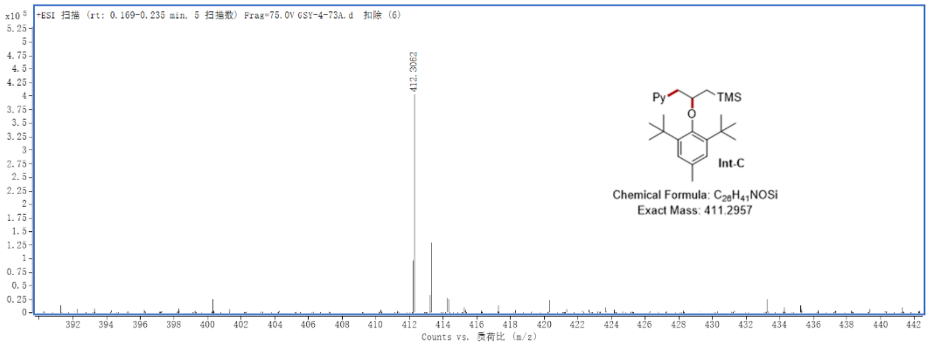

Supplementary Figure 21. HRMS (m/z) (ESI) spectra of BHT-adducts **Int-B** and **Int-C**.

7.7 Kinetic studies and intermediates exploration

a) Kinetic studies

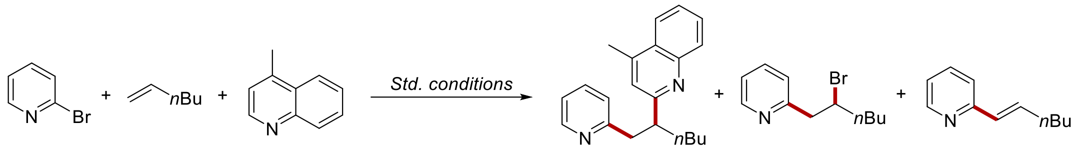

1a **2b** **3a** **7a** **8** **9**

To an oven-dried 4 mL vial was added Ir(ppy)$_2$(dtbbpy)PF$_6$ (0.001 mmol, 0.5 mol%), 2-bromopyridine **1a** (0.40 mmol, 2.0 equiv.), 1-hexene **2b** (1.0 mmol, 5.0 equiv.), 4-methylquinoline **3a** (0.20 mmol, 1.0 eq.), AcOH (0.2 mmol, 1.0 eq.), dimethyl terephthalate as internal standard (0.1 mmol) and TFE (4 mL, 0.05 M) in the nitrogen glove box. The vial was capped with a septum and wrapped with parafilm. The reaction was sequentially stirred under visible light irradiation (Kessil PR160, λ_{max}= 456 nm, 40 W, irradiation temperature maintained between 25-30 °C). Every 1-2 hours an aliquot of 10 µL was removed *via* syringe and analyzed by GC-FID. After a total of 14 h the determined yields were plotted against the reaction time.

Time (h)	0	1	2	3	4	6	8	10	12	14
Yield **7a** (%)	0	8.5	12.3	18.3	23.9	31.4	35.9	38.3	39.9	42.8
Yield **8** (%)	0	0	0	0	0	0	0	0	0	0
Yield **9** (%)	0	2.4	3.0	3.4	5.2	7.5	8.7	9.4	10.0	10.7

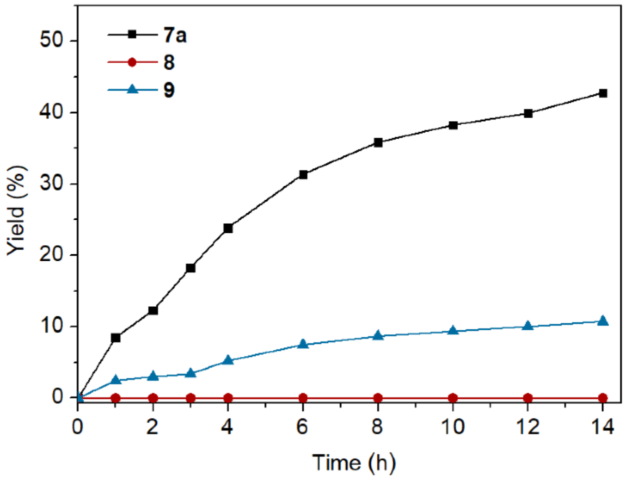

Supplementary Figure 22. Kinetic studies.

b) Intermediates exploration

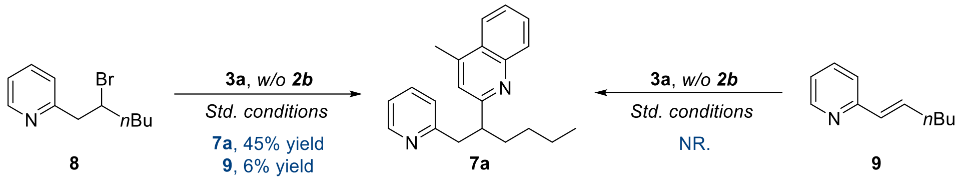

To an oven-dried 4 mL vial was added Ir(ppy)$_2$(dtbbpy)PF$_6$ (0.001 mmol, 0.5 mol%), side-products **8** or **9** (0.40 mmol, 2.0 equiv.), 4-methylquinoline **3a** (0.20 mmol, 1.0 eq.), AcOH (0.2 mmol, 1.0 eq.) and TFE (4 mL, 0.05 M) in the nitrogen glove box. The vial was capped with a septum and wrapped with parafilm. The reaction was sequentially stirred under visible light irradiation (Kessil PR160, λ_{max}= 456 nm, 40 W, irradiation temperature maintained between 25-30 °C). The reaction mixture was stirred under visible light irradiation for 20 h and the yields were determined by GC-FID analysis with 1,3,5-trimethoxybenzene as internal standard.

7.8 Impact of varying amounts of additives on chemoselectivity

Supplementary Table 10. Influence of the TsOH dosage[a]

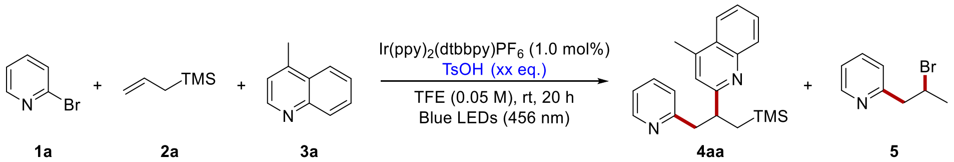

Entry	TsOH (eq.)	Yield of product (%)	
		**4aa**	**5**[b]
1	0.2	82	0
2	0.4	81	0
3	0.6	72	0
4	0.8	54	0
5	1.0	33	0.5
6	1.2	5	1
7	1.4	1	5
8	1.6	0	15
9	1.8	0	24
10	2.0	0	41

[a] Reaction conditions: **1a** (0.20 mmol), **2a** (0.5 mmol), **3a** (0.10 mmol), Ir(ppy)$_2$(dtbbpy)PF$_6$ (1.0 mol%), TsOH (xx mmol), TFE (0.05 M), blue LEDs (λ_{max} = 456 nm), room temperature, N_2, 20 h, GC-FID yields of the crude reaction mixture using 1,3,5-trimethoxybenzene as internal standard. [b] Yields were calculated based on **1a**.

Supplementary Table 11. Influence of the AcOH dosage[a]

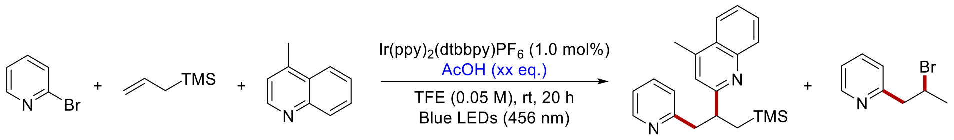

Entry	AcOH (eq.)	Yield of product (%)	
		**4aa**	**5**[b]
1	0.2	88	0
2	0.4	91	0
3	0.6	92	0
4	0.8	93	0
5	1.0	93	0
6	1.2	92	0
7	1.4	93	0
8	1.6	93	0
9	1.8	93	0
10	2.0	93	0

[a] Reaction conditions: **1a** (0.20 mmol), **2a** (0.5 mmol), **3a** (0.10 mmol), Ir(ppy)$_2$(dtbbpy)PF$_6$ (1.0 mol%), AcOH (xx mmol), TFE (0.05 M), blue LEDs (λ_{max} = 456 nm), room temperature, N_2, 20 h, GC-FID yields of the crude reaction mixture using 1,3,5-trimethoxybenzene as internal standard. [b] Yields were calculated based on **1a**.

7.9 Control experiments of quinoline additive

According to general procedure, the control experiments were carried out to demonstrate the important role of quinoline **3/3’**. As shown in supplementary Figure 23, under AcOH/TFE standard conditions, **1a** and **2a** didn’t react smoothly to produce halopyridylation product **5** or its elimination product **5’**. Strong acid TFA replacing AcOH contributed to 68% yield of **5** and 6% yield of **5’**. These results shown that AcOH/TFE conditions are not acidic enough to activate 2-bromopyridine **1a** interacting with PC. However, when 2,4-dimethylquinoline (1.0 equiv.) was added to AcOH/TFE standard conditions, the model reaction could produce **5** and **5’** in 45% and 15% yields respectively.

As for multicomponent reactions, 2,4-dimethylpyridine **3n** just delivered 15% yield of 1,2-dihetroarylation product **4an** under AcOH/TFE conditions, while the addition of 2,4-dimethylquinoline (1.0 equiv.) could double the yield of **4an** (33 %) accompanied with 17% yield of **5** and **5’**. Therefore, the control experiments above strongly demonstrated that quinoline **3/3’** could promote the generation of pyridyl radical from halopyridine **1**.

a

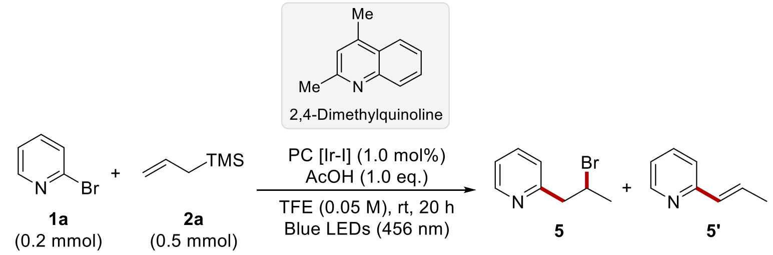

Entry	Variation	Yield of **5** (%)	Yield of **5'** (%)
1	None	Trace	Trace
2	TFA instead of AcOH	68	6
3	+ 2,4-Dimethylquinoline (1.0 eq.)	45	15

b

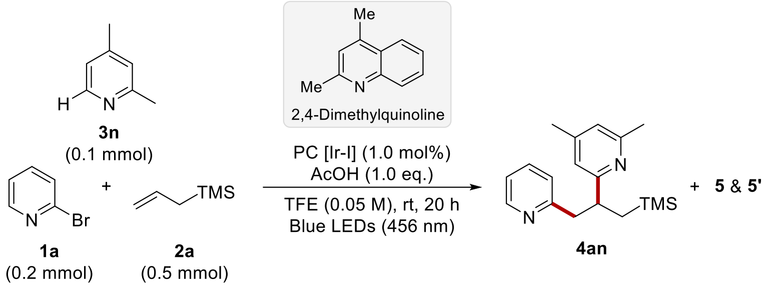

Entry	Variation	Yield of **4an** (%)	Yield of **5+5'** (%)
1	None	15	Trace
2	TFA instead of AcOH	Trace	8
3	+ 2,4-Dimethylquinoline (1.0 eq.)	33	17

Supplementary Figure 23. Control experiments of quinoline additive. **a**, Effect of adding 2,4-dimethylquinoline on photocatalytic bromopyridylation of alkene. **b**, Effect of adding 2,4-dimethylquinoline on photocatalytic 1,2-diheteroarylation of alkene.

7.10 Plausible mechanism for the generation of side-products 5 and 6

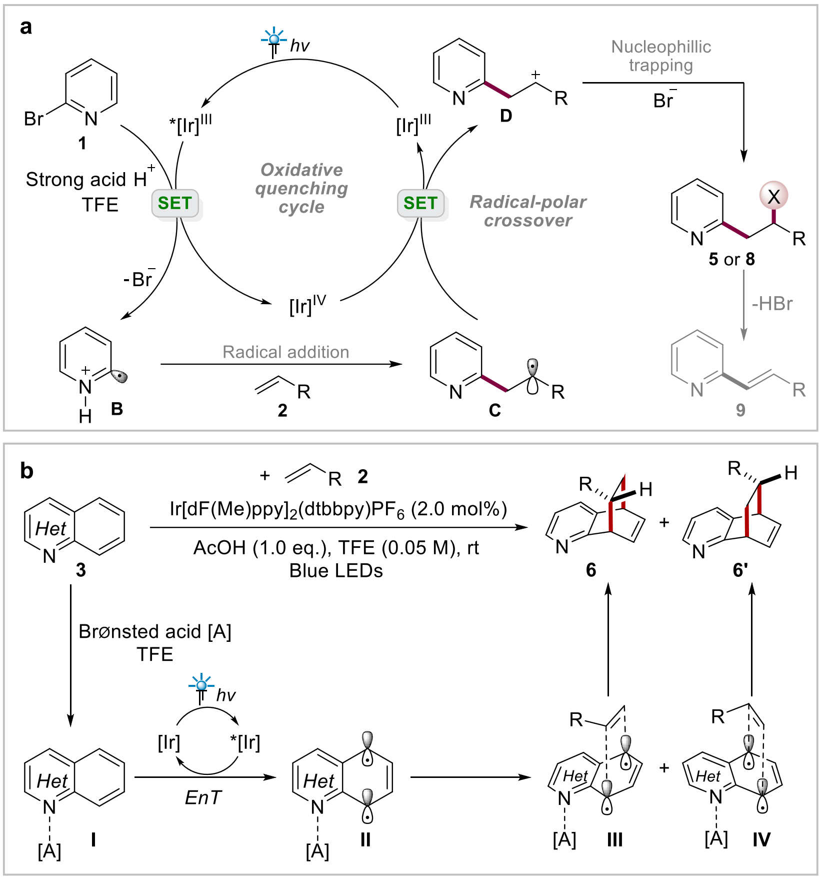

Supplementary Figure 24. The proposed mechanism for the generation of side-products **5** and **6**. **a**, Photo-induced catalytic halopyridylation of alkenes. **b**, Photochemical intermolecular dearomative cycloaddition of bicyclic azaarenes with alkenes.

Comments on plausible mechanism of the generation of **5** and **6**:

Based on the mechanistic studies of our previous work, the generation of side-products **5** or **8** is following the mechanism of acid-promoted domino photocatalytic oxidative quenching activation of halopyridines and radical-polar crossover pathway. And when high triple-state energy photocatalyst is employed, photo-induced EnT for intermolecular dearomative cycloaddition of quinolines with alkenes is occurred to produce side-product **6**.[3]

7.11 Determination of the excited-state oxidation potential of photocatalyst Ir-I in TFE

Cyclic Voltammetry (CV) analysis was performed on an Electrochemical Analyzer potentiostat model 620E from CH Instruments at a sweep rate of 0.1 V/s. Samples were prepared as TFE solution, which were degassed by sparging with solvent-saturated N_2 gas for 5 minutes prior to measurements. The measurement was performed using 0.1 M solution of tetra-*n*-butylammonium hexafluorophosphate. A saturated calomel electrode (SCE) was used as the reference electrode while a glassy carbon electrode and a platinum wire were used as the working electrode and counter electrode, respectively. The ground-state oxidation potential of Ir-I in TFE was determined using the cyclic voltammetry, affording $E_{ox} = 0.98$ V vs. SCE.

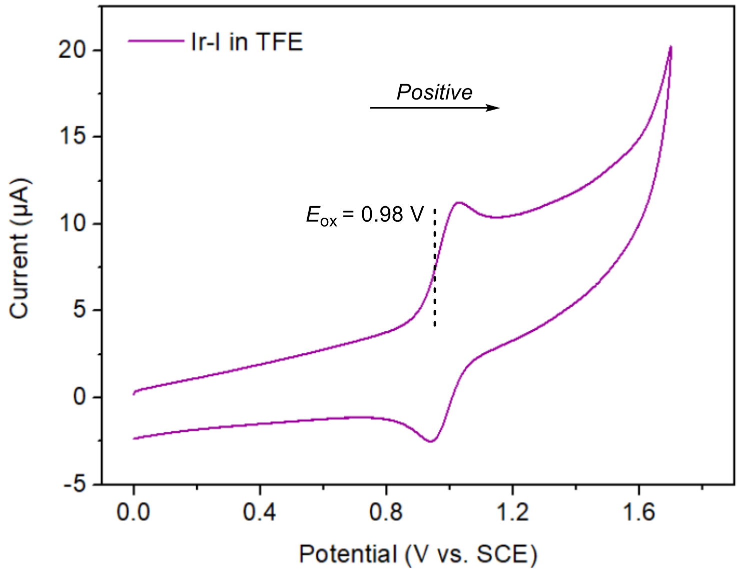

Supplementary Figure 25. CV spectra obtained for $Ir(ppy)_2(dtbbpy)PF_6$ in TFE solvent.

UV-vis absorption spectra were recorded at room temperature on a Shimadzu UV-2600 double beam spectrophotometer with a 1 cm quartz cuvette. For emission studies, aerated solutions were bubbled with compressed air for 5 minutes and spectra were taken using the cuvette for absorption analysis. These two spectra use the same TFE solution of photocatalyst Ir-I (C = $5*10^{-5}$ M). For steady-state measurements, samples were excited at 390 nm for photocatalyst Ir-I. UV-vis absorption and steady-state emission spectra of Ir-I in TFE were studied to determine the optical band gap. And $E_{0,0}$ can be calculated using the intersection point between the normalized absorption and emission spectra as $E_{0,0} = 2.52$ eV.

Therefore, the redox potential of the excited state *Ir-I in TFE is $^*E_{ox} = -1.54$ V ($^*E_{ox} = E_{ox} - E_{0,0} = 0.98 - 2.52 = -1.54$ V).

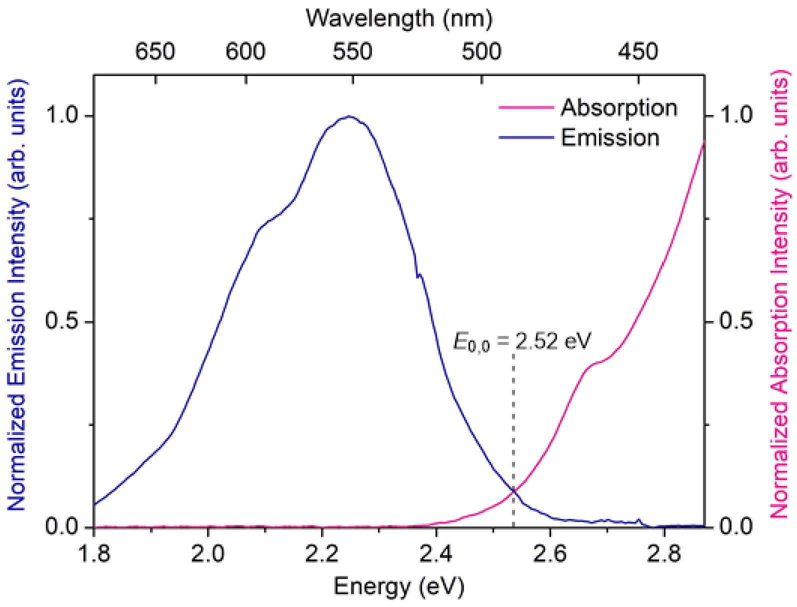

Supplementary Figure 26. Optical gap determination for Ir(ppy)$_2$(dtbbpy)PF$_6$ in TFE solvent.

7.12 Unactivated alkyl bromides exploration and possible XAT process

To verify the XAT reactivity of quinolines **3**/**3'**, the reactions between quinoline and unactivated alkyl bromides (E_{red} < 2 V vs. SCE) were carried out under the standard conditions. However, as shown in Supplementary Figure 27, primary and secondary alkyl bromides could react smoothly with quinoline, affording Minisci-type products in decent yields. It is worth noting that there are no common halogen abstracting reagents existing in these conditions, such as organic tin, silicon and amine. And photocatalyst Ir-I is not reductive enough to activate bromoalkenes.

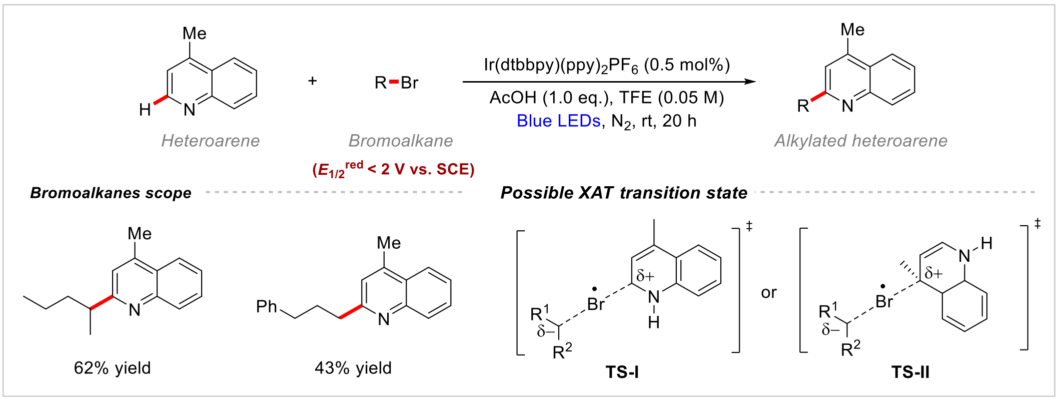

Supplementary Figure 27. Unactivated alkyl halides exploration and possible XAT process.

The radical intermediate **3'** is strongly nucleophilic like the α-aminoalkyl radical. And the α-aminoalkyl radical is able to promote the homolytic activation of carbon-halogen bonds via halogen-atom transfer (XAT). In this case, intermediate **3'** might serve as halogen abstracting reagent to undergo XAT with bromopyridine **1**, producing the pyridyl radical **Int-I**. This XAT benefits from related kinetic

polar effects and manifest the similar reactivity to α-aminoalkyl radical. Probably, an irreversible dissociation of the resulting bromoamine **3''** into the aromatized quinolinium bromide **3** provides the driving force to the process. Therefore, the results above suggest the possibility of XAT process interacting between quinoline **3'** and halides, affording carbon radicals.

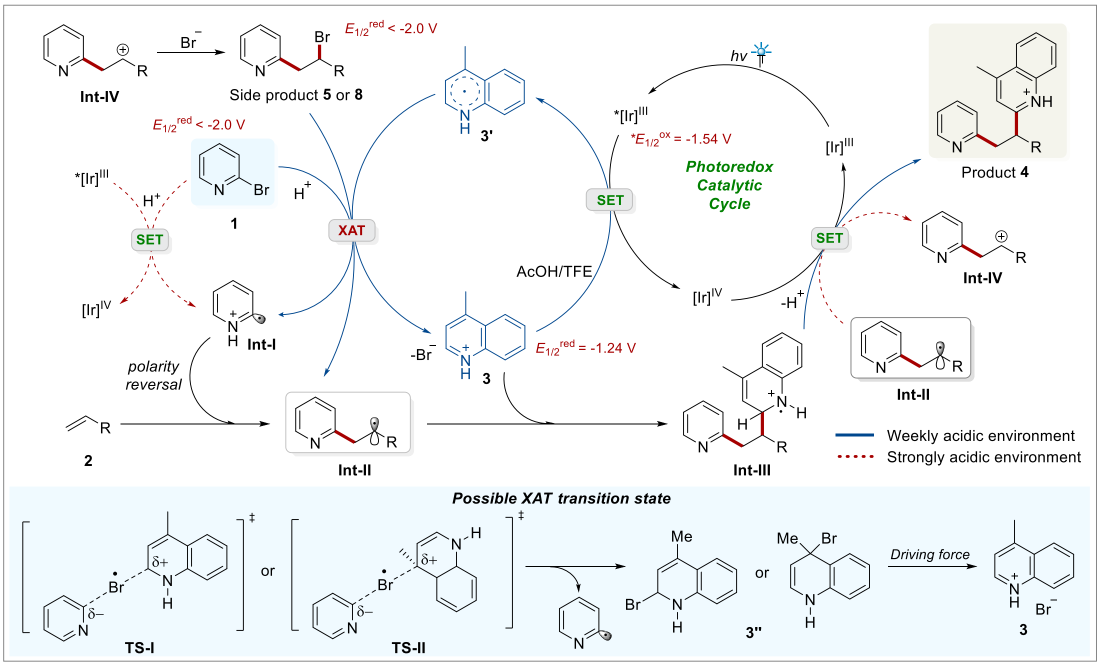

Supplementary Figure 28. The possible mechanism of XAT process.

8. Supplementary Note 6

Copies of NMR spectra of products:

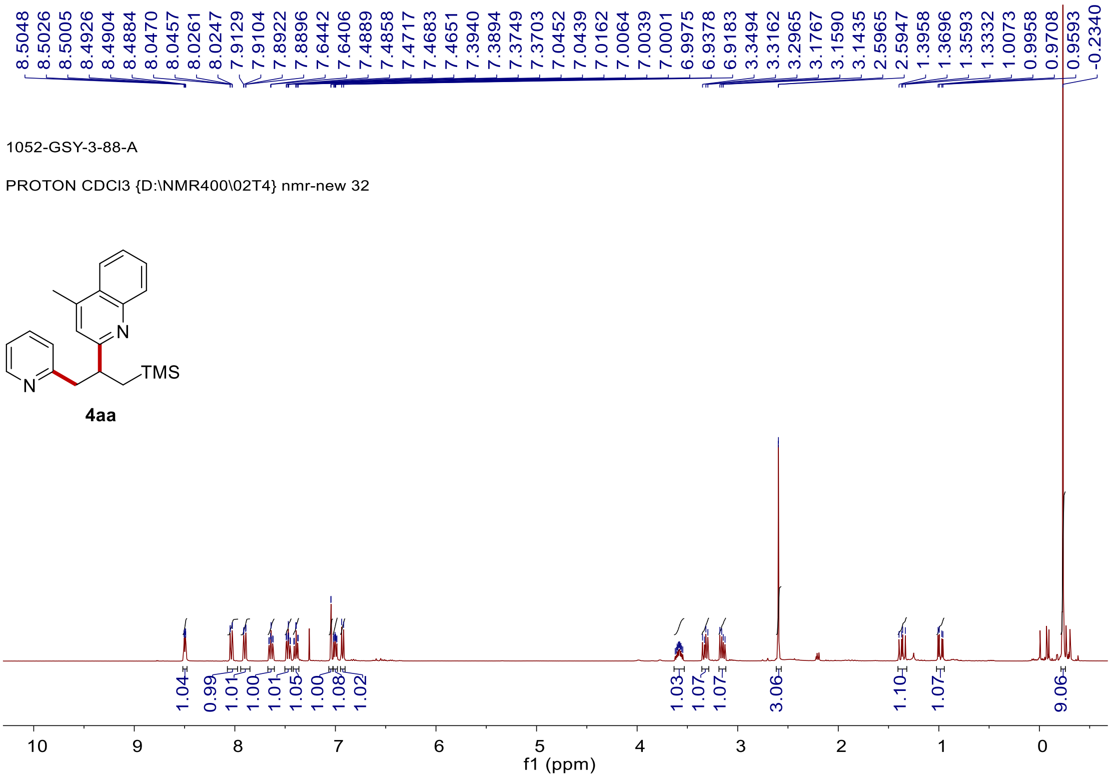

Supplementary Figure 29. ^{1}H NMR spectra of compound **4aa**.

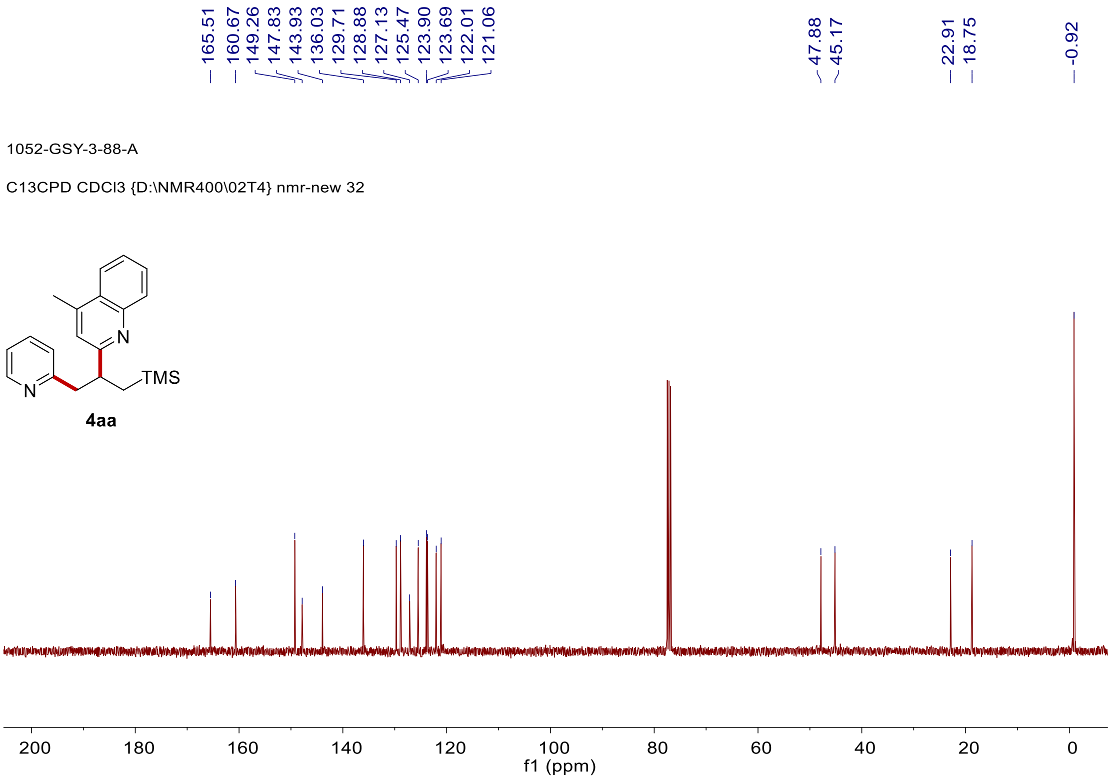

Supplementary Figure 30. ^{13}C NMR spectra of compound **4aa**.

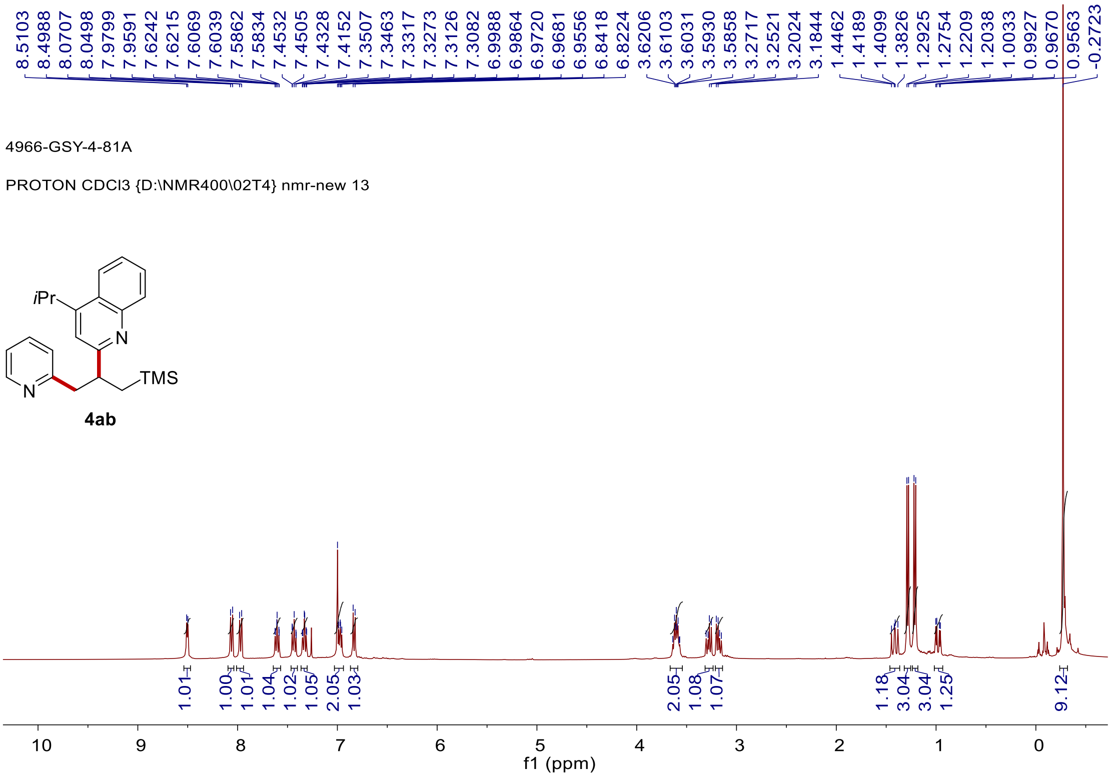

Supplementary Figure 31. ^{1}H NMR spectra of compound **4ab**.

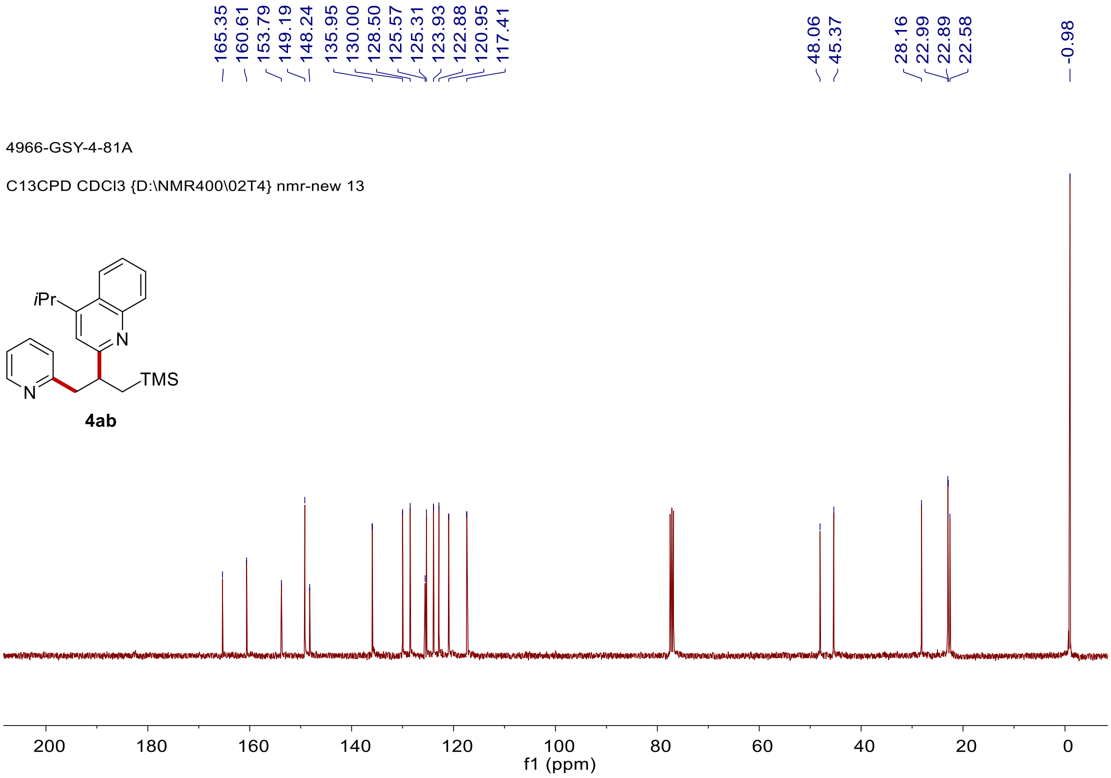

Supplementary Figure 32. ^{13}C NMR spectra of compound **4ab**.

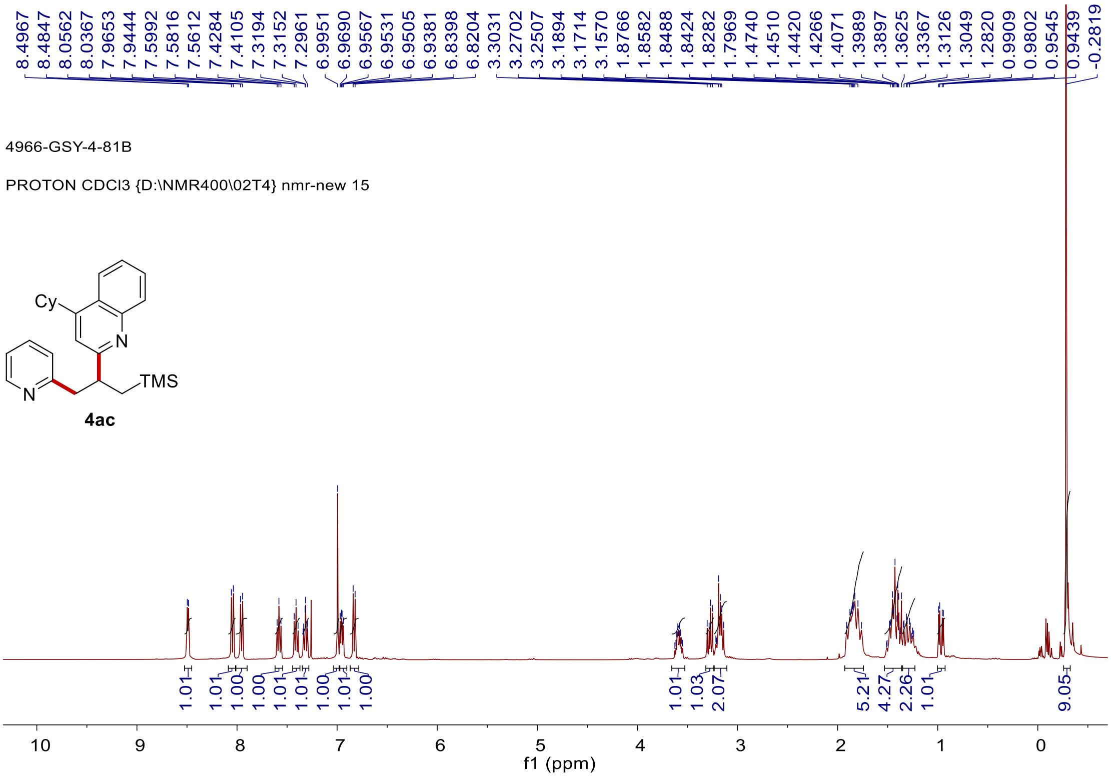

Supplementary Figure 33. ^{1}H NMR spectra of compound **4ac**.

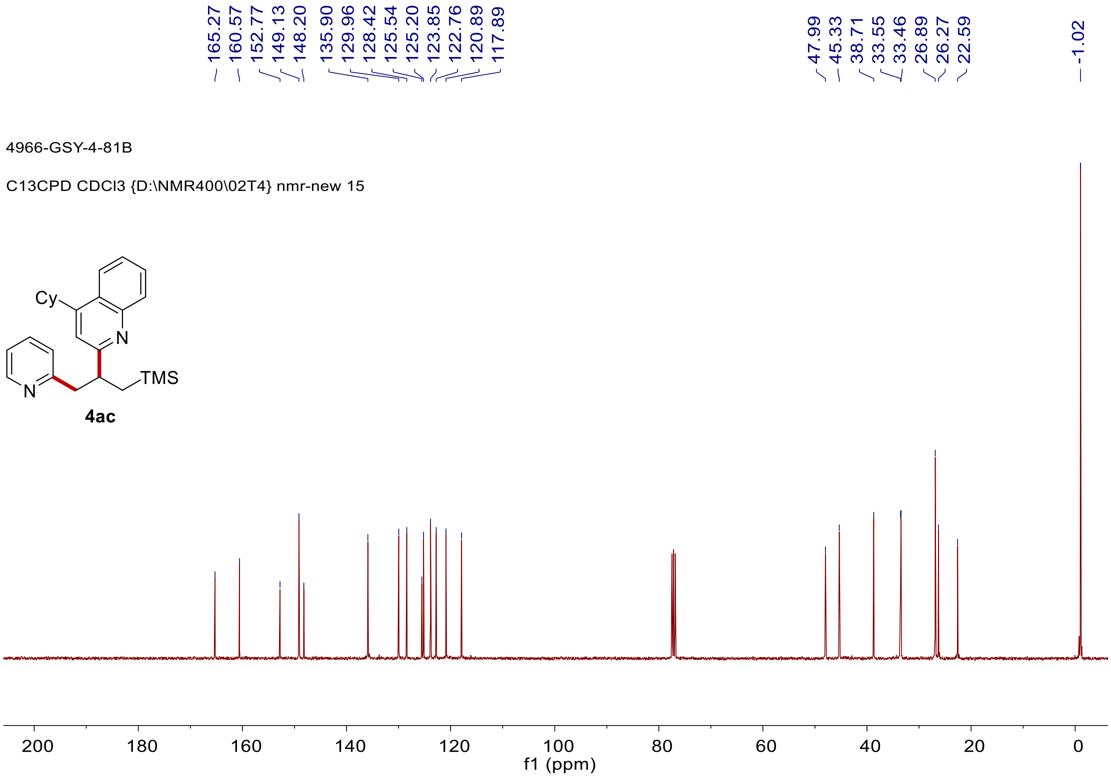

Supplementary Figure 34. ^{13}C NMR spectra of compound **4ac**.

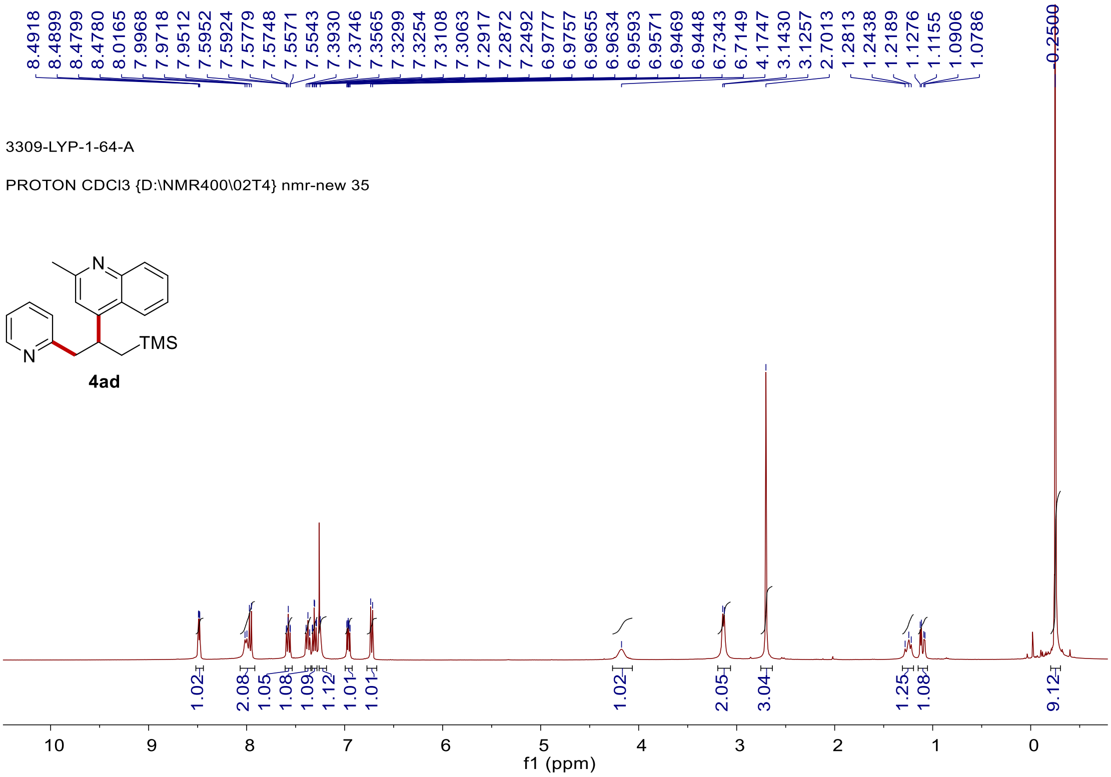

Supplementary Figure 35. ^{1}H NMR spectra of compound **4ad**.

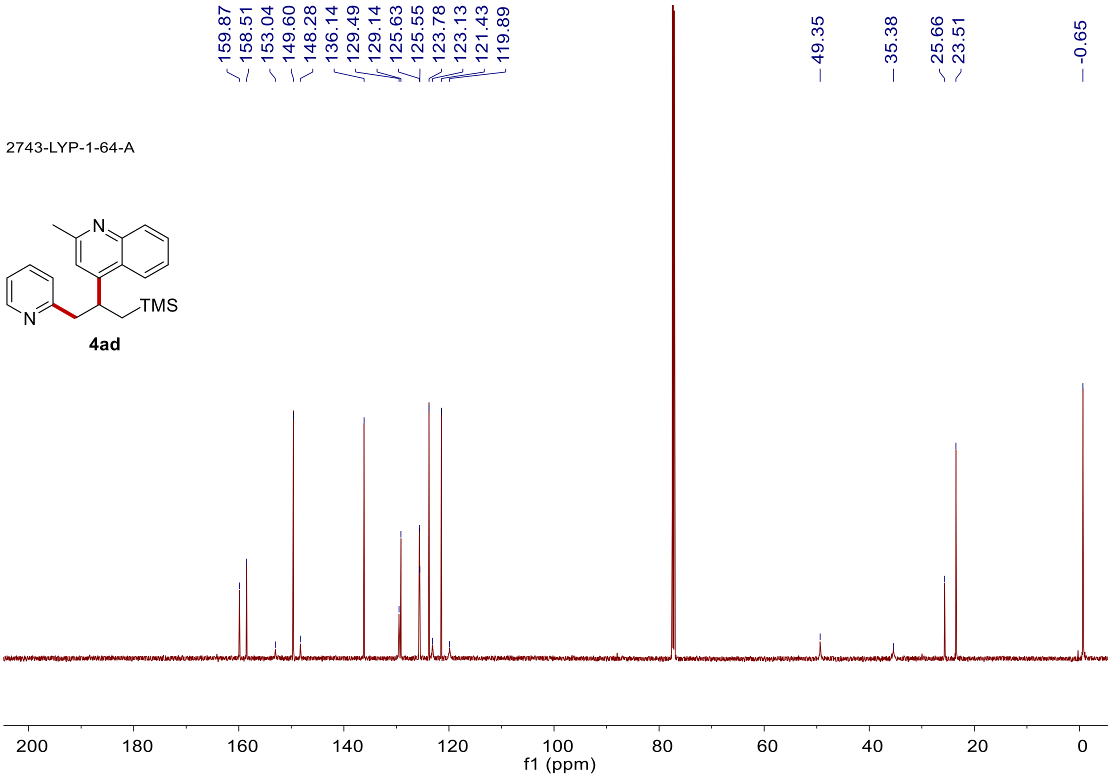

Supplementary Figure 36. ^{13}C NMR spectra of compound **4ad**.

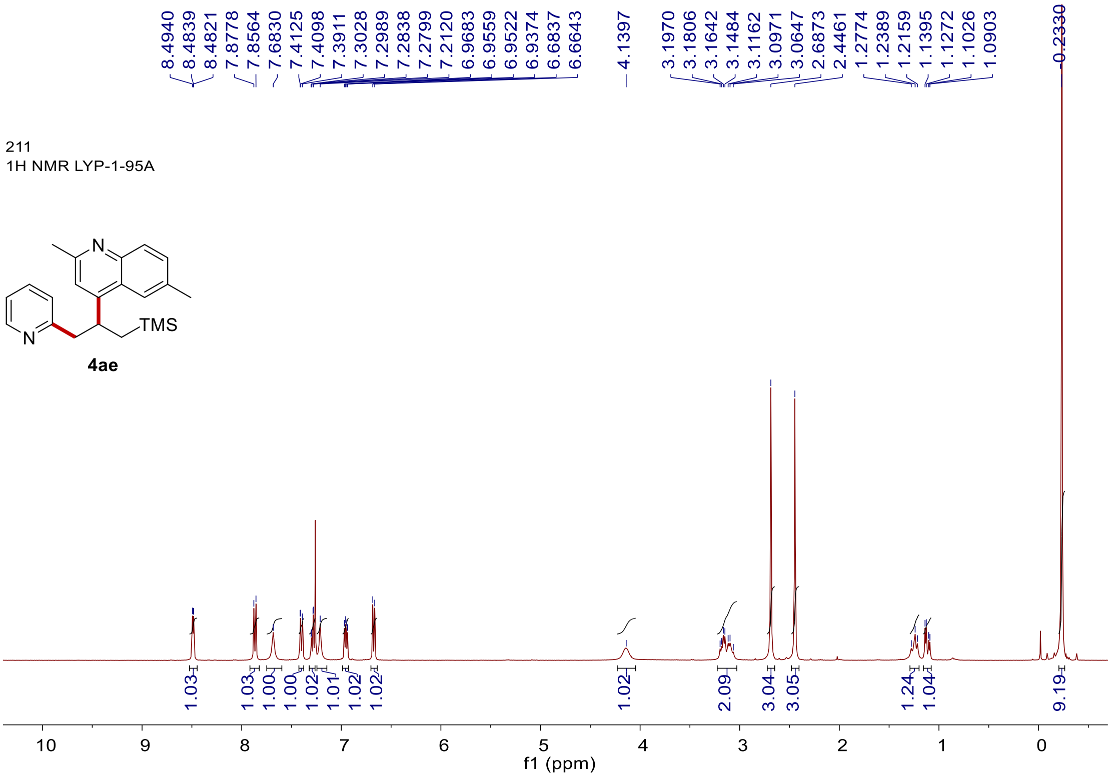

Supplementary Figure 37. ^{1}H NMR spectra of compound **4ae**.

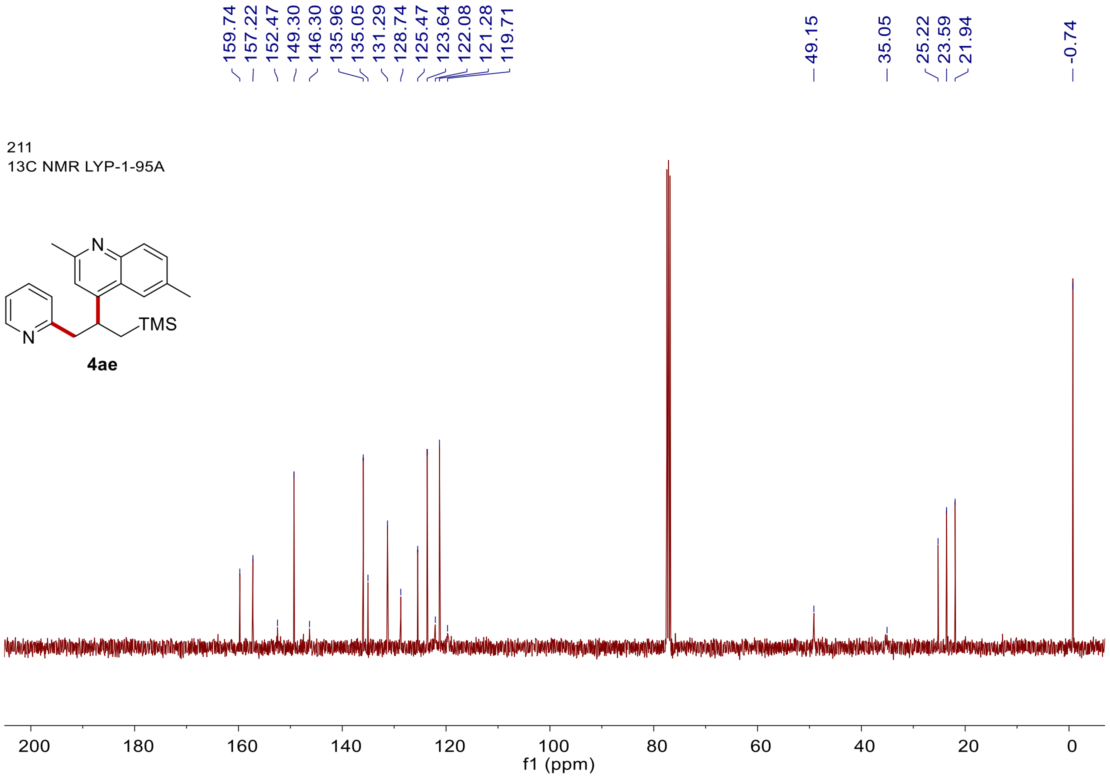

Supplementary Figure 38. ^{13}C NMR spectra of compound **4ae**.

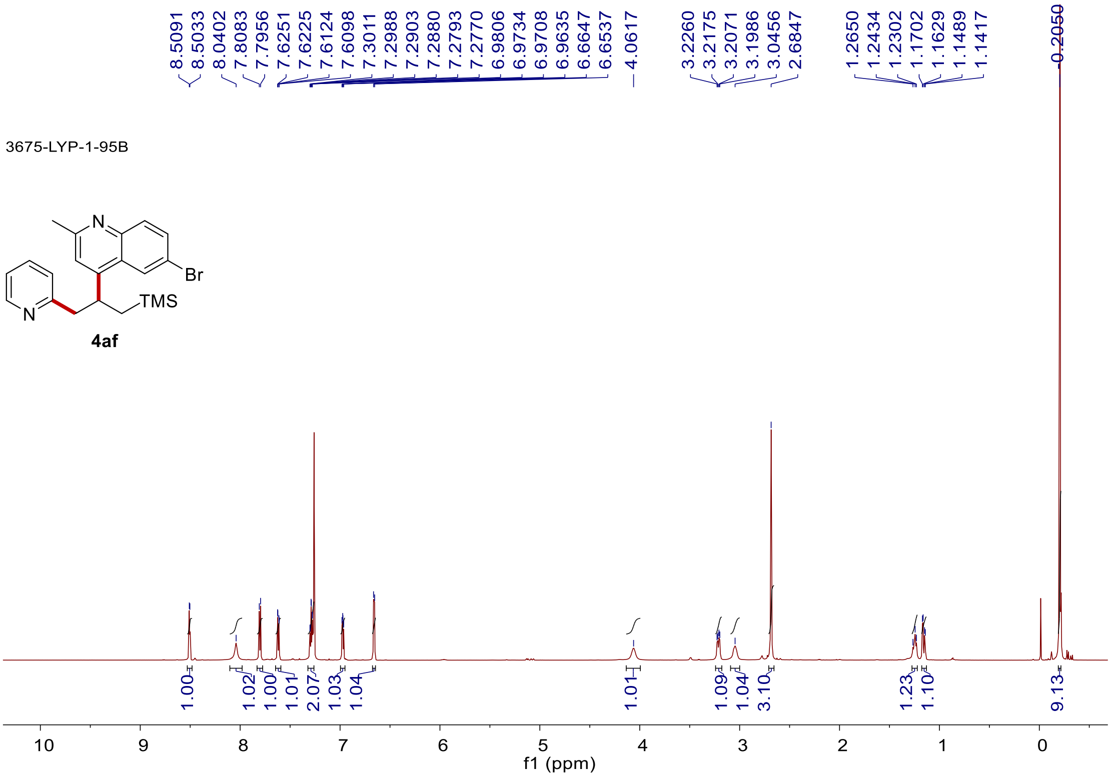

Supplementary Figure 39. ^{1}H NMR spectra of compound **4af**.

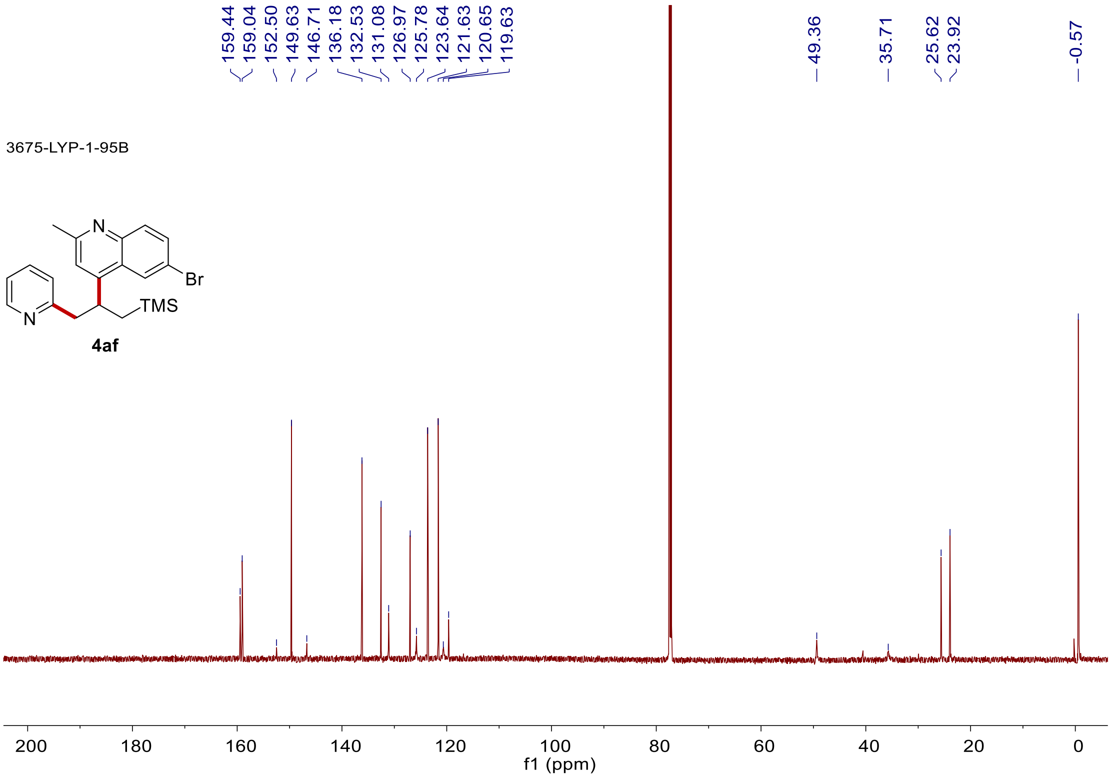

Supplementary Figure 40. ^{13}C NMR spectra of compound **4af**.

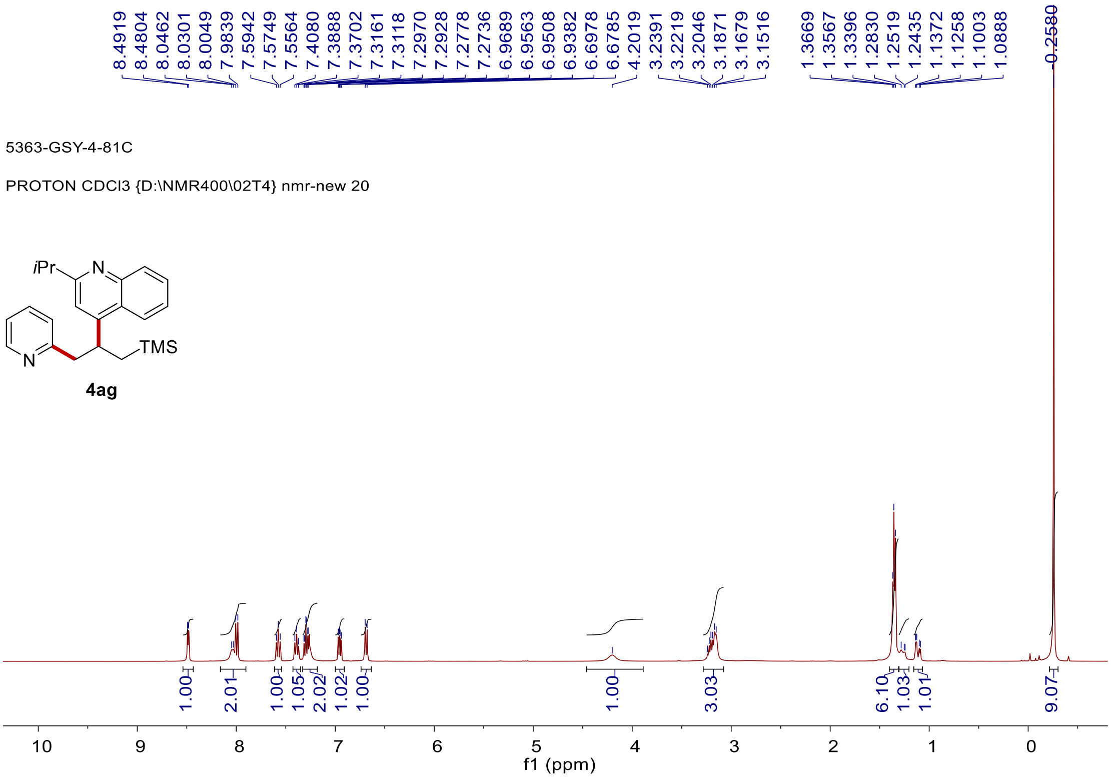

Supplementary Figure 41. ^{1}H NMR spectra of compound **4ag**.

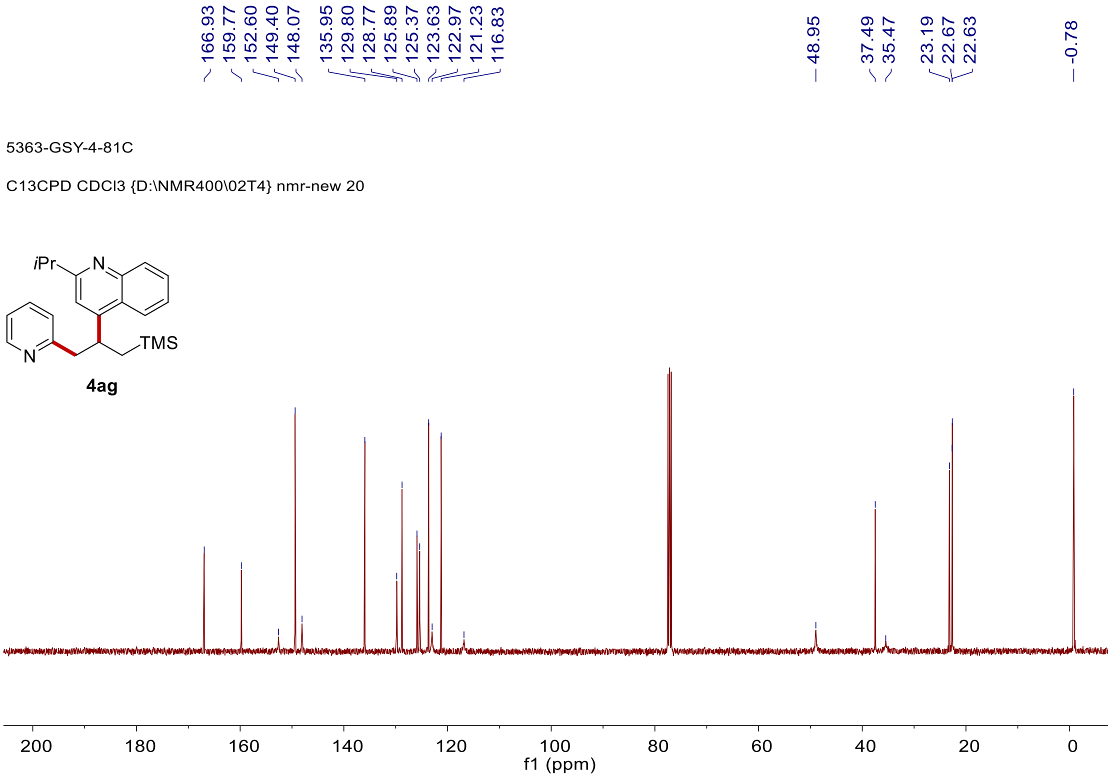

Supplementary Figure 42. ^{13}C NMR spectra of compound **4ag**.

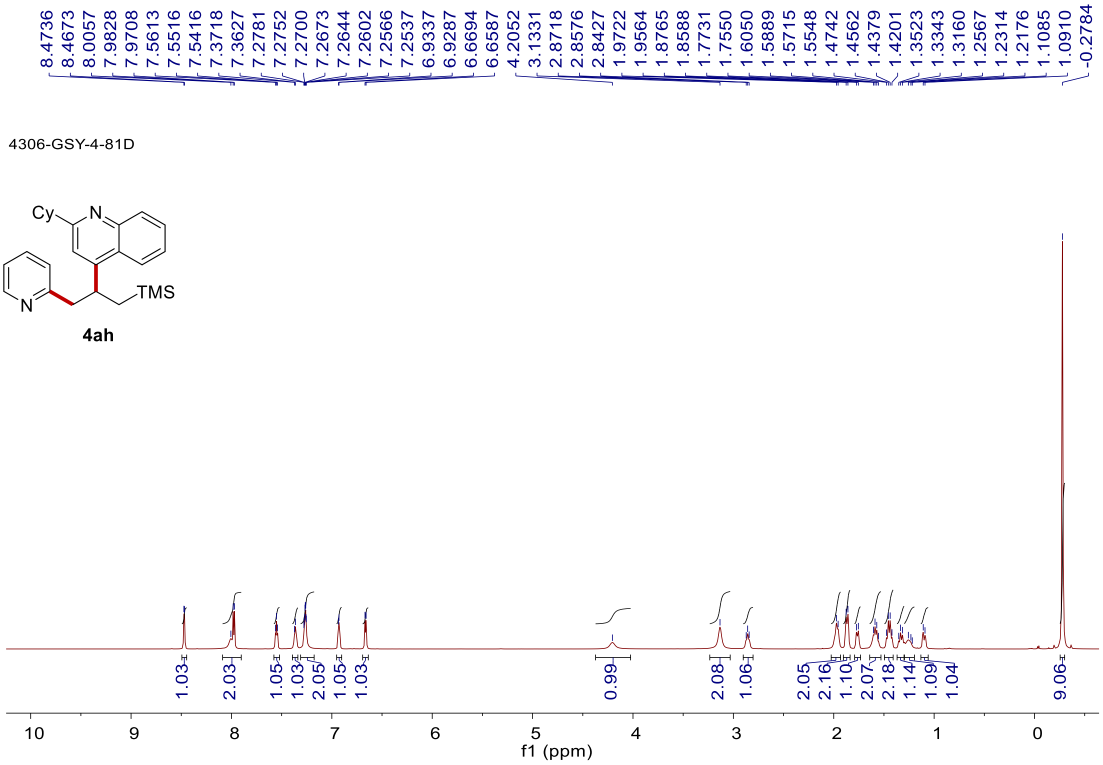

Supplementary Figure 43. ^{1}H NMR spectra of compound **4ah**.

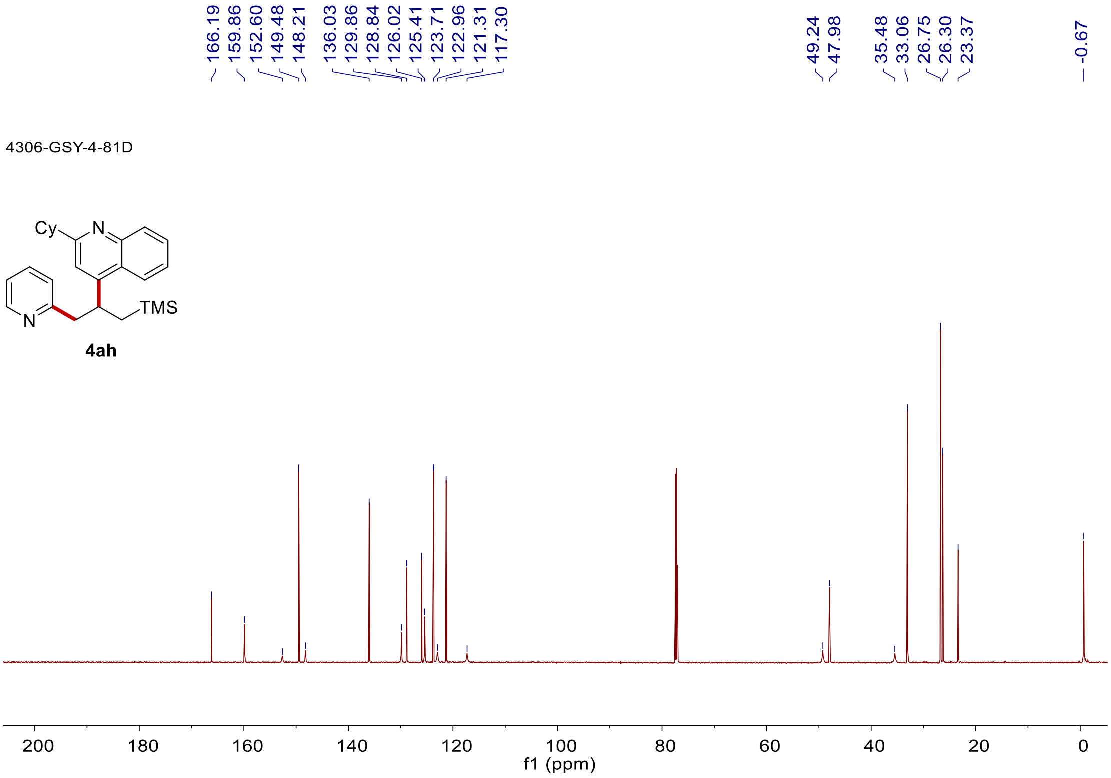

Supplementary Figure 44. ^{13}C NMR spectra of compound **4ah**.

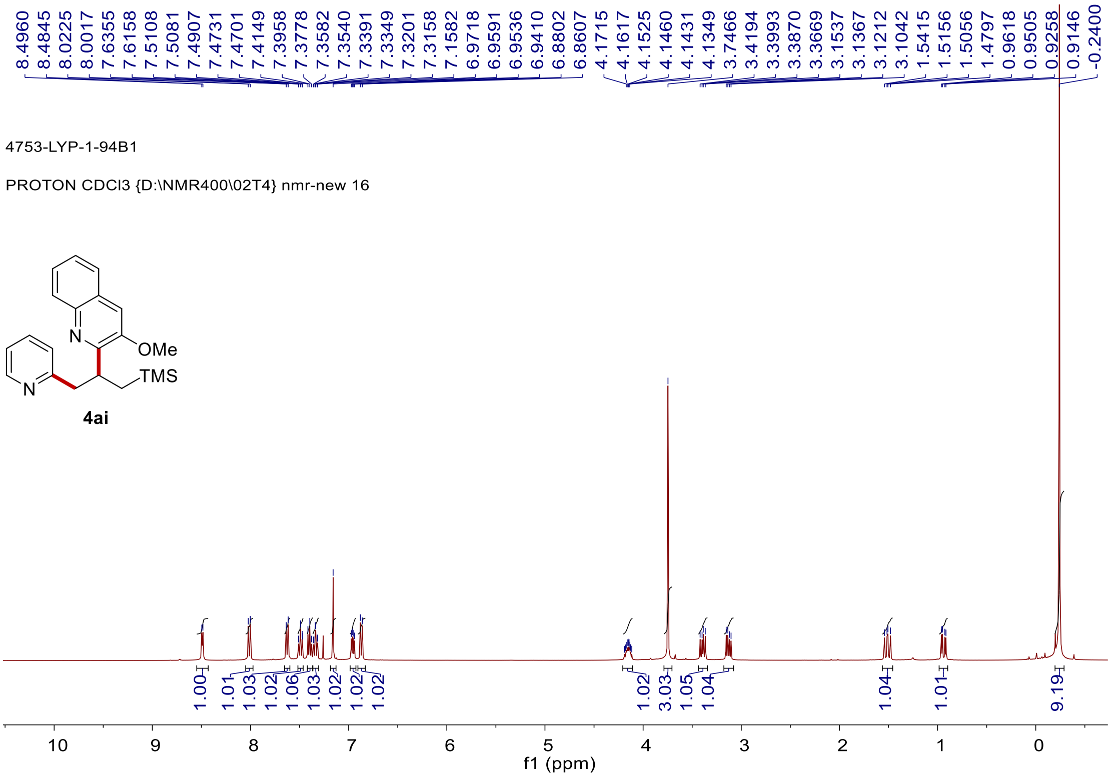

Supplementary Figure 45. ^{1}H NMR spectra of compound **4ai**.

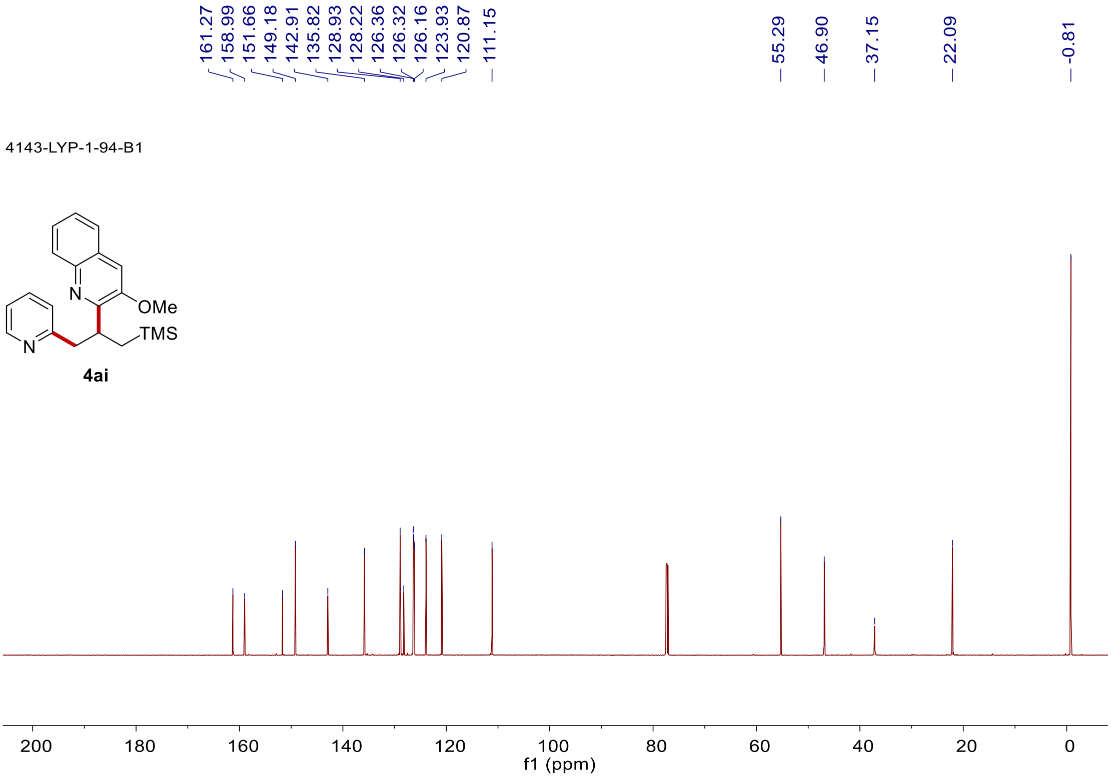

Supplementary Figure 46. ^{13}C NMR spectra of compound **4ai**.

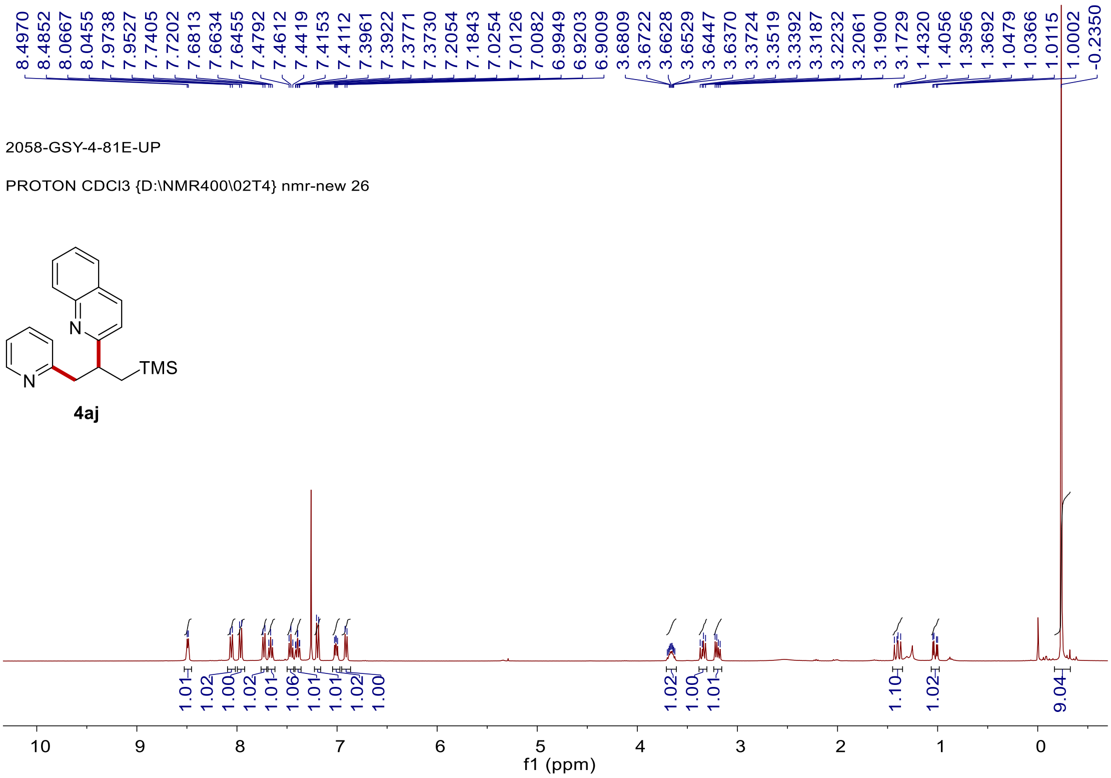

Supplementary Figure 47. ^{1}H NMR spectra of compound **4aj**.

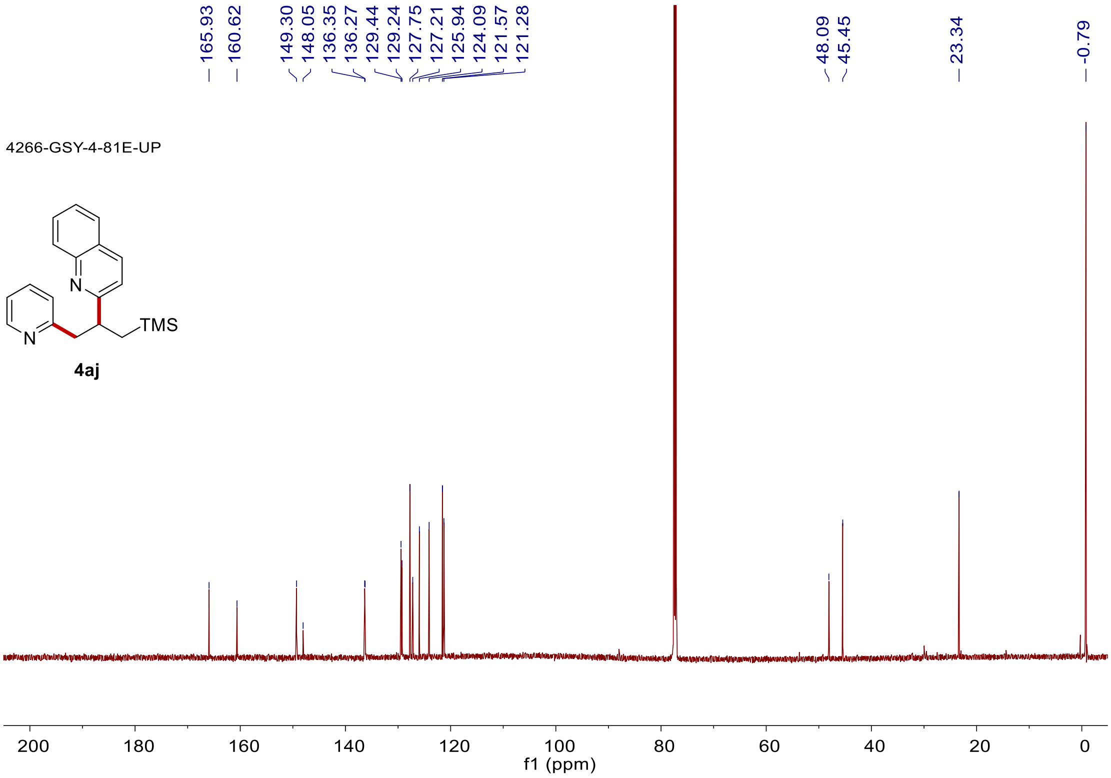

Supplementary Figure 48. ^{13}C NMR spectra of compound **4aj**.

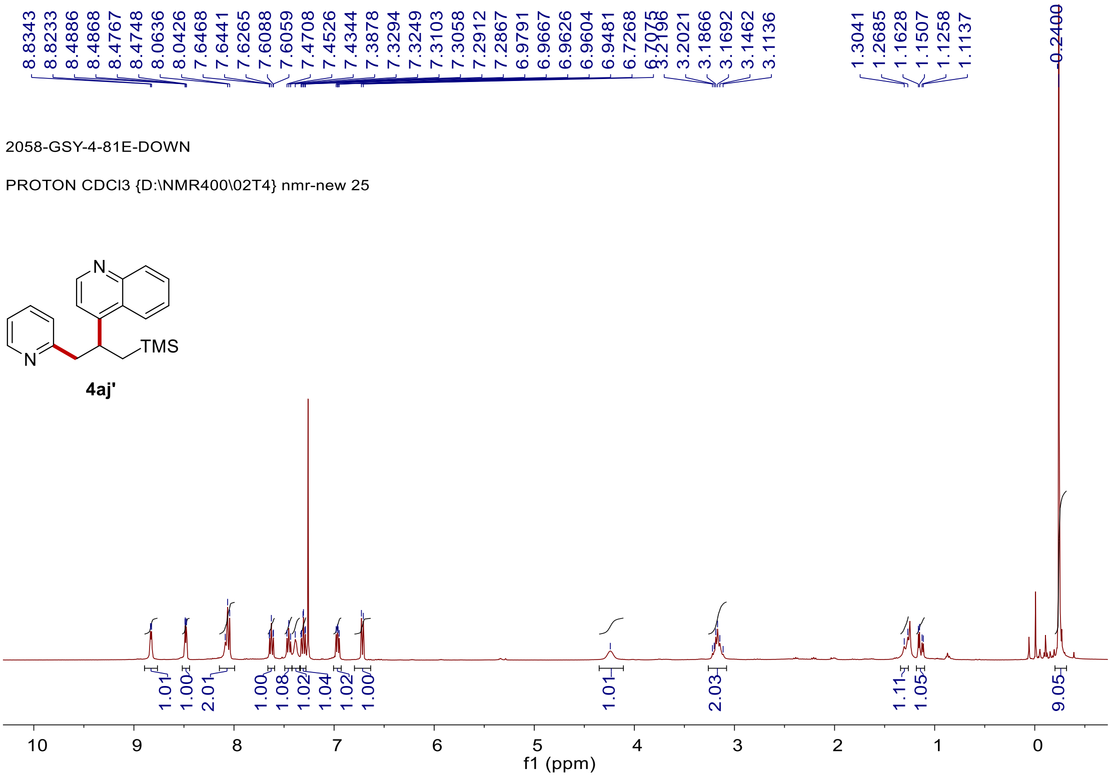

Supplementary Figure 49. ^{1}H NMR spectra of compound **4aj'**.

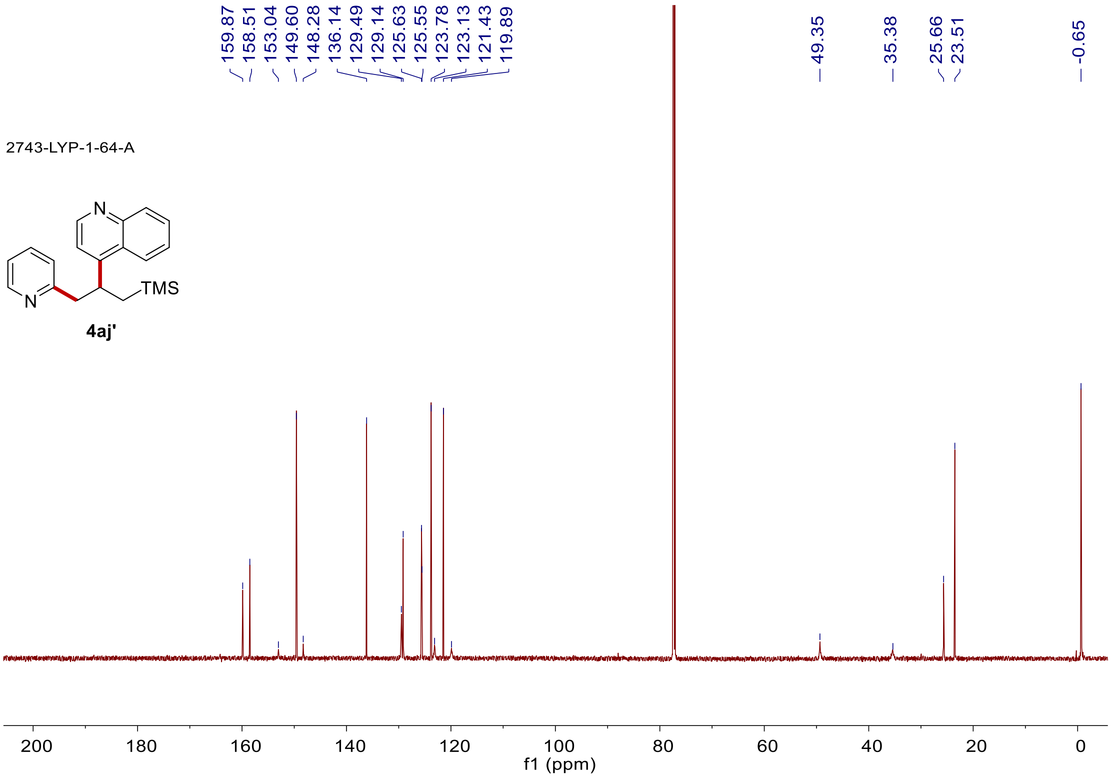

Supplementary Figure 50. ^{13}C NMR spectra of compound **4aj'**.

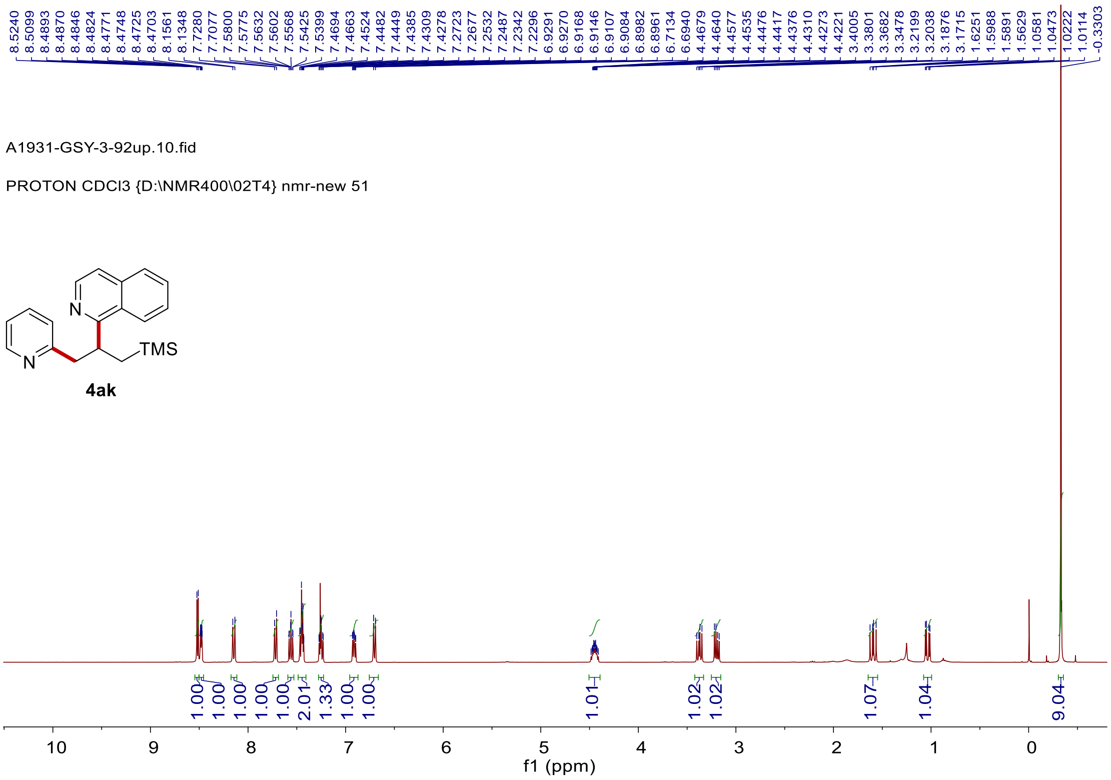

Supplementary Figure 51. ^{1}H NMR spectra of compound **4ak**.

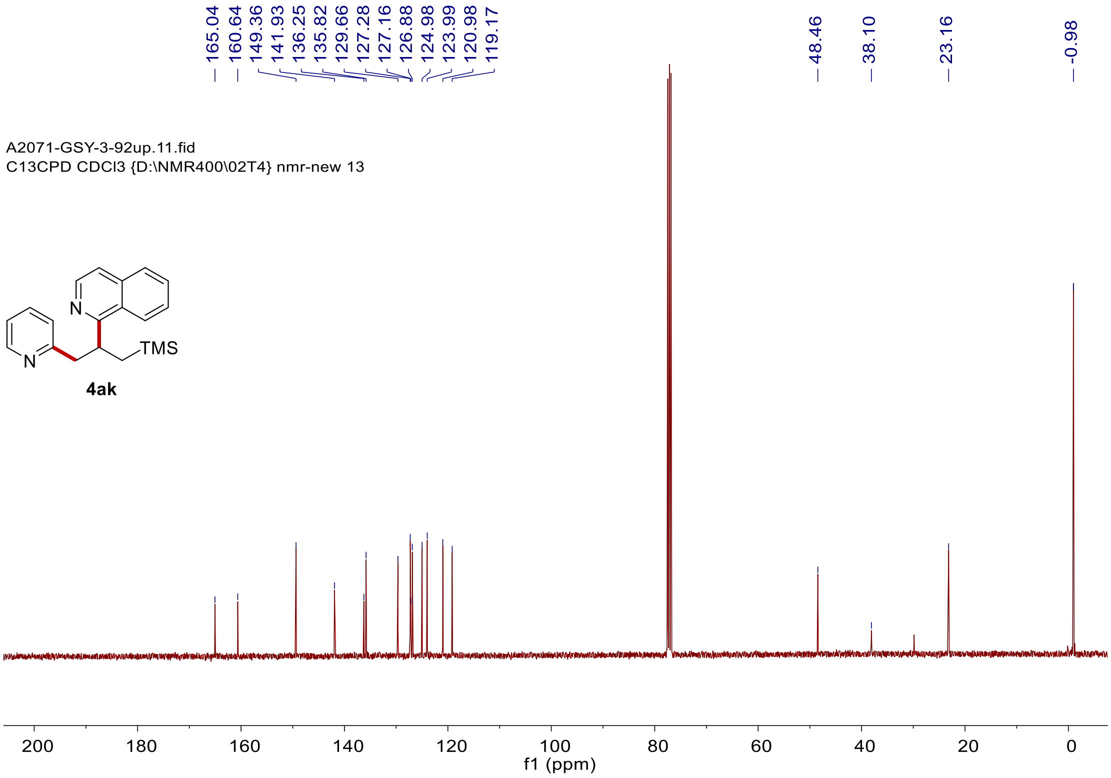

Supplementary Figure 52. ^{13}C NMR spectra of compound **4ak**.

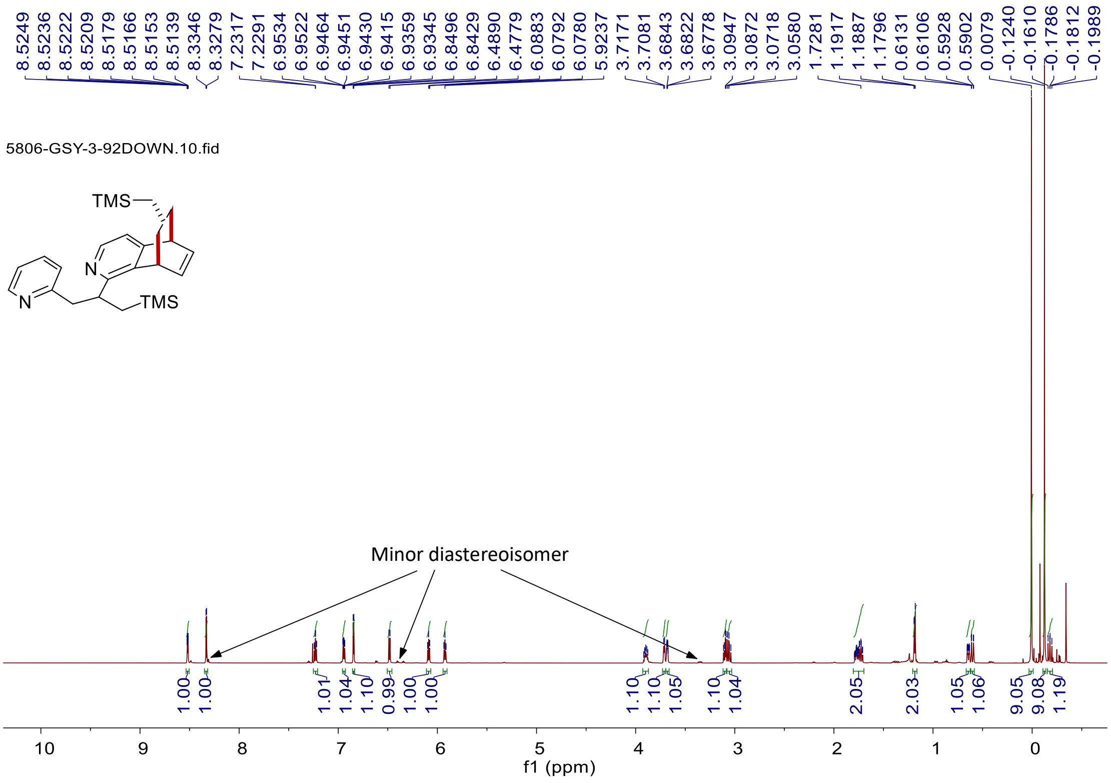

Supplementary Figure 53. ^{1}H NMR spectra of compound **4ak'**.

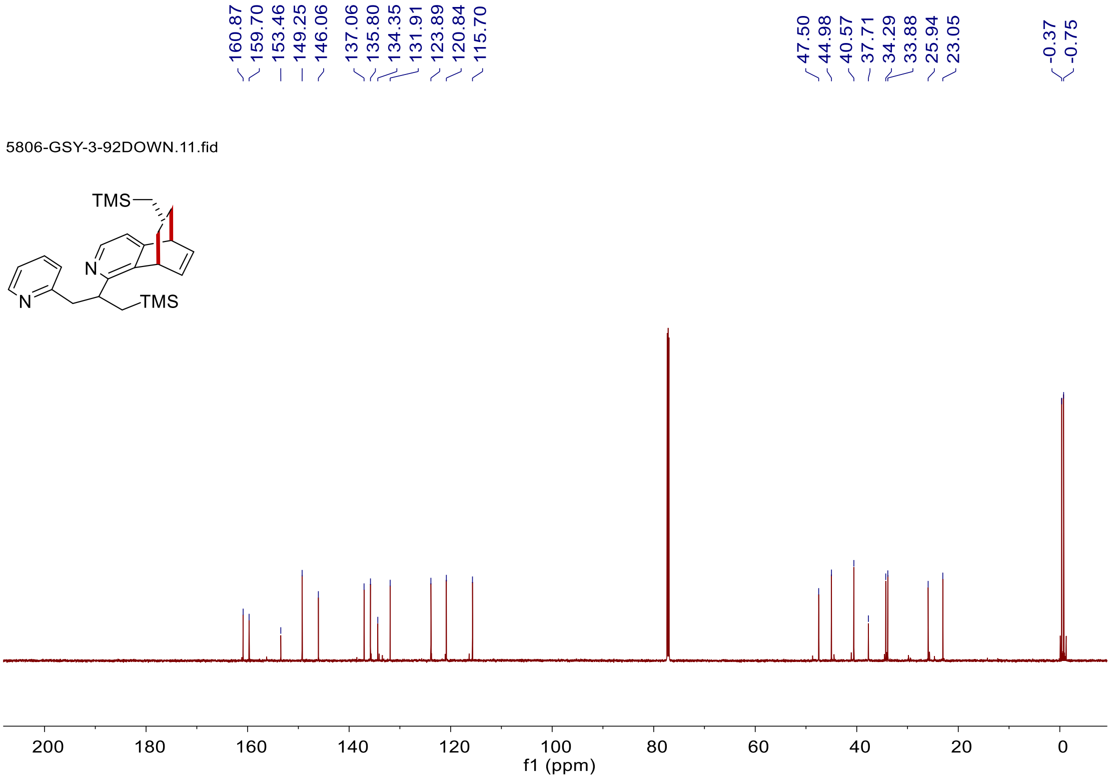

Supplementary Figure 54. ^{13}C NMR spectra of compound **4ak'**.

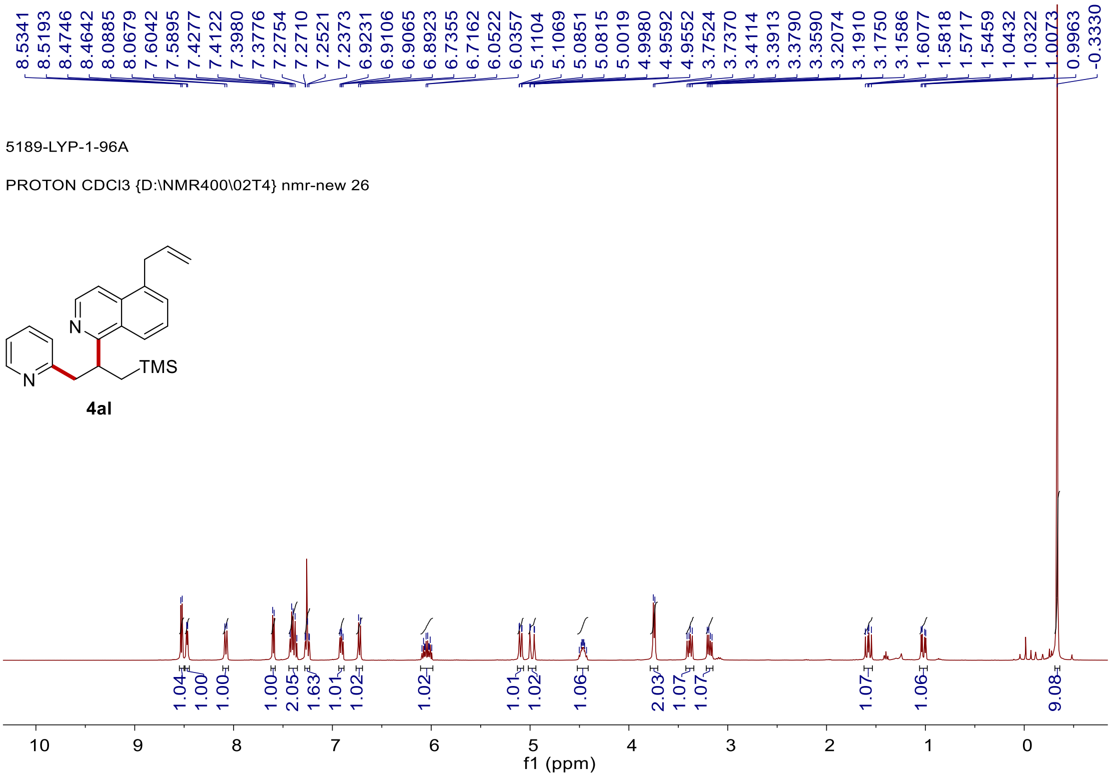

Supplementary Figure 55. ^{1}H NMR spectra of compound **4al**.

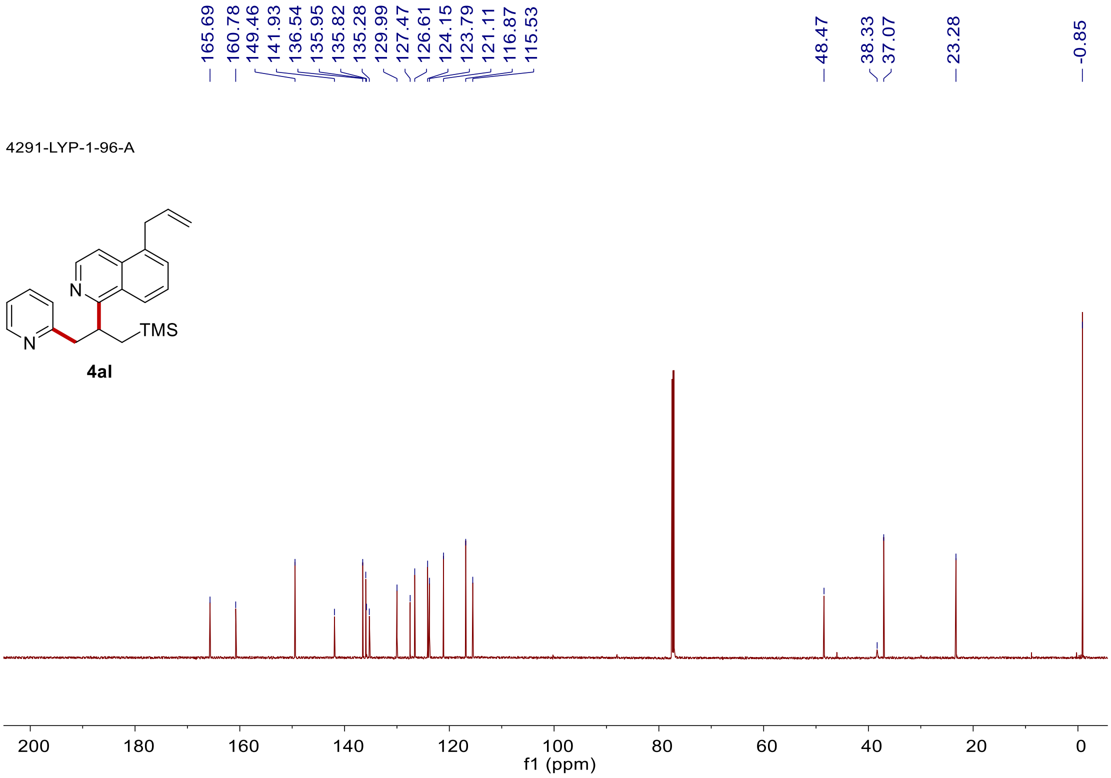

Supplementary Figure 56. ^{13}C NMR spectra of compound **4al**.

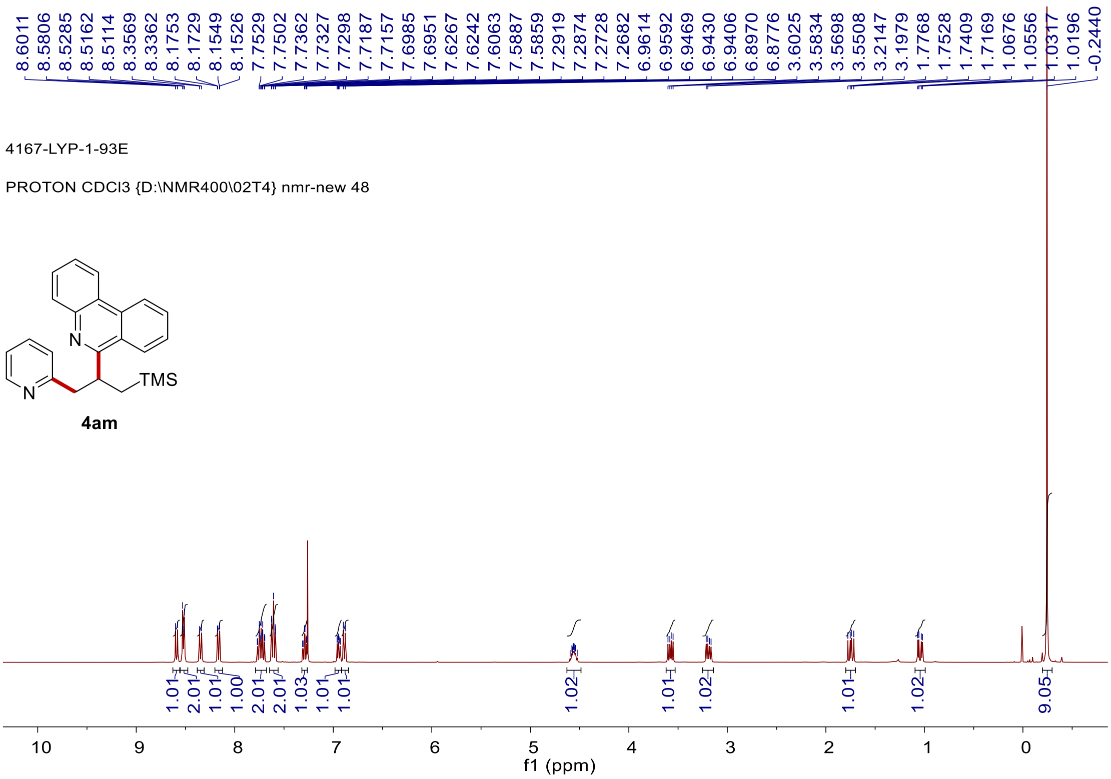

Supplementary Figure 57. ^{1}H NMR spectra of compound **4am**.

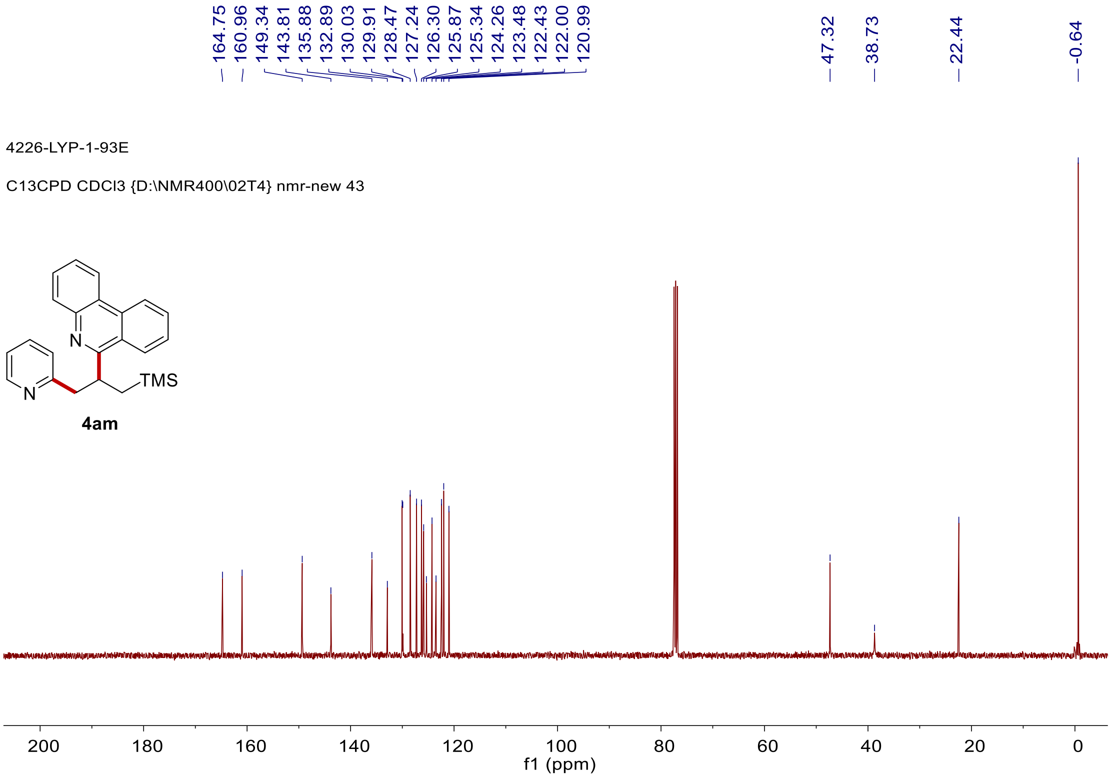

Supplementary Figure 58. ^{13}C NMR spectra of compound **4am**.

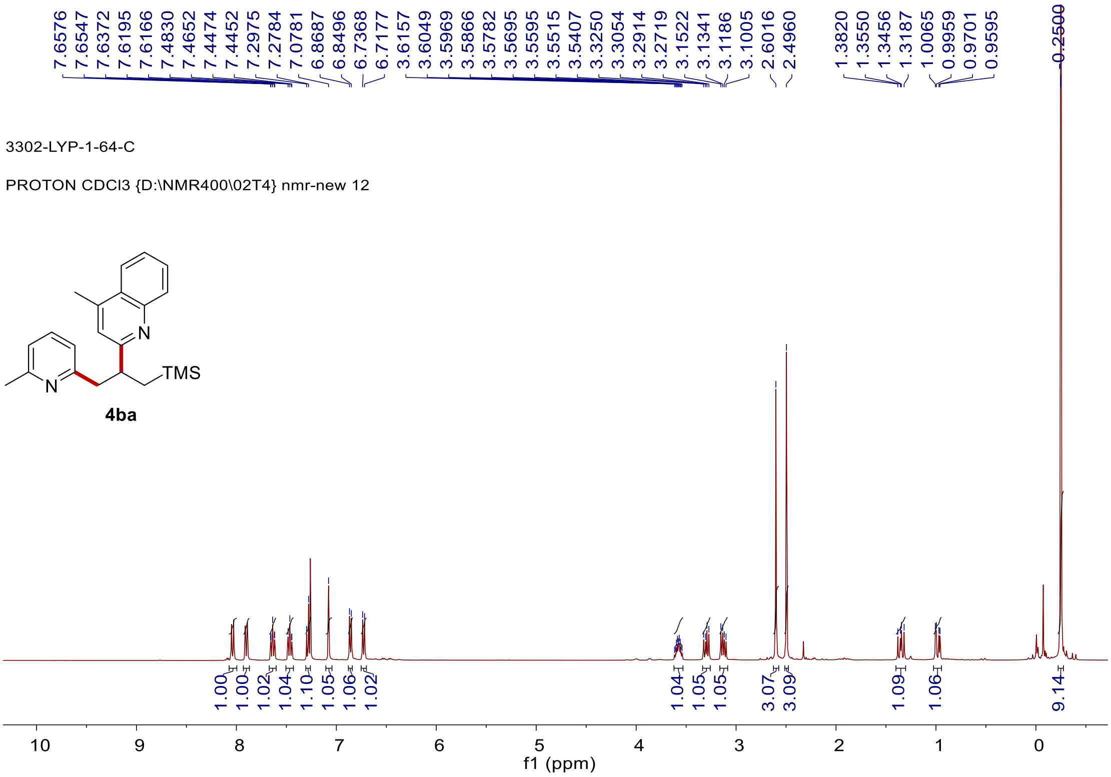

Supplementary Figure 59. ^{1}H NMR spectra of compound **4ba**.

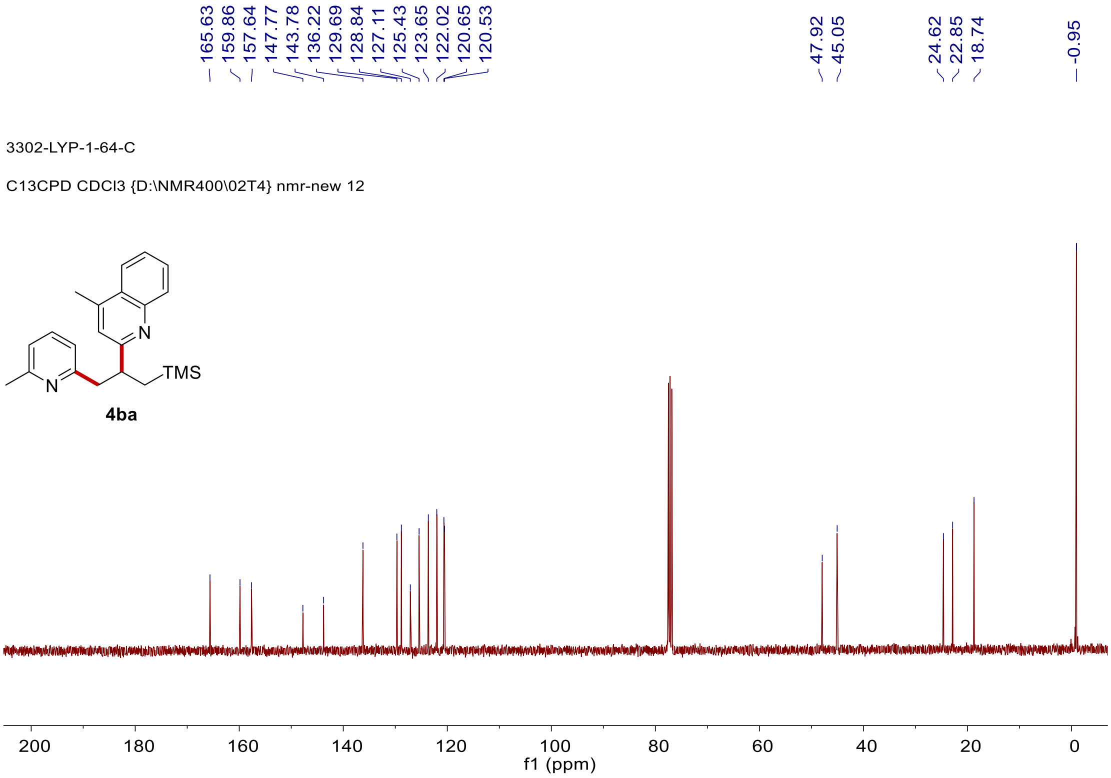

Supplementary Figure 60. ^{13}C NMR spectra of compound **4ba**.

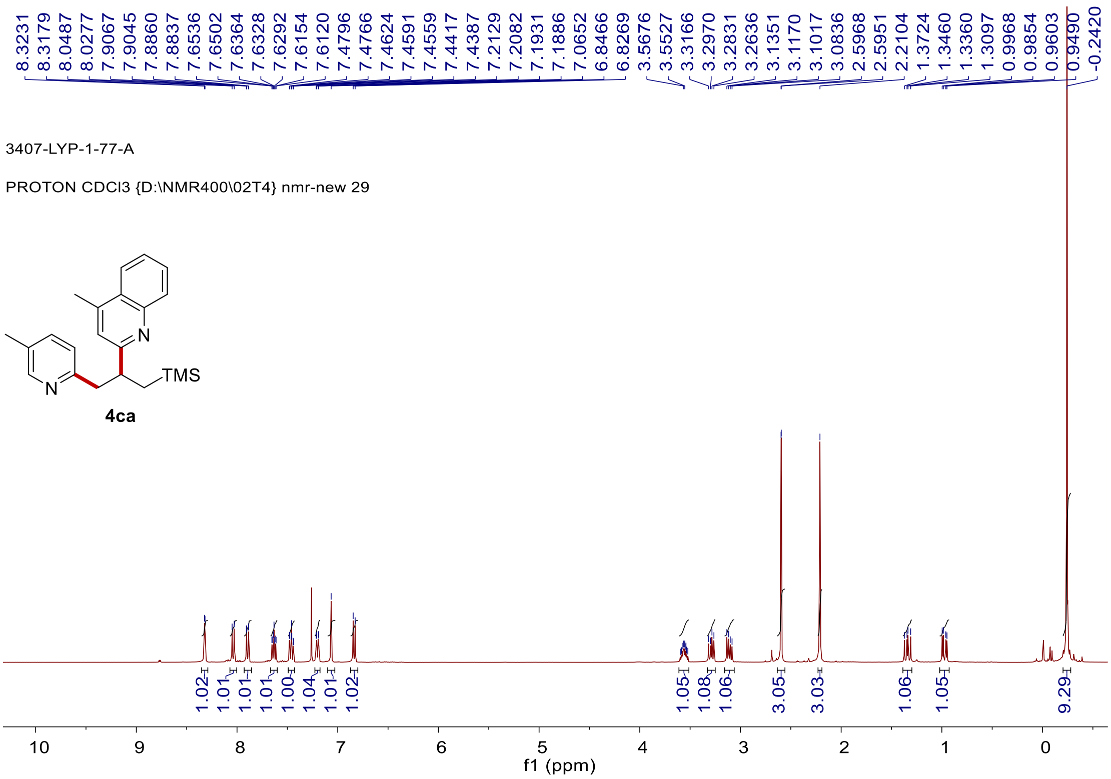

Supplementary Figure 61. ^{1}H NMR spectra of compound **4ca**.

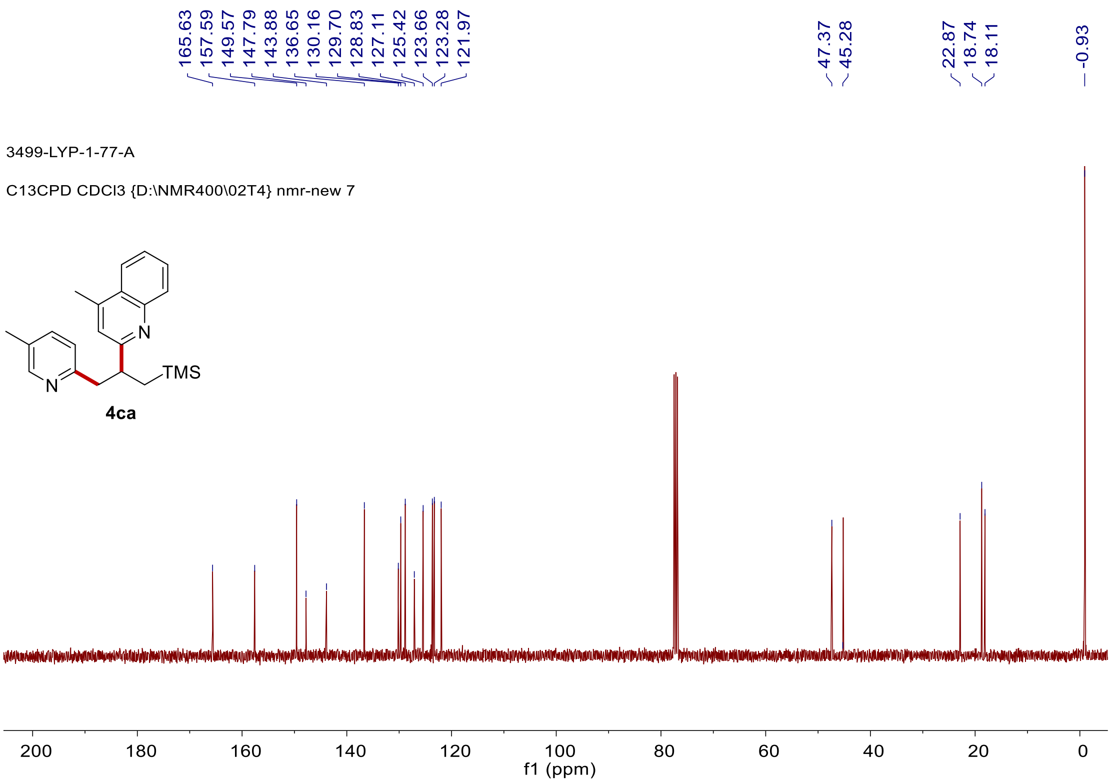

Supplementary Figure 62. ^{13}C NMR spectra of compound **4ca**.

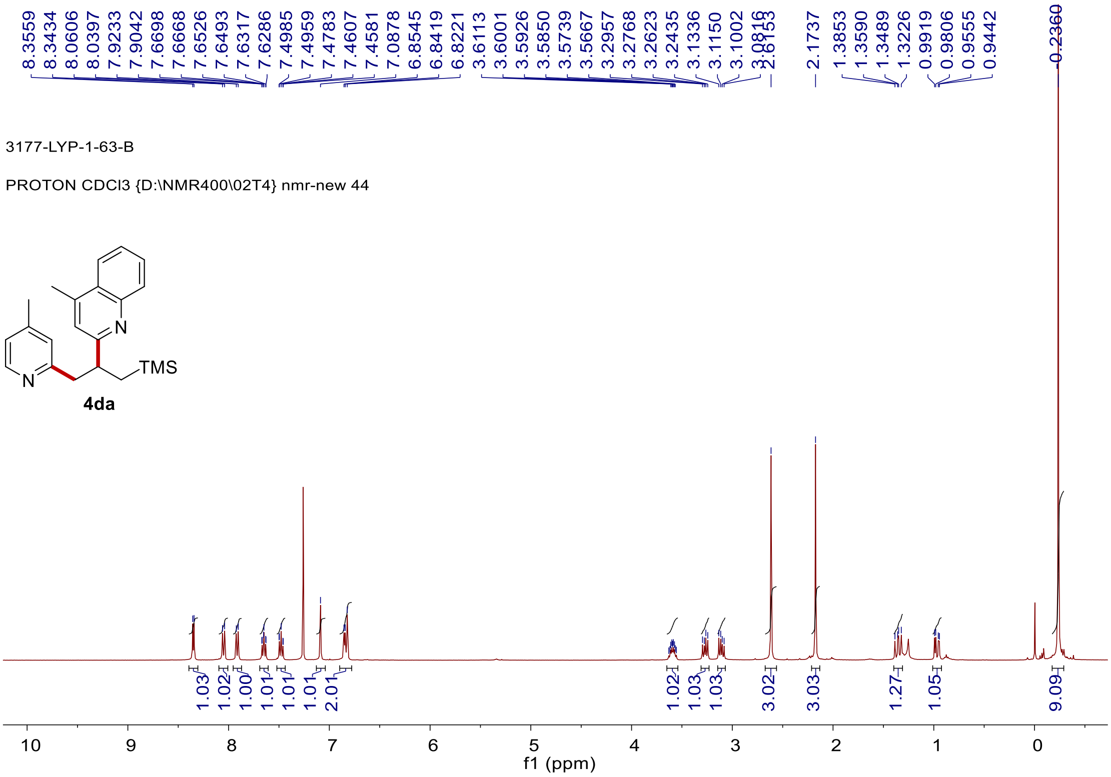

Supplementary Figure 63. ^{1}H NMR spectra of compound **4da**.

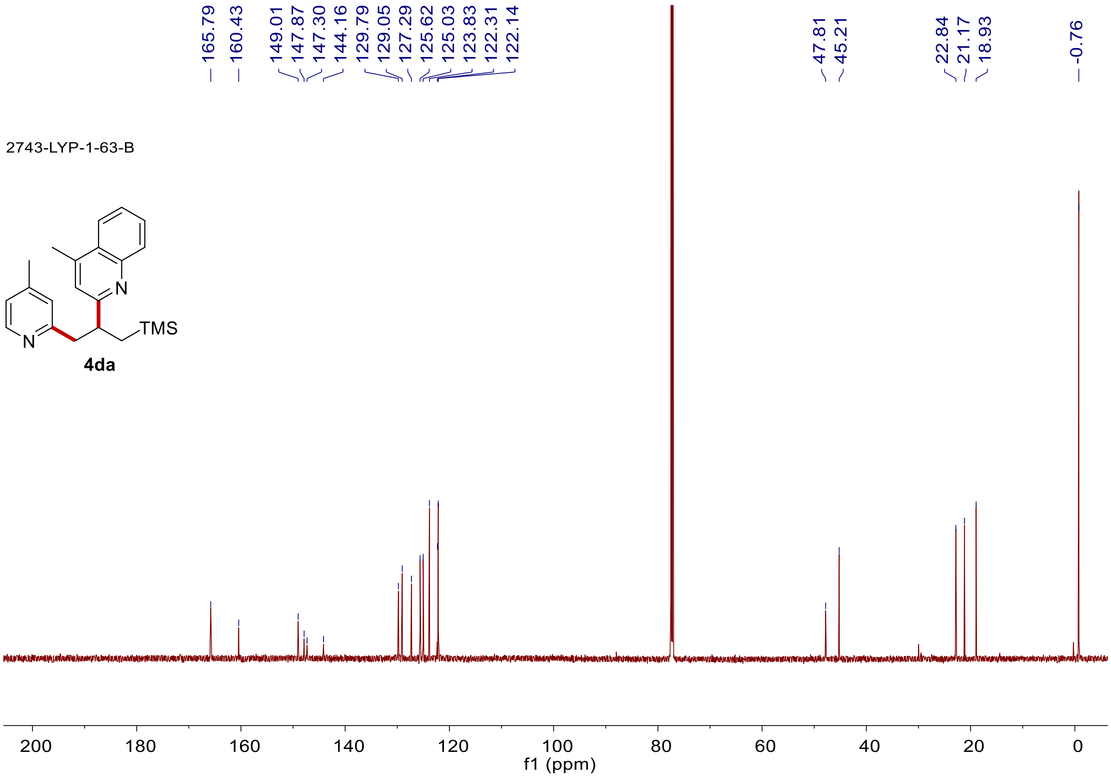

Supplementary Figure 64. ^{13}C NMR spectra of compound **4da**.

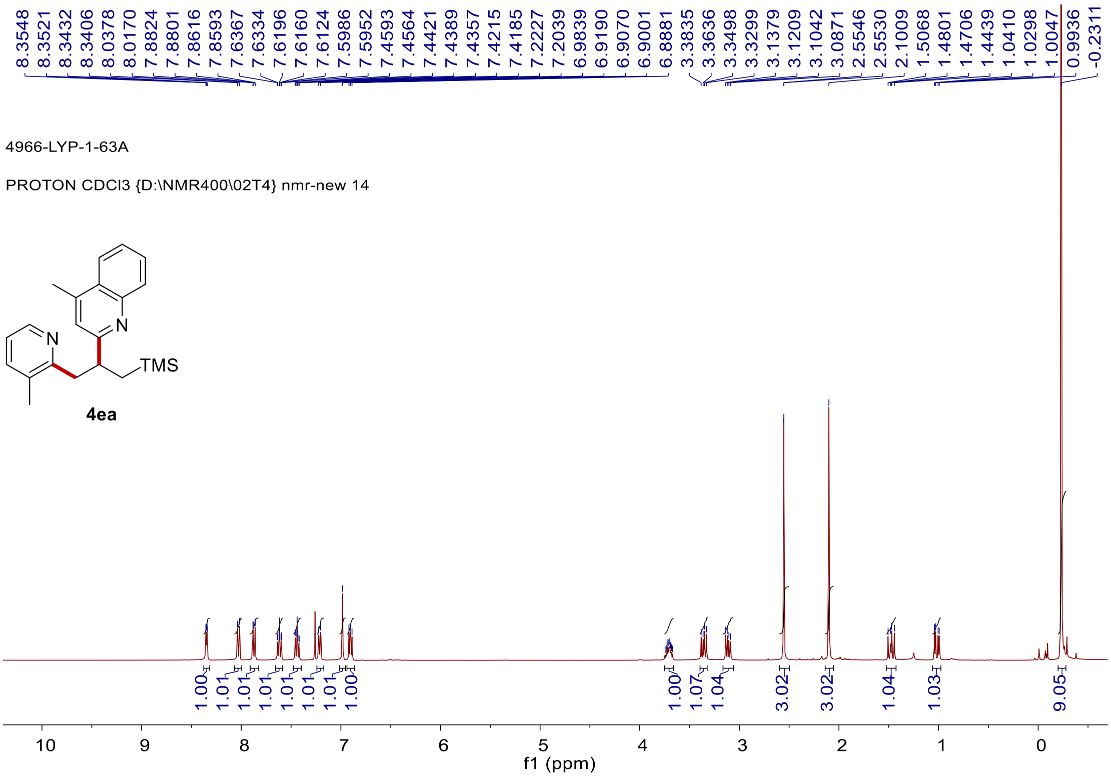

Supplementary Figure 65. ^{1}H NMR spectra of compound **4ea**.

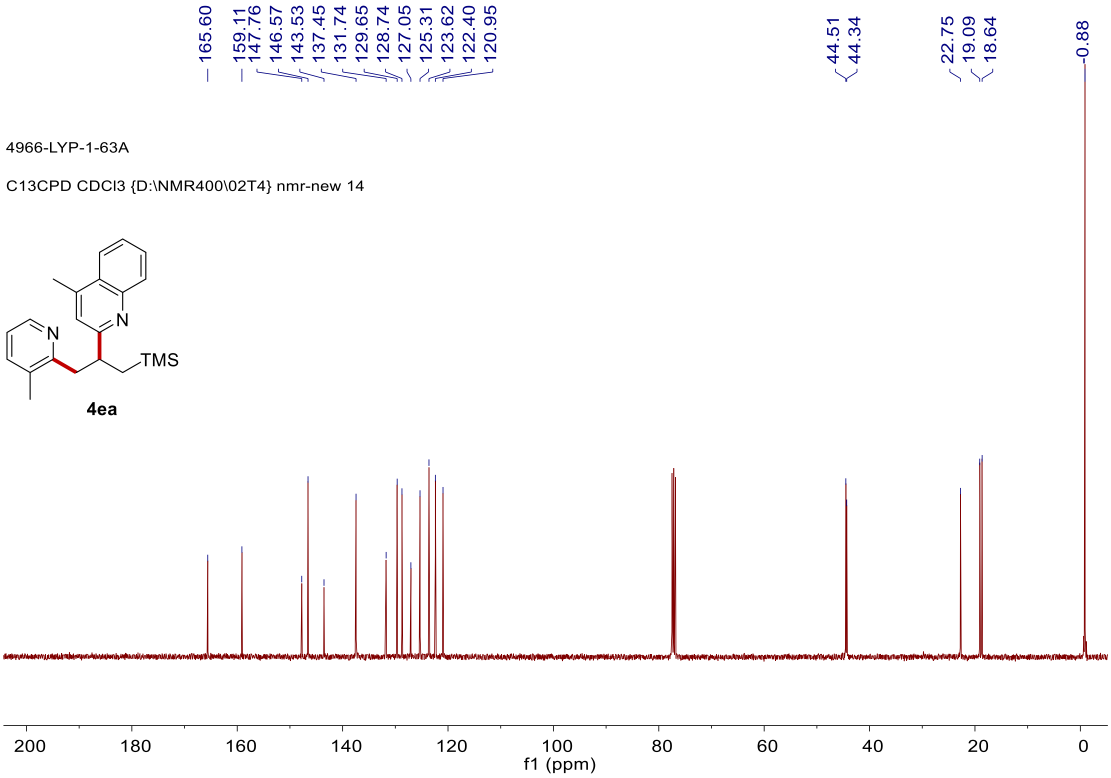

Supplementary Figure 66. ^{13}C NMR spectra of compound **4ea**.

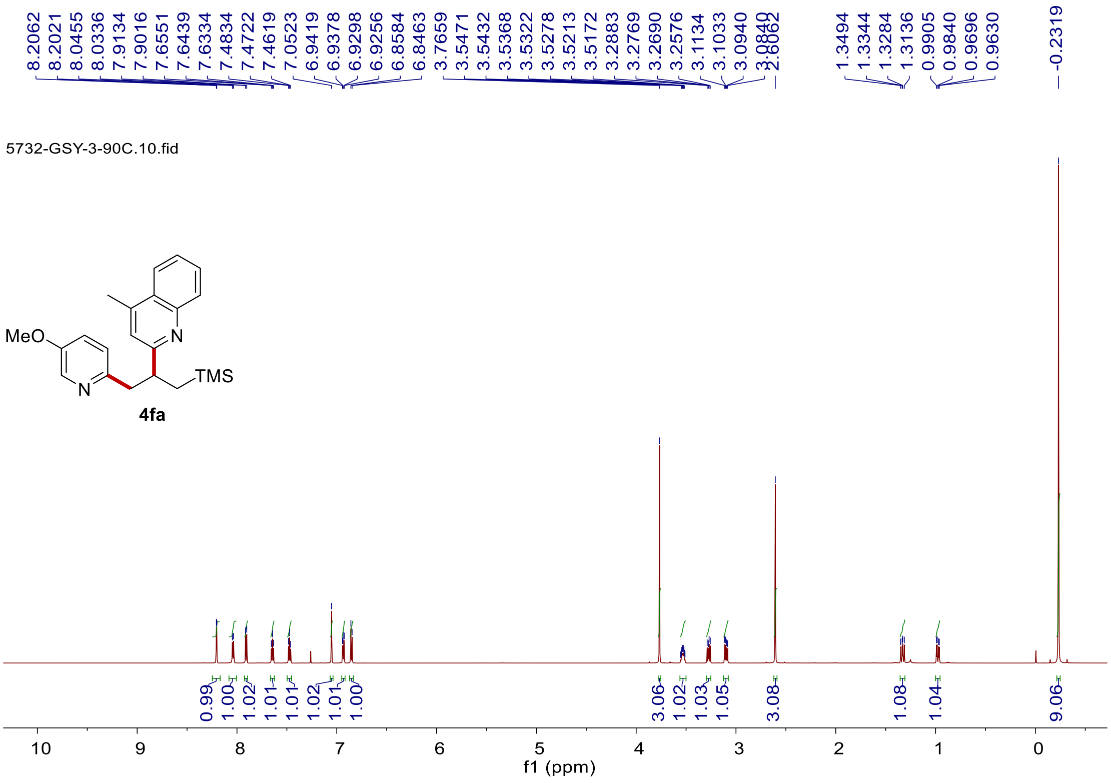

Supplementary Figure 67. ^{1}H NMR spectra of compound **4fa**.

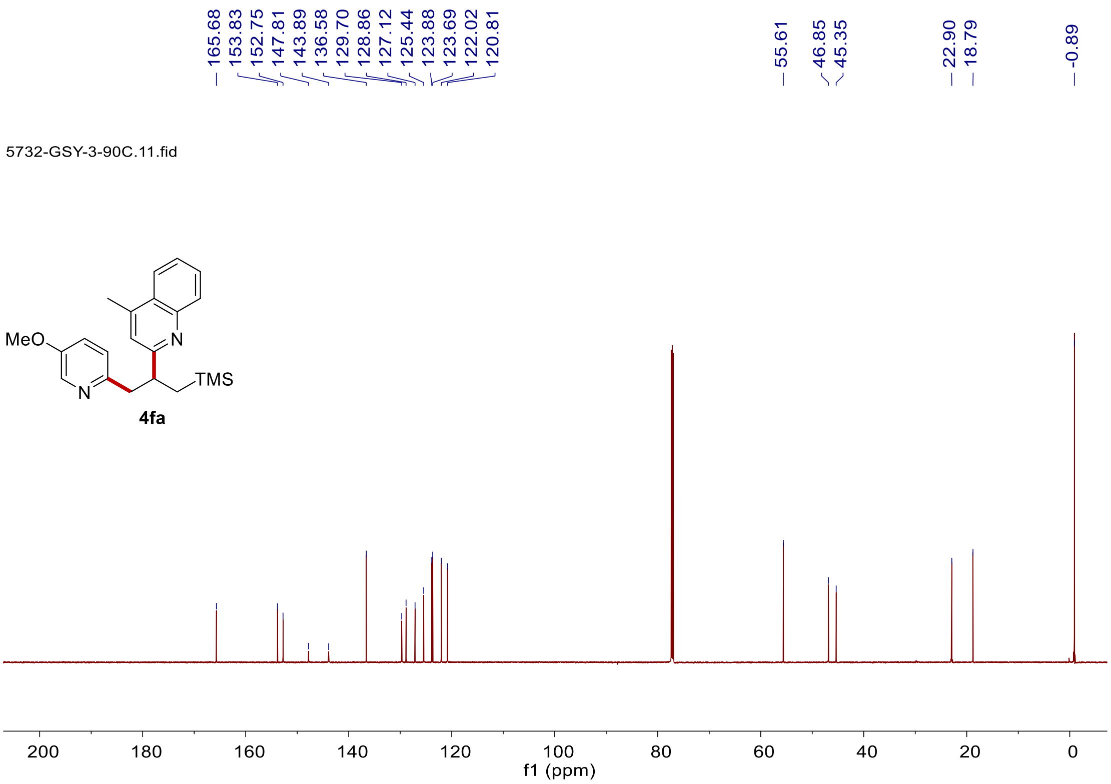

Supplementary Figure 68. ^{13}C NMR spectra of compound **4fa**.

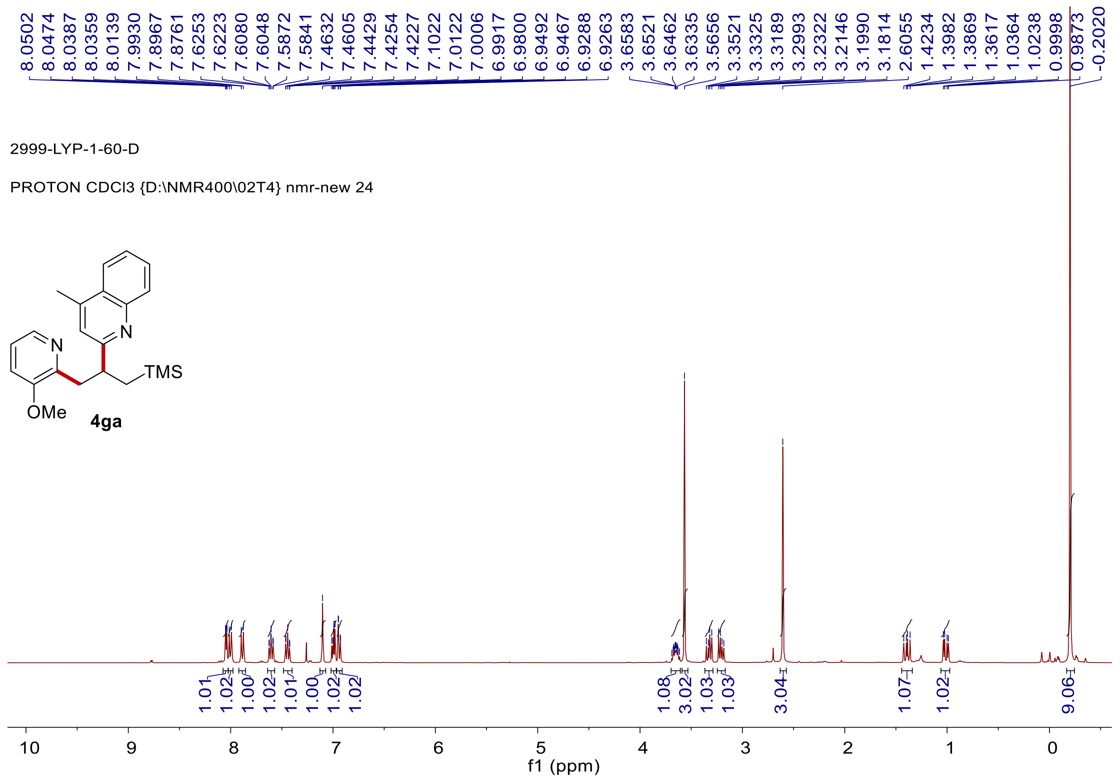

Supplementary Figure 69. ^{1}H NMR spectra of compound **4ga**.

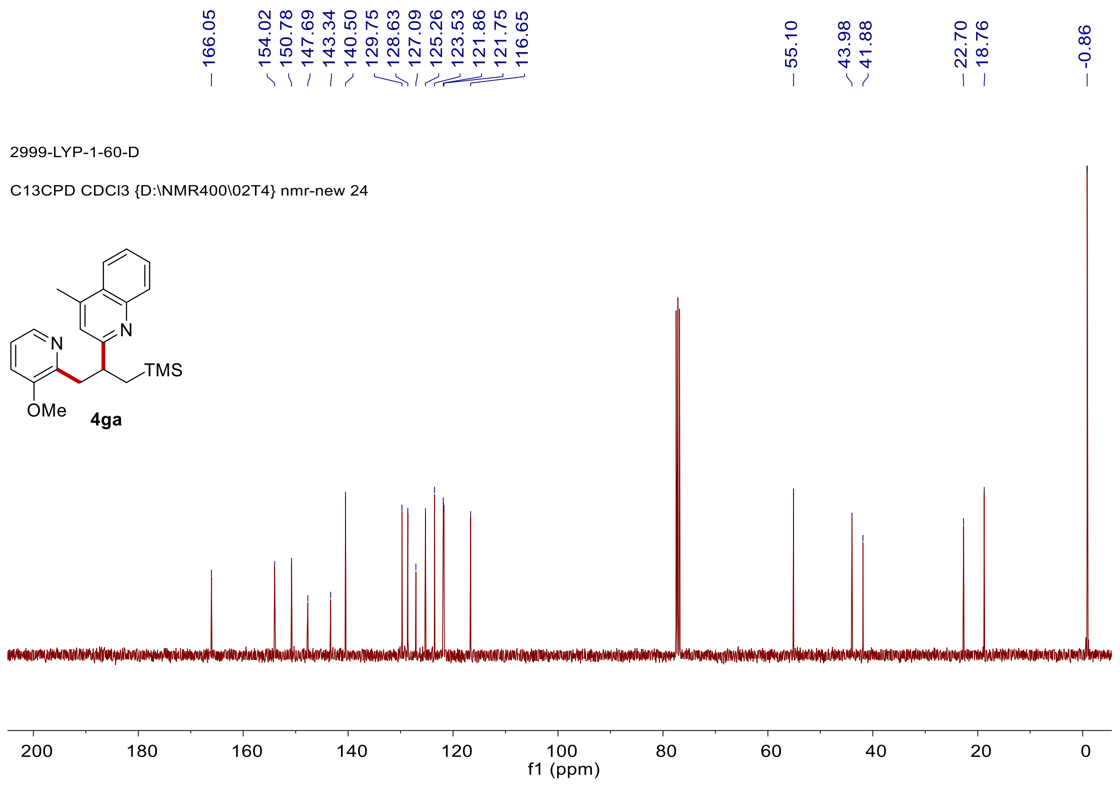

Supplementary Figure 70. ^{13}C NMR spectra of compound **4ga**.

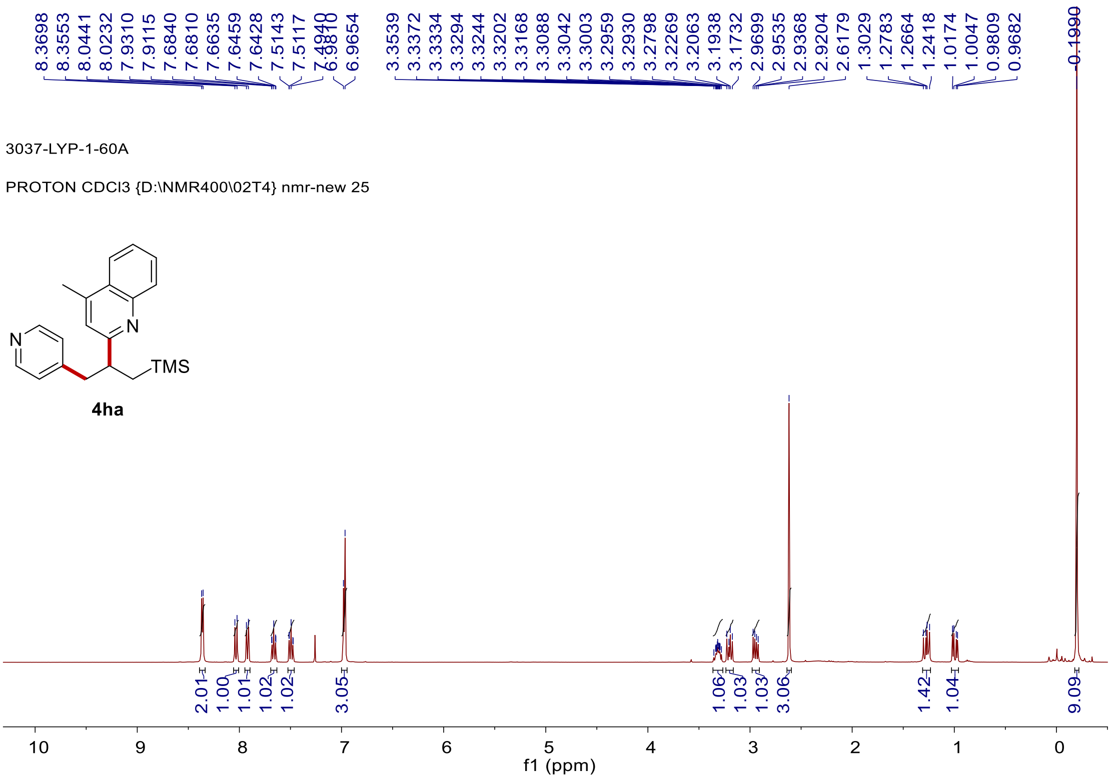

Supplementary Figure 71. ^{1}H NMR spectra of compound **4ha**.

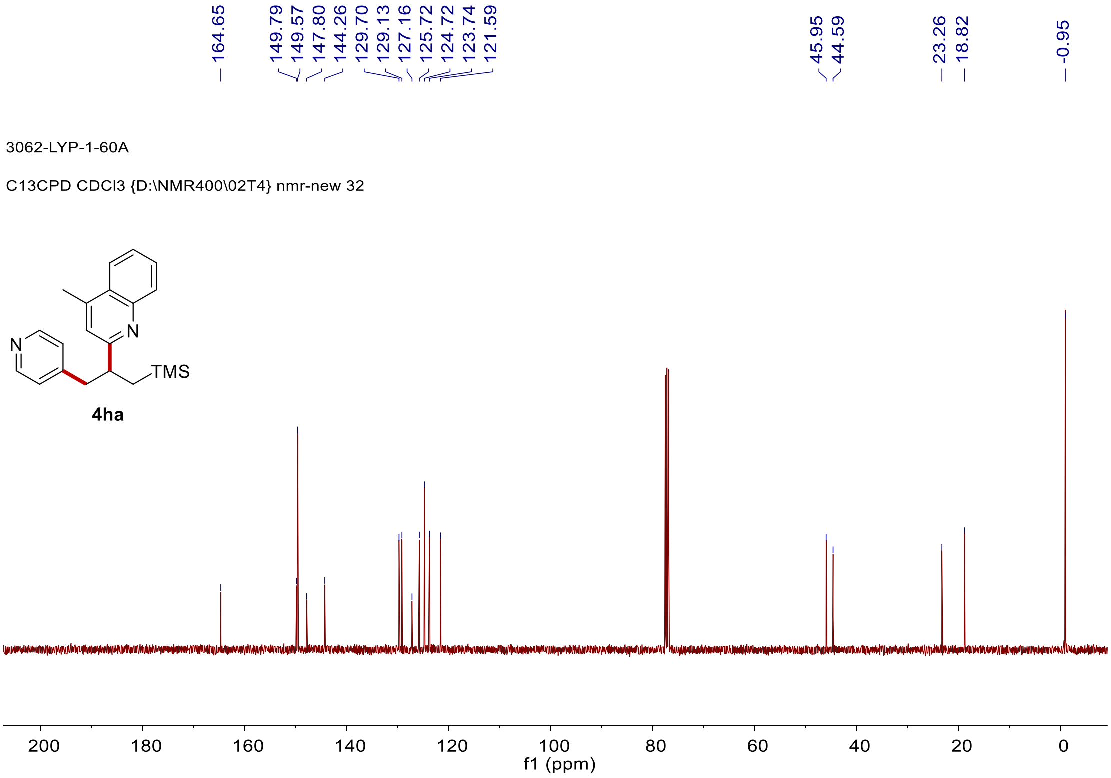

Supplementary Figure 72. ^{13}C NMR spectra of compound **4ha**.

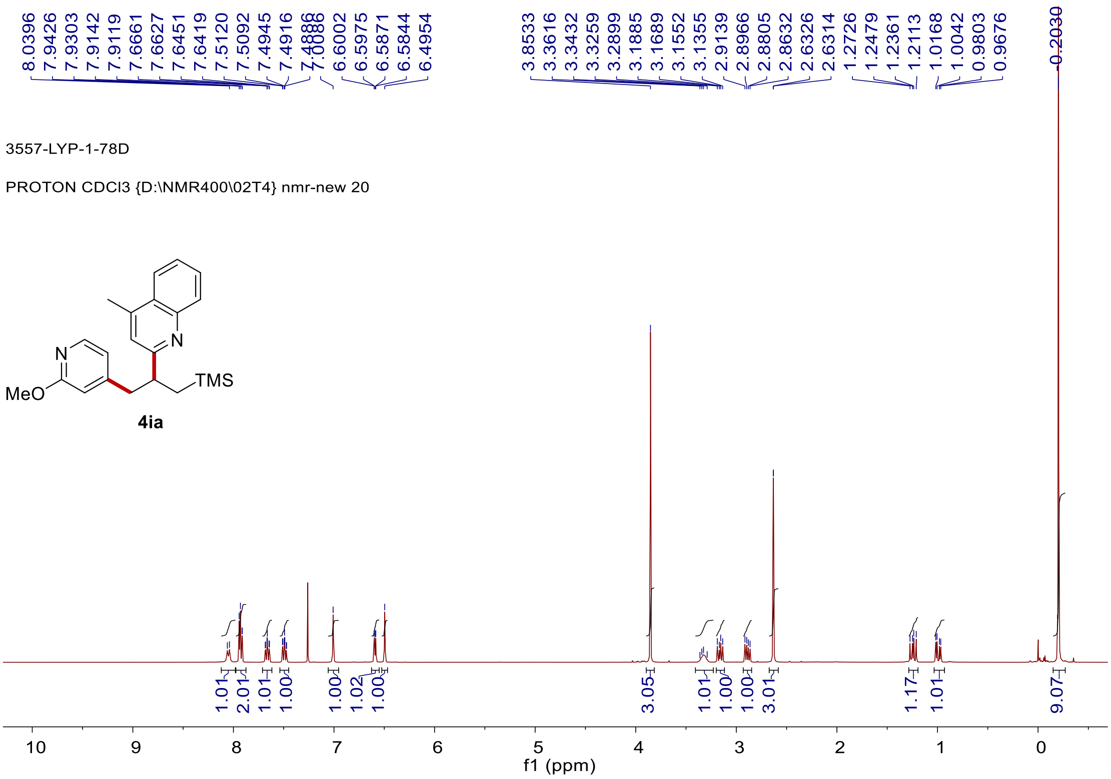

Supplementary Figure 73. ^{1}H NMR spectra of compound **4ia**.

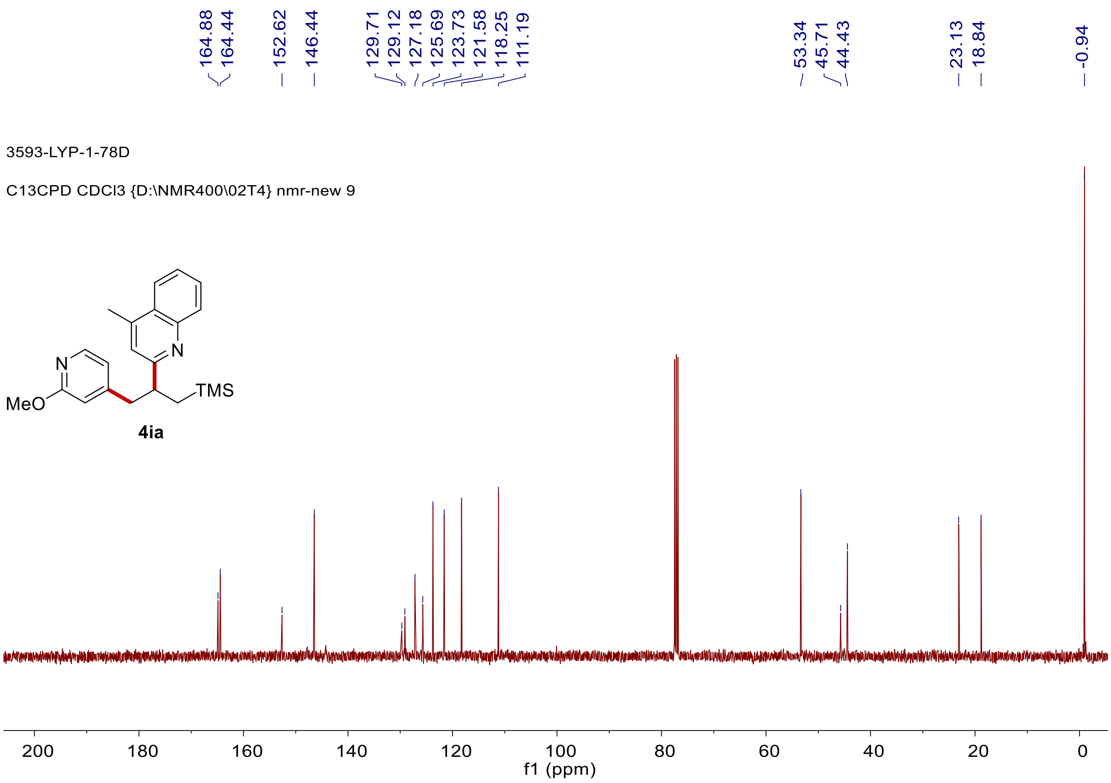

Supplementary Figure 74. ^{13}C NMR spectra of compound **4ia**.

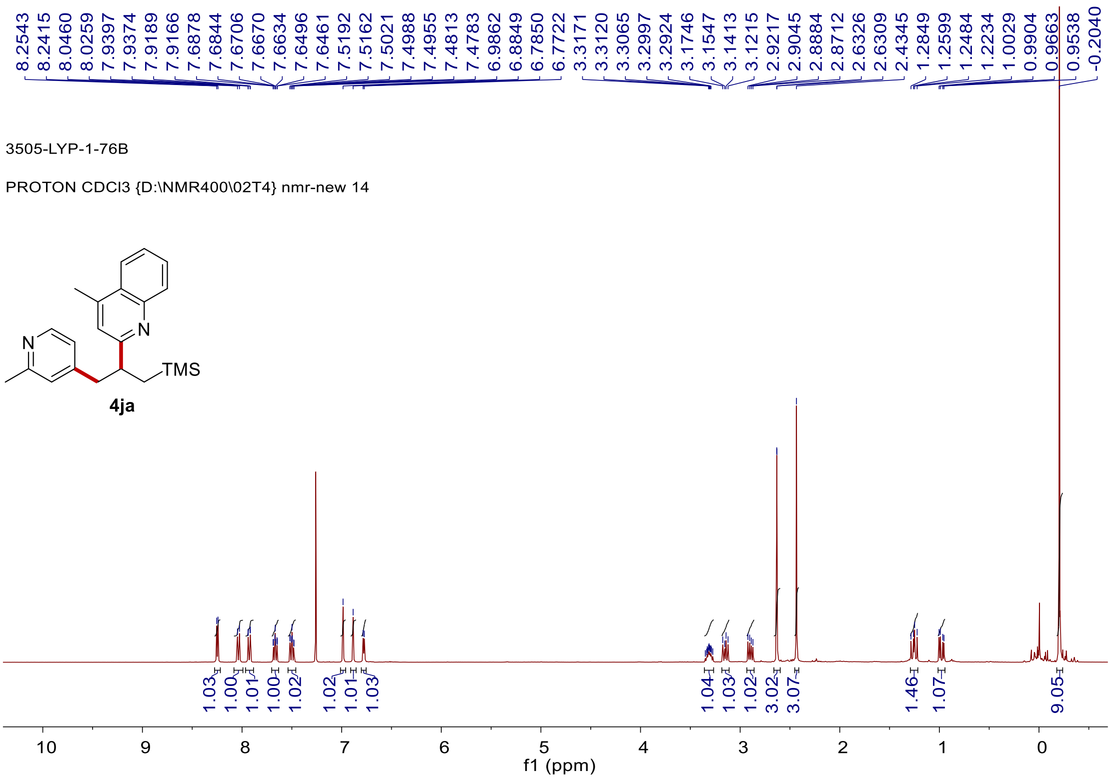

Supplementary Figure 75. ^{1}H NMR spectra of compound **4ja**.

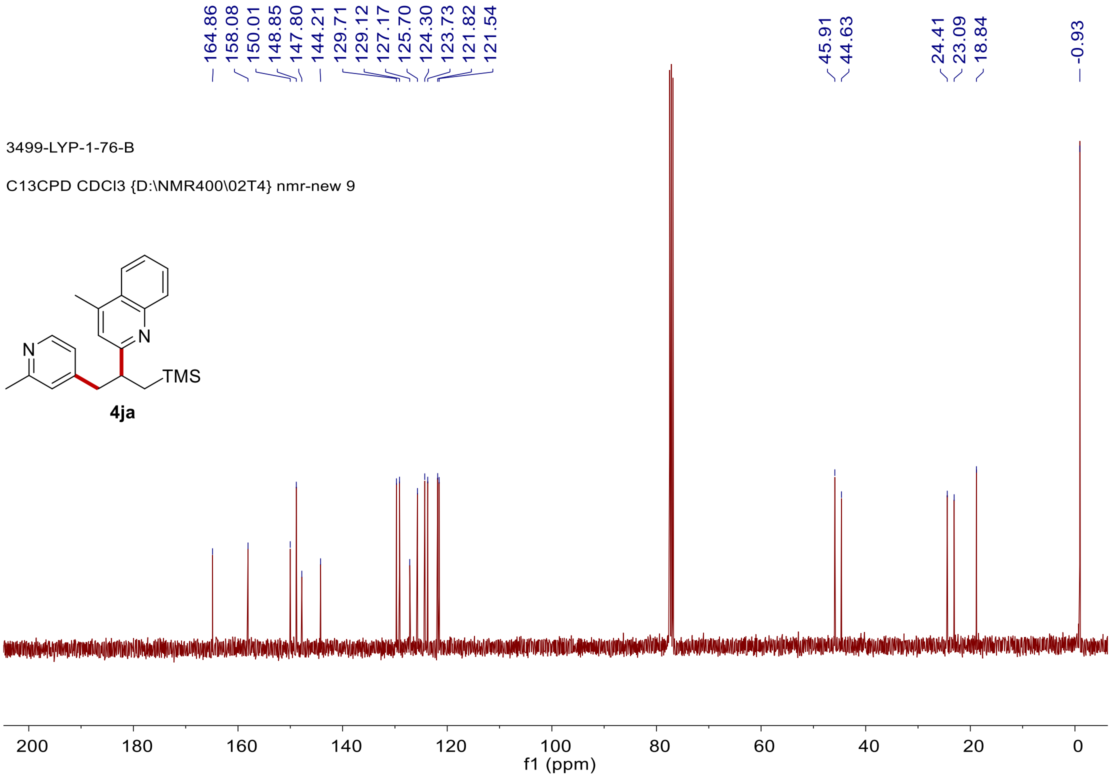

Supplementary Figure 76. ^{13}C NMR spectra of compound **4ja**.

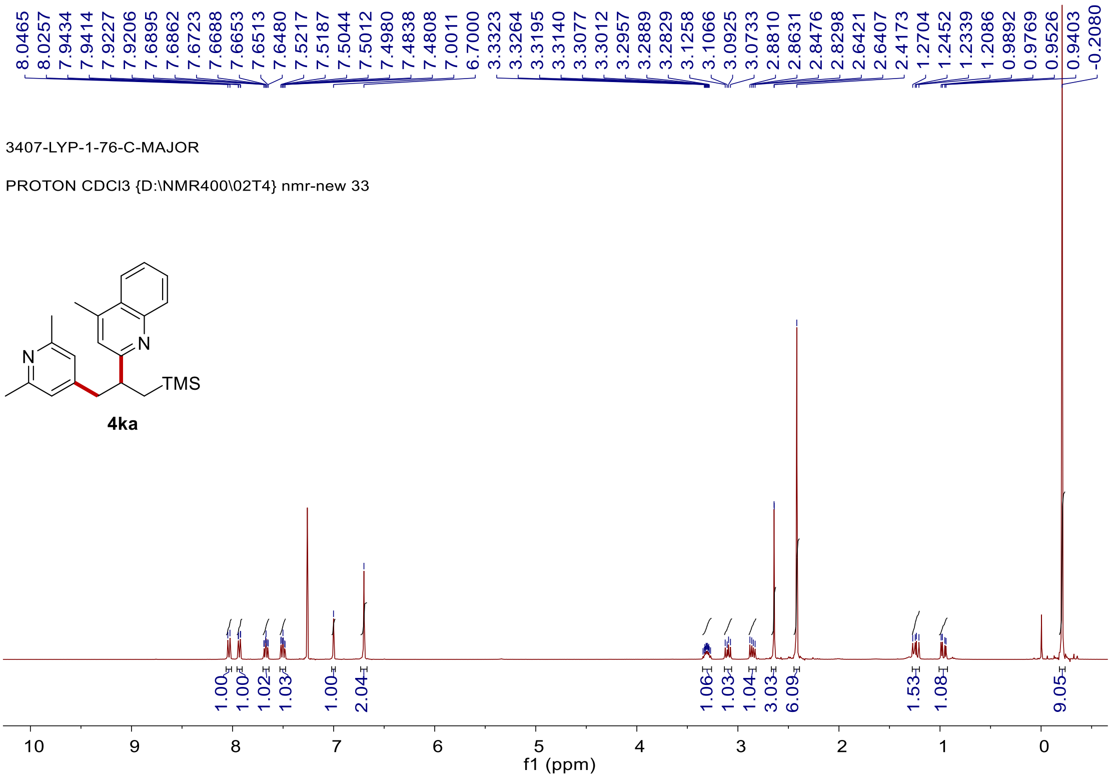

Supplementary Figure 77. ^{1}H NMR spectra of compound **4ka**.

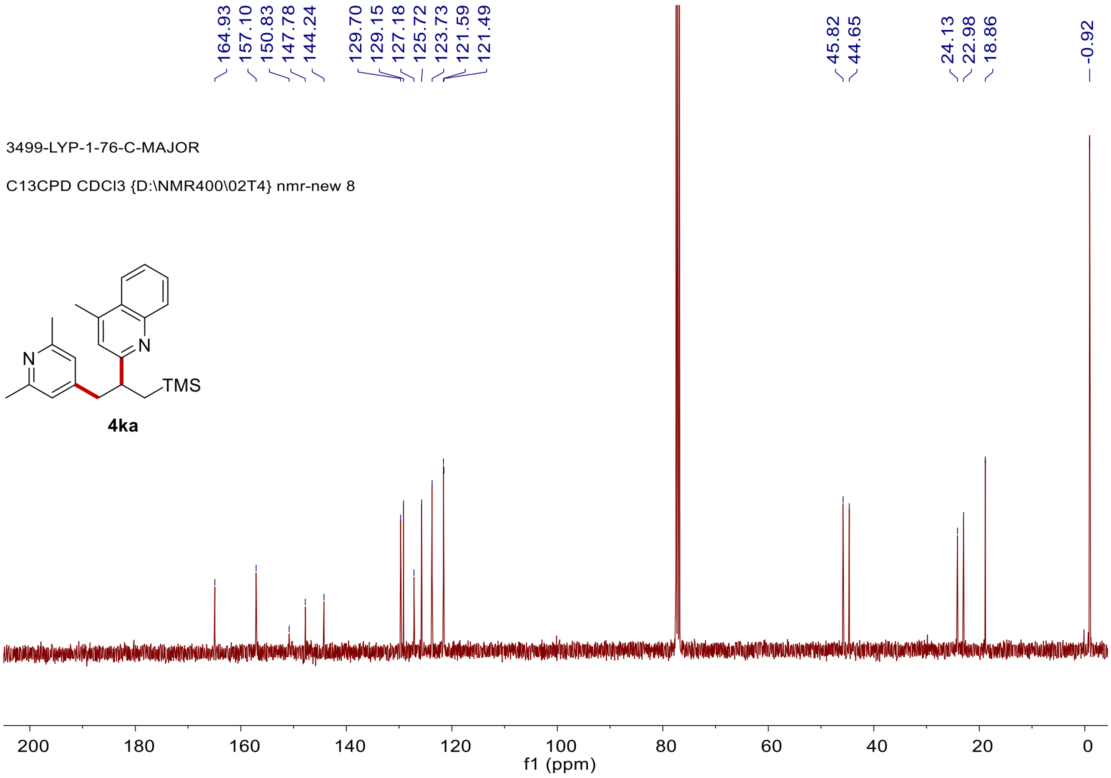

Supplementary Figure 78. ^{13}C NMR spectra of compound **4ka**.

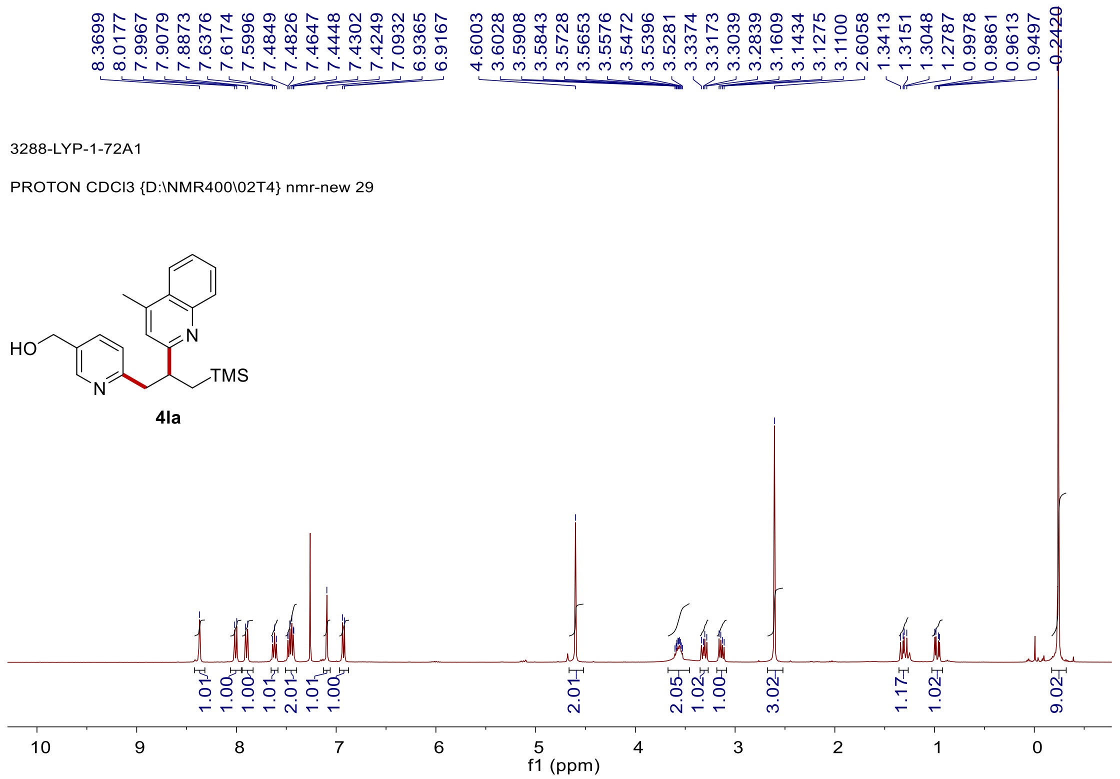

Supplementary Figure 79. ^{1}H NMR spectra of compound **4la**.

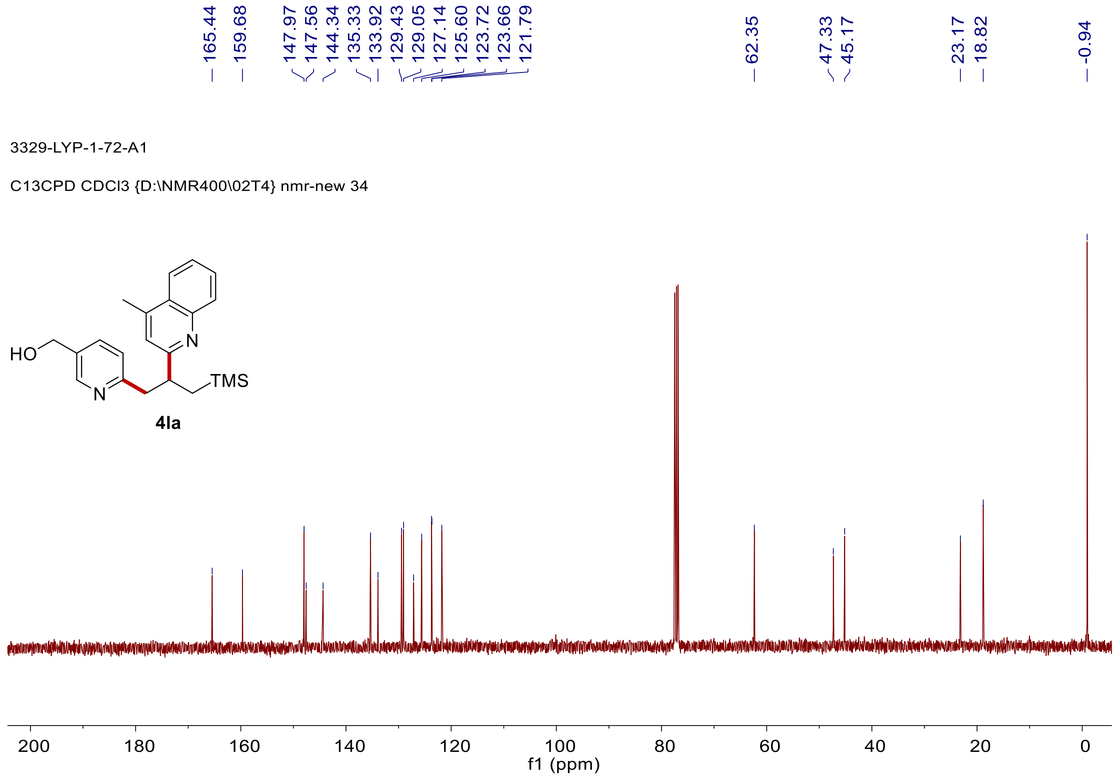

Supplementary Figure 80. ^{13}C NMR spectra of compound **4la**.

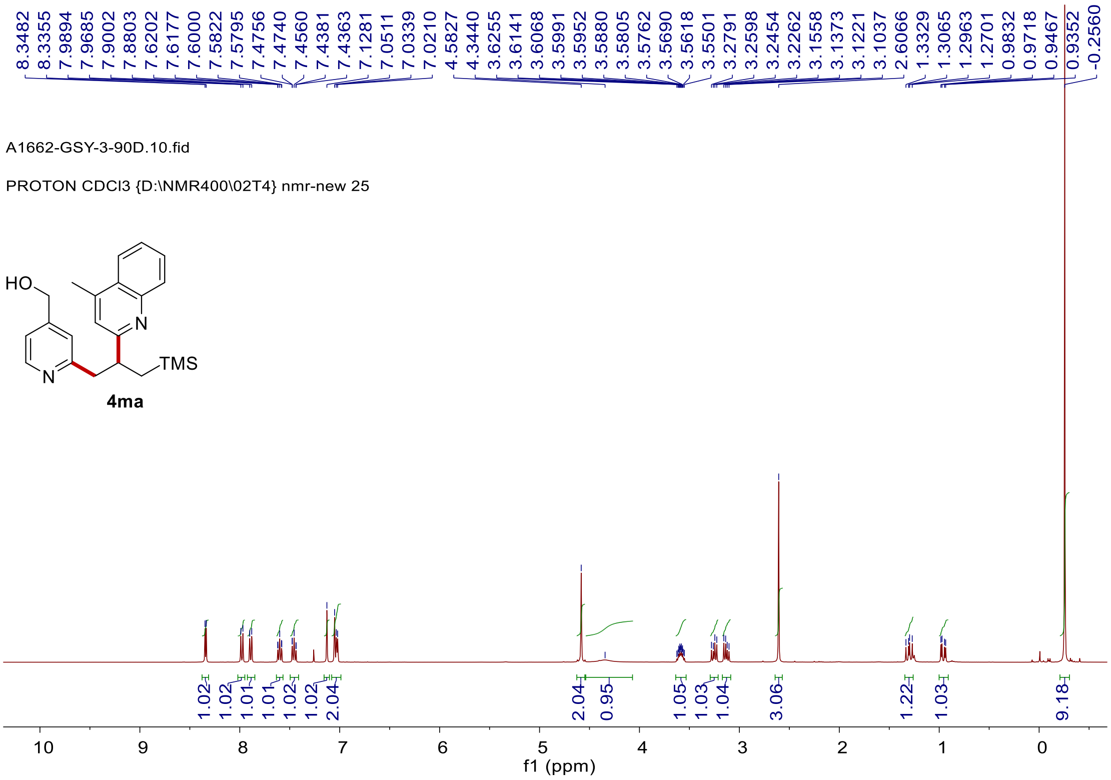

Supplementary Figure 81. ^{1}H NMR spectra of compound **4ma**.

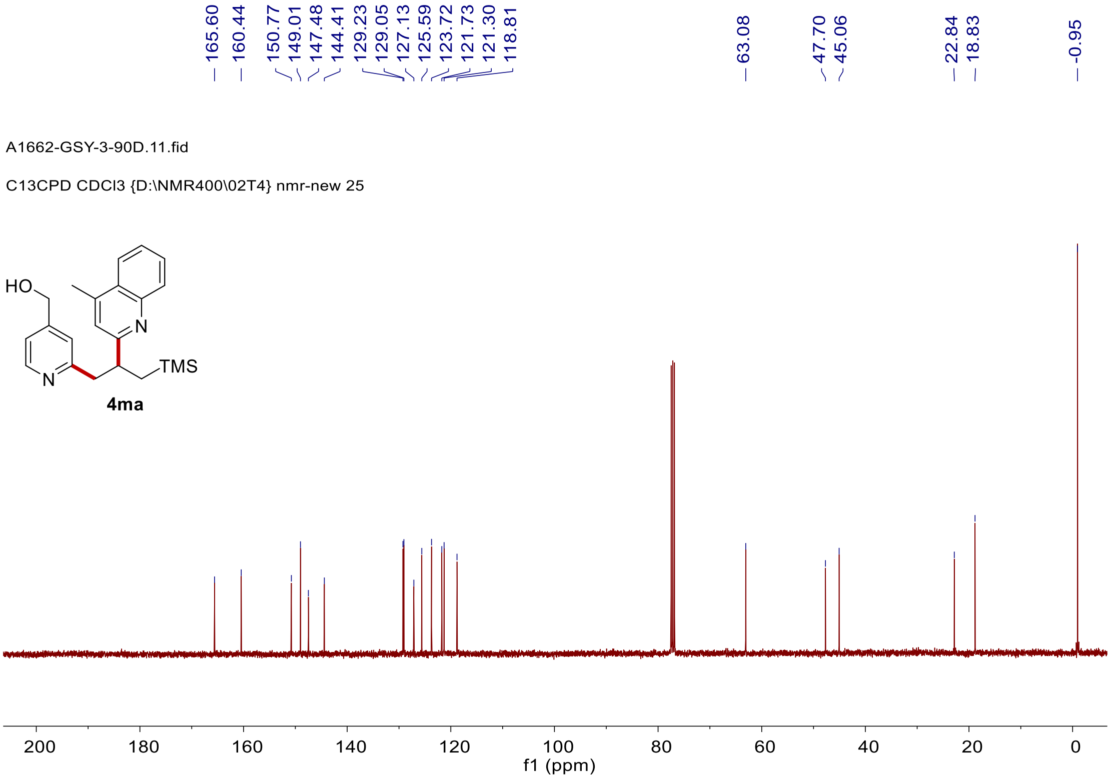

Supplementary Figure 82. ^{13}C NMR spectra of compound **4ma**.

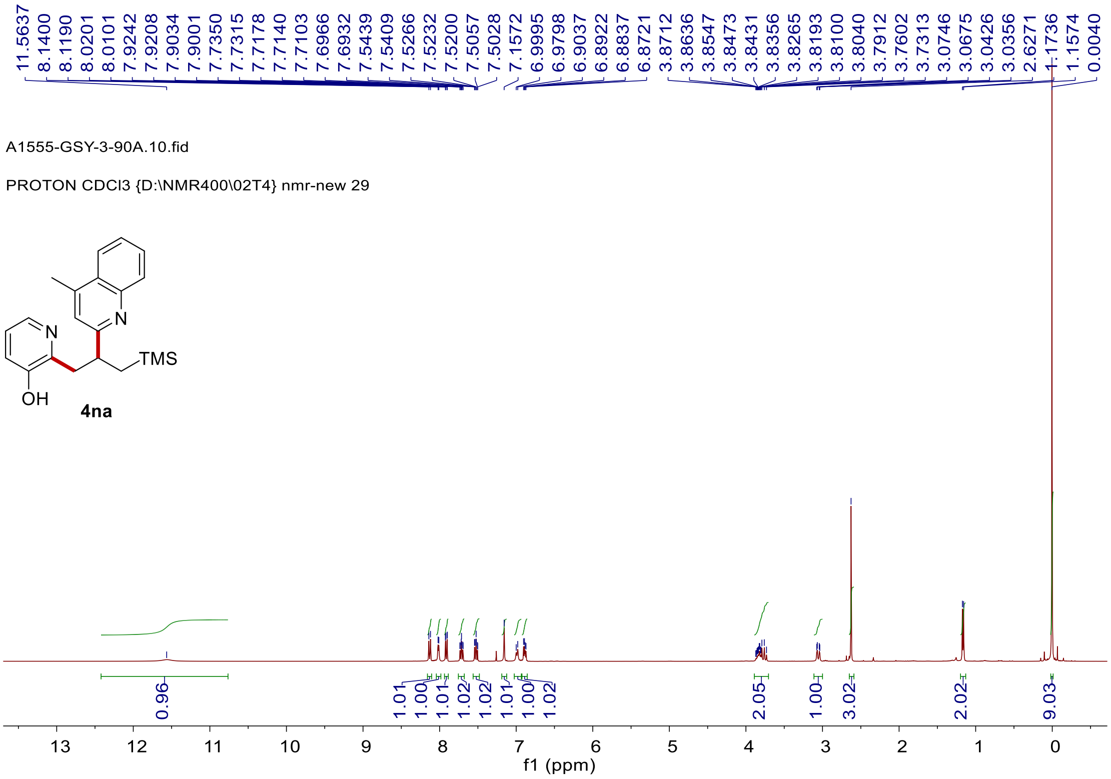

Supplementary Figure 83. ^{1}H NMR spectra of compound **4na**.

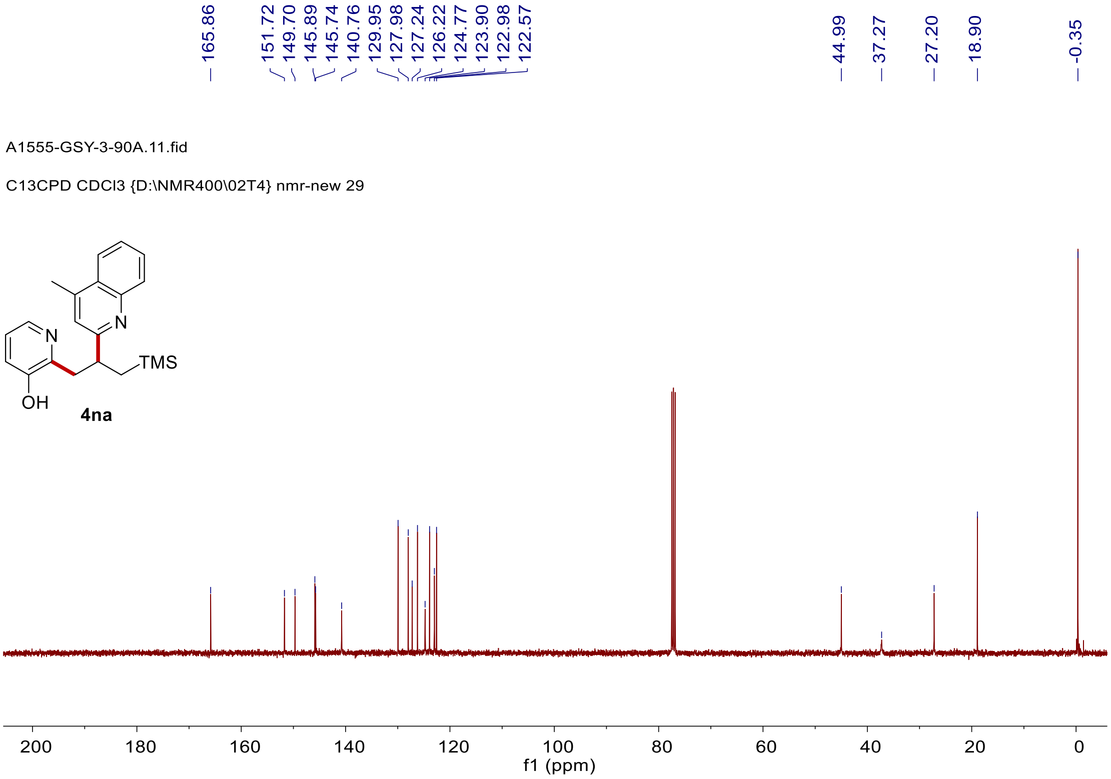

Supplementary Figure 84. ^{13}C NMR spectra of compound **4na**.

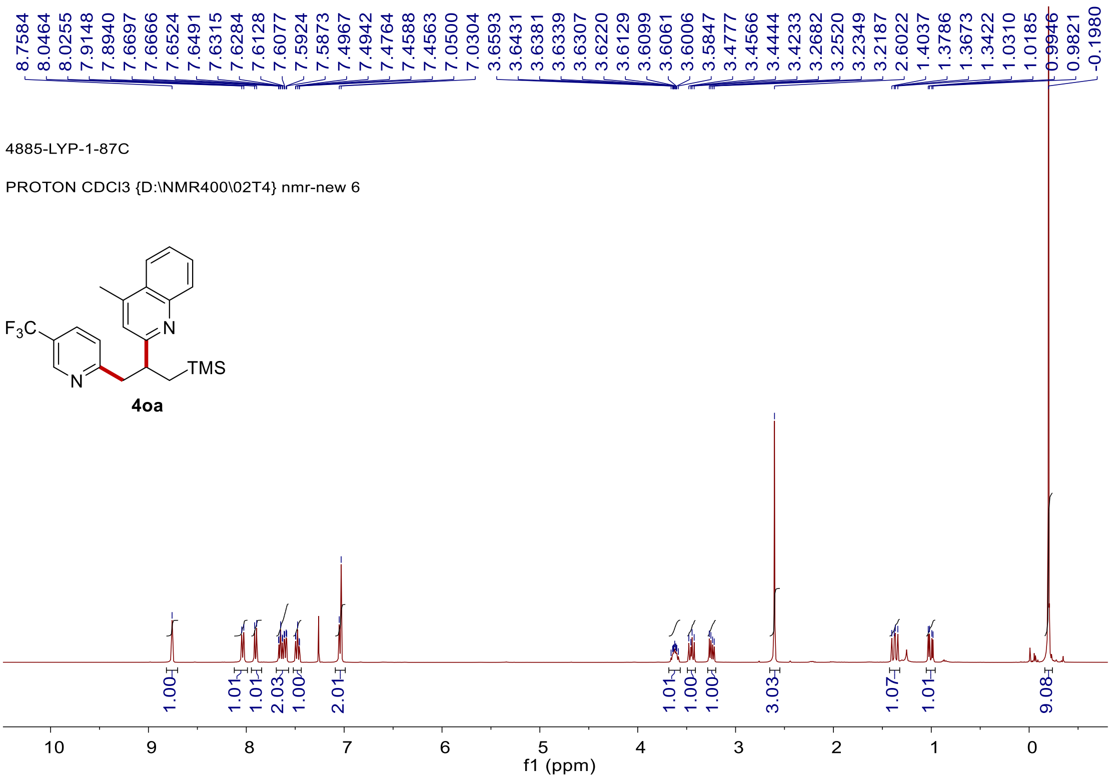

Supplementary Figure 85. ^{1}H NMR spectra of compound **4oa**.

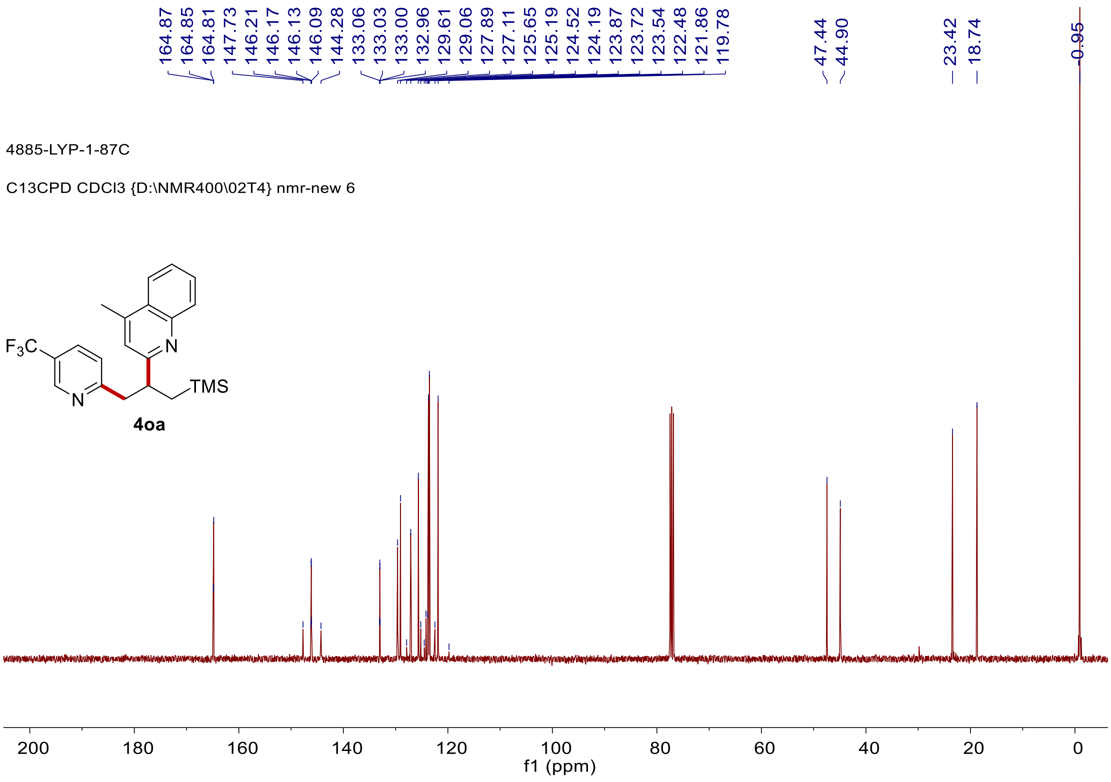

Supplementary Figure 86. ^{13}C NMR spectra of compound **4oa**.

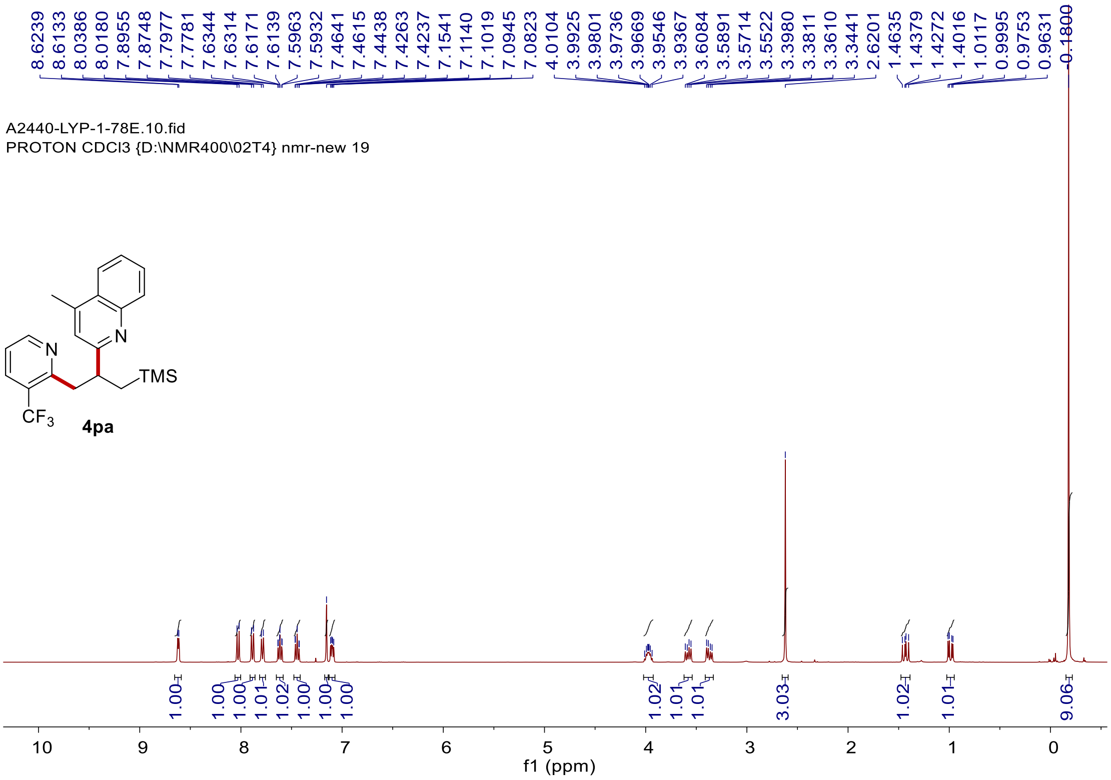

Supplementary Figure 87. ^{1}H NMR spectra of compound **4pa**.

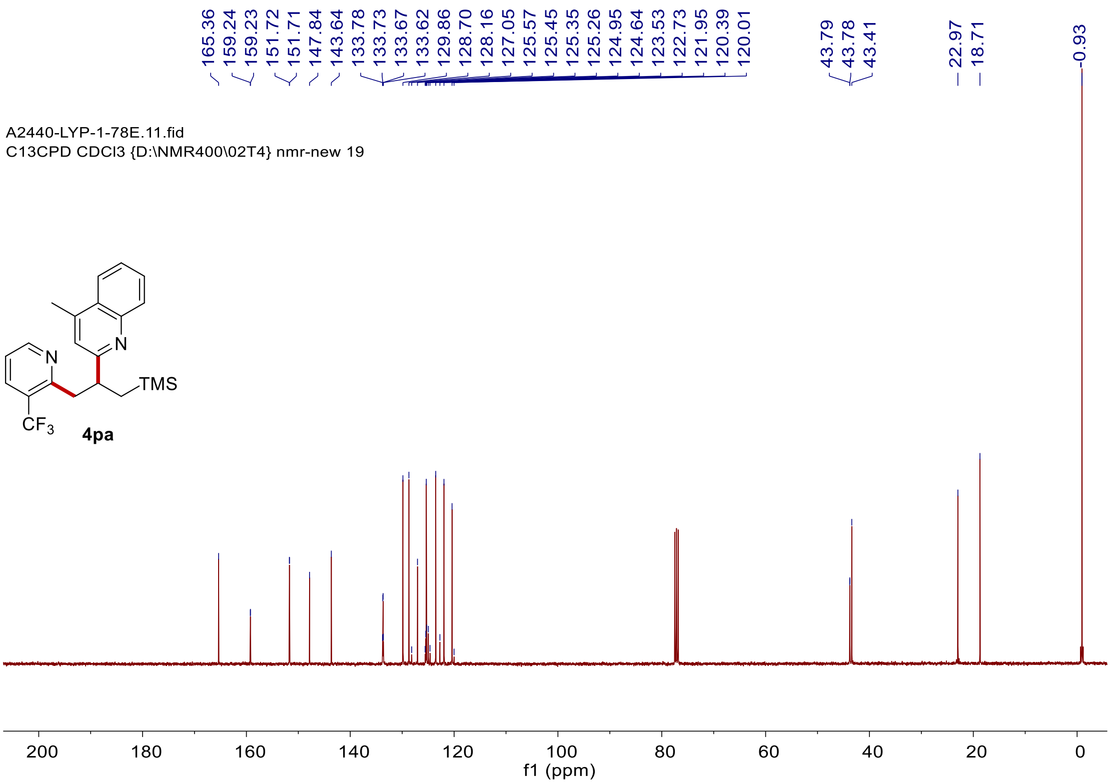

Supplementary Figure 88. ^{13}C NMR spectra of compound **4pa**.

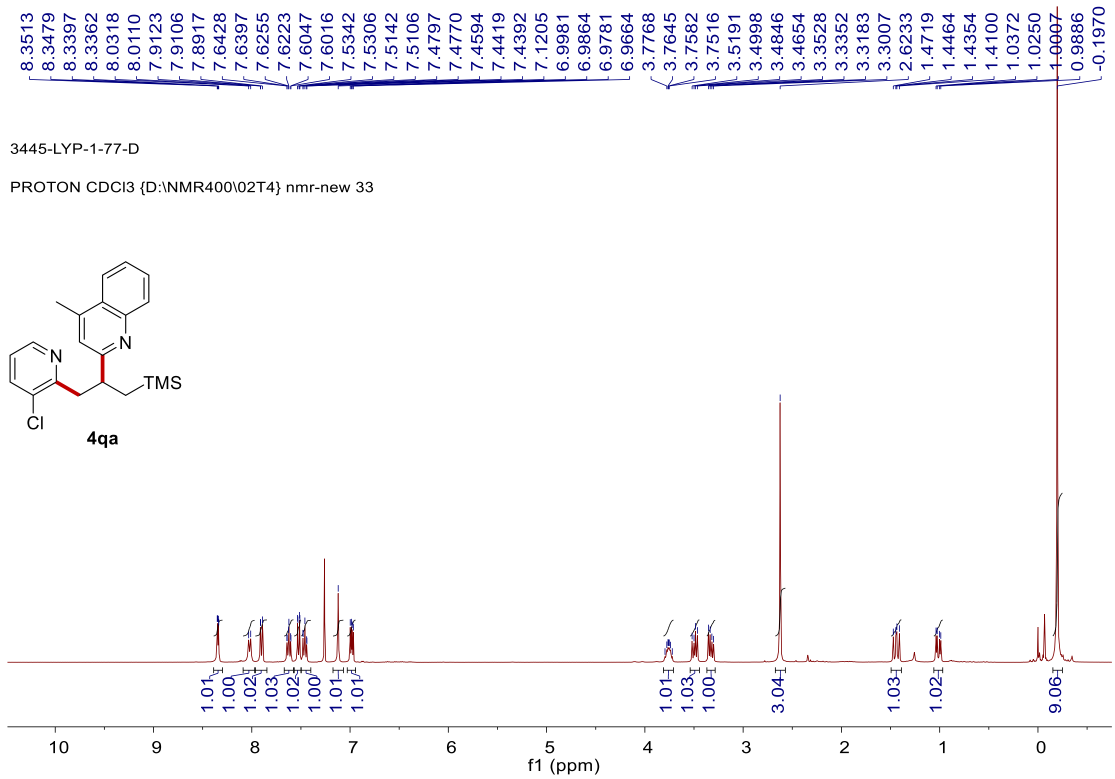

Supplementary Figure 89. ^{1}H NMR spectra of compound **4qa**.

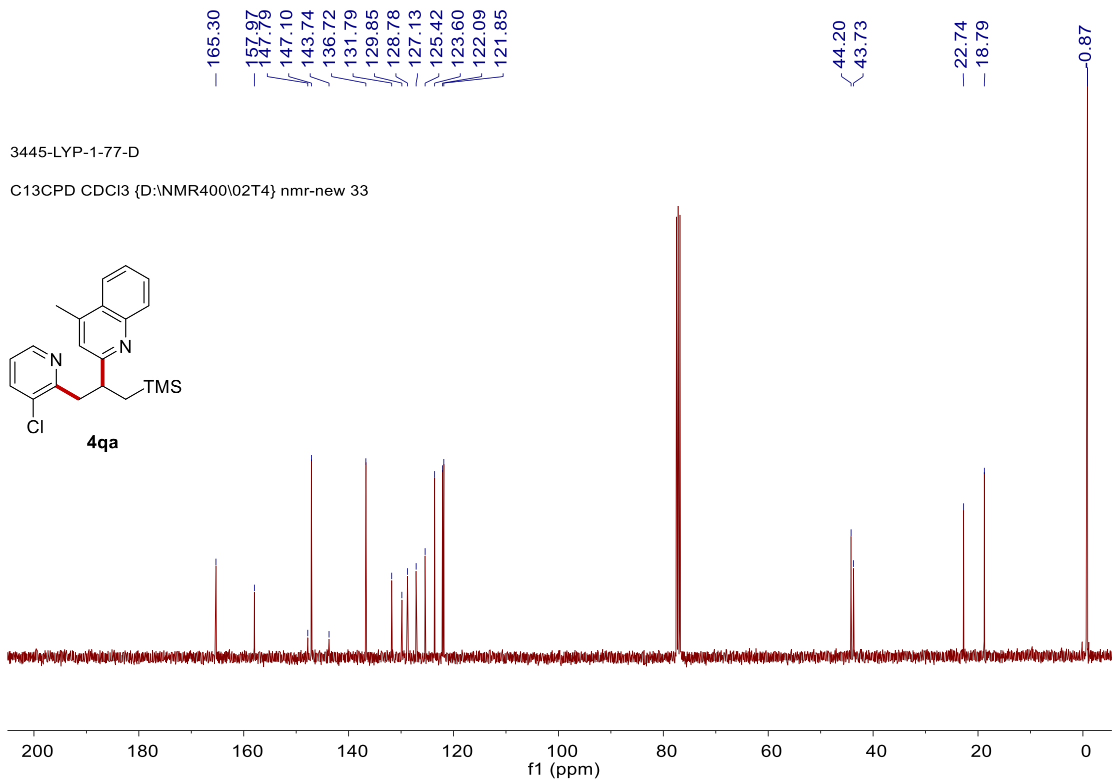

Supplementary Figure 90. ^{13}C NMR spectra of compound **4qa**.

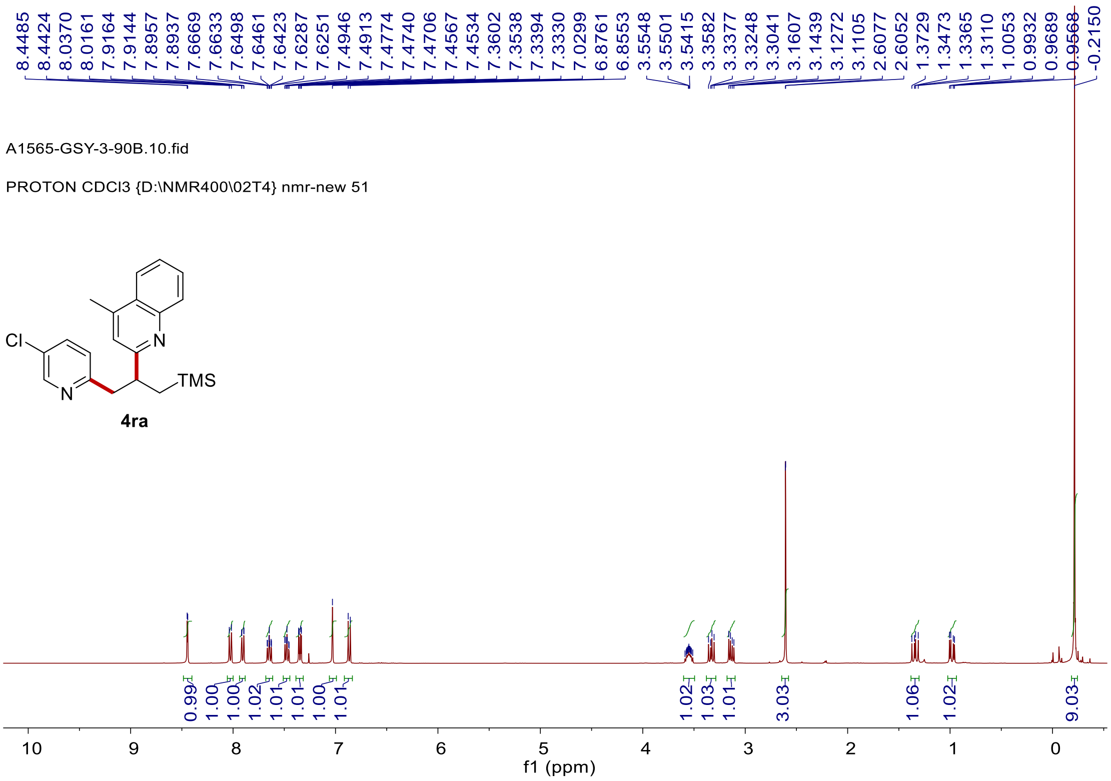

Supplementary Figure 91. ^{1}H NMR spectra of compound **4ra**.

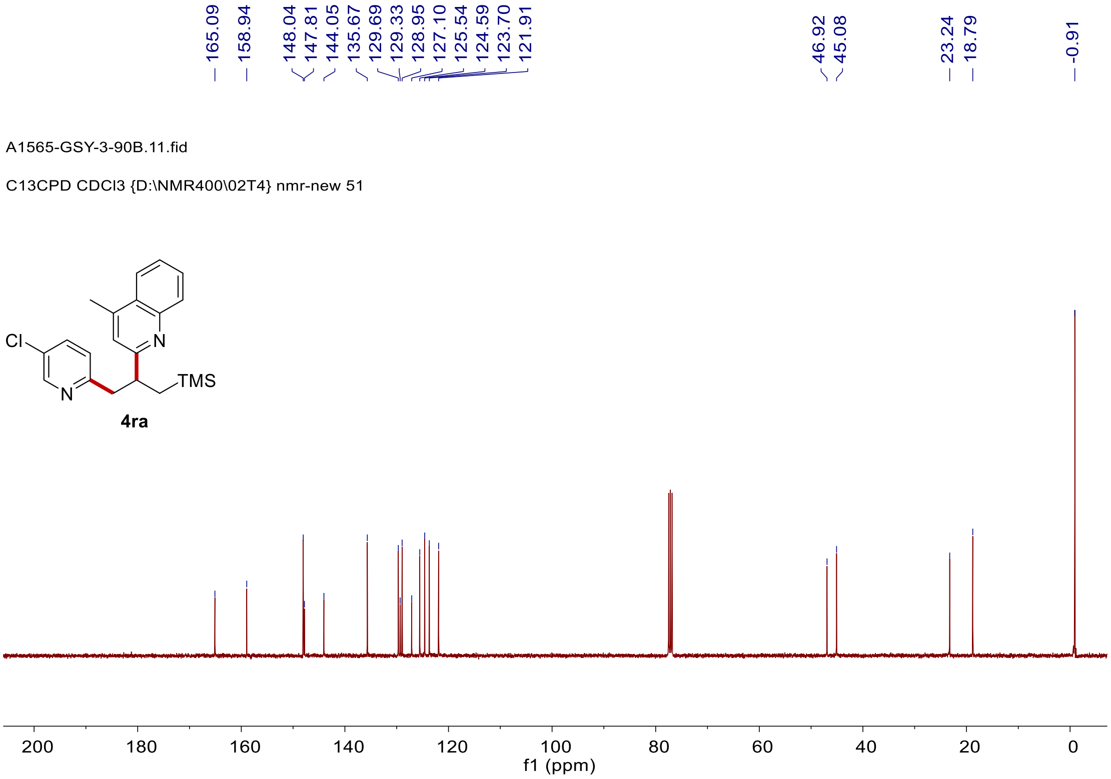

Supplementary Figure 92. ^{13}C NMR spectra of compound **4ra**.

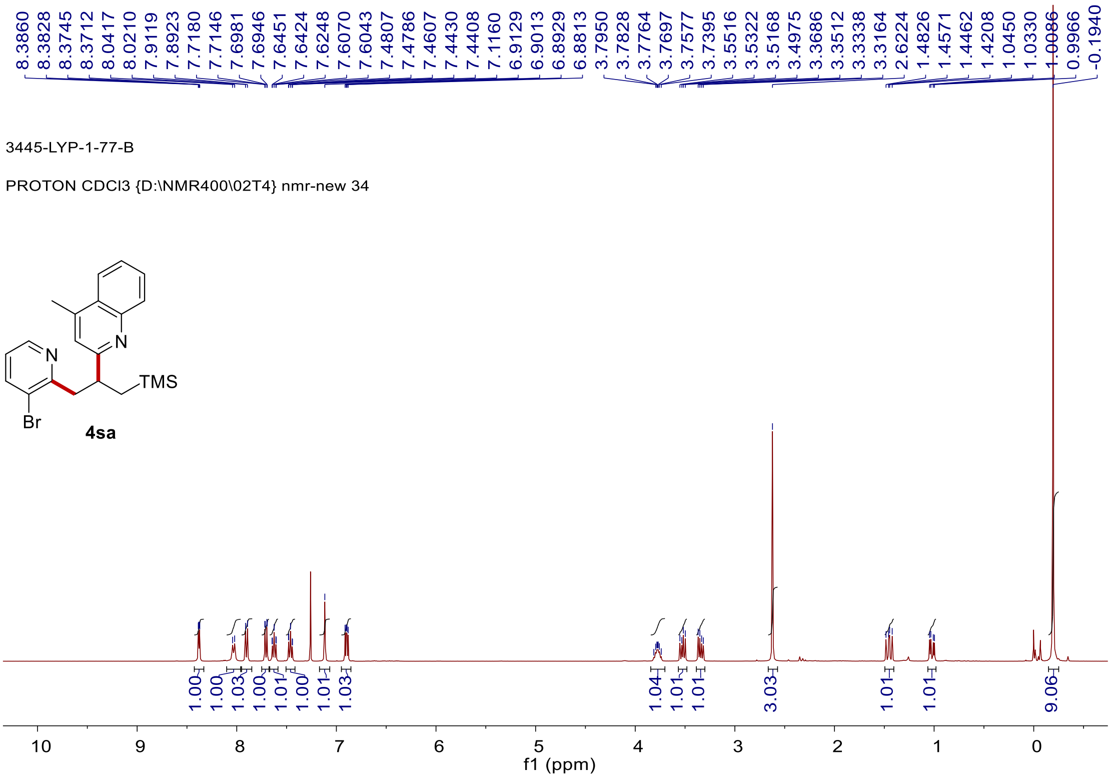

Supplementary Figure 93. ^{1}H NMR spectra of compound **4sa**.

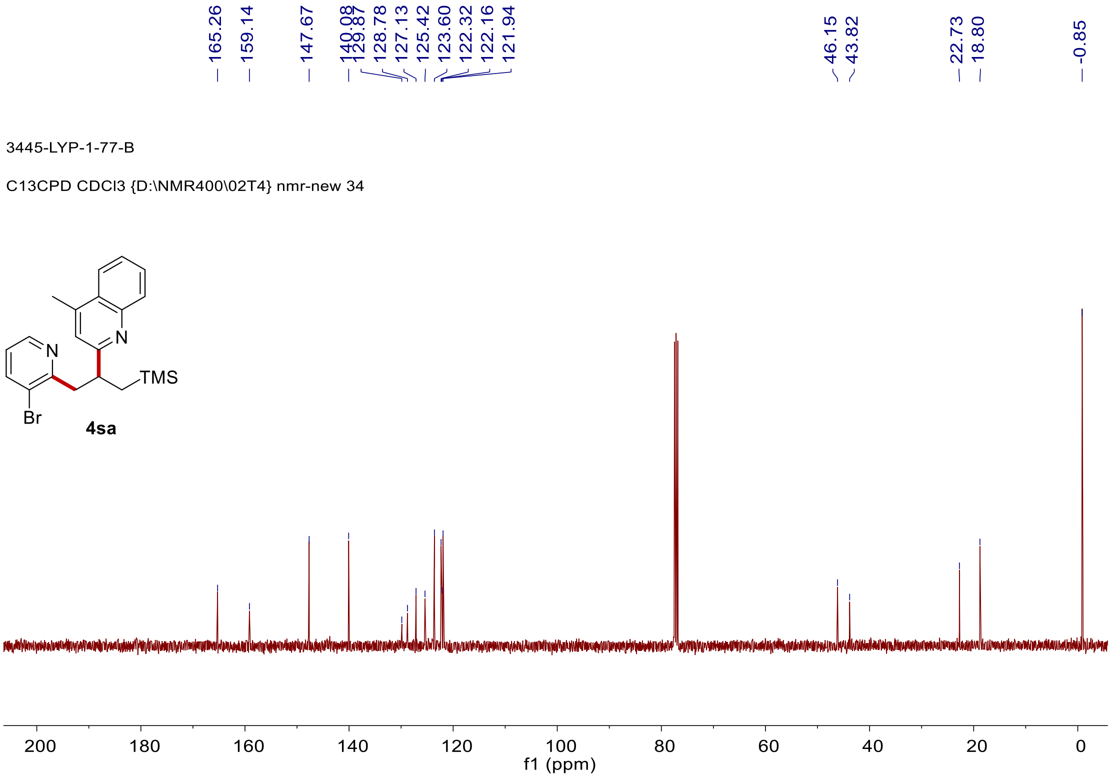

Supplementary Figure 94. ^{13}C NMR spectra of compound **4sa**.

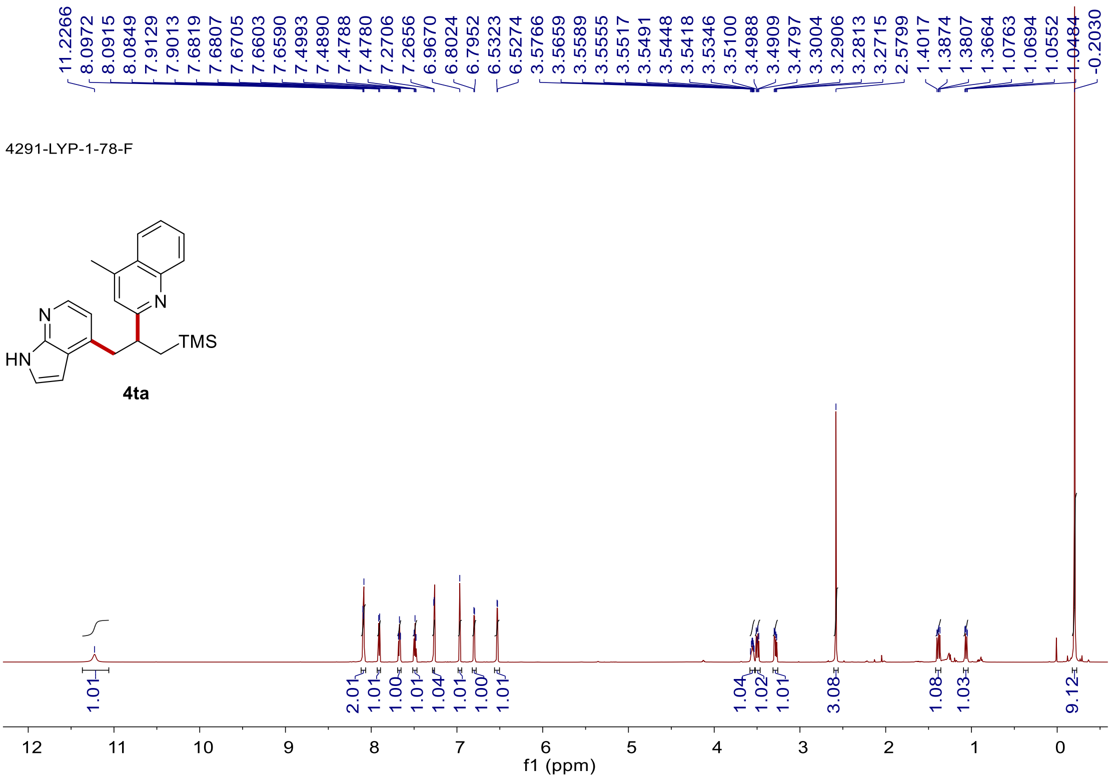

Supplementary Figure 95. ^{1}H NMR spectra of compound **4ta**.

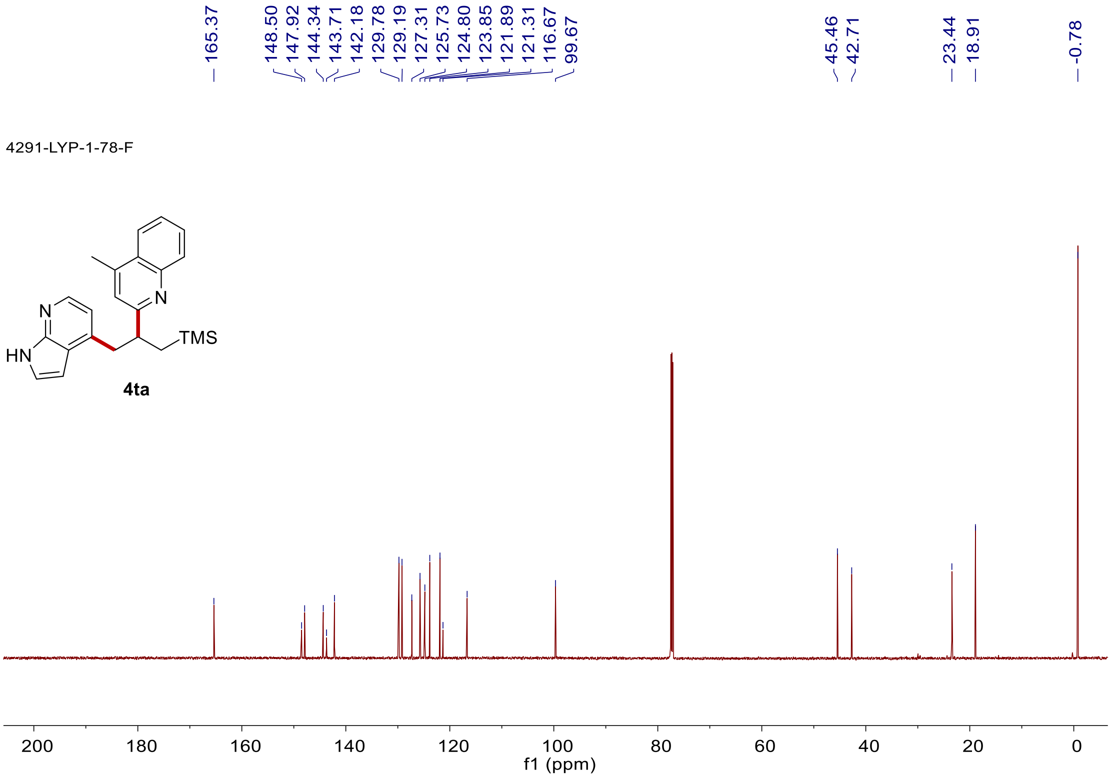

Supplementary Figure 96. ^{13}C NMR spectra of compound **4ta**.

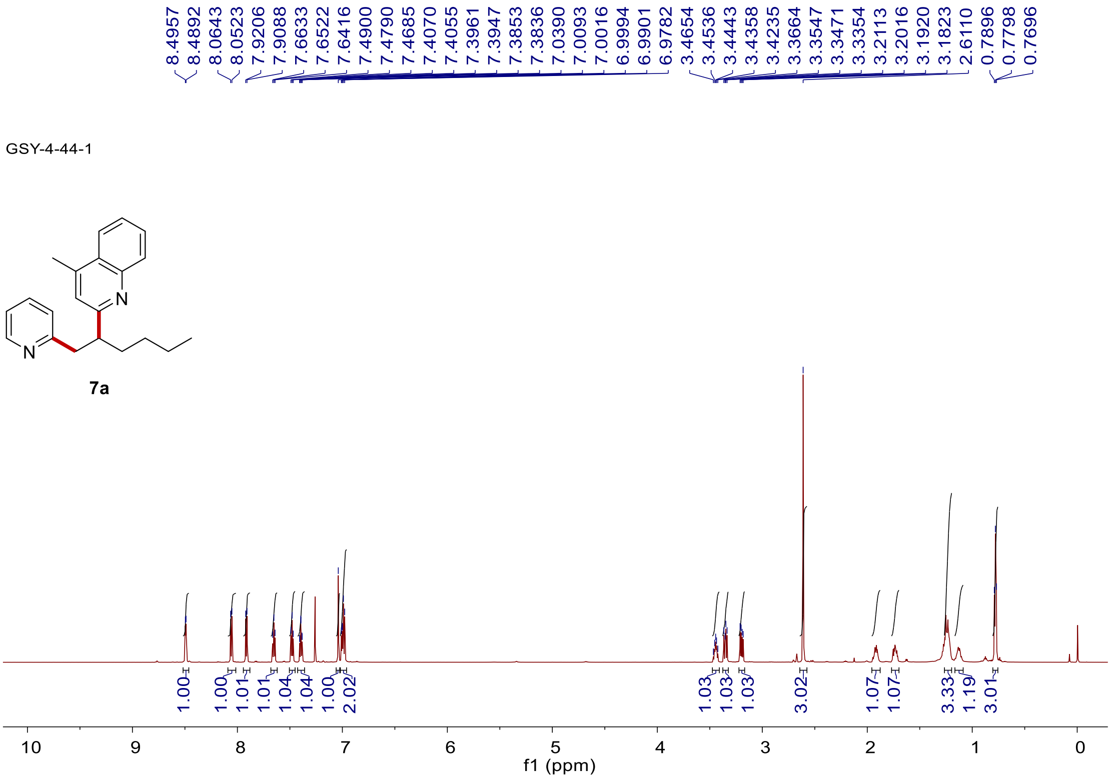

Supplementary Figure 97. ^{1}H NMR spectra of compound **7a**.

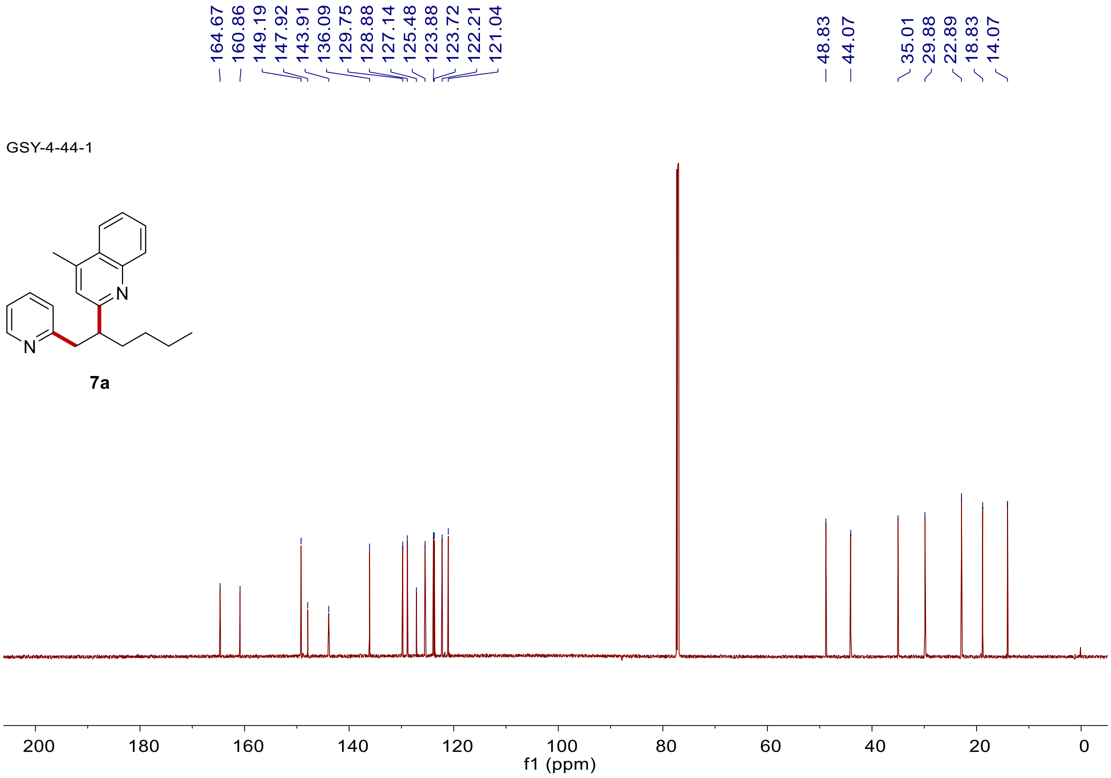

Supplementary Figure 98. ^{13}C NMR spectra of compound **7a**.

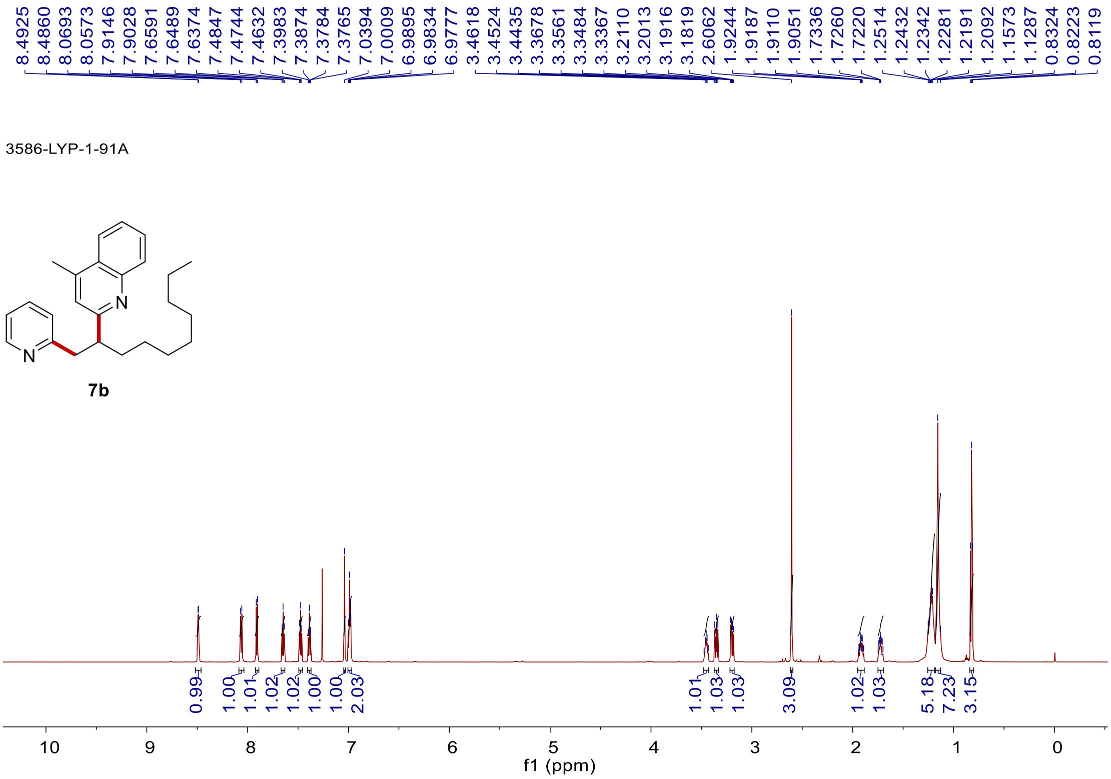

Supplementary Figure 99. ^{1}H NMR spectra of compound **7b**.

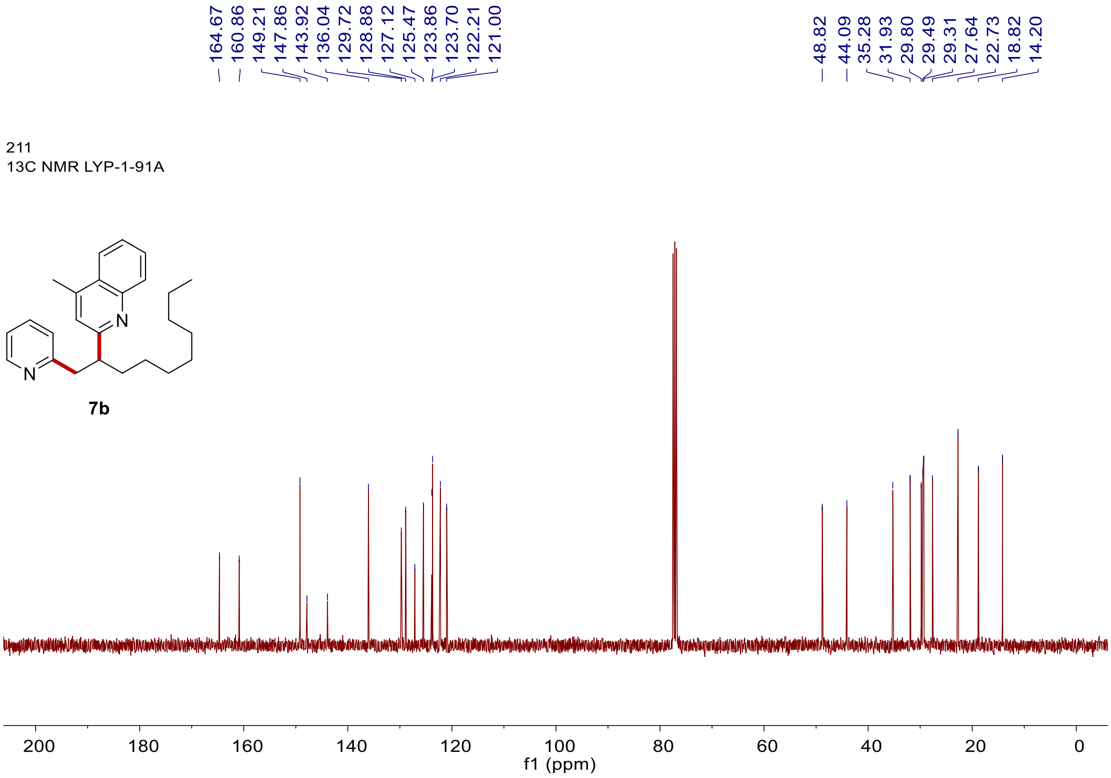

Supplementary Figure 100. ^{13}C NMR spectra of compound **7b**.

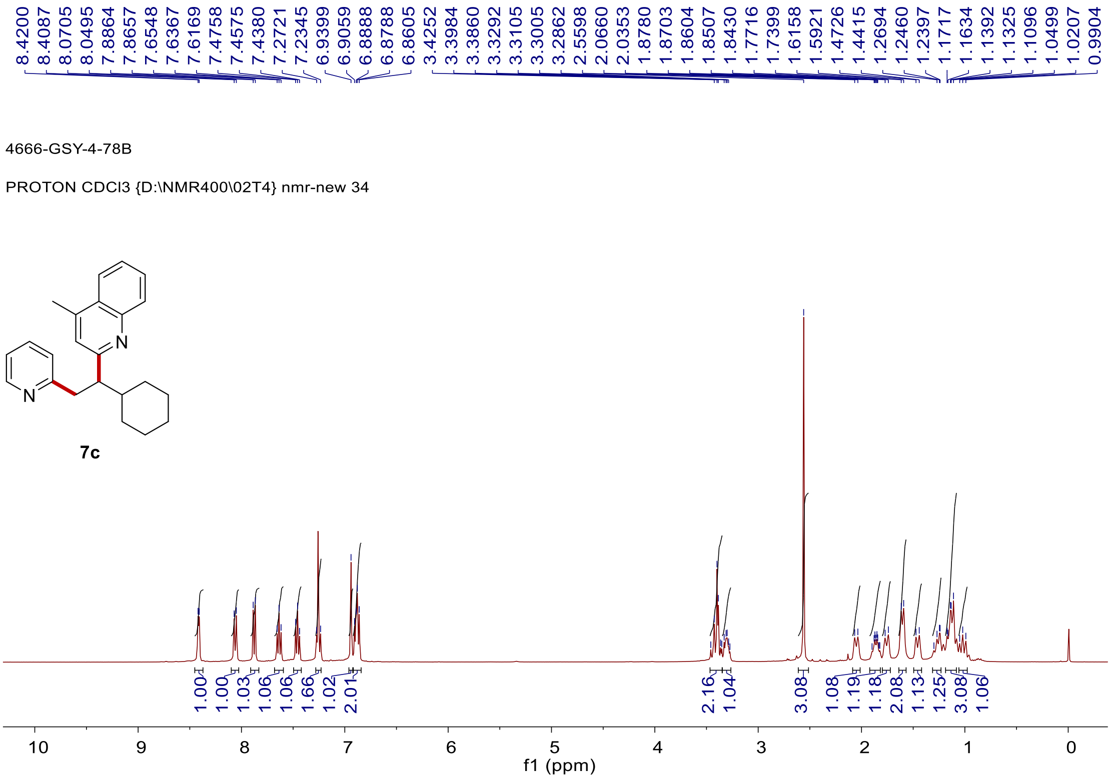

Supplementary Figure 101. ^{1}H NMR spectra of compound **7c**.

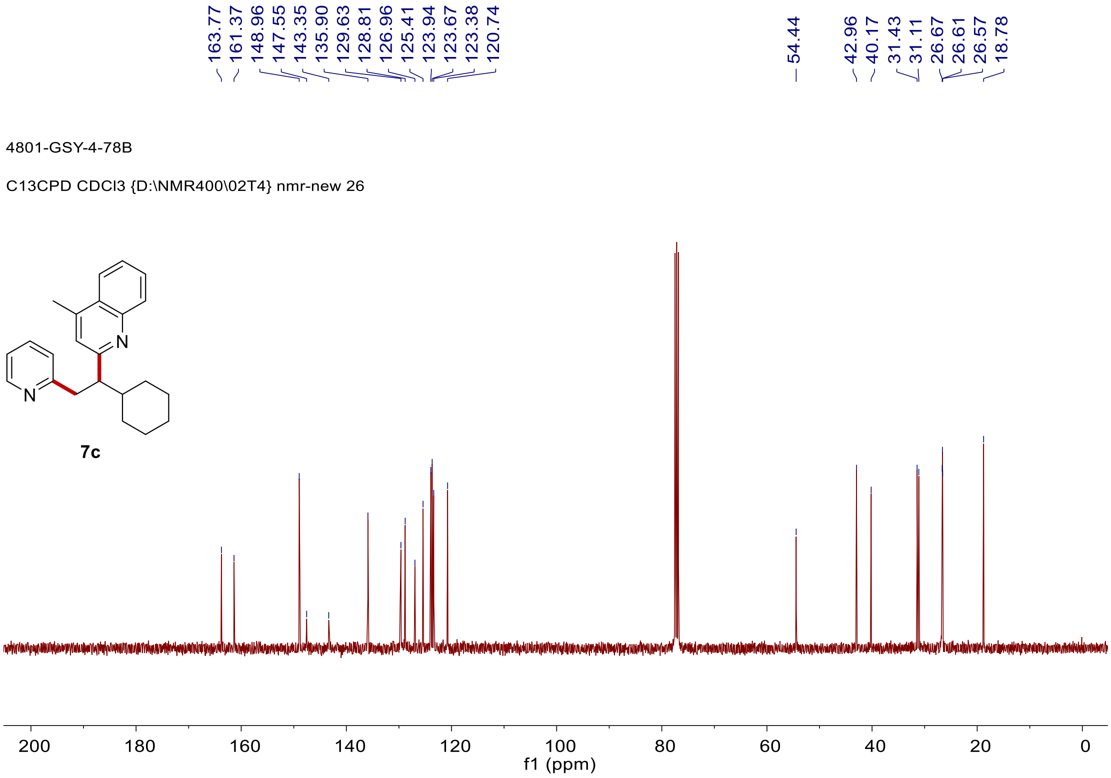

Supplementary Figure 102. ^{13}C NMR spectra of compound **7c**.

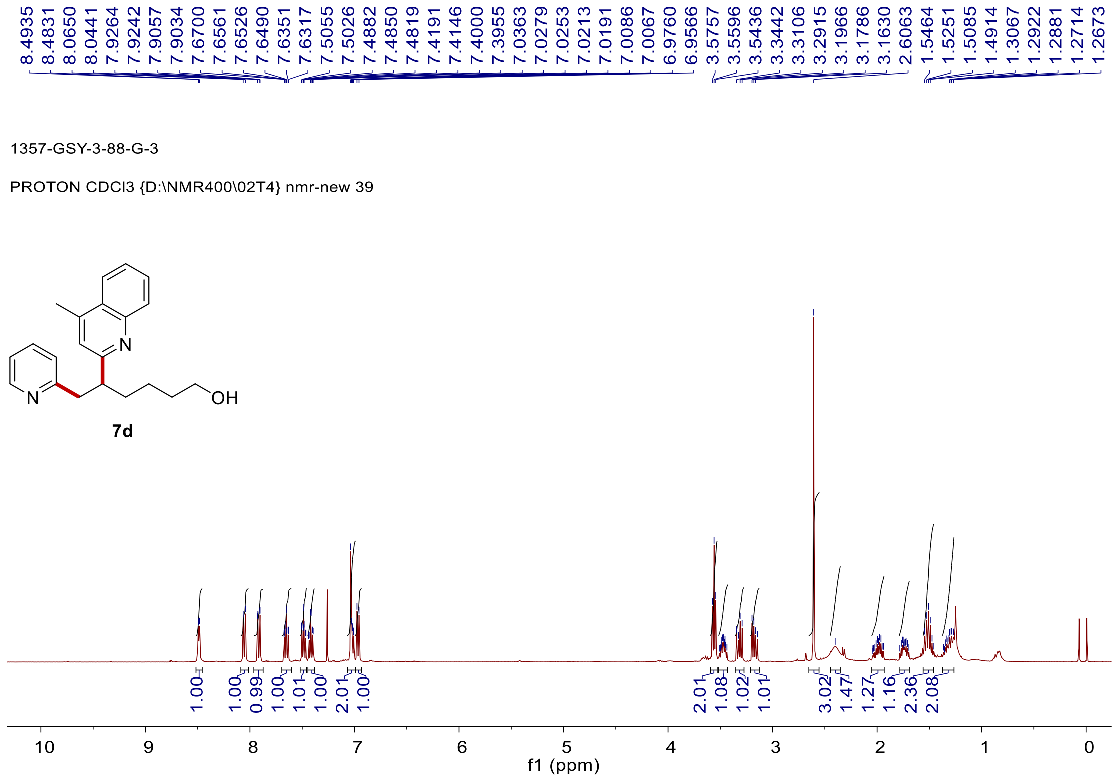

Supplementary Figure 103. ^{1}H NMR spectra of compound **7d**.

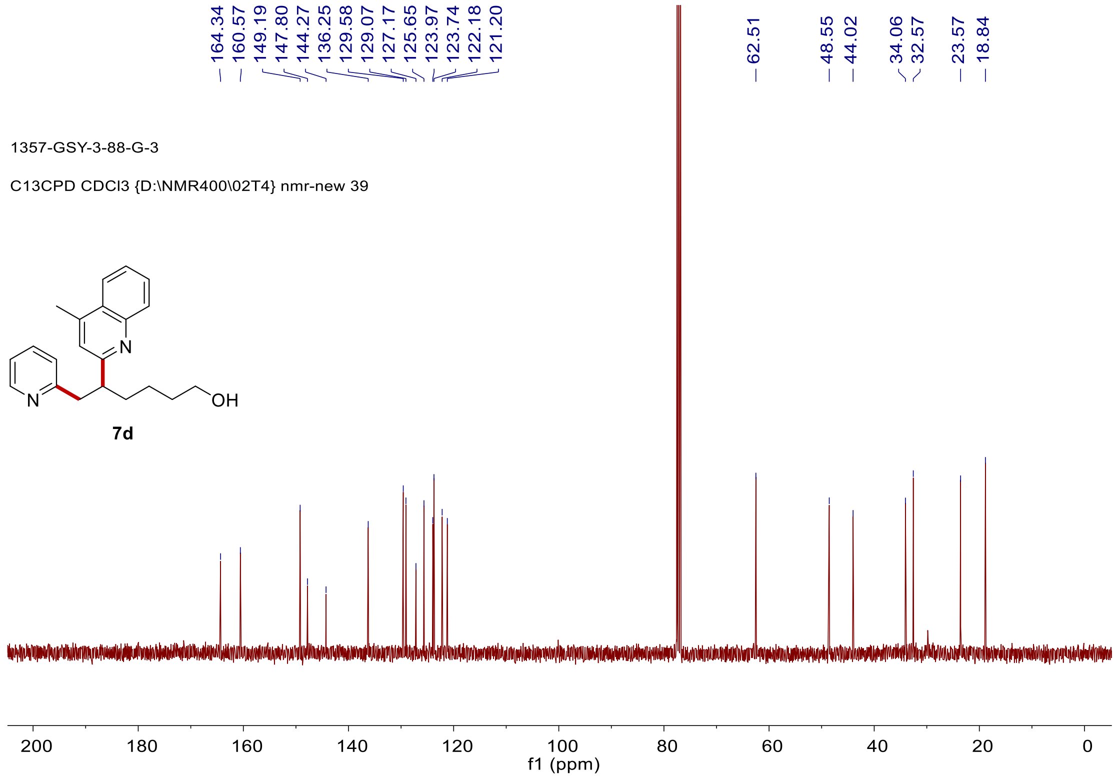

Supplementary Figure 104. ^{13}C NMR spectra of compound **7d**.

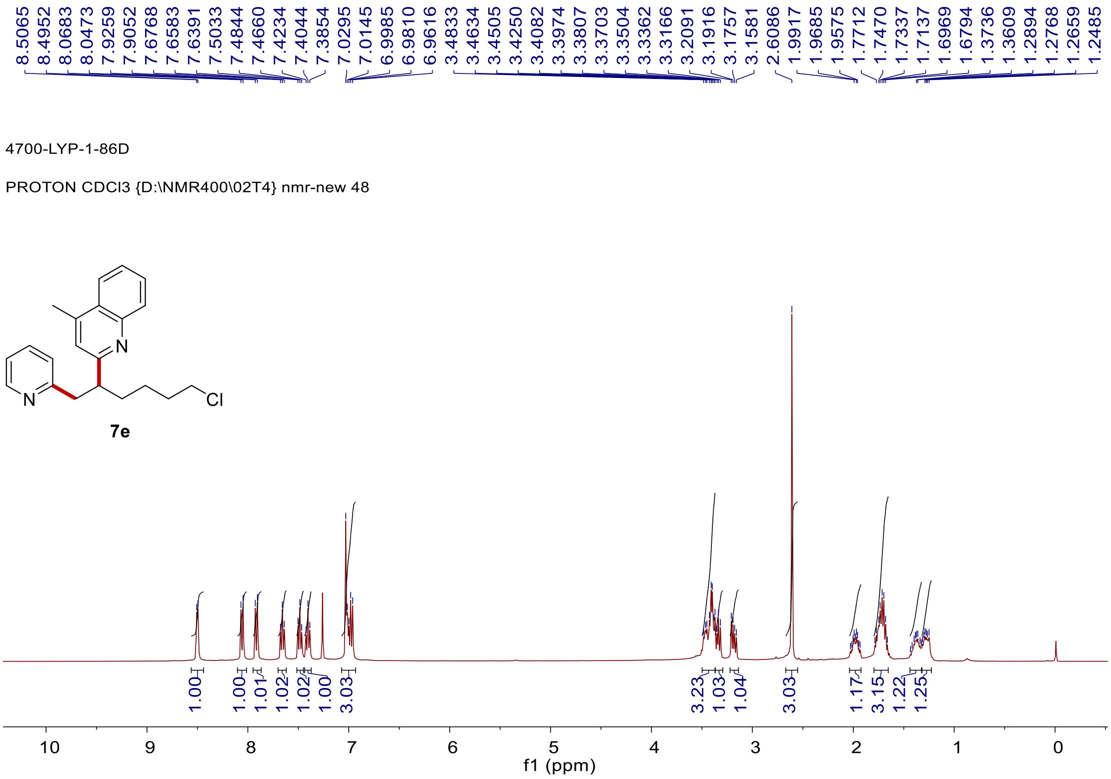

Supplementary Figure 105. ^{1}H NMR spectra of compound **7e**.

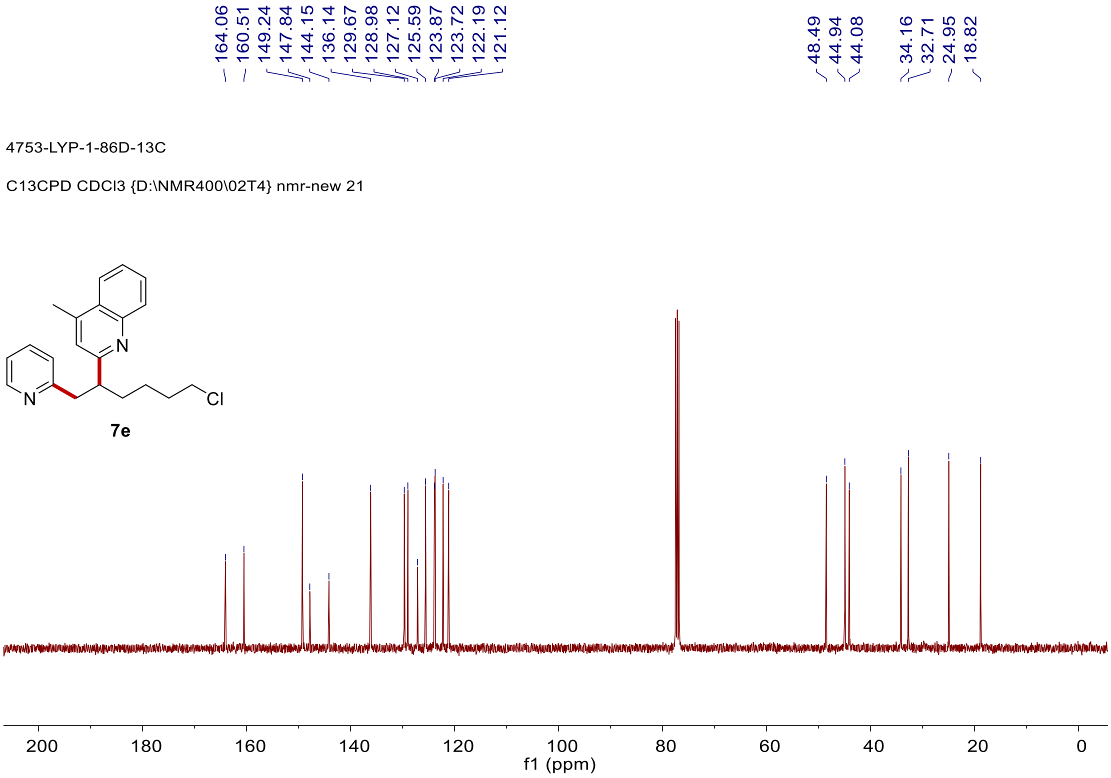

Supplementary Figure 106. ^{13}C NMR spectra of compound **7e**.

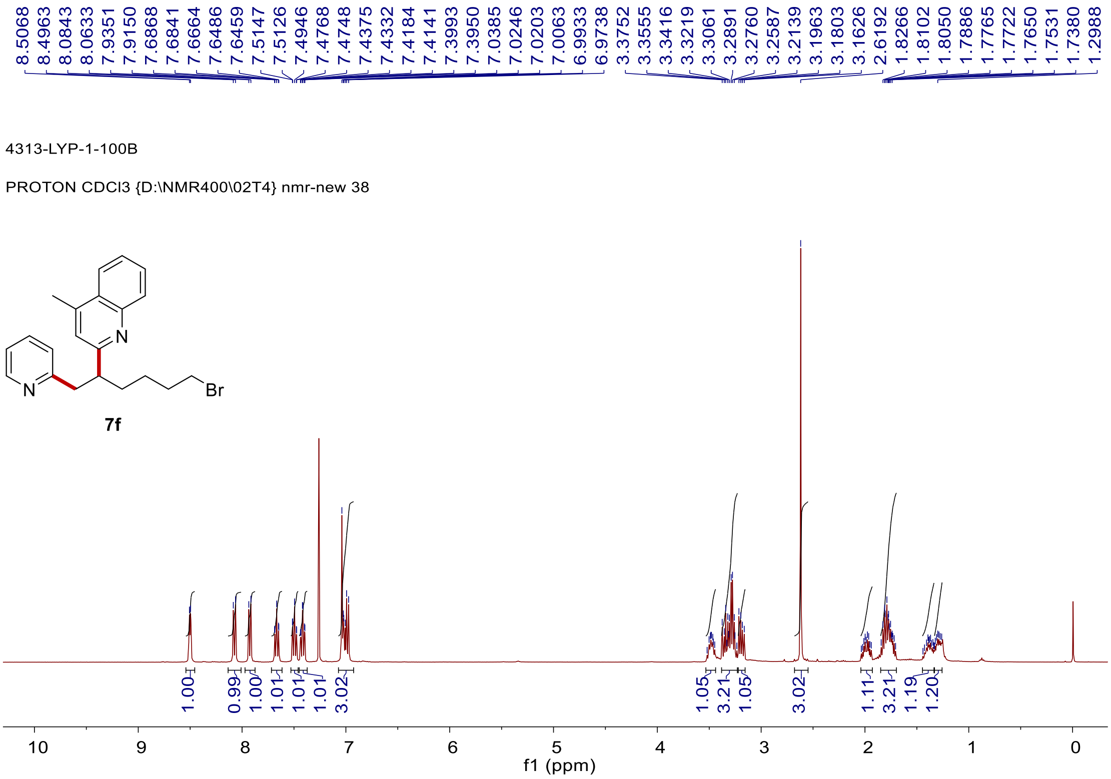

Supplementary Figure 107. ^{1}H NMR spectra of compound **7f**.

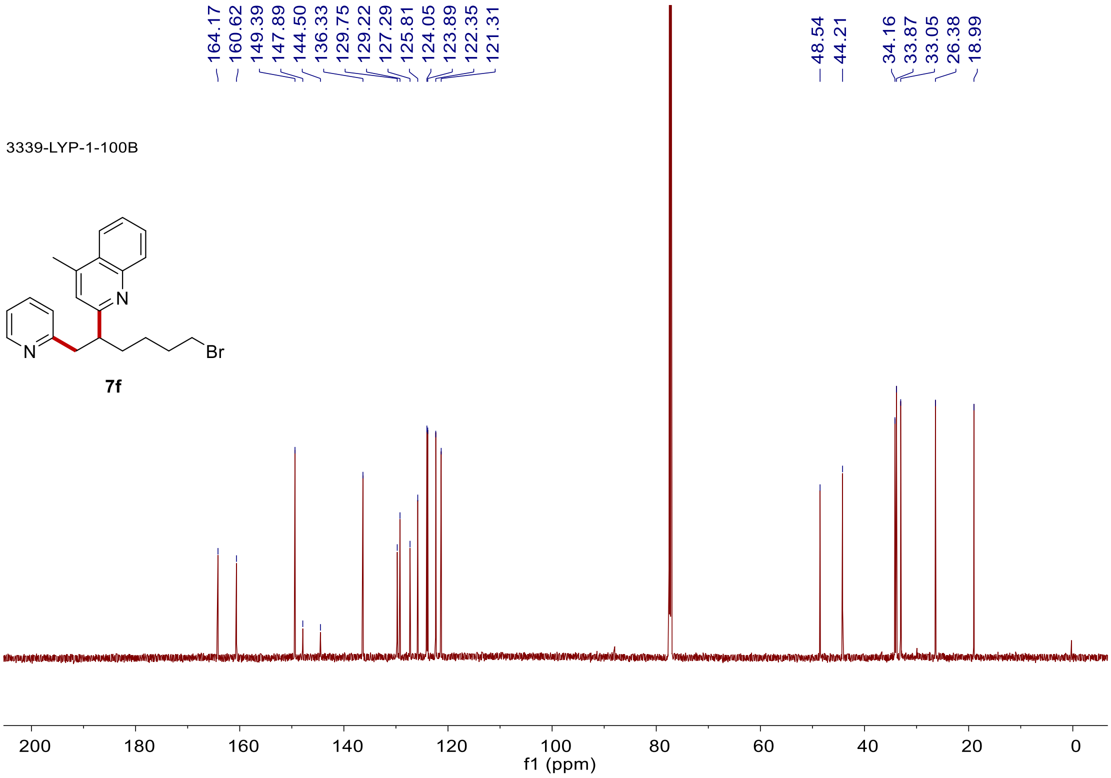

Supplementary Figure 108. ^{13}C NMR spectra of compound **7f**.

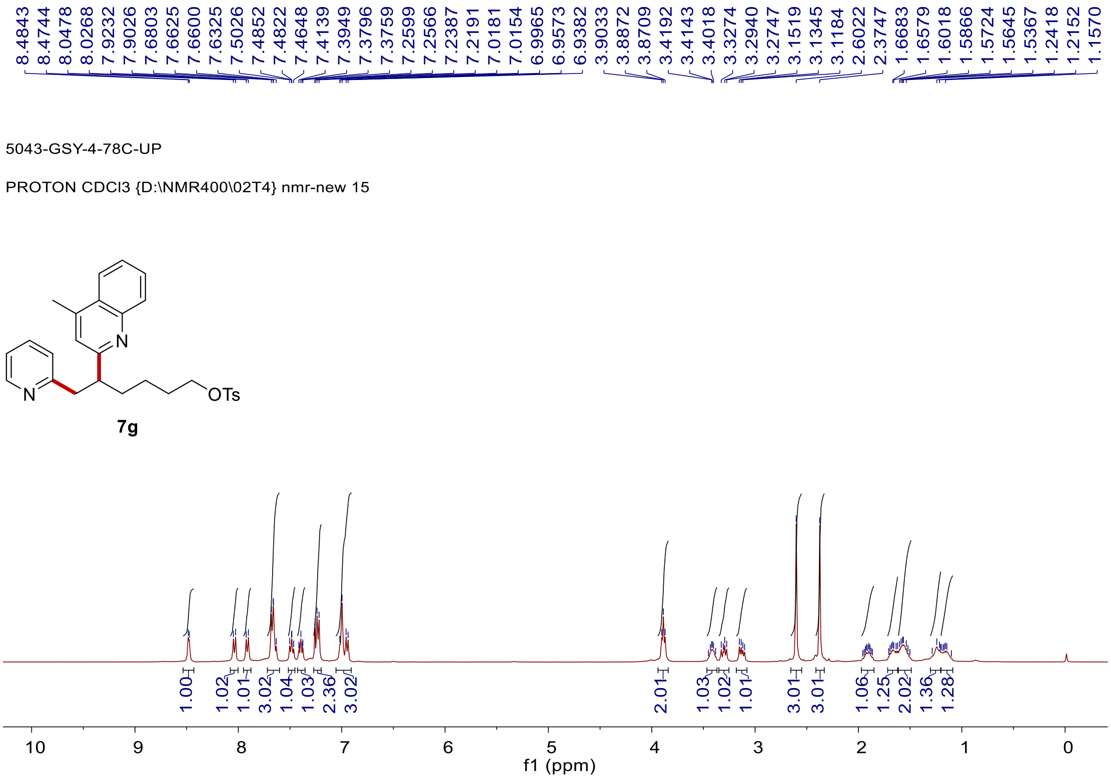

Supplementary Figure 109. ^{1}H NMR spectra of compound **7g**.

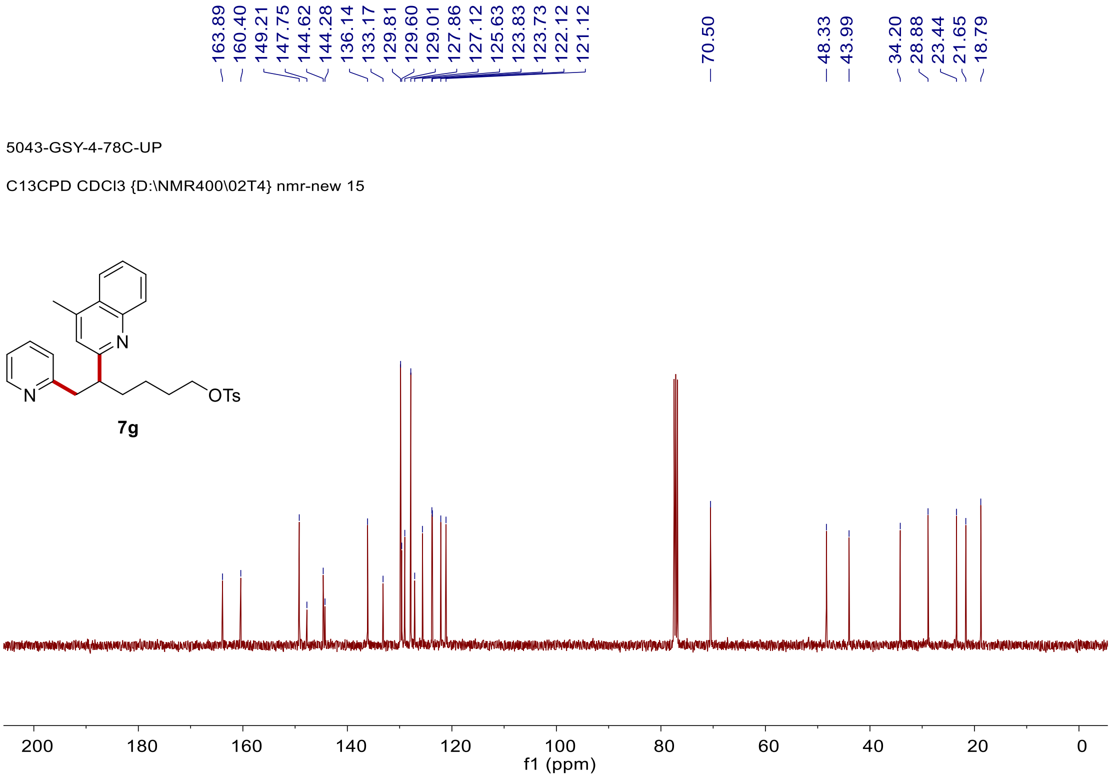

Supplementary Figure 110. ^{13}C NMR spectra of compound **7g**.

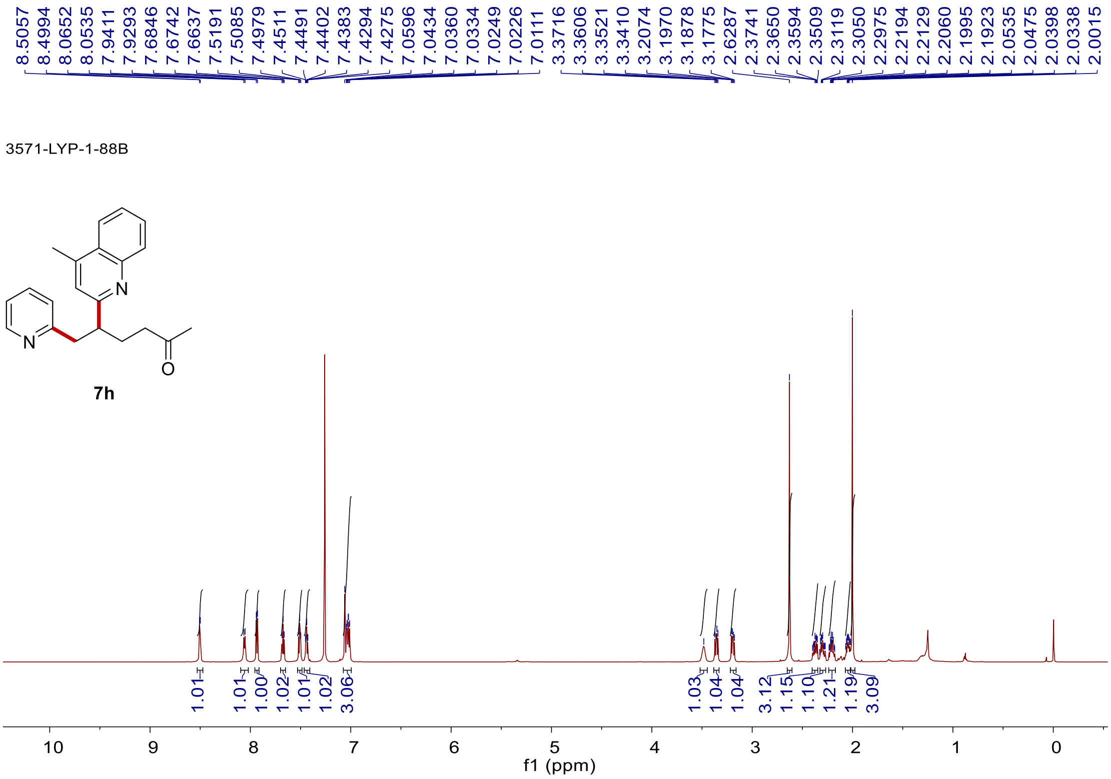

Supplementary Figure 111. ^{1}H NMR spectra of compound **7h**.

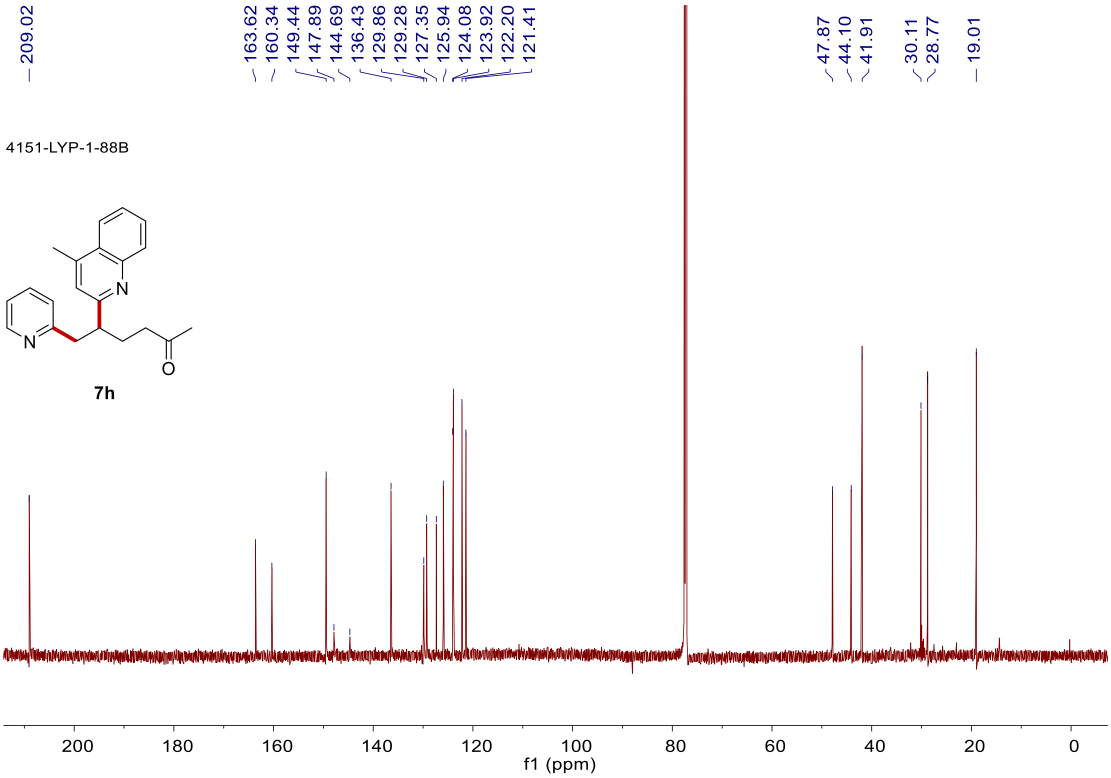

Supplementary Figure 112. ^{13}C NMR spectra of compound **7h**.

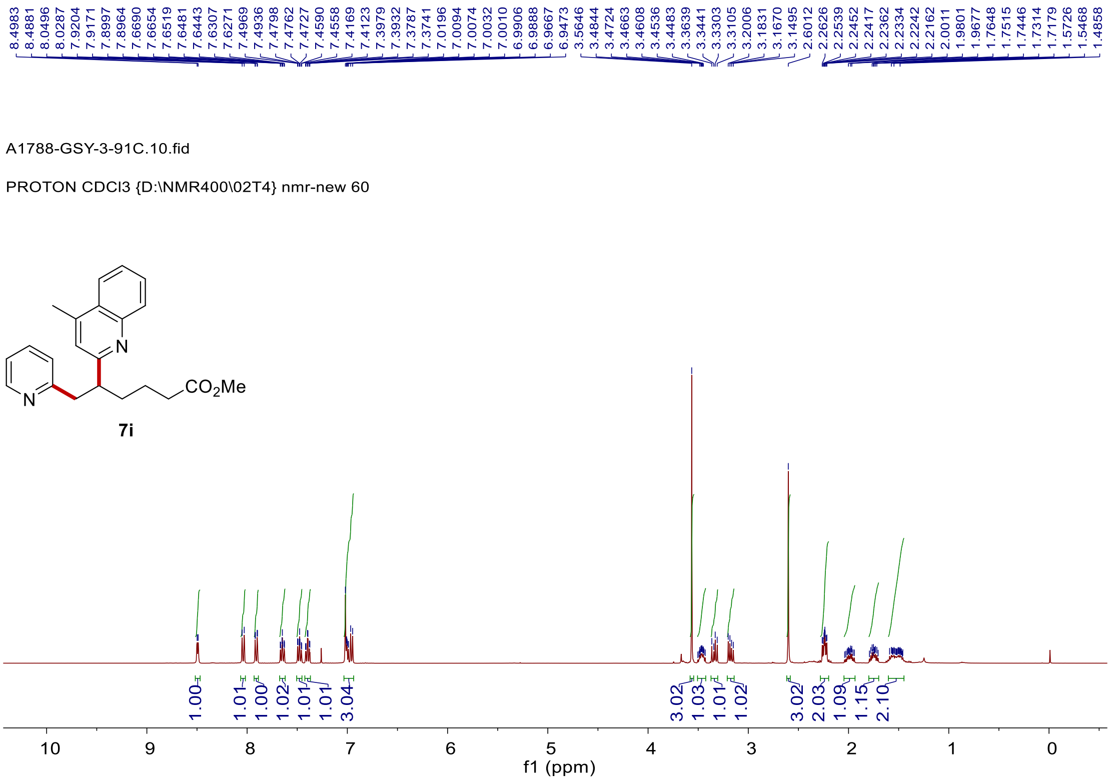

Supplementary Figure 113. ^{1}H NMR spectra of compound **7i**.

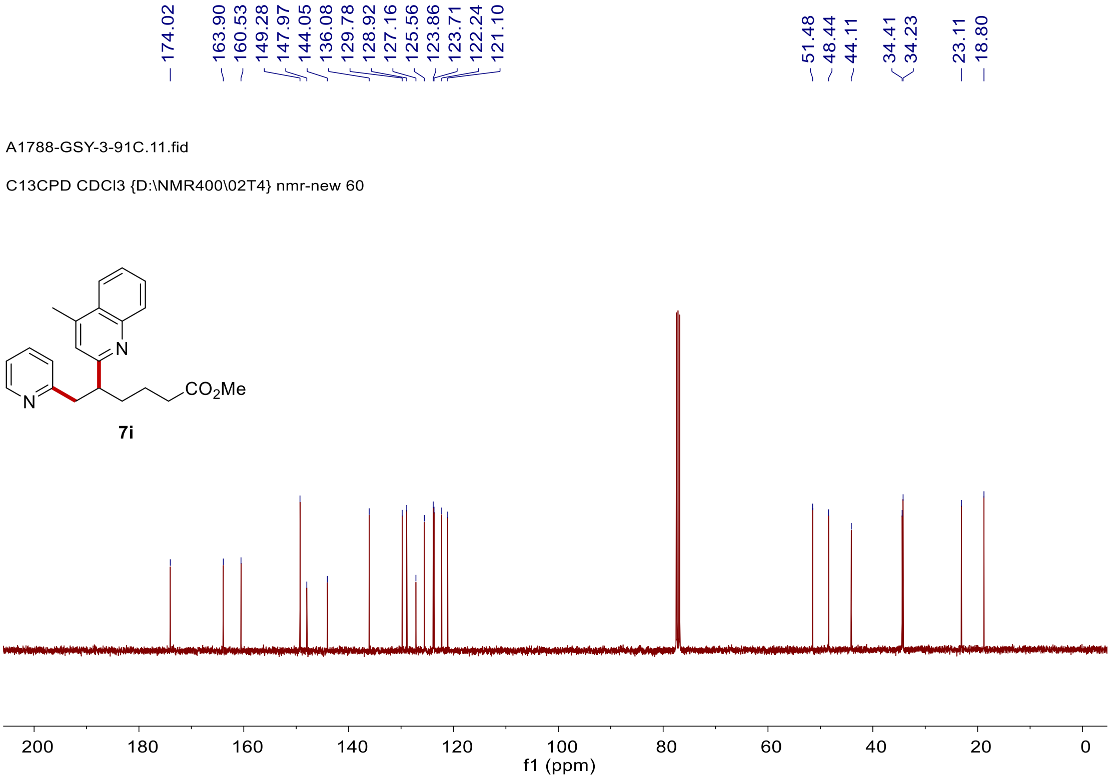

Supplementary Figure 114. ^{13}C NMR spectra of compound **7i**.

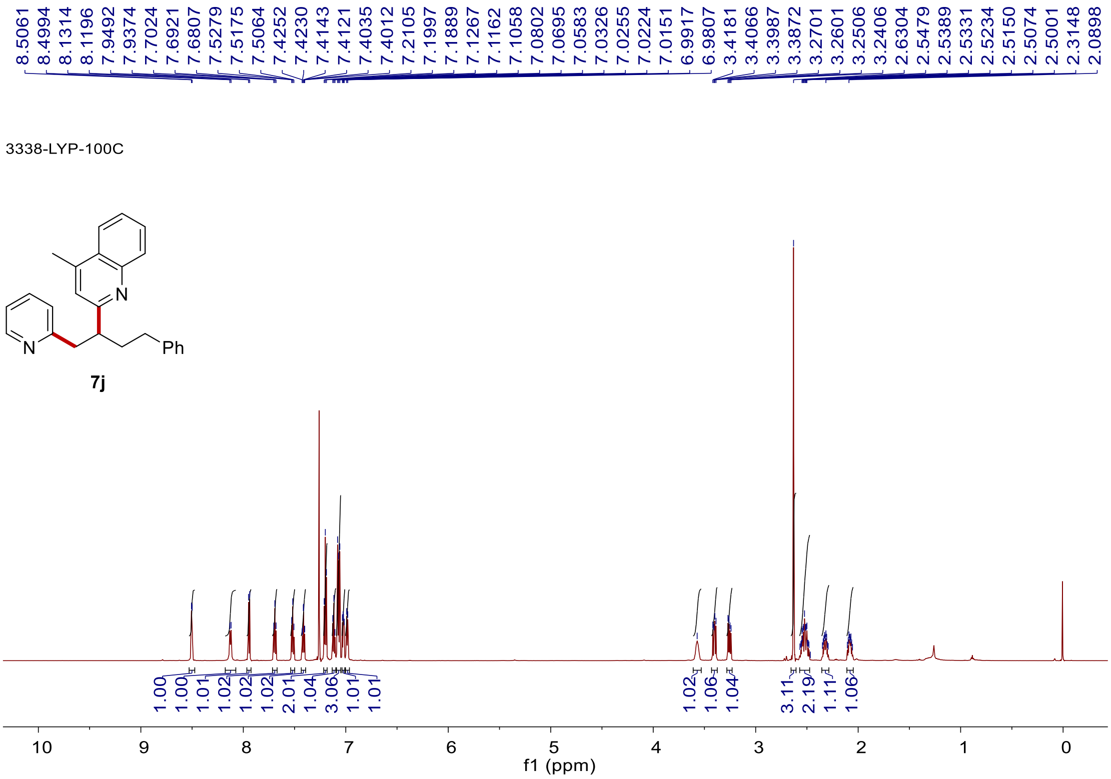

Supplementary Figure 115. ^{1}H NMR spectra of compound **7j**.

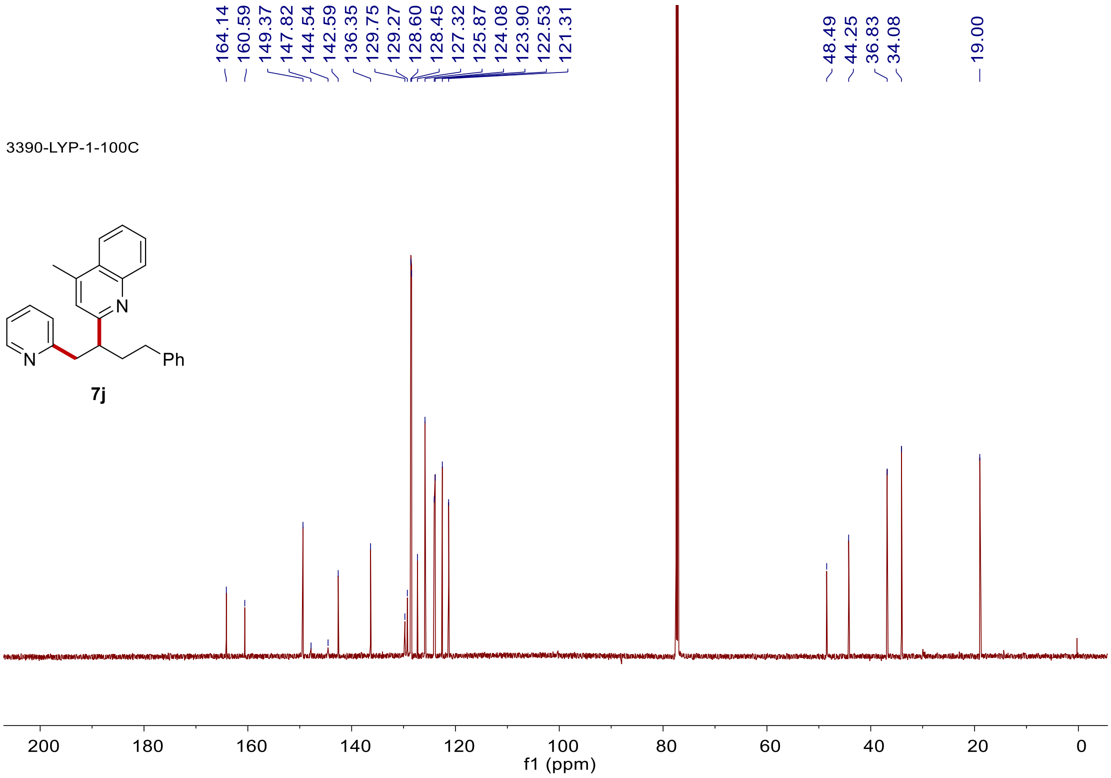

Supplementary Figure 116. ^{13}C NMR spectra of compound **7j**.

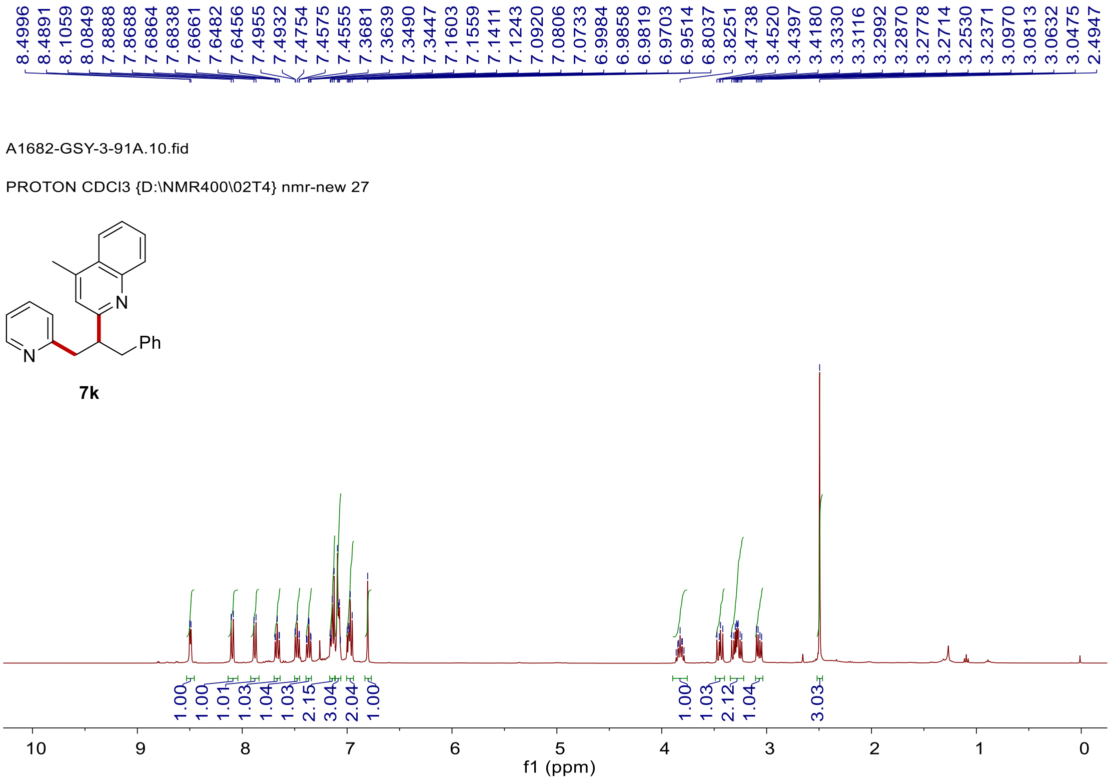

Supplementary Figure 117. ^{1}H NMR spectra of compound **7k**.

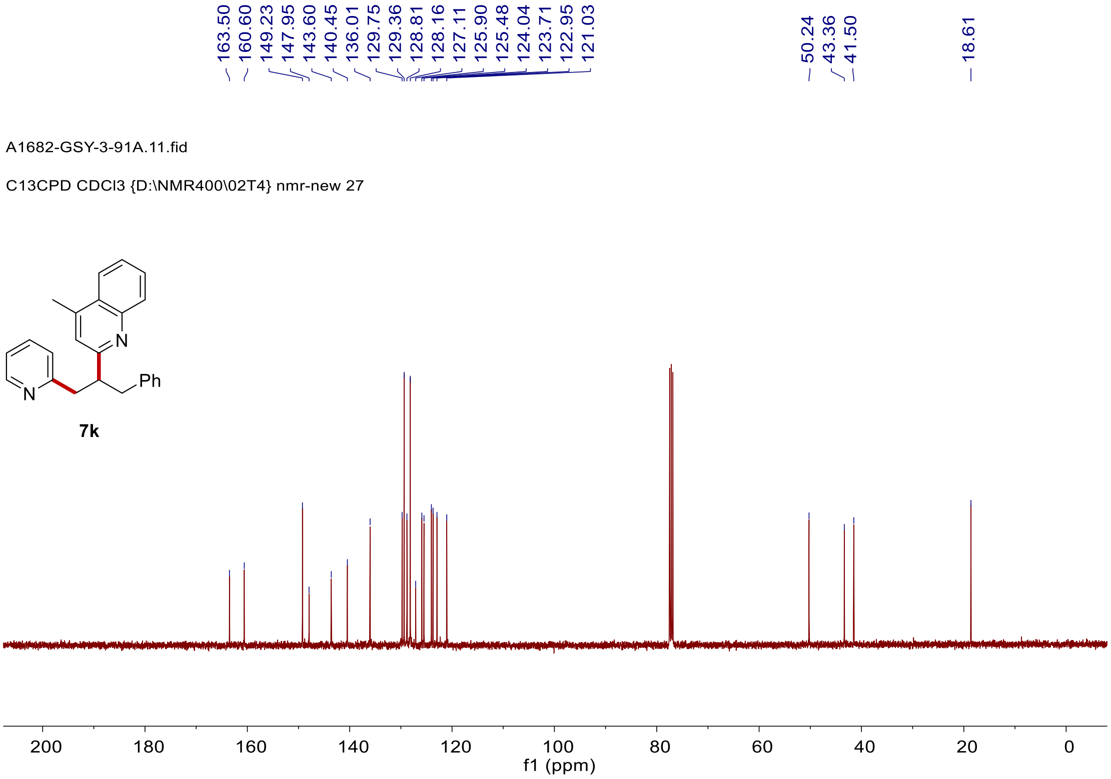

Supplementary Figure 118. ^{13}C NMR spectra of compound **7k**.

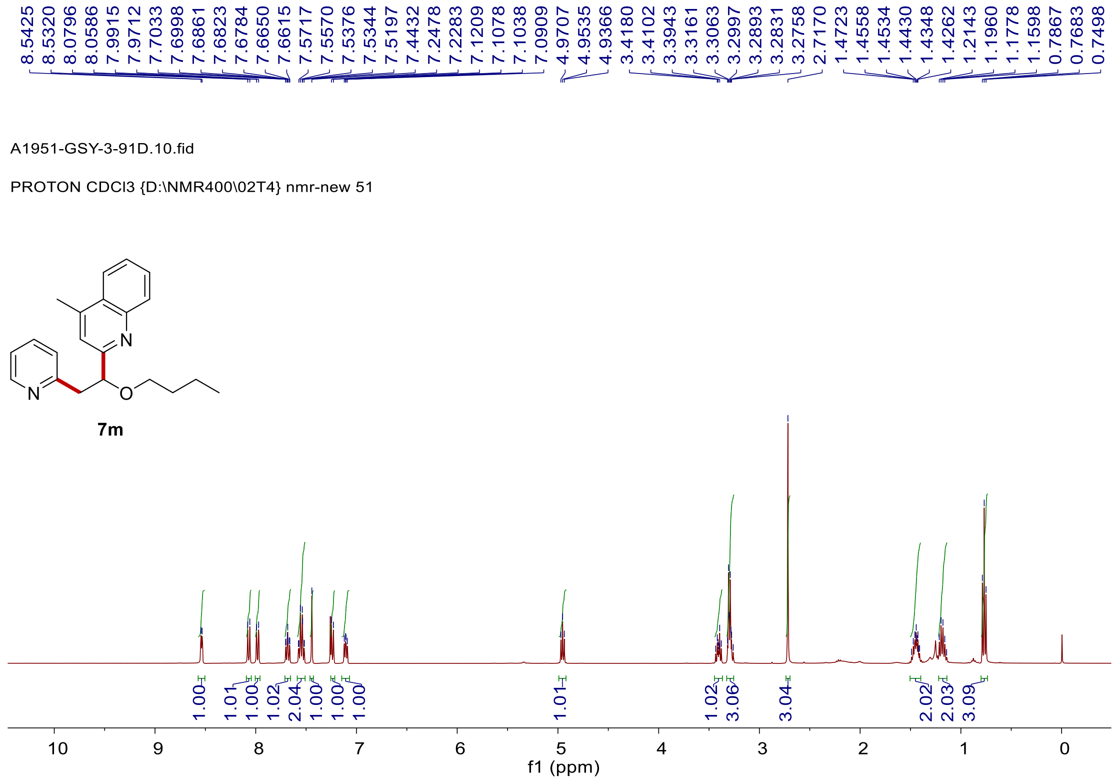

Supplementary Figure 119. ^{1}H NMR spectra of compound **7m**.

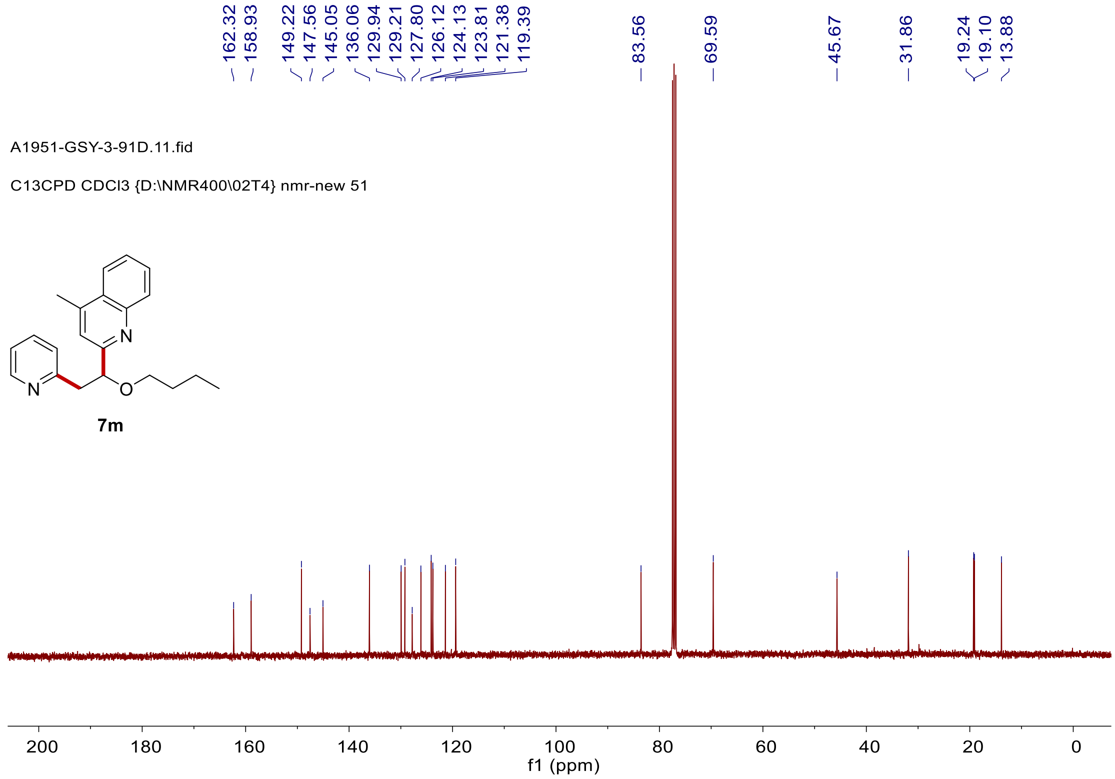

Supplementary Figure 120. ^{13}C NMR spectra of compound **7m**.

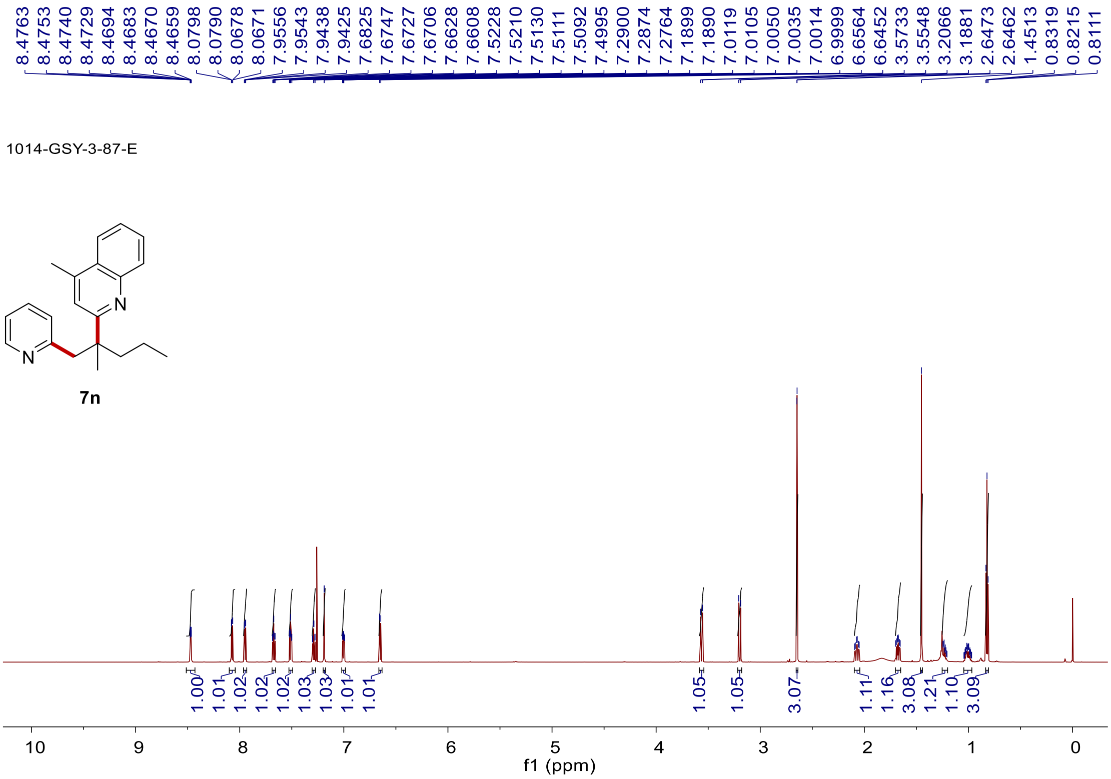

Supplementary Figure 121. ^{1}H NMR spectra of compound **7n**.

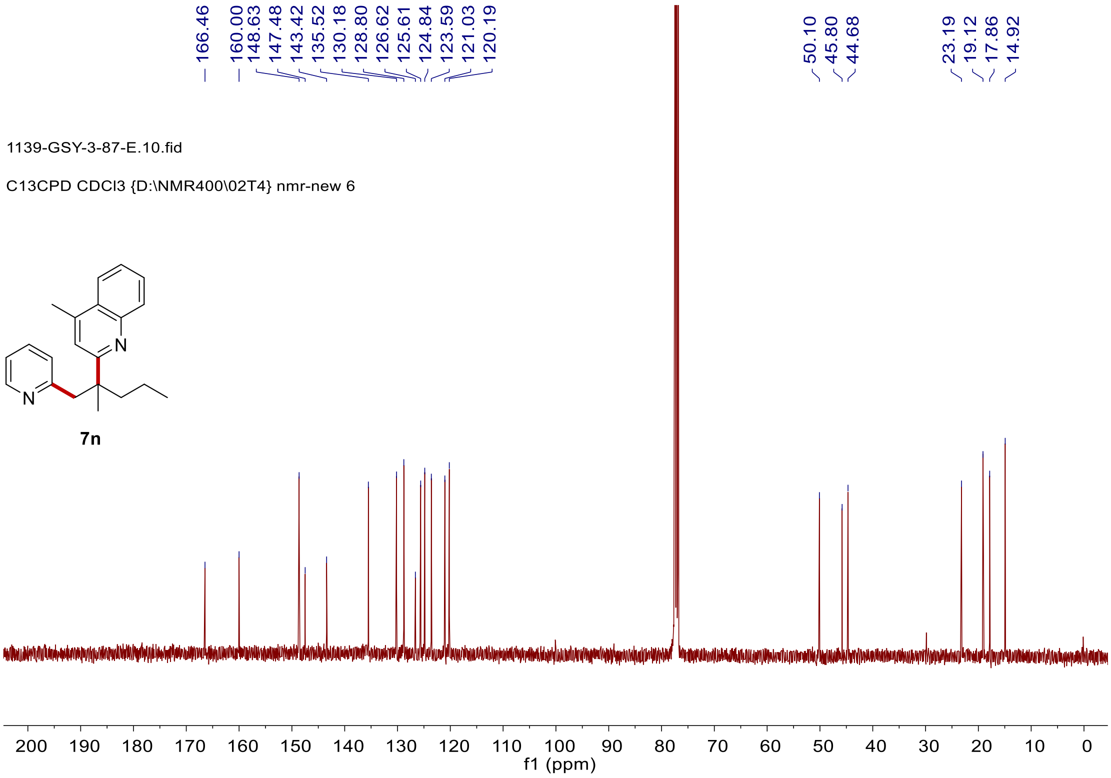

Supplementary Figure 122. ^{13}C NMR spectra of compound **7n**.

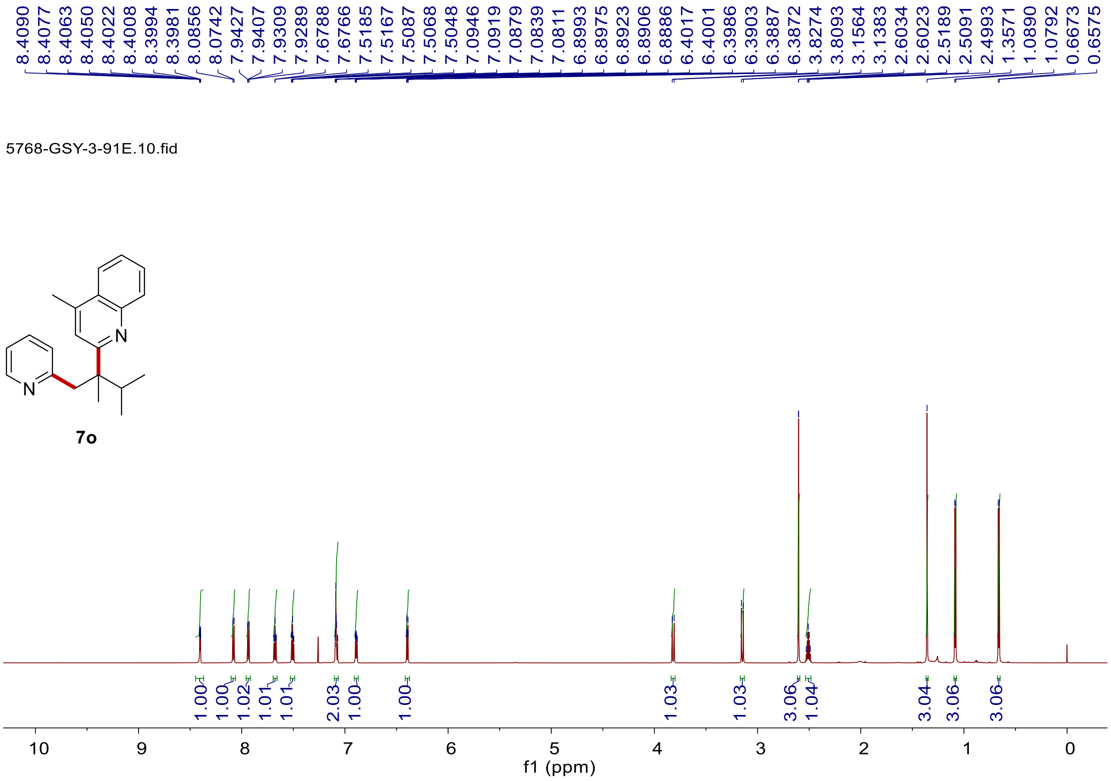

Supplementary Figure 123. ^{1}H NMR spectra of compound **7o**.

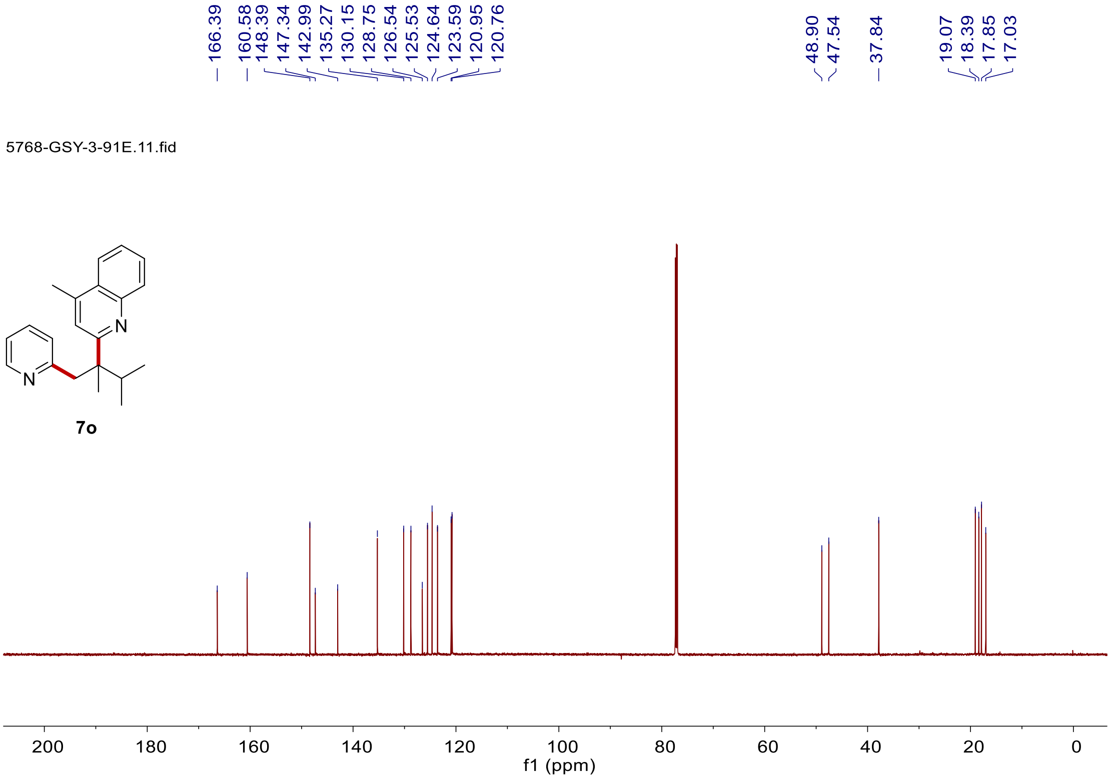

Supplementary Figure 124. ^{13}C NMR spectra of compound **7o**.

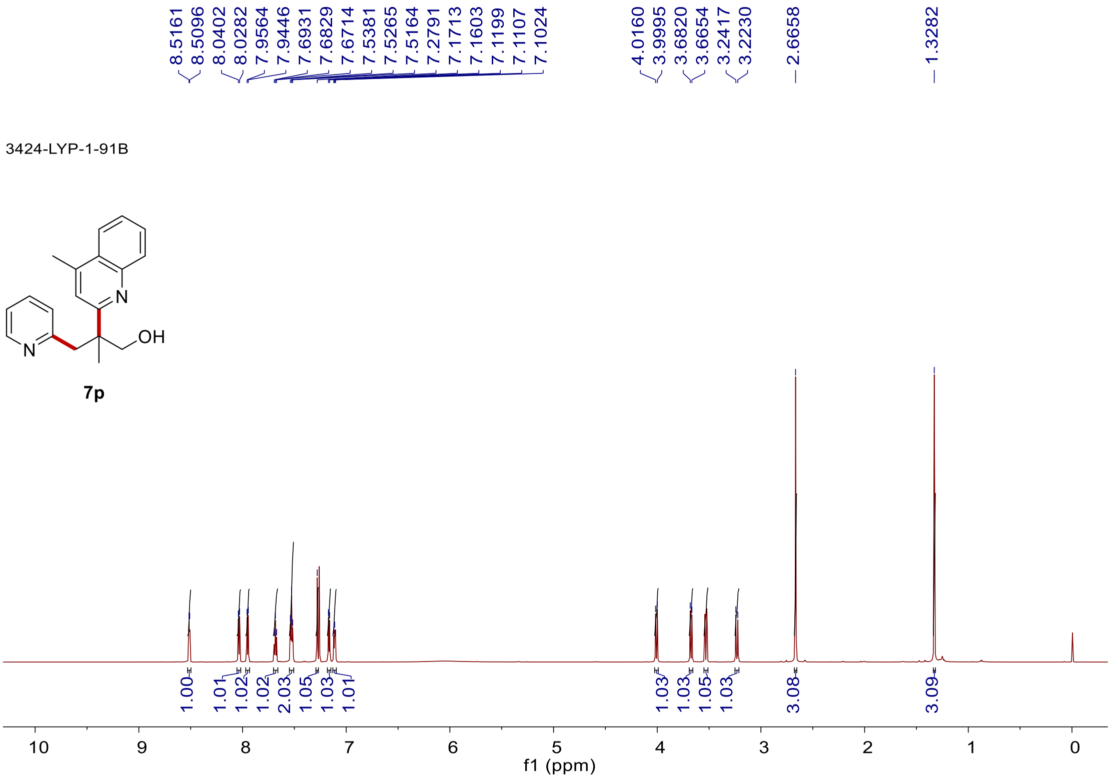

Supplementary Figure 125. ^{1}H NMR spectra of compound **7p**.

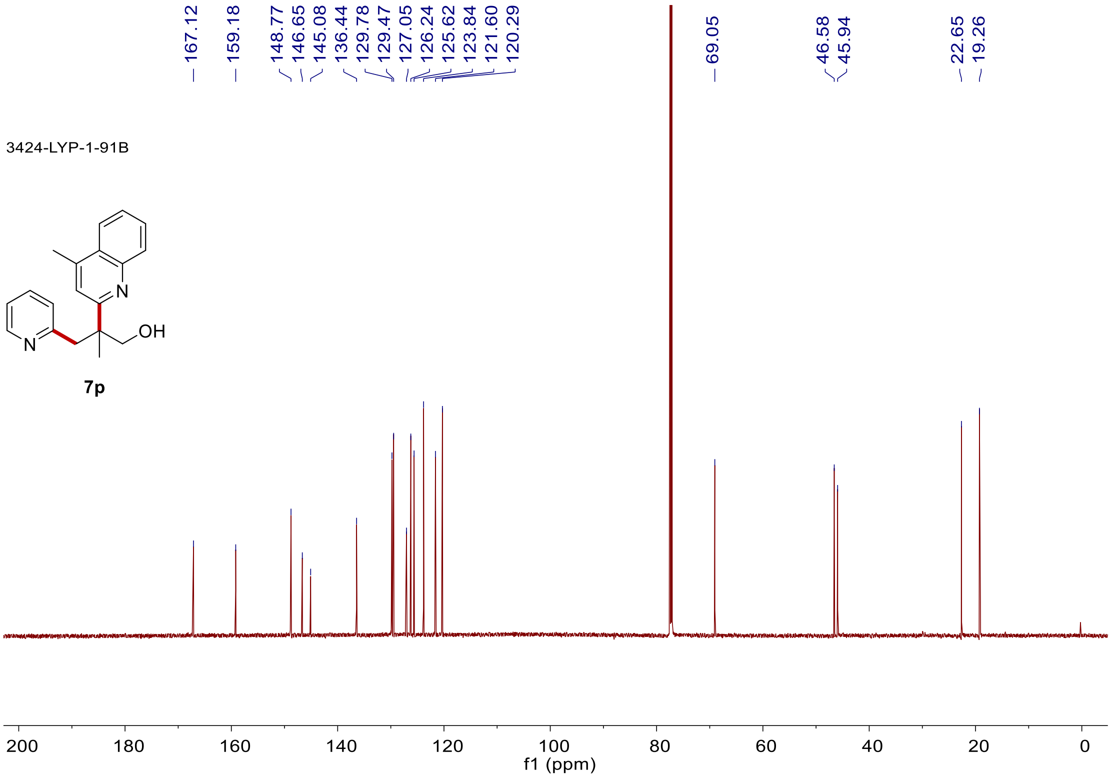

Supplementary Figure 126. ^{13}C NMR spectra of compound **7p**.

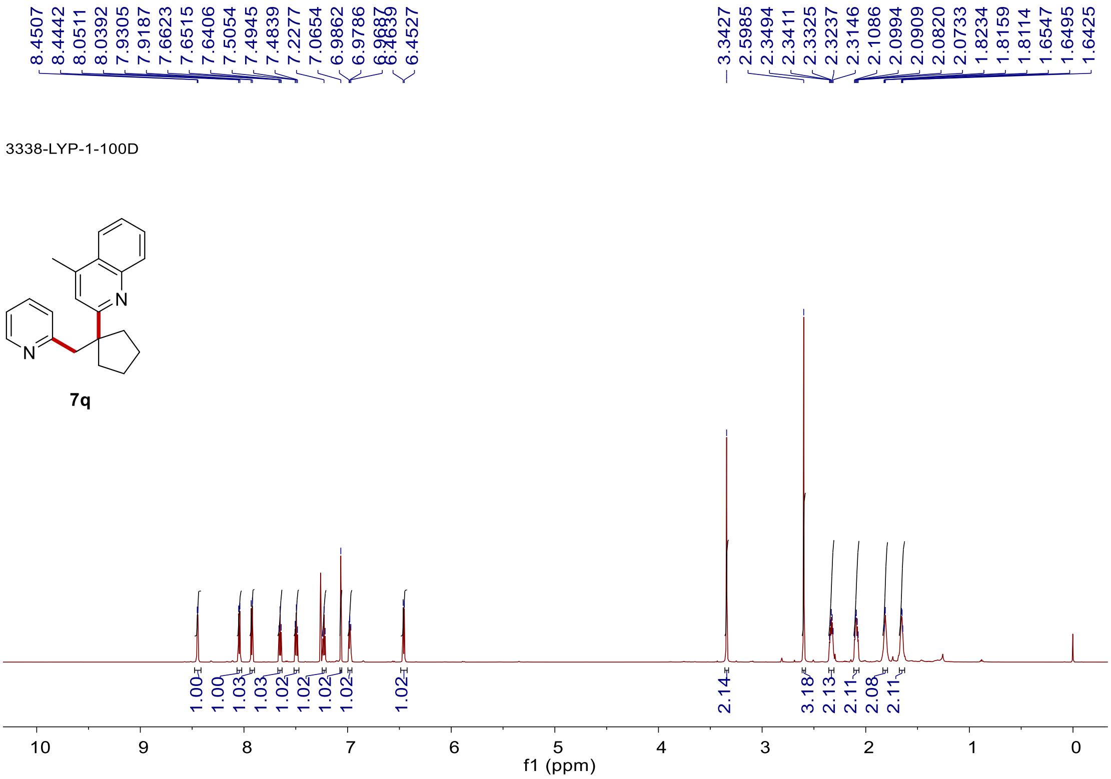

Supplementary Figure 127. ^{1}H NMR spectra of compound **7q**.

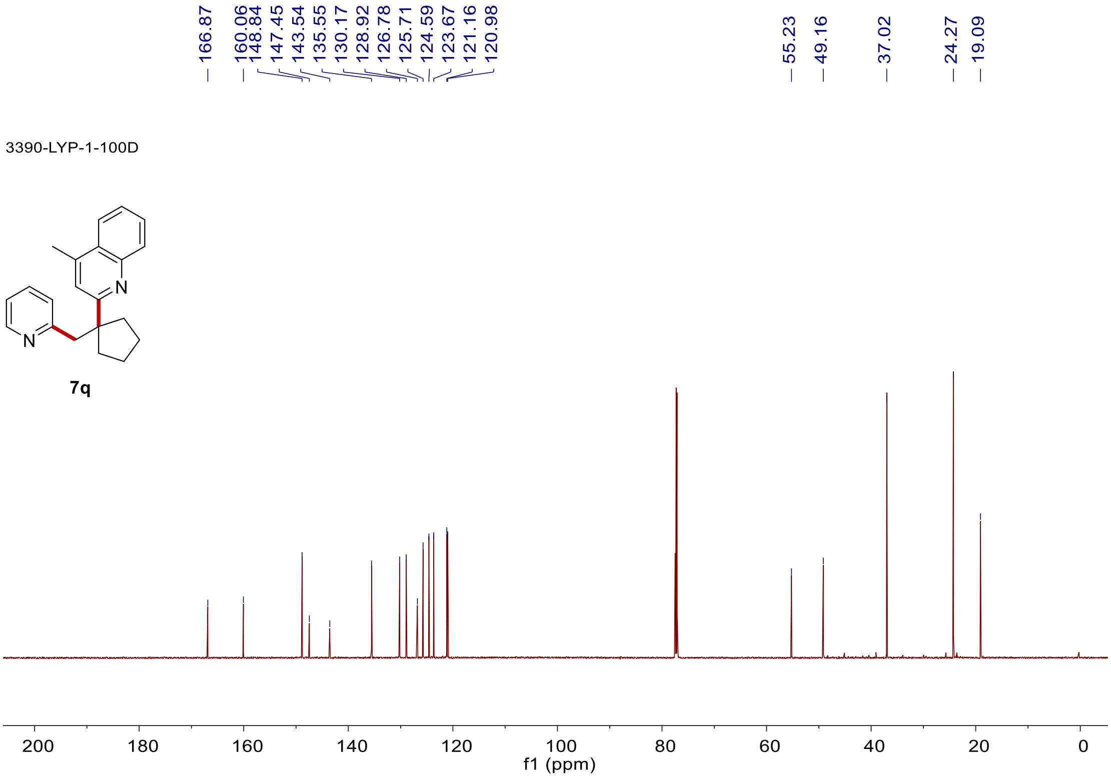

Supplementary Figure 128. ^{13}C NMR spectra of compound **7q**.

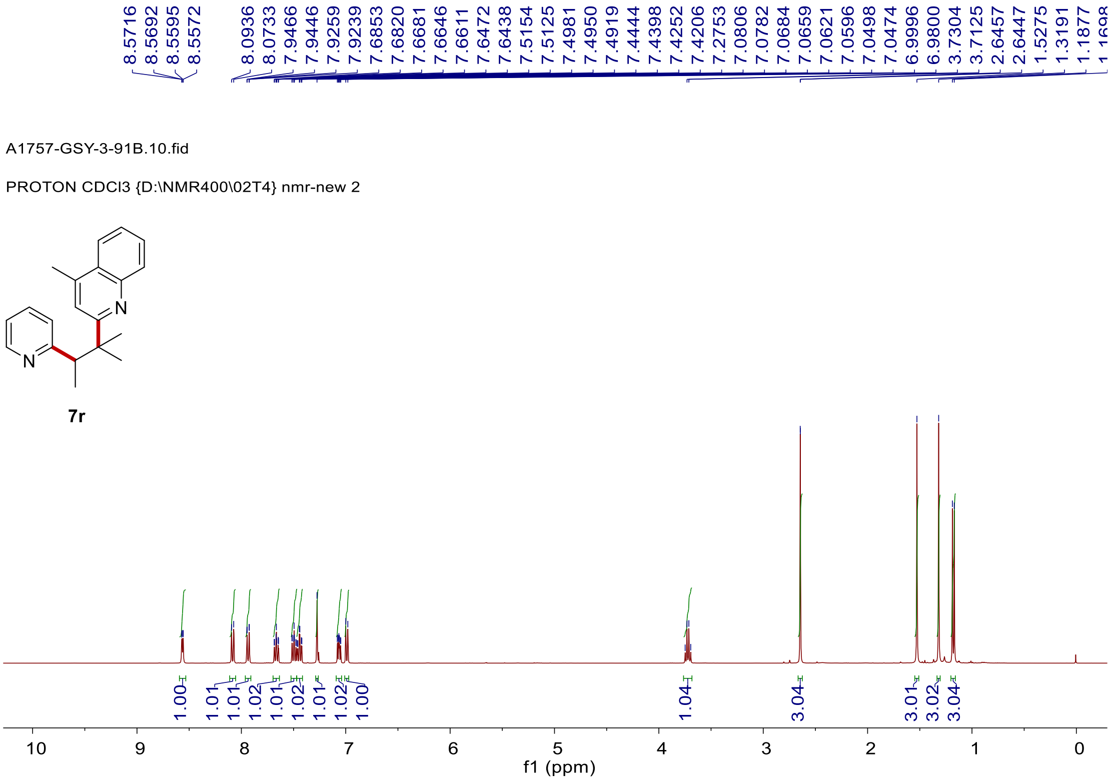

Supplementary Figure 129. ^{1}H NMR spectra of compound **7r**.

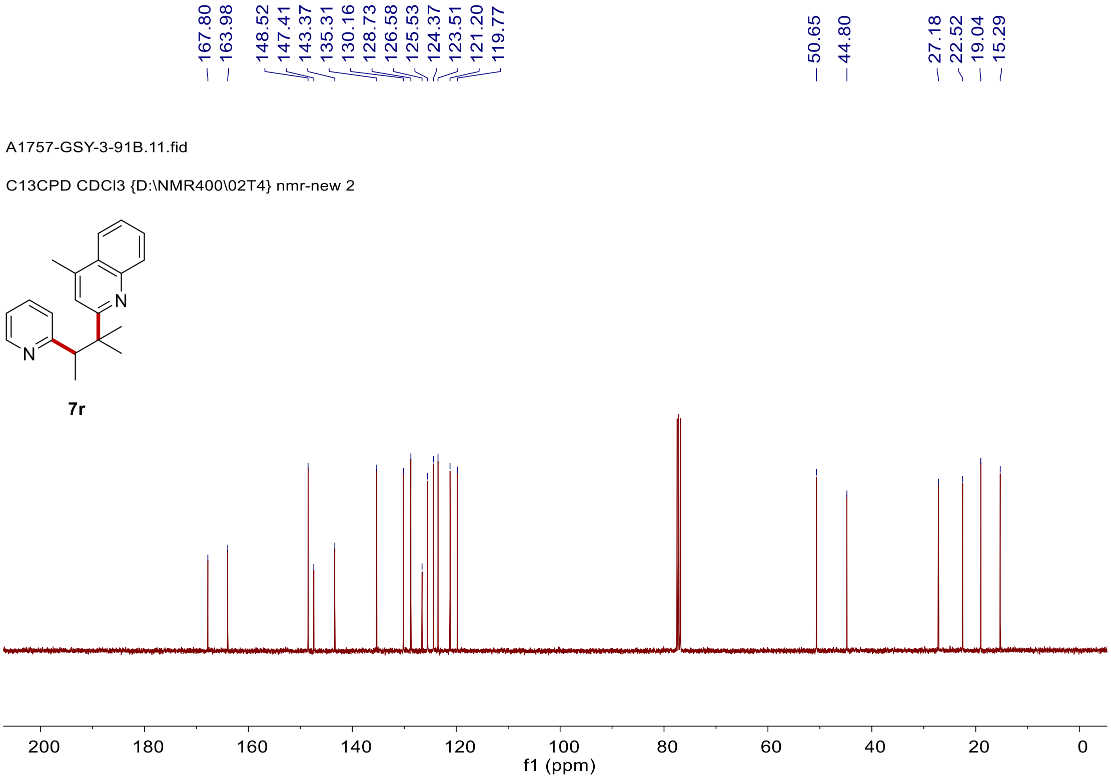

Supplementary Figure 130. ^{13}C NMR spectra of compound **7r**.

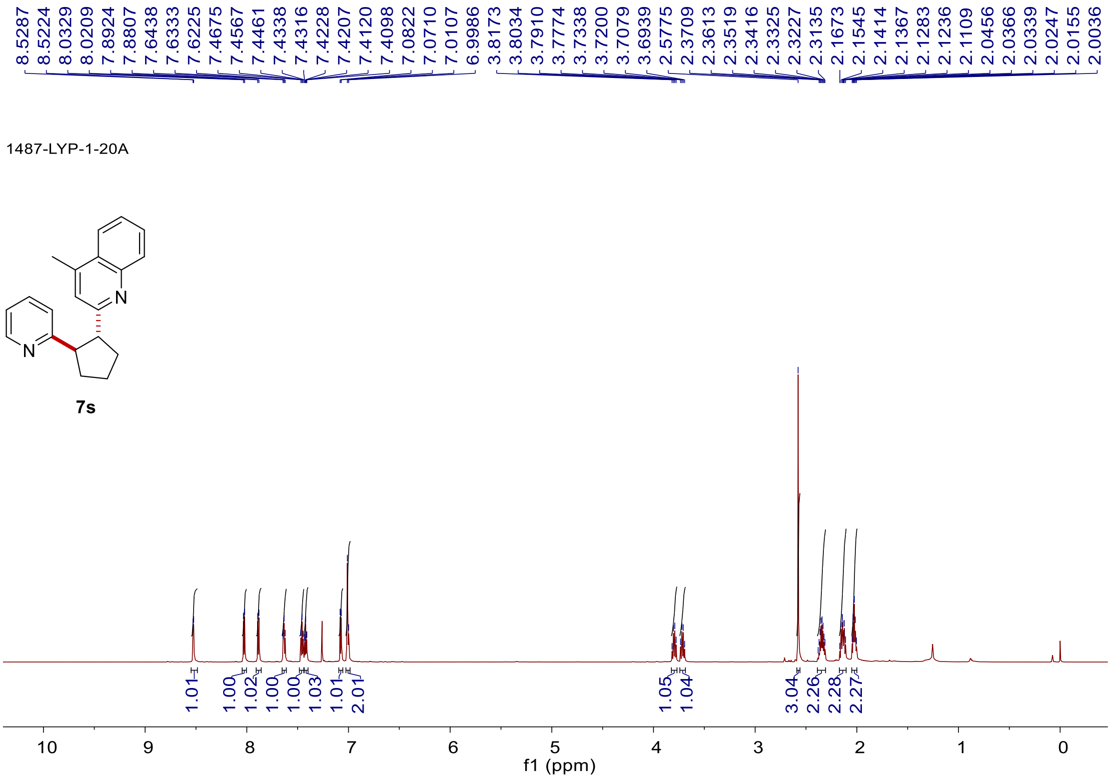

Supplementary Figure 131. ^{1}H NMR spectra of compound **7s**.

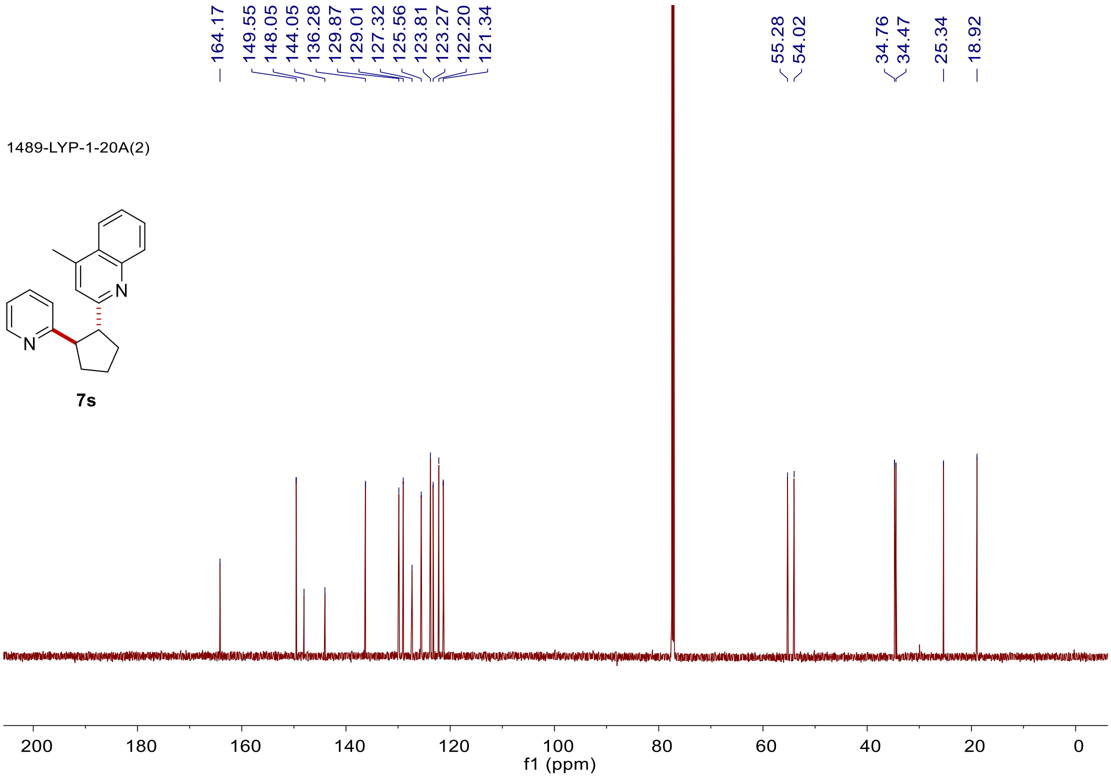

Supplementary Figure 132. ^{13}C NMR spectra of compound **7s**.

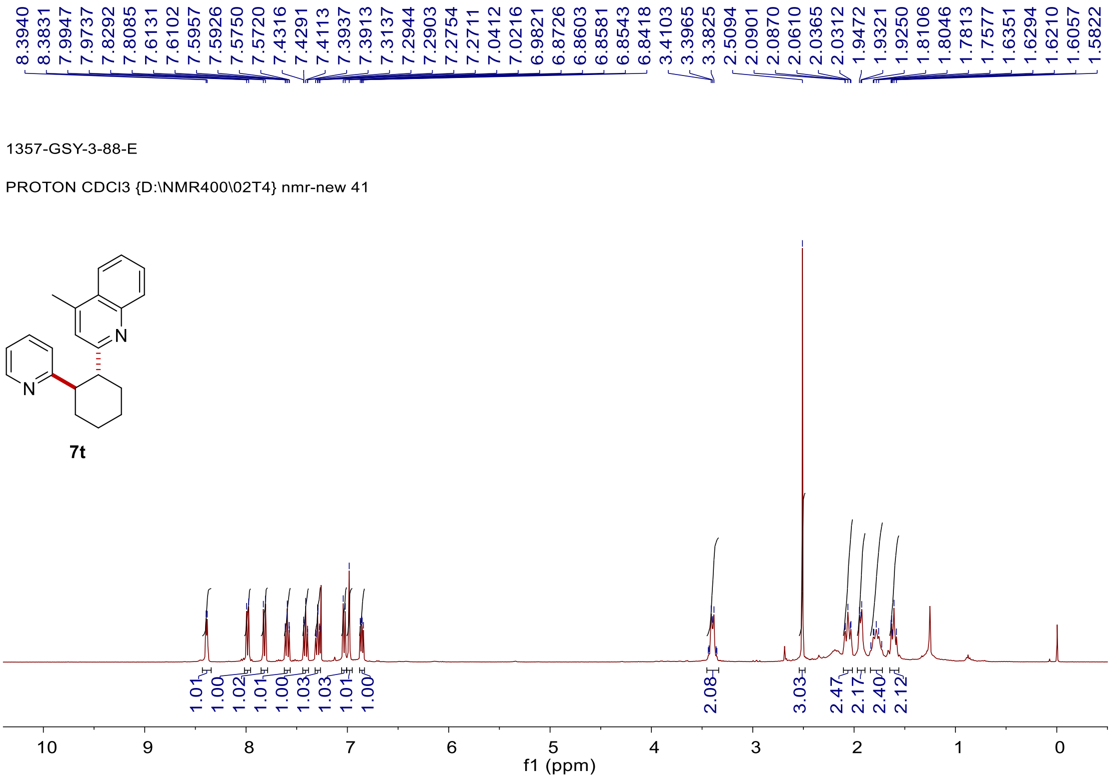

Supplementary Figure 133. ^{1}H NMR spectra of compound **7t**.

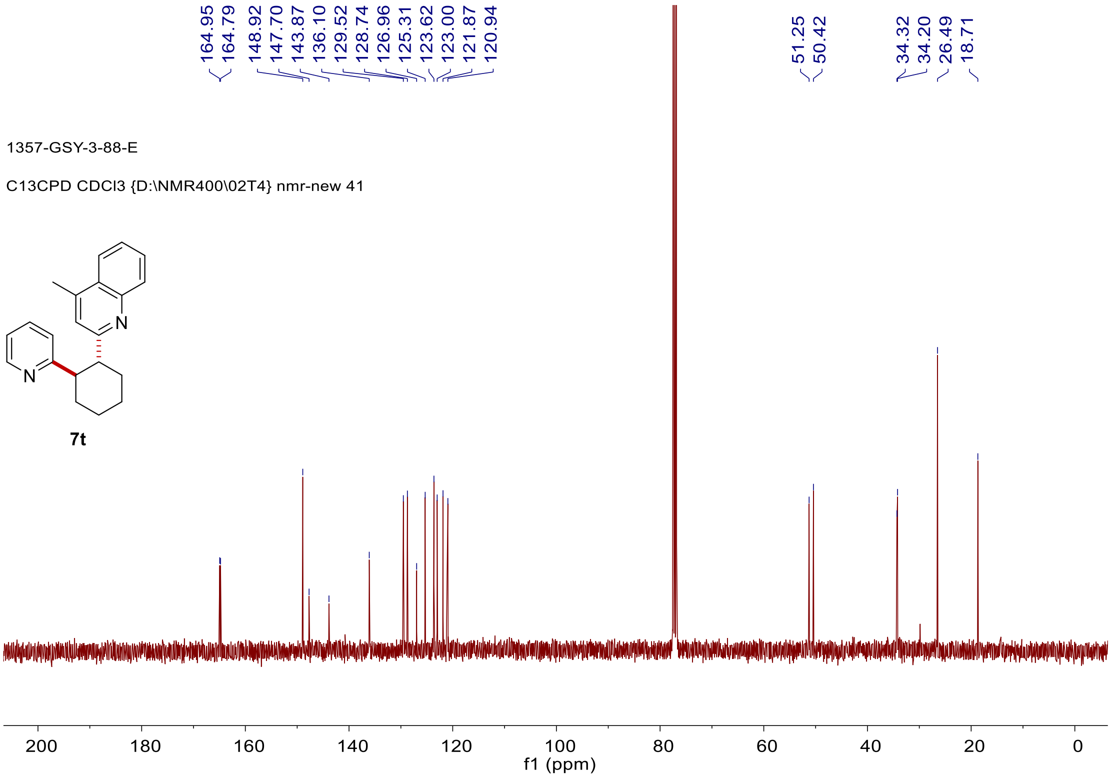

Supplementary Figure 134. ^{13}C NMR spectra of compound **7t**.

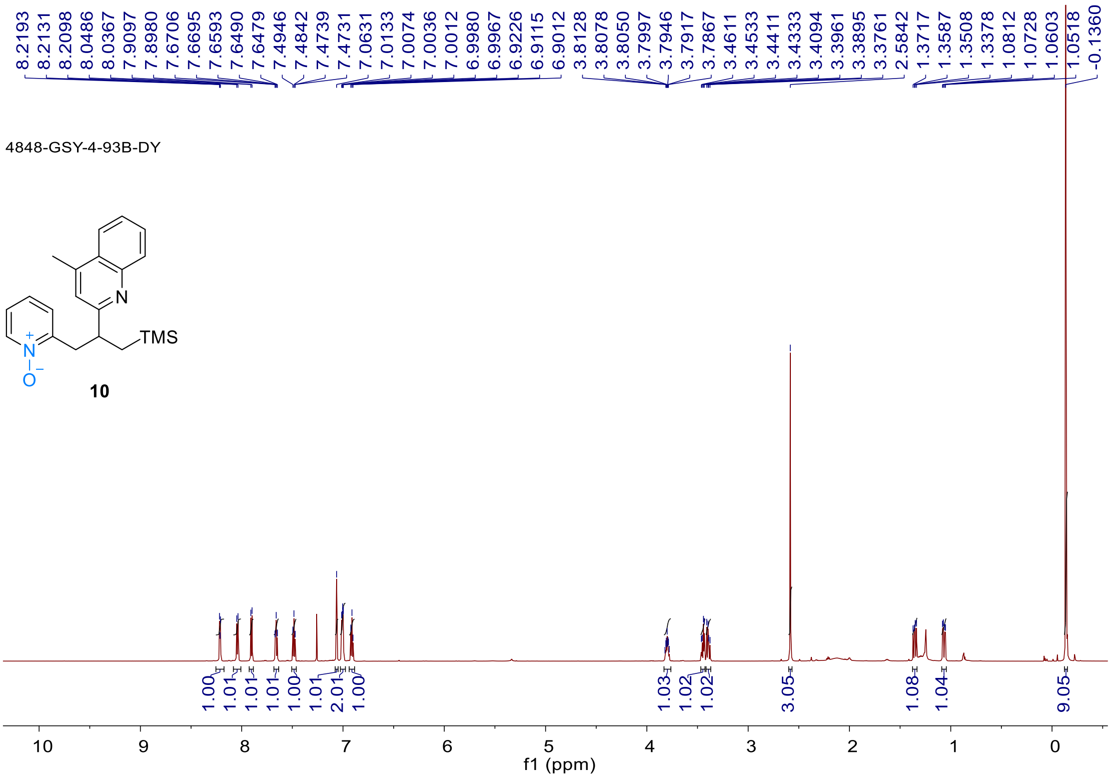

Supplementary Figure 135. ^{1}H NMR spectra of compound **10**.

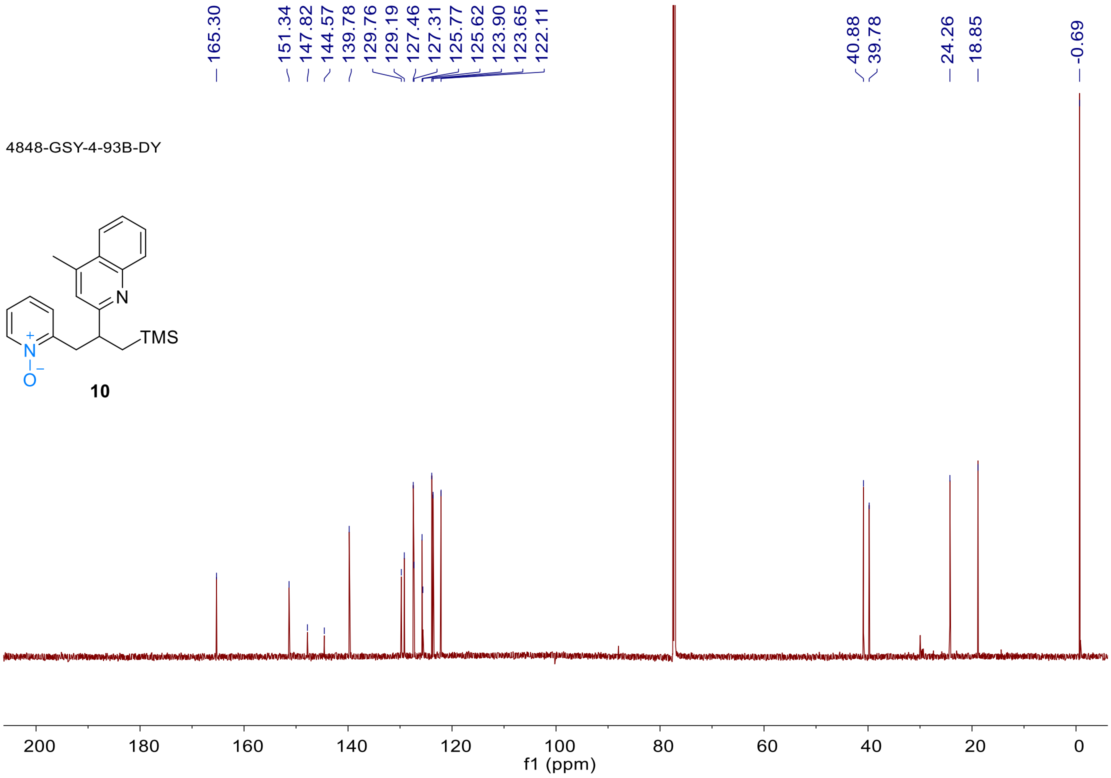

Supplementary Figure 136. ^{13}C NMR spectra of compound **10**.

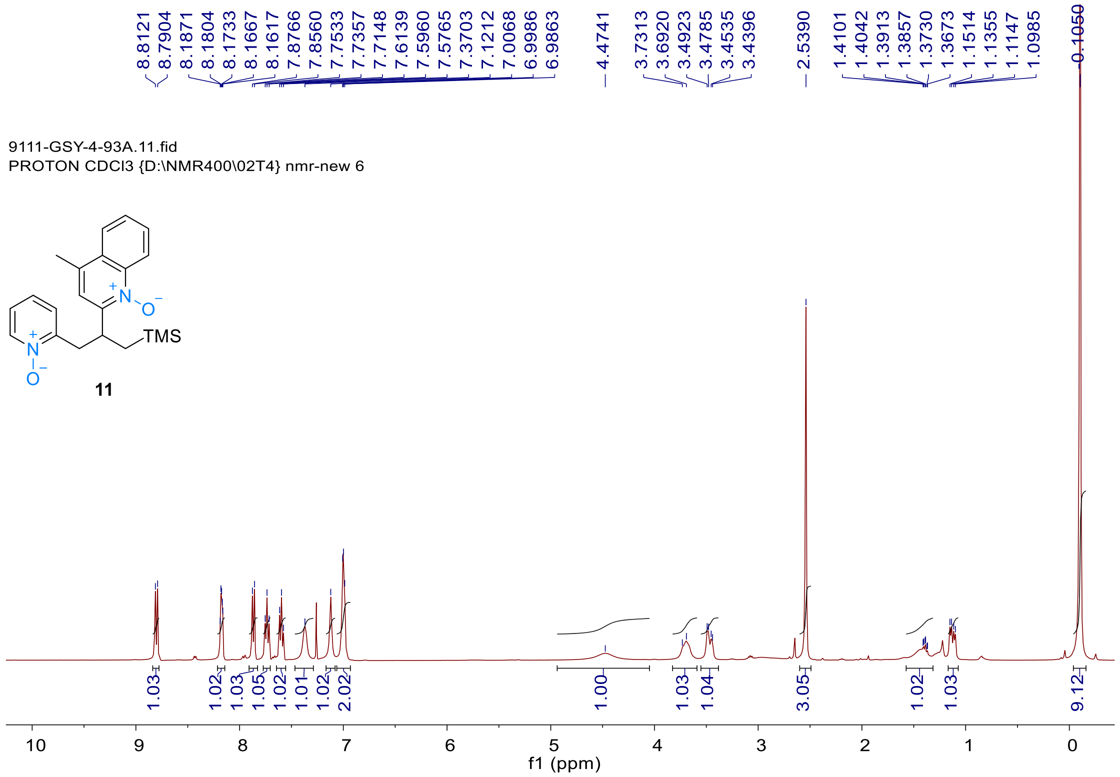

Supplementary Figure 137. ^{1}H NMR spectra of compound **11**.

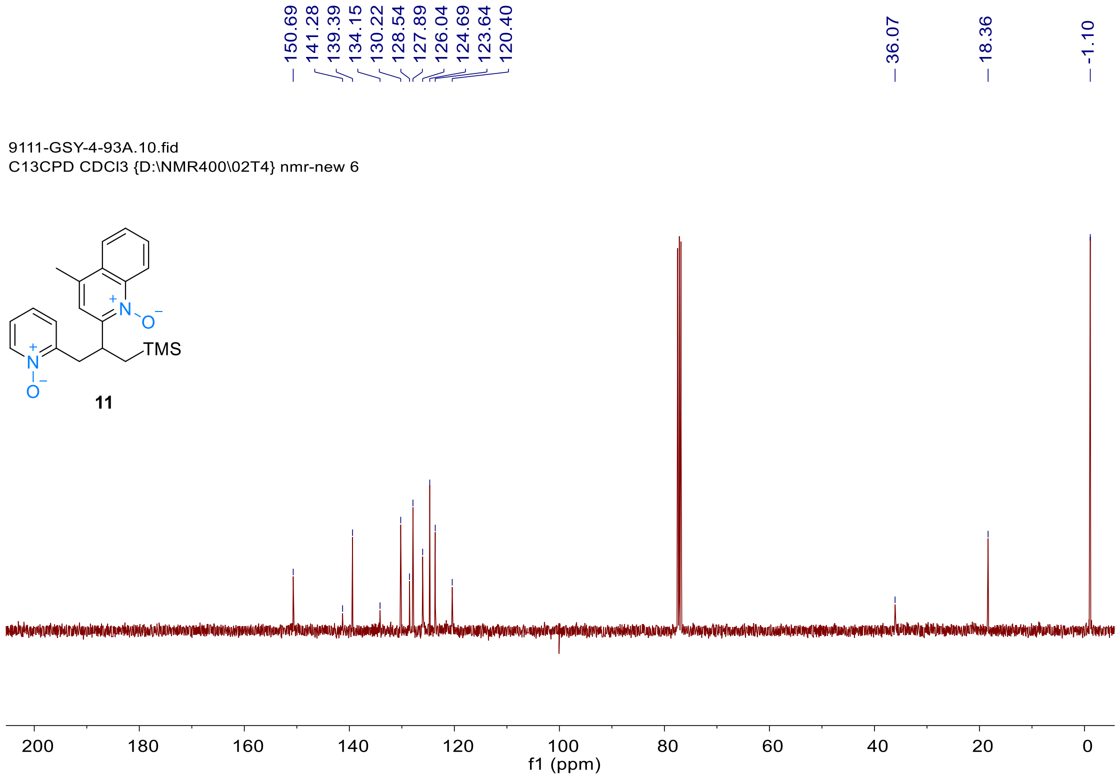

Supplementary Figure 138. ^{13}C NMR spectra of compound **11**.

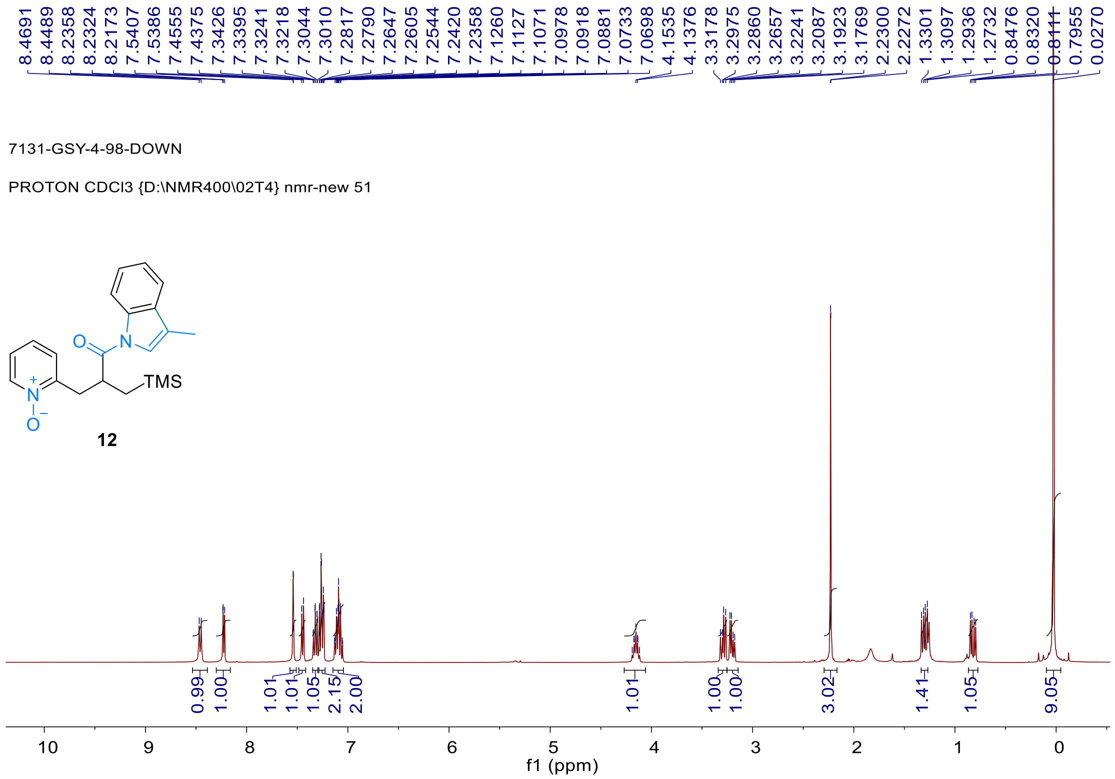

Supplementary Figure 139. ^{1}H NMR spectra of compound **12**.

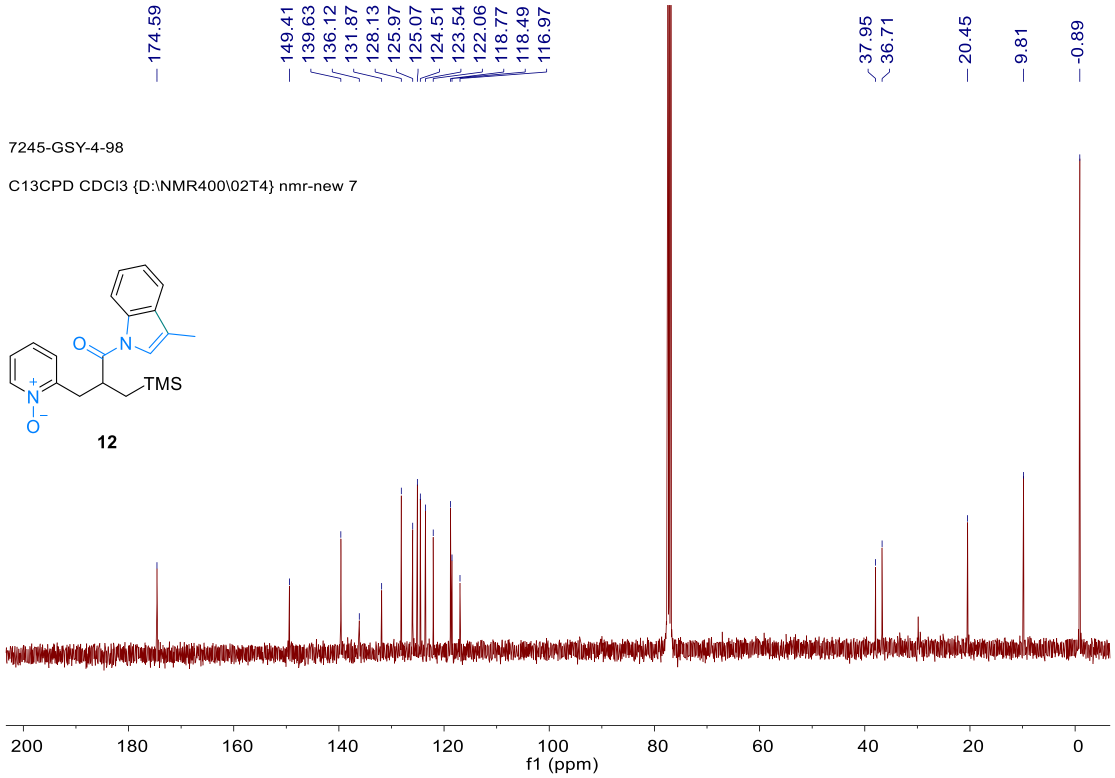

Supplementary Figure 140. ^{13}C NMR spectra of compound **12**.

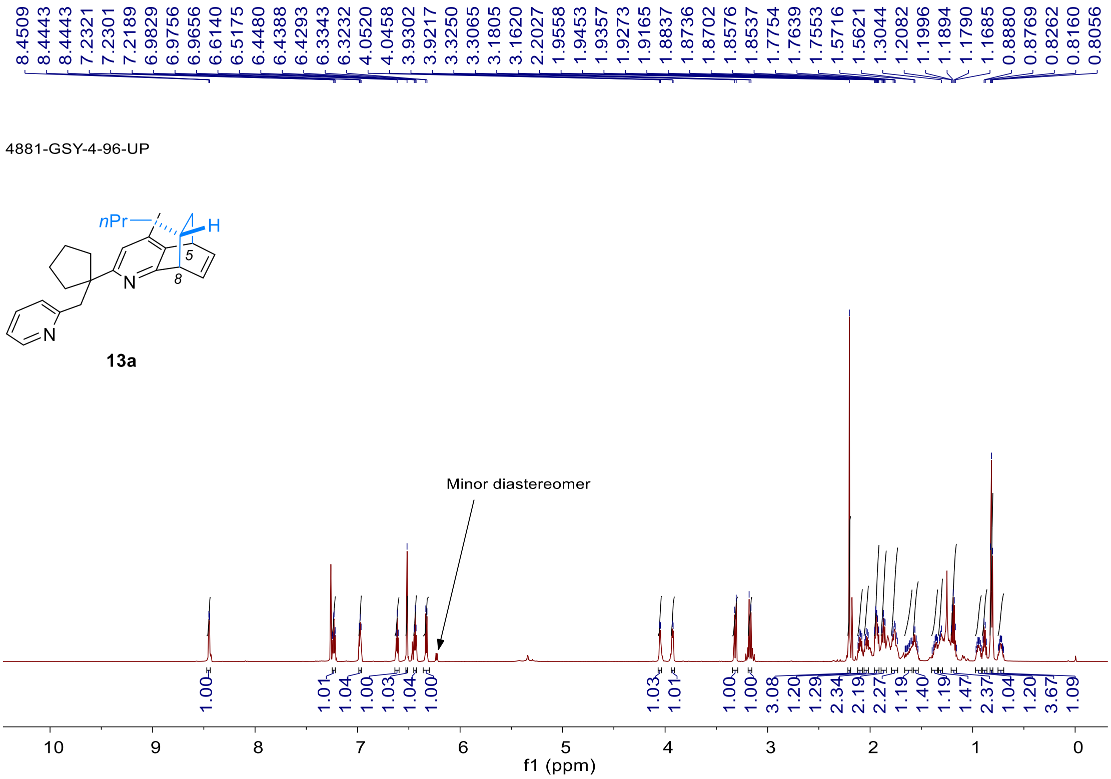

Supplementary Figure 141. ^{1}H NMR spectra of compound **13a**.

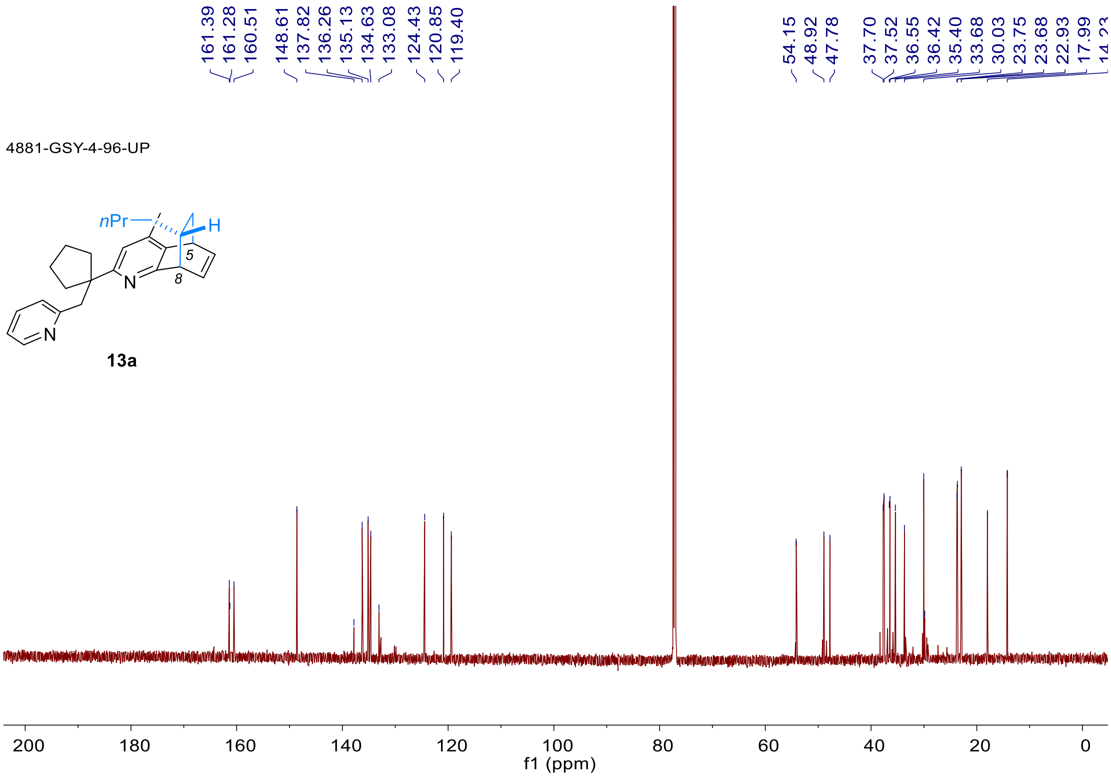

Supplementary Figure 142. ^{13}C NMR spectra of compound **13a**.

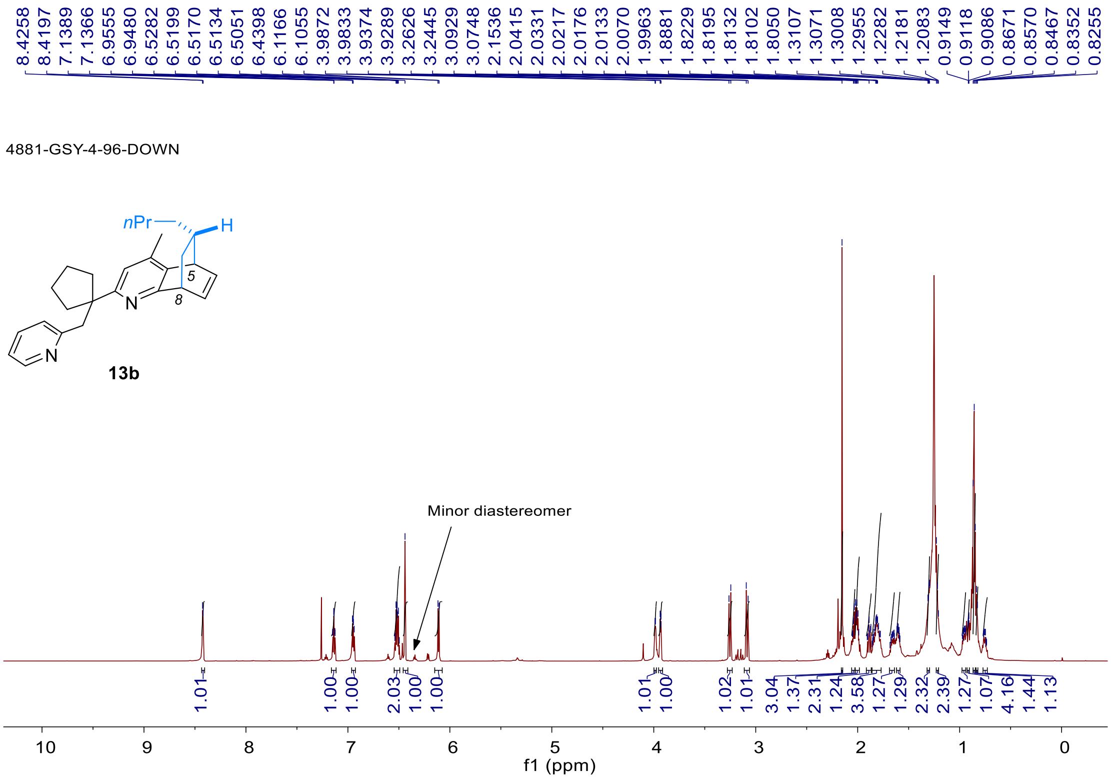

Supplementary Figure 143. ^{1}H NMR spectra of compound **13b**.

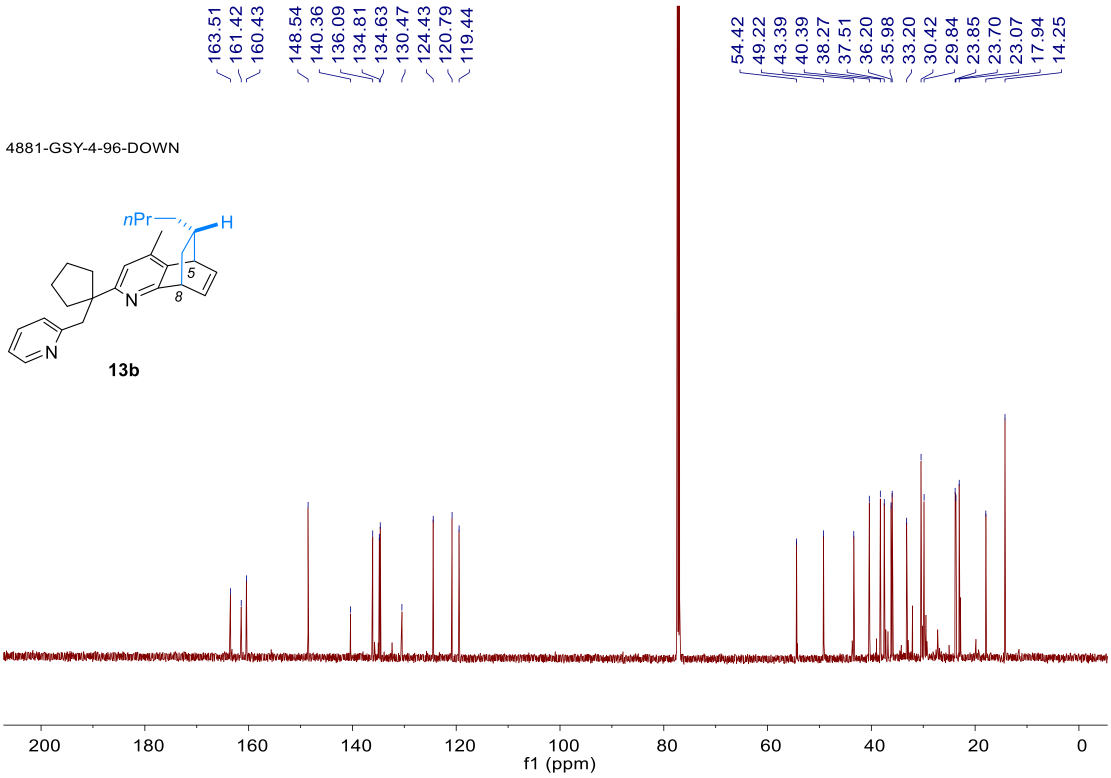

Supplementary Figure 144. ^{13}C NMR spectra of compound **13b**.

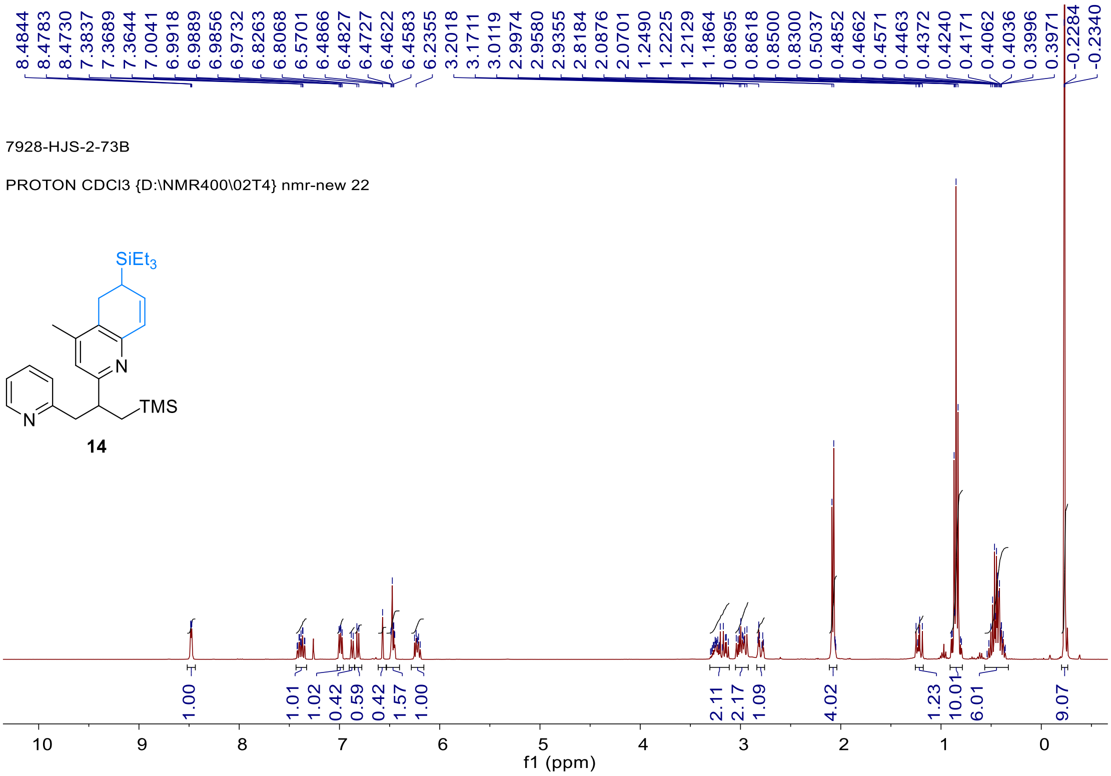

Supplementary Figure 145. ^{1}H NMR spectra of compound **14**.

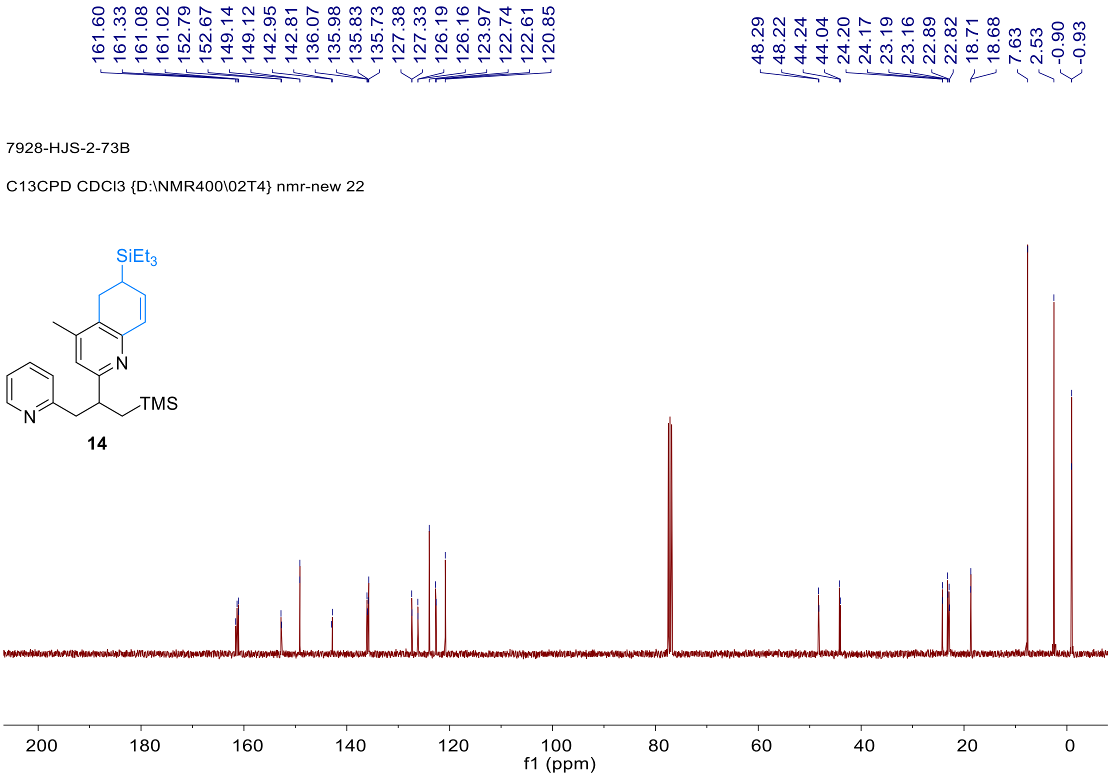

Supplementary Figure 146. ^{13}C NMR spectra of compound **14**.

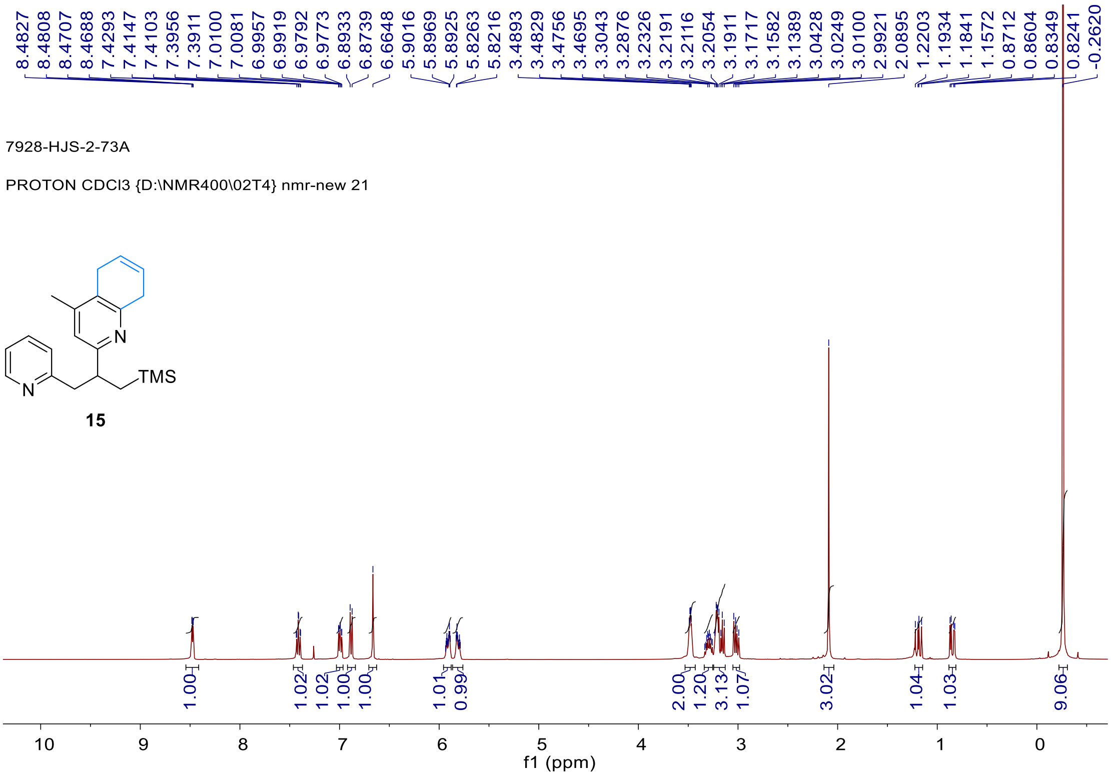

Supplementary Figure 147. ^{1}H NMR spectra of compound **15**.

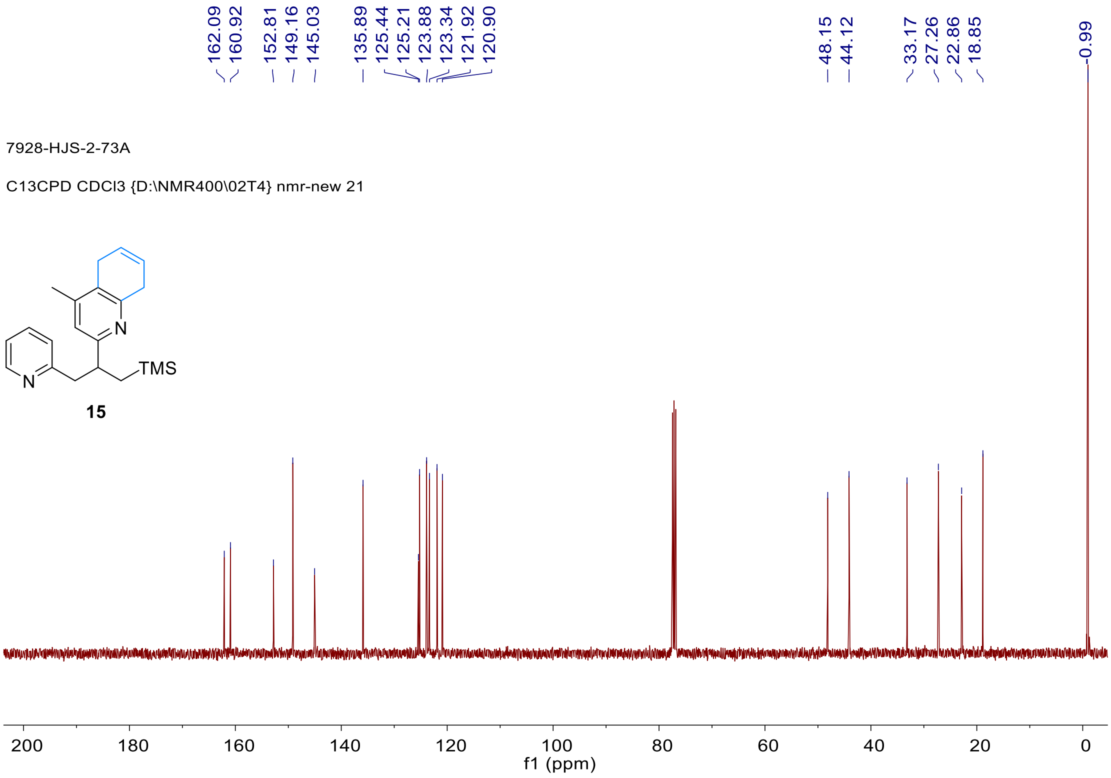

Supplementary Figure 148. ^{13}C NMR spectra of compound **15**.

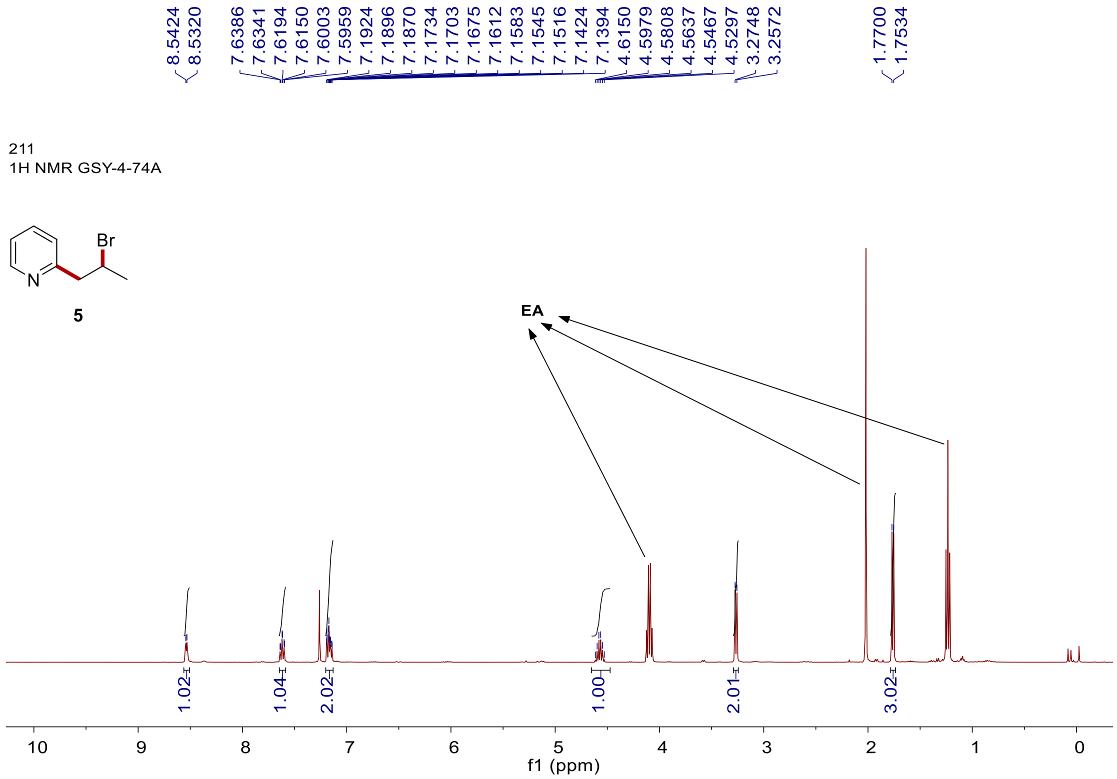

Supplementary Figure 149. ^{1}H NMR spectra of compound **5**.

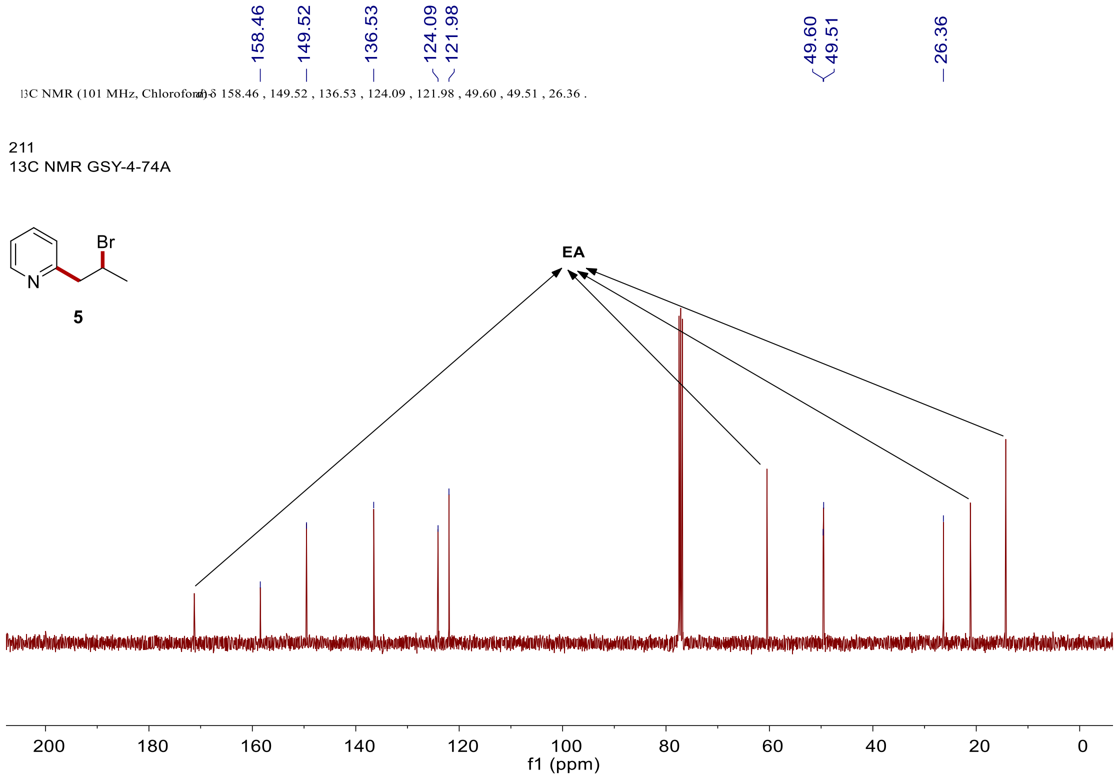

Supplementary Figure 150. ^{13}C NMR spectra of compound **5**.

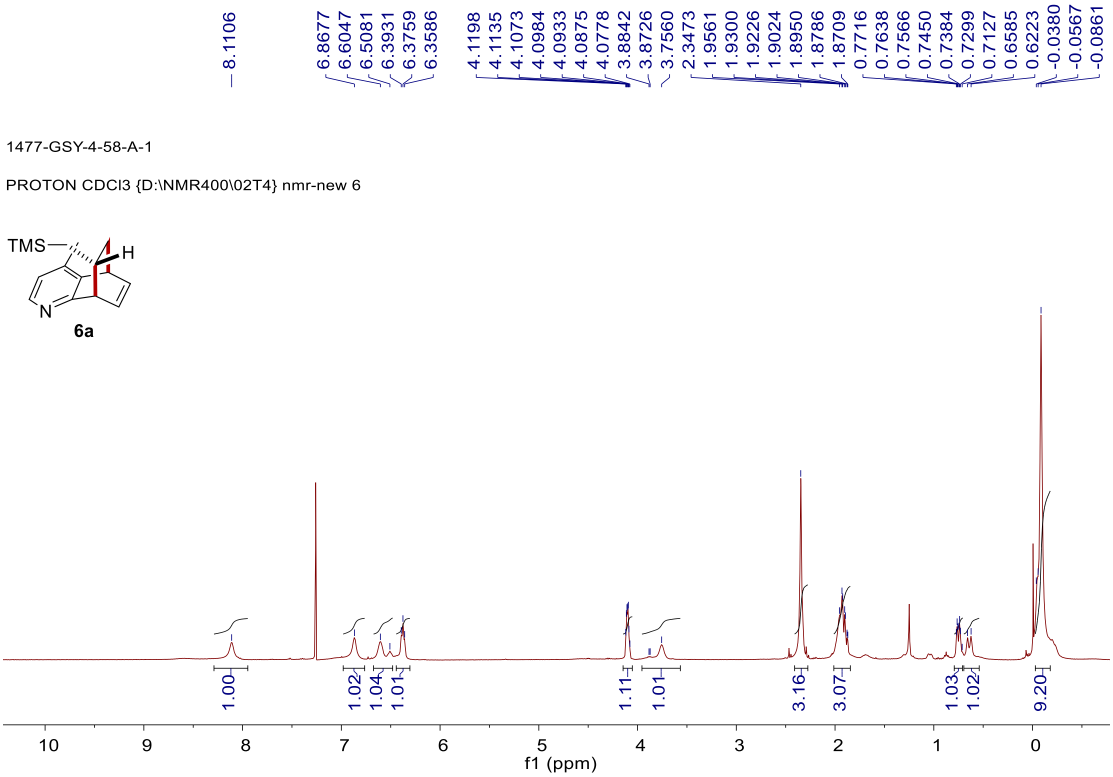

Supplementary Figure 151. ^{1}H NMR spectra of compound **6a**.

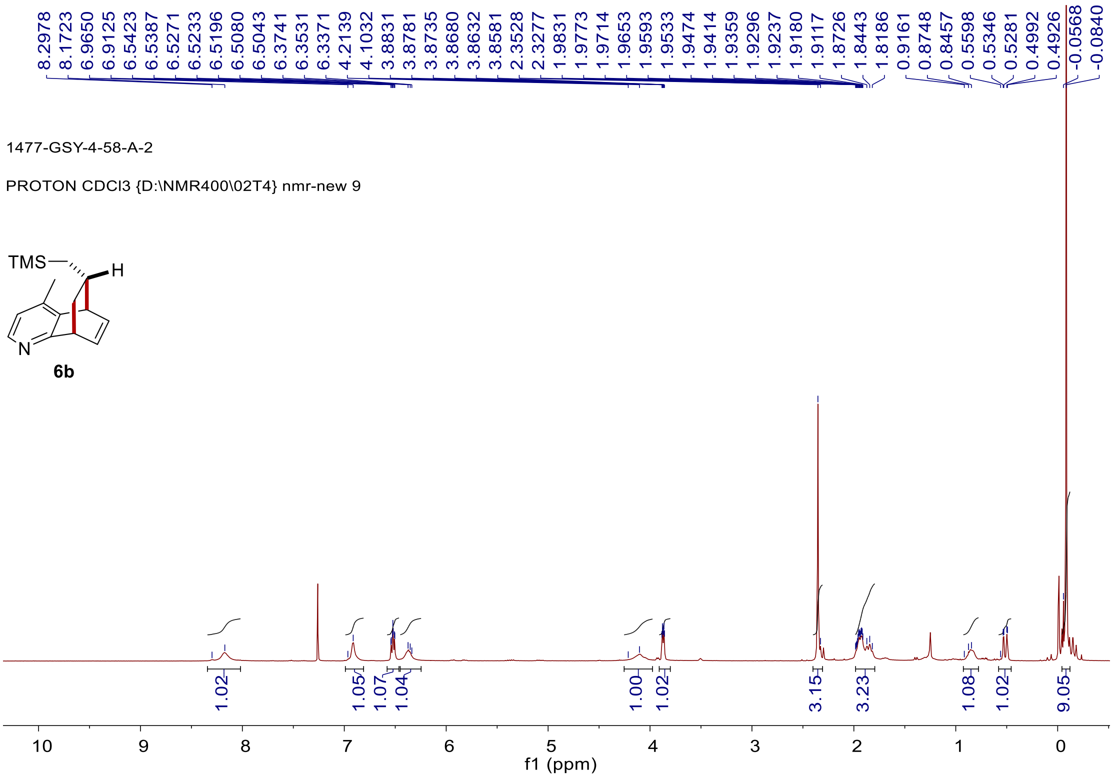

Supplementary Figure 152. ^{1}H NMR spectra of compound **6b**.

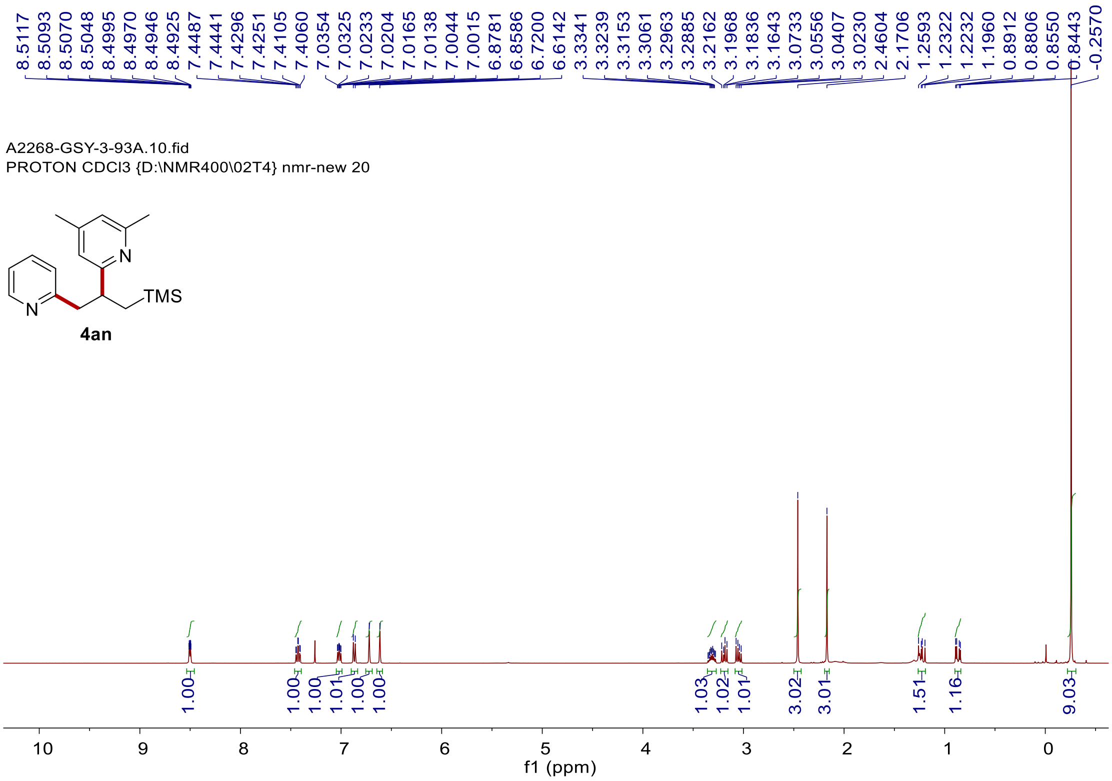

Supplementary Figure 153. ^{1}H NMR spectra of compound **4an**.

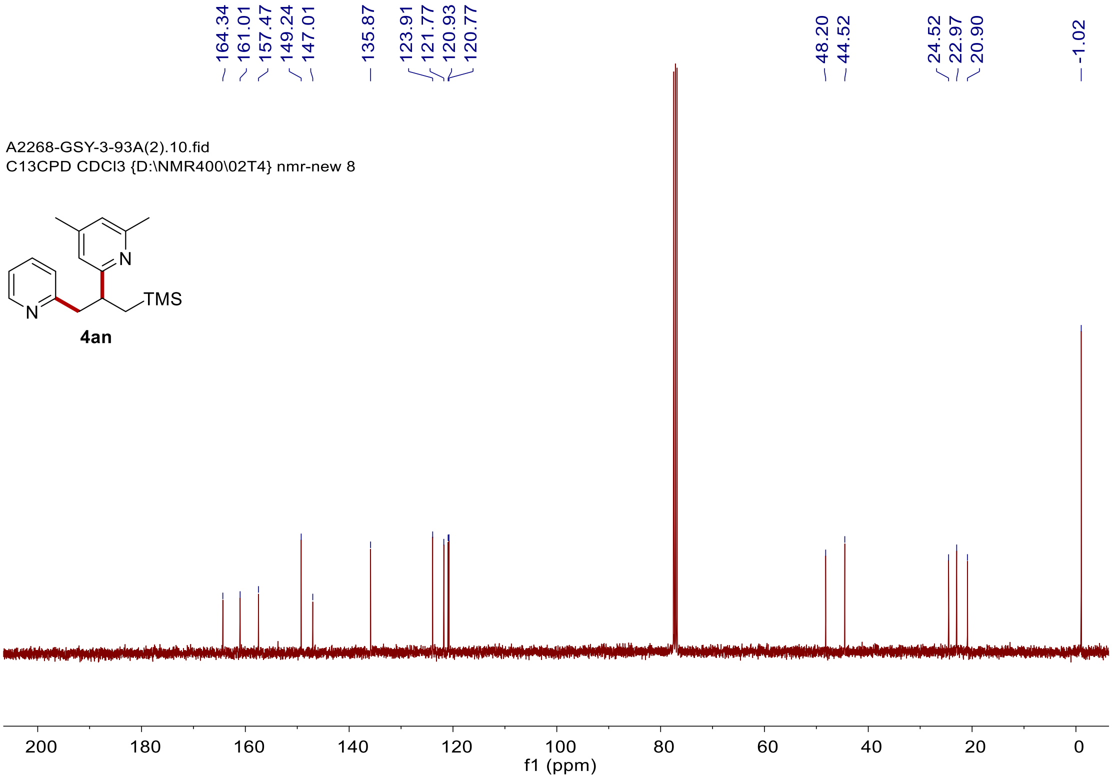

Supplementary Figure 154. ^{13}C NMR spectra of compound **4an**.

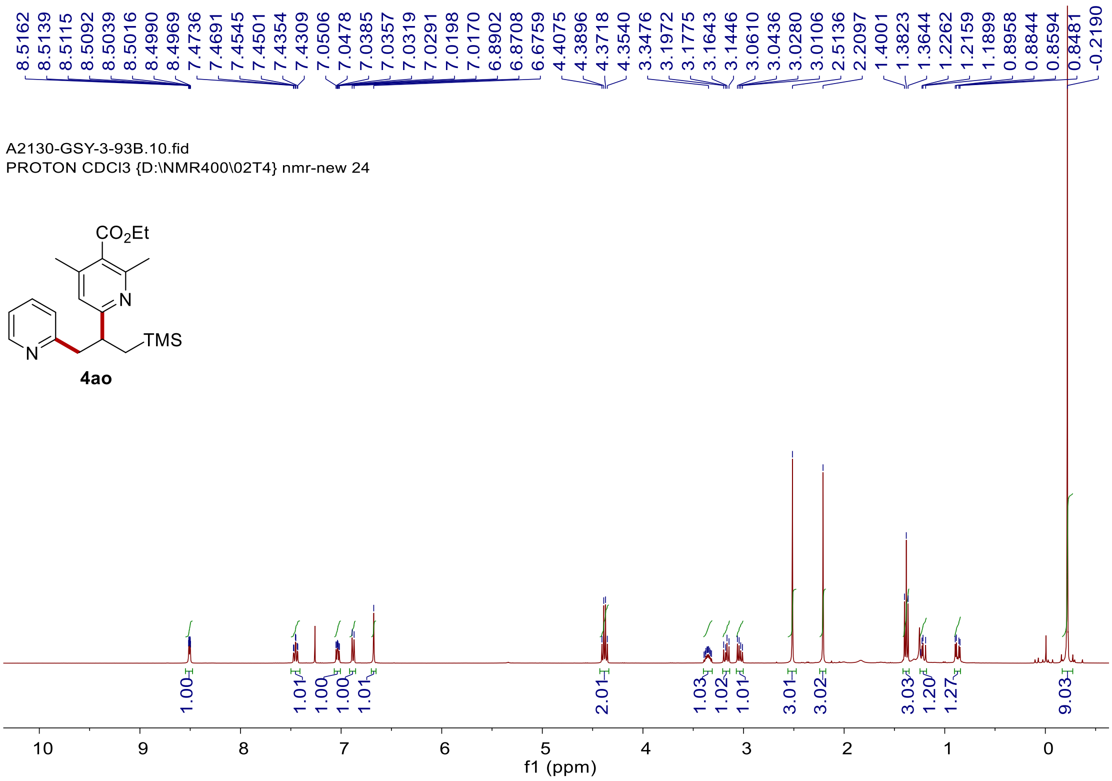

Supplementary Figure 155. ^{1}H NMR spectra of compound **4ao**.

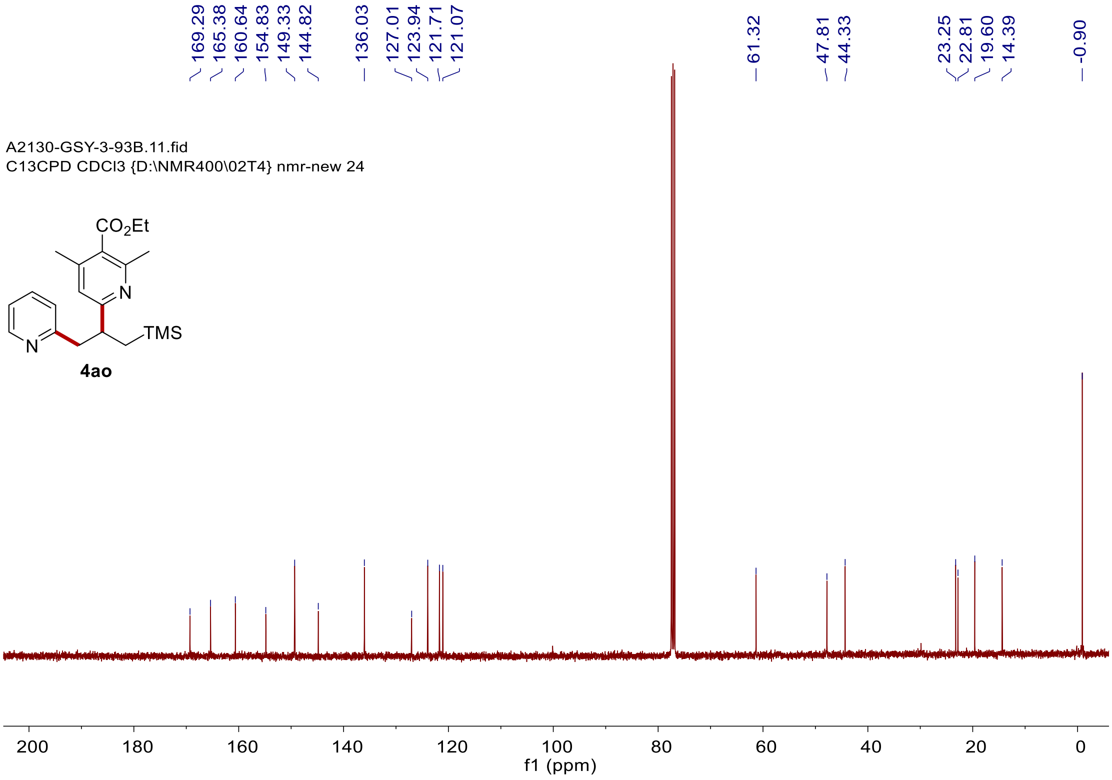

Supplementary Figure 156. ^{13}C NMR spectra of compound **4ao**.

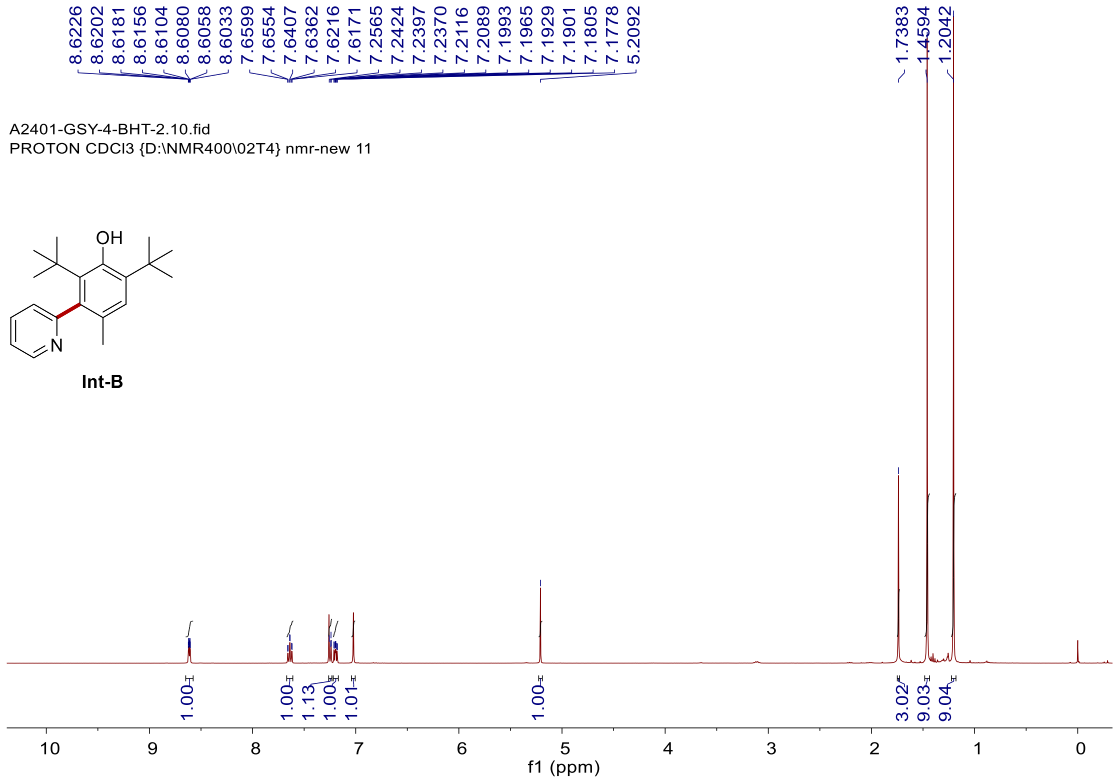

Supplementary Figure 157. ^{1}H NMR spectra of compound **Int-B**.

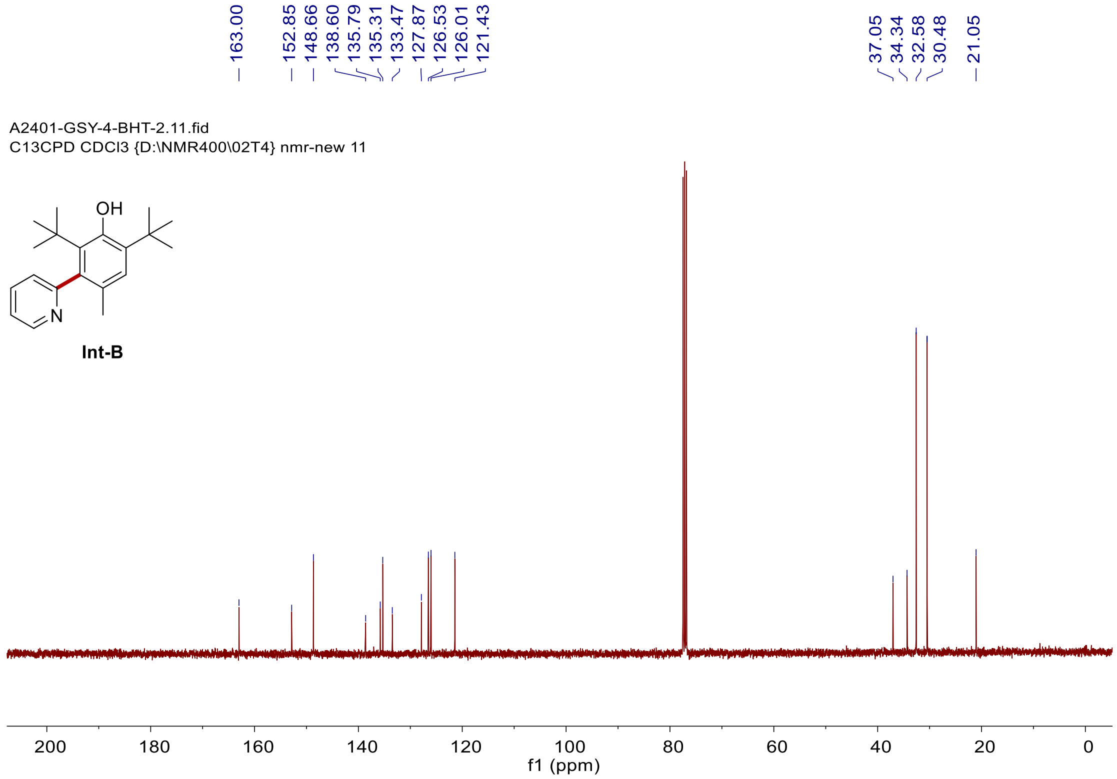

Supplementary Figure 158. ^{13}C NMR spectra of compound **Int-B**.

9. Supplementary Note 7

X-ray crystal structure:

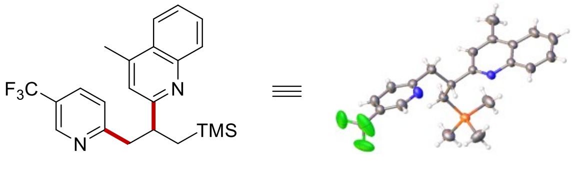

Supplementary Figure 159. Crystal data and structure refinement for **4oa**

Identification code	4oa
Empirical formula	$C_{22}H_{25}N_2F_3Si$
Formula weight	402.53
Temperature/K	293(2)
Crystal system	triclinic
Space group	P-1
a/Å	12.2627(5)
b/Å	12.5746(6)
c/Å	15.2784(8)
α/°	109.019(5)
β/°	91.270(4)
γ/°	96.434(4)
Volume/Å^3	2208.95(18)
Z	4
ρ_{calc}g/cm^3	1.210
μ/mm^{-1}	1.232
F(000)	848.0
Crystal size/mm^3	0.17 × 0.14 × 0.12
Radiation	CuKα (λ = 1.54178)
2Θ range for data collection/°	7.5 to 135.22
Index ranges	-14 ≤ h ≤ 13, -15 ≤ k ≤ 14, -17 ≤ l ≤ 18
Reflections collected	26929
Independent reflections	7833 [R_{int} = 0.0595, R_{sigma} = 0.0417]
Data/restraints/parameters	7833/0/513
Goodness-of-fit on F^2	1.070
Final R indexes [I>=2σ (I)]	R_1 = 0.0789, wR_2 = 0.2332
Final R indexes [all data]	R_1 = 0.0981, wR_2 = 0.2511
Largest diff. peak/hole / e Å^{-3}	0.60/-0.43

10. Supplementary References

[1] K. K. Popov, J. L. P. Campbell, O. Kysilka, J. Hosek, C. D. Davies, M. Pour, P. Kocovsky, *J. Org. Chem.* **2022**, *87*, 920

[2] J. Woo, A. H. Christian, S. A. Burgess, Y. Jiang, U. F. Mansoor, M. D. Levin, *Science* **2022**, *376*, 527.

[3] J. Ma, S. Chen, P. Bellotti, R. Guo, F. Schäfer, A. Heusler, X. Zhang, C. Daniliuc, M. K. Brown, K. N. Houk, F. Glorius, *Science* **2021**, *371*, 1338.

[4] C. Hu, C. Vo, R. R. Merchant, S.-J. Chen, J. M. E. Hughes, B. K. Peters, T. Qin, *J. Am. Chem. Soc.* **2023**, *145*, 25.

[5] M. A. Cismesia, T. P. Yoon, *Chem. Sci.* **2015**, *6*, 5426.

[6] H. J. Kuhn, et al. Pure Appl. Chem. 2004, 76, 2105.

[7] J. N. Demas, W. D. Bowman, E. F. Zalewski, R. A. Velapoldi, *J. Phys. Chem.* **1981**, *85*, 2766.
